# Using RDNA sequences to define dinoflagellate species

**DOI:** 10.1371/journal.pone.0264143

**Published:** 2022-02-25

**Authors:** Brittany M. Ott, R. Wayne Litaker, William C. Holland, Charles F. Delwiche

**Affiliations:** 1 Joint Institute for Food Safety and Applied Nutrition (JIFSAN), University of Maryland—College Park, College Park, MD, United States of America; 2 Cell Biology and Molecular Genetics, University of Maryland—College Park, College Park, MD, United States of America; 3 CSS, Inc. Under Contract to National Oceanic and Atmospheric Administration (NOAA), National Ocean Service, National Centers for Coastal Ocean Science, Beaufort Laboratory, Beaufort, North Carolina, United States of America; 4 National Oceanic and Atmospheric Administration, National Ocean Service, National Centers for Coastal Ocean Science, Beaufort Laboratory, Beaufort, North Carolina, United States of America; IRIG-CEA Grenoble, FRANCE

## Abstract

Dinoflagellate species are traditionally defined using morphological characters, but molecular evidence accumulated over the past several decades indicates many morphologically-based descriptions are inaccurate. This recognition led to an increasing reliance on DNA sequence data, particularly rDNA gene segments, in defining species. The validity of this approach assumes the divergence in rDNA or other selected genes parallels speciation events. Another concern is whether single gene rDNA phylogenies by themselves are adequate for delineating species or if multigene phylogenies are required instead. Currently, few studies have directly assessed the relative utility of multigene versus rDNA-based phylogenies for distinguishing species. To address this, the current study examined D1-D3 and ITS/5.8S rDNA gene regions, a multi-gene phylogeny, and morphological characters in *Gambierdiscus* and other related dinoflagellate genera to determine if they produce congruent phylogenies and identify the same species. Data for the analyses were obtained from previous sequencing efforts and publicly available dinoflagellate transcriptomic libraries as well from the additional nine well-characterized *Gambierdiscus* species transcriptomic libraries generated in this study. The D1-D3 and ITS/5.8S phylogenies successfully identified the described *Gambierdiscus* and *Alexandrium* species. Additionally, the data showed that the D1-D3 and multigene phylogenies were equally capable of identifying the same species. The multigene phylogenies, however, showed different relationships among species and are likely to prove more accurate at determining phylogenetic relationships above the species level. These data indicated that D1-D3 and ITS/5.8S rDNA region phylogenies are generally successful for identifying species of *Gambierdiscus*, and likely those of other dinoflagellates. To assess how broadly general this finding is likely to be, rDNA molecular phylogenies from over 473 manuscripts representing 232 genera and 863 described species of dinoflagellates were reviewed. Results showed the D1-D3 rDNA and ITS phylogenies in combination are capable of identifying 97% of dinoflagellate species including all the species belonging to the genera *Alexandrium*, *Ostreopsis* and *Gambierdiscus*, although it should be noted that multi-gene phylogenies are preferred for inferring relationships among these species. A protocol is presented for determining when D1-D3, confirmed by ITS/5.8S rDNA sequence data, would take precedence over morphological features when describing new dinoflagellate species. This protocol addresses situations such as: a) when a new species is both morphologically and molecularly distinct from other known species; b) when a new species and closely related species are morphologically indistinguishable, but genetically distinct; and c) how to handle potentially cryptic species and cases where morphotypes are clearly distinct but have the same rDNA sequence. The protocol also addresses other molecular, morphological, and genetic approaches required to resolve species boundaries in the small minority of species where the D1-D3/ITS region phylogenies fail.

## Introduction

Dinoflagellates are important constituents of marine and freshwater microbial food webs. There are over 2,400 described species exhibiting diverse autotrophic, mixotrophic, heterotrophic, and parasitic life histories [1–4]. The photosynthetic species contribute significantly to annual productivity, particularly in coastal and shallow water reefs systems [5–7]. A small subset of species (<5%) also form harmful algal blooms that adversely impact human and animal health, disrupt normal ecosystem services, and cause significant economic losses [8]. Due to their ecological and toxicological importance, significant effort has been directed towards defining dinoflagellate species and determining their phylogenetic relationships [9, 10].

Dinoflagellate species are traditionally described based upon morphological differences in the overall size and shape of the cell and, in the case of armored dinoflagellates, the arrangement, size, and shape of thecal plates covering the cell surface [2, 11, 12]. Species with indistinguishable morphologies are sometimes differentiated based on differences in the structure of their organelles or other internal features. These ultrastructural characteristics, along with the overall cell morphology, have also been used as a basis for inferring the evolutionary relationships among the major dinoflagellate lineages (prorocentroid, dinophysoid, gonyaulacoid, peridinioid and gymnodinoid) [13, 14]. Beginning in the 1990s, DNA sequence data for certain genes from single-cell dinoflagellate isolates began to be sequenced at an ever-increasing pace, and thus became available for phylogenetic study. These phylogenies were most commonly based upon ribosomal (rDNA) genes with mitochondrial, cytochrome b, mitochondrial cytochrome c oxidase 1, heat shock protein 90 (hsp90), and others being used as well [15–20]. Most of these genes, other than the rDNA genes, proved uninformative with respect to differentiating dinoflagellate species. Plastid genes such as *rbcL* (encoding the large subunit of ribulose-1,5-bisphosphate carboxylase/oxygenase), which have been widely used for other phototrophic lineages, are problematic for dinoflagellates because of the existence of numerous non-photosynthetic species, independent acquisition of plastids in several lineages, and the peculiar nature of the peridinin-type dinoflagellate plastid genome and its *rbcL* gene [21, 22].

The molecular phylogenies based on rDNA genes appeared to be the most reliable for determining phylogenetic relationships among dinoflagellates, but even they sometimes yielded terminal clades (i.e., species) that did not correspond with those defined based on morphological characters [21, 22]. Similarly, sequence-based phylogenies also indicated different relationships at higher phylogenetic levels than inferred from morphology alone [13, 23, 24]. This led to now widely-accepted insights such as the recognition that the gymnodinioid form has evolved independently several times, and that prorocentroids reflect a derived rather than ancestral morphology [11]. However, there was little agreement regarding relationships among higher level taxa in different analyses, particularly those based solely on rDNA data. It soon became evident that no single gene, or combination of several commonly-used genes, consistently resolved the evolutionary relationships among the major dinoflagellate lineages. There was simply insufficient signal in the available single-gene sequences to identify reliable branching patterns among the deeper lineages.

Despite the failure to resolve the deeper phylogenetic branches, there is abundant evidence suggesting single gene phylogenies based on rDNA genes are useful in distinguishing closely-related species that morphology along cannot fully resolve [25–36]. These single-gene phylogenies have also revealed the existence of numerous morphologically indistinguishable (cryptic) species. The utility of the rDNA gene segments in particular for delineating species is unsurprising given that ribosomal genes have been used to differentiate related metazoan species [37], as well as apicomplexan and other protozoan species [38]. Despite this evidence demonstrating that ribosomal genes can serve as diagnostic criteria for describing new species, concern exists regarding whether the ITS/5.8S, D1-D3, D8-D10 LSU or SSU rDNA genes alone are sufficient for distinguishing species of dinoflagellates [39, 40]. It is logical to postulate that expansive multigene phylogenies, compared to those based on a single gene or even handful of genes, will yield a stronger statistical estimate of species boundaries as well as phylogenetic relationships. The greatly decreased cost of DNA/RNA sequencing now makes it feasible to compare the phylogenies found using a wide variety of datasets, including both rDNA sequences and multiple protein-coding genes.

The additional power of using multigene phylogenies to resolve phylogenetic relationships among dinoflagellate species was demonstrated by the work of Janouškovec *et al*., 2017 [41]. They aligned 101 genes obtained from transcriptomes of 43 protist species, including 26 dinoflagellate species, generating well-supported phylogenies that, for the first time, confirmed dinoflagellates are indeed monophyletic and determined relationships among many major dinoflagellate lineages. This work largely resolved several long-standing controversies regarding the evolution of the group, including placement and monophyly of prorocentroids, alliances of several gymnodinioid lineages, and placement of *Noctiluca*. The present study extends the work of Janouškovec *et al*. (2017) to address whether single gene D1-D3 or ITS/5.8S rDNA phylogenies yielded the same species-specific terminal clades within the single genus *Gambierdiscus* as revealed using multigene phylogenies. Whether the multigene phylogeny performed better at resolving the phylogentic relationship among the various species was also examined.

We chose the dinoflagellate genus *Gambierdiscus* as a test case due to its importance in seafood safety. Certain species produce potent neurotoxins known as ciguatoxins, which bioaccumulate in the food webs and lead to ciguatera fish and shellfish poisoning (CP), globally the largest cause of non-bacterial seafood poisoning [42–45]. Having a clear understanding of which species are present in a region and which are making toxin is critical to better understanding and predicting CP outbreaks [46–48]. Consequently, extensive taxonomic work on the genus has been conducted, generating a large dataset of rDNA sequences useful in comparative analyses of rDNA vs. multigene phylogenies [34, 47, 49–56].

Results from this study showed both the rDNA and multigene phylogenies resolved *Gambierdiscus* species boundaries equally well but that multigene phylogenies provided stronger evidence for the phylogenetic relationship among species. To further investigate the extent to which rDNA sequences could resolve other dinoflagellate species, the study was extended to include a literature survey of 323 genera representing 863 described species (473 published studies) to determine how well species in a given genus was resolved based on either SSU, ITS/5.8S or D1-D3 phylogenies. The survey revealed that the combination of D1-D3 and ITS/5.8S phylogenies were capable of resolving 97% of the species surveyed.

## Methods

### Cell cultures

The *Gambierdiscus* isolates screened in this study were obtained either from the National Centre for Marine Algae and Microbiota (East Boothbay, Maine, USA; formally CCMP) or from single cell isolates collected at locations throughout the Caribbean, Gulf of Mexico or tropical Pacific. The cultures analyzed were *Gambierdiscus* sp. ribotype 2 (isolate NCMA 1655), *G*. *australes* (isolate NCMA 1653), *G*. *belizeanus* (isolate NCMA 399), *G*. *caribaeus* (isolate NCMA 1733), *G*. *carolinianus* (isolate Kenny 6), *G*. *excentricus* (isolates Bahamas Gam 5 and Pulley’s Ridge Gam 2), *G*. *pacificus* (isolate NCMA 1650), *G*. *ruetzleri* (isolate WH55 Gam 4), and *G*. *silvae* (isolate Curacao Gam 11). These isolates were cultured, maintained, and collected as described in Litaker *et al*., 2017 [46]. Briefly, cells were cultured in a Percival Scientific incubator (Perry, IA, USA) maintained at 27˚C with a 12:12 h light:dark cycle. Photosynthetically-active radiation (PAR) was maintained at 90–100 μmol photons m-2 s^-1^ (Full Spectrum Solutions, Jackson, MI, USA). Growth medium consisted of 0.2 μm filtered Gulf Stream seawater (salinity 33) in 250 mL tissue culture flasks with vented caps (BD Biosciences, Bedford, MA, USA). Vitamins and nutrients were added according to a modified K-medium protocol [57–59]. Cells were counted every three to four days using a Beckman Coulter Multisizer™ 3 particle counter (Beckman Coulter Inc., Brea, CA) equipped with a 280 μm aperture. Samples were mixed thoroughly to ensure the cells were evenly distributed prior to counting [58]. Cell densities were maintained at relatively low levels (250 to 1000 cells mL^-1^) to avoid nutrient or CO_2_ limitation. Cultures were diluted with fresh media as needed to maintain cells in continual log phase growth [58]. When a sufficient culture volume was obtained, cells were harvested by collecting 350,000 to a 1,000,000 cells on a 20 μm sieve and washing them with filtered seawater into a 50 mL centrifuge tube. These cells were then pelleted using centrifugation at 3200 x *g* for 10 min, and the supernatant carefully decanted. One mL of RNA later was added to each cell pellet and frozen overnight –20˚C, then placed in -80°C until shipment.

### Sequencing

Samples were processed by adding *Gambierdiscus* cells from each single-cell isolate to separate RNase-free cryotubes containing 350 mg of 1 mm Zirconia beads (cat. 1107110ZX) and homogenized using a bead-beater (Mini-Beadbeater, BioSpec Products) for two minutes. The RNA was then extracted using the Macherey-Nagel RNA Plant Isolation kit (Macherey-Nagel, Bethlehem, PA; ref. 740949.50) as per the manufacturer’s instructions, and DNA was removed using the included DNase. Initial RNA integrity was examined on an agarose gel and DNase removal was confirmed using PCR with dinoflagellate-specific PCR primers (EukA: 5’-AACCTGGTTGATCCTGCCAGT-3’; DinoR: 5’-TTATTCACCGGAWCACTCAATCGG; [59, 60]). Prior to sequencing, quality and quantity was determined using the Agilent 2100 Bioanalyzer (Agilent, Santa Clara, CA).

Sequencing was performed in one of two ways: submission of total RNA to the University of Maryland-College Park Institute for Bioscience and Biotechnology Research (IBRR; College Park, MD)(the two *Gambierdiscus excentricus* samples, *Gambierdiscus belizeanus*, and *Gambierdiscus carolinianus*) where the libraries were prepared by IBRR, or the RNA was processed in-house with completed libraries submitted to the University of Maryland Institute for Genomic Sciences (IGS; Baltimore, MD)(*Gambierdiscus australes*, *Gambierdiscus ruetzleri*, *Gambierdiscus pacificus*, *Gambierdiscus caribaeus*, *Gambierdiscus* sp. ribotype 2, and *Gambierdiscus silvae*). For samples where the completed libraries were submitted to IGS, cDNA was synthesized using the SmartSeq v4 RNA kit for Sequencing (Takara Bio USA, Inc., Mountain View, CA), as per the manufacturer’s instructions, using 10 ng of total RNA and 8 cDNA amplification cycles. Subsamples of cDNA from each species were taken and processed using Covaris shearing. Libraries for sheared and not-sheared samples were prepped using the Nextera XT DNA Library Prep Kit (Illumina, San Diego, CA), as per the manufacturer’s instructions, using the Index Primer combinations found in S1 Table in S1 File.

The four samples that were submitted to IBBR in College Park, MD were run on a single Hi-Seq1000 lane using 100 bp, paired-end read chemistry. The twelve samples (six species, each with a sheared and a not-sheared subsample) that were submitted to IGS in Baltimore, MD were run on an additional Hi-Seq4000 lane using 150 bp, paired-end chemistry.

### Initial data processing

The raw data was processed by first examining the read quality using FastQC (Babraham Bioinformatics, Babraham Institute, Cambridge, UK) and if trimming was required (i.e. trailing end base pair quality, presence of adapter sequences, etc.), the reads were then passed through Trimmomatic (v. 0.36; [61]). After this initial examination of the data, the *G*. *australes* and *G*. *ruetzleri* transcriptomes were determined to be of insufficient quality (i.e. degraded sequence data) for use in this study and thus were removed from further analyses. The data for the remaining transcriptomes was assembled into contigs using rnaSPAdes (v. 3.11.1; [62]) and any contigs less than 200 bp were eliminated from further study (see Table 1). In addition to the raw data obtained from this study, transcriptomic data from the following sources was processed and incorporated as well: two libraries each of *Gambierdiscus* sp. ribotype 2, culture collection “1655”, two each of *Gambierdiscus* sp. ribotype 2 from the environmental isolate “Mixed PR”. These four samples were sequenced previously at the University of Maryland-College Park IBBR using 100 bp paired-end chemistry on one HiSeq1000 lane, although the data was not bioinformatically processed until this study. Raw data publicly available at NCBI was also used in this study (see Table 2), utilizing the same bioinformatics procedures indicated above.

**Table 1:** Transcriptome statistics.

	Initial Assembly			> 200 bp contigs			Raw Reads		Trimmed Reads	
Transcriptome*	N50	G/C	Total #	N50	G/C	Total #	G/C	Total #	G/C	Total #
*Alexandrium catenella*	1005 bp	66%	150,099	1076 bp	66%	112,672	63%	21,397,132	N/A	N/A
*Alexandrium monilatum*	1352 bp	66%	128,383	1400 bp	66%	101,744	64%	33,579,814	N/A	N/A
*Gambierdiscus australes*	1075 bp	61%	130,919	1148 bp	61%	98,898	54%	29,128,195	N/A	N/A
***Gambierdiscus belizanus***	1532 bp	61%	145,496	1610 bp	61%	105,152	60%	46,931,124	N/A	N/A
***Gambierdiscus caribaeus***	265 bp	55%	906,425	265 bp	55%	903,720	50%	36,936,740	50%	36,936,739
***Gambierdiscus carolinianus***	1609 bp	61%	142,860	1676 bp	61%	107,602	60%	59,417,493	N/A	N/A
***Gambierdiscus excentricus* (1)**	1562 bp	62%	133,230	1629 bp	62%	100,698	61%	46,936,012	N/A	N/A
***Gambierdiscus excentricus* (2)**	1579 bp	62%	138,159	1655 bp	62%	101,031	61%	49,081,709	N/A	N/A
*Gambierdiscus excentricus*	1354 bp	62%	157,017	1416 bp	62%	123,461	60%	135,064,950	N/A	N/A
***Gambierdiscus pacificus***	595 bp	54%	411,958	602 bp	54%	407,581	53%	56,780,145	53%	56,780,141
*Gambierdiscus polynesiensis*	1166 bp	61%	310,864	1285 bp	61%	227,927	60%	1,371,510,284	N/A	N/A
***Gambierdiscus sp*. ribotye 2** (**R2)**	271 bp	54%	844,803	271 bp	54%	841296	52%	44,656,329	53%	44,656,321
***Gambierdiscus sp*. R2 NCMA1655 (1)**	1260 bp	58%	291,995	1447 bp	59%	184,886	60%	61,870,415	N/A	N/A
***Gambierdiscus sp*. R2 NCMA1655 R2 (2)**	1211 bp	57%	311,122	1414 bp	58%	192,648	60%	53,905,214	N/A	N/A
***Gambierdiscus sp*. R2 Mixed PR (1)**	1282 bp	57%	280,735	1508 bp	59%	162,308	60%	55,148,193	N/A	N/A
***Gambierdiscus sp*. R2 Mixed PR (2)**	1613 bp	61%	157,668	1713 bp	61%	105,802	60%	63,383,135	N/A	N/A
***Gambierdiscus silvae***	267 bp	54%	1,225,491	297 bp	54%	1,223,149	50%	21,369,907	50%	21,369,905
*Neoceratium fusus*	1329 bp	54%	159,185	1385 bp	54%	125,643	53%	31,515,157	N/A	N/A
*Pyrodinium bahamense*	1270 bp	64%	172,941	1328 bp	64%	136,557	61%	31,354,710	N/A	N/A

*Sequences in **bold** indicate those first presented in this paper; the numbers in parentheses (1 and 2) indicate duplicate samples; “Mix.” indicates Mixed PR, environmental samples; “1655” is a sample from culture collection.

**Table 2:** Publicly available transcriptomes used in this phylogenetic study.

Organism Name	Strain	Sample Name	SRA Identifier	Read Length*
***Alexandrium catenella***	OF101	MMETSP0790	SRR1296704	50 bp PE
***Alexandrium monilatum***	CCMP3105	MMETSP0097	SRR1296898	50 bp PE
***Gambierdiscus australes***	CAWD 149	MMETSP0766_2	SRR1296893	50 bp SE
***Gambierdiscus excentricus***	VGO790	N/A	SRR3348983	100 bp PE
***Gambierdiscus polynesiensis***	CAWD 212	N/A	SRR3358210	100 bp SE
***Neoceratium fusus***	PA161109	MMETSP1075	SRR1300301	50 bp PE
***Pyrodinium bahamense***	pbaha01	MMETSP0796	SRR1296702	50 bp PE

* PE indicates Paired End reads; SE indicates Single End reads.

### Gambierdiscus single gene region phylogenies

#### D1 to D3 large ribosomal subunit region phylogeny

**Fig 1:**
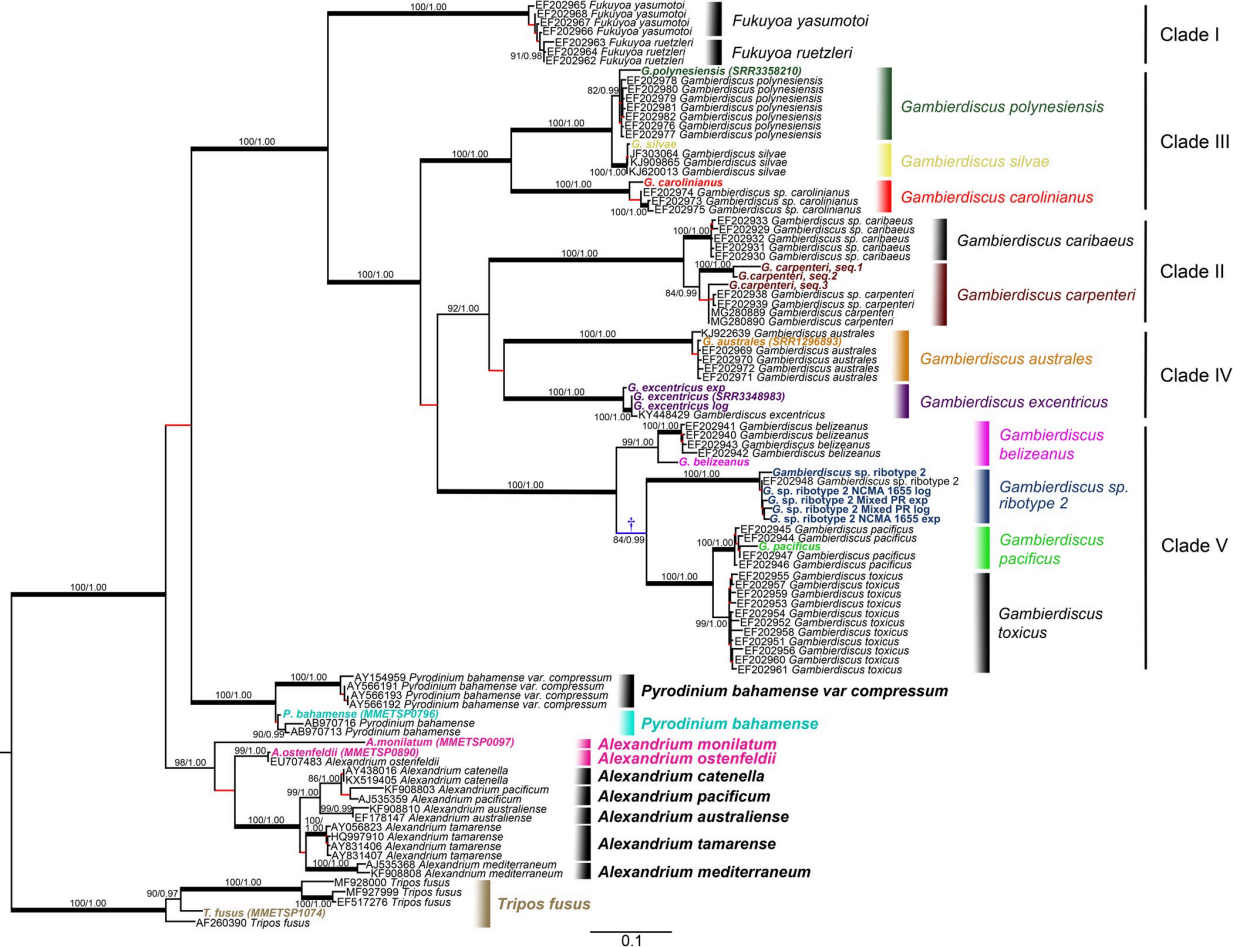
Phylogenetic tree of *Gambierdiscus* using the large ribosomal subunit, D1 to D3, region. Maximum likelihood tree (generated by RAxML), supported by Bayesian analysis. Branches indicated in red are not supported (ML bootstrap ≤80%; Bayesian posterior probability < 1). Bolded taxa are sequences obtained from high-throughput transcriptomics. Those with an identifier following the name resulted from transcriptomes pulled from NCBI. Branch with lower support (†) indicates a possible single common ancestor of *G*. *belizeanus*, *G*. *pacificus*, and *G*. sp. Ribotype 2. Nucleotide tree with 99 taxa. There are 0.1 substitutions per site.

The D1 to D3 region of the Large Ribosomal subunit was isolated from our transcriptome data and examined to determine its usefulness in identifying species respective to the multi-gene trees generated as described below in the “Analysis of Core Eukaryotic Genes” and “Analysis of Genes from Janouškovec et al., (2017)” sections. This was done by using a reciprocal best-BLASTn with our SPAdes generated contigs and D1 to D3 regions from numerous *Gambierdiscus* species published as individual nucleotide sequences in NCBI (see Fig 1). We then used the Map to Reference tool in Geneious to map the closest-relative NCBI reference sequence to the best reciprocal BLASTn hit, determining the region of the contig that contained the D1 to D3 sequence. The sequences isolated from our transcriptomes and those obtained from NCBI were aligned using MUSCLE and analyzed using RAxML with 500 bootstraps and the GTRGAMMA model. This represented a sufficient number of replicates to produce a consistent tree. The resultant tree was visualized using FigTree [63].

#### ITS/5.8S rDNA region phylogeny

To test the discriminatory power of ITS/5.8 regions to delineate *Gambierdiscus*, as well as closely related *Fukuyoa* and *Alexandrium* species, a consensus sequence for each species was assembled and pulled from their respective transcriptomes. In the case of *G*. *excentricus*, there were no reference sequences available and so a sequence from the known 3’ end of the SSU region was used to find contigs extending into the 5’ portion of ITS1. The identified 5’ portion of ITS1 was then used to discover the full ITS/5.8S region, the sequences of which were then aligned in Geneious using MUSCLE with default parameters. A phylogeny based on these aligned sequences was then produced as described in section “D1 to D3 Large Ribosomal Subunit Region Phylogeny”.

### Analysis of Core Eukaryotic Genes

#### Isolating orthologs

Core Eukaryotic Genes obtained from the BUSCO (https://busco.ezlab.org) and CEGMA (https://korflab.ucdavis.edu/datasets/cegma) databases were used as a reference to find genes that would be present in every species of *Gambierdiscus* and all outgroup taxa (*Alexandrium* spp. *T*. *fusus* and *P*. *bahamense*). As the Core genes were provided as amino acid rather than nucleotide data, we used Transdecoder (v. 5.1.0; https://github.com/TransDecoder) to translate our RNA-seq data into amino acid data for comparison. A reciprocal best-BLASTp was conducted using BLAST+ software [64, 65] with our SPAdes assembled contigs as query and the Core Eukaryotic Genes (a total of 577 genes that were greater than 100 amino acids in length) as reference. Genes that had only one to four top hits (based on bitscore) were selected to continue, leaving a total of 39 genes. All top hits from all taxa for these 39 genes were examined by first aligning the sequences for each individual gene with MUSCLE (v. 3.8.31; [66, 67]) followed by phylogenetic analysis using RAxML (v. 8.0.0 [68]; https://github.com/stamatak/standard-RAxML), with 100 bootstraps and the PROTGAMMMAAUTO model and visualized using FigTree. (http://tree.bio.ed.ac.uk/software/figtree/).

For genes that had multiple best hits from BLASTp, we determined the orthologs through pairwise and group comparisons. Using MEGA7 [69], we calculated Dayhoff/JTT distances for each pairwise alignment and any sequence that had a distance value greater than 2x the average pairwise distance was eliminated. In cases of clearly paralogous gene duplicates (i.e. separated clades containing duplicate copies for each taxon), we calculated Dayhoff/JTT distances within and between each group. The group that was the most complete (i.e. contained at least one sequence per taxon) and, when both were complete, had the lowest within-group distance value, was selected for analysis. Genes where no one group that contained copies of all (or nearly all) taxa were eliminated; leaving a total of 32 genes. These genes were analyzed by RAxML a second time. Genes were eliminated if the tree diameter was greater than 1 and/or the outgroup (*Alexandrium spp*., *Tripos fusus* and *Pyrodinium bahamense*) and in-group (*Gambierdiscus* spp.) were not reciprocally monophyletic as they are known to be [41]. This left a total of 28 genes for 19 taxa (see S1-S45 Figs in S1 File, and S2 Table in S1 File), with a total of 45,828 nucleotides total in length.

#### Final phylogenetic analysis

The nucleotide sequences for each of these 28 genes were aligned using MUSCLE, concatenated, and analyzed using RAxML with 500 bootstraps and the GTRGAMMA model, which was selected using jModelTest2 (v. 2.1.10; [70, 71]). In addition, a Bayesian MCMCMC phylogenetic analysis was performed with MrBayes (v. 3.2.6; [72]). The best-fit model (GTR) used for the Bayesian analyses was selected using the Akaike Information Criterion in MrModeltest (v. 2.4; [73]). Bayesian analyses were performed with six Markov Chains [74] for 3,000,000 generations. Posterior probabilities (PP) were calculated, with the stabilization of the model parameters (i.e. burn-in) occurring around 2,800,000 generations (where the first 2,800,000 generations were discarded, while the remaining 200,000 were utilized to determine the tree). Every 100^th^ tree following stabilization was sampled to determine a 50% majority rule consensus tree.

#### Parametric bootstrapping

In addition to the standard, non-parametric bootstrapping of our 28 gene, concatenated tree, we also performed parametric bootstrapping to evaluate consistency of phylogenetic signal among the 28 genes. Pseudo-replicates were generated with Seq-Gen (https://github.com/rambaut/Seq-Gen) using the GTR model. Simulated datasets were generated for each of the different gene lengths and were run through RAxML to obtain likelihood values. This was replicated 100 times. These values were then plotted into histograms using R, along with two additional likelihood values: 1) the likelihood of the original gene alignment (with no reference to the final concatenated tree) and 2) the likelihood of the original gene alignment given the final, 28-gene concatenated tree (see S46 Fig in S1 File).

### Analysis of genes from Janouškovec et al. (2017)

#### Isolating orthologs

In addition to our examination of the Core Eukaryotic Genes from BUSCO and CEGMA datasets, we also examined a dataset of 101 genes that were used in Janouškovec et al., 2017 [41] to determine the phylogenetic relationships among multiple dinoflagellate genera. This data set had already been curated for dinoflagellates with the express purpose of eliminating any multigene families. First, a reciprocal best-BLASTn was run with our SPAdes assembled contigs and the 101 genes in this dataset. We then isolated the genes that had only one to four significant reciprocal-best hits, leaving a total of 17 genes (see S1-S45 Figs in S1 File, and S2 Table in S1 File), with 22,743 nucleotides total in length. We aligned all of the sequences from each taxon for each gene using MUSCLE and analyzed them with RAxML using 500 bootstraps and the GTRGAMMA model. Using Geneious 11.1.5 (https://geneious.com), we examined the pairwise distances and eliminated that any sequence with an average percent identity of 20% or lower than the next lowest average. When more than one sequence per taxon remained but were monophyletic and did not have a conspicuously long branch, the longest sequence (bp) was chosen to proceed. This new dataset is termed here “JJ-PNAS” to indicate its close relationship to the Janouškovec et al. (2017) dataset.

#### Final phylogenetic analysis

The final nucleotide sequences for each of these 17 genes were aligned using MUSCLE, concatenated and analyzed using RAxML with 500 bootstraps and the GTRGAMMA model. Additionally, a tree was produced for the same alignment using the Bayesian Markov Chain Monte Carlo method as implemented with MrBayes using the same parameters described in the “ITS/5.8S rDNA Region Phylogeny” section, with the exception of the best-fit model, which was determined to be SYM+G using MrModelTest2.

### Data location

Sequence reads were deposited in the National Center for Biotechnological Information, Short Read Archive (NCBI, SRA) as BioSamples SAMN14442098 to SAMN14442113 under BioProject PRJNA614967 (see S3 Table in S1 File for specifics). The LSU D1-D3 rDNA sequences isolated from each of the transcriptomes assembled in this study are available at Genbank under Accessions: MT248299- MT248319. The ITS1 rDNA sequences also isolated from the transcriptomes are available at Genbank under Accessions: MZ964947- MZ964963. Gene alignments and additional data can be found in at figshare at https://figshare.com/projects/Gambierdiscus_Transcriptome-Based_Phylogenies/77709.

### Digital code and procedure archive

The code used in this project can be obtained from https:github.com/brittanymareeott/Gambierdiscus. For more detailed procedures, please visit https:github.com/brittanymareeott/Gambierdiscus/wiki/.

### Literature survey of SSU, ITS/5.8S or D1-D6 LSU rDNA phylogenies to determine species delimitation

A comprehensive review of rDNA phylogenies from 473 articles was completed to address how well phylogenies based on a single locus could discriminate dinoflagellate species. Specifically, the goal was to determine how well rDNA phylogenies based on either SSU, ITS/5.8S, or D1-D2 / D1-D3 / D1-D6 LSU rDNA clades distinguished dinoflagellates species/ribotypes (i.e. putative new species), given there is concern regarding whether single-locus phylogenies can provide a strict threshold of reciprocal monophyly for delineating species [40]. Articles included in this survey were identified using Web of Science, Google searches, and from the reference sections of various taxonomic studies. The data were summarized in tabular form and included the following information: genus, species / ribotype; whether a SSU, ITS/5.8S or D1-D2/D1-D3/D1-D6 LSU rDNA phylogeny exists for each species/ribotype entry; how well the phylogeny did in distinguishing species/ribotype (see below); and a representative listing of the articles containing phylogenies from which the data were extracted.

The nomenclatural changes that occurred over time were also reflected in the table with the most current name followed by previous homotypic genus and species names shown in parentheses. These nomenclatural changes were determined using AlgaeBase with the understanding that AlgaeBase is not a definitive taxonomic authority. However, AlgaeBase is relatively up to date and provides the best means we are familiar with of assessing nomenclatural changes over the past 25 years for such a diverse group of dinoflagellates.

Classifying how well species/ribotype(s) were delineated in a given phylogeny was nuanced and dependent on a combination of factors. The first factor was whether single or multiple sequences were used to represent a given species/ribotype in a particular phylogeny. In cases where the single sequence fell on a well-separated branch, it was taken as tentatively representing a distinct species. Multiple sequences attributed to the same species, and falling into a distinct clade, was interpreted as definitive evidence that the clade represented a distinct species. The second factor was whether all the sequences falling into a given clade were properly identified. Culture isolates can be difficult to distinguish. As a result, some distinct clades contain sequences from the same species that have been simply misidenidentified. When this situation arises, it can be difficult to discern whether the sequences ascribed to multiple species which fall into the same clade represent a case is mistaken identity, or if the sequences are from two recently diverged, valid species that still share the same rDNA sequences. Taking these potential complications into consideration, the species and ribotypes identified in the various individual phylogenies were evaluated and assigned to one of the following five designations:

- Yes unconditionally (= Y), represents cases where two or more sequences from the same species formed a distinct phylogentic cluter in one or more of the phylogenies reviewed.
- Yes provisionally (= YP), indicates when a single sequence representing a given species fell on a branch distinct from other species.
- Yes, but ambiguous (= YA), designates situations where a distinct clade contains sequences ascribed to multiple species, but where there is morphological or other evidence from the original description indicating the sequences were from the same species, only misidentified when submitted to GenBank.
- No (= N), denotes cases where sequences from different known morphologically distinct species fall into the same clade–i.e. have indistinguishable rDNA sequences. In these instances, phylogenies based on the rDNA gene segment fail to delineate what are morphologically distinct species.
- No, but ambiguous (= NA), represents a rarer version of the YA designation where there is some evidence the different sequences in the same clade are actually from distinct species, but this could not be fully resolved given the available data.

Since individual rDNA phylogenetic trees frequently utilized different species-specific sequences, the results were not always consistent. As a result, species / ribotypes were sometimes assigned more than one of the various designations (Y, YP, YA, N or NA). These contradictory results are listed in S3 Table in S1 File.

As a means of summarizing the data from the literature, a three-category classification system for evaluating whether or not a phylogeny resolved a particular species was developed. This analysis was limited to only described species, and not ribotypes. The three categories were–(A) “Not resolved”, i.e. the rDNA phylogeny failed to resolve species, (B) “Ambiguous”, the results were inconclusive, and (C) “Resolved”, the rDNA phylogeny yielded a clade consistent with a distinct species. The not resolved category included any species/rDNA region having received any of the following classifications(s)—N, NA, N/NA, N/YA, or Y/N. The ambiguous category was comprised of those species/rDNA regions receiving either a YA, Y/YA or YP/YA classification. The resolved category included the Y or YP classifications. Once the data were classified, the number of species for a given rDNA region falling into each category were counted. Next, the percentage of species falling into each of three categories for each rDNA region were calculated. Though not included in the analysis of the described species, the total number of undescribed ribotypes potentially representing new species was tallied as well to provide an estimate of how many undescribed species have been identified to date (see S3 Table in S1 File.

## Results

### D1-D3 region phylogeny

The D1-D3 phylogeny yielded terminal clusters consistent with the previous phylogentic analyses and all the described species within the genus *Gambierdiscus* [51–60, 75] (Fig 1). In each instance, the transcriptomic D1-D3 consensus sequences for species grouped with corresponding species-specific sequences obtained from GenBank. In some cases, the transcriptomic data for the D1-D3 alleles obtained from a given species exhibited greater intraspecific divergences among the various alleles than revealed by initial sequencing efforts. This observation is consistent with previous studies demonstrating significantly varying pseudogene frequencies in the genomes of even closely related species [75, 76]. For example, *G*. *carpenteri* contained variants with small insertions and deletions that lead to at least three distinct consensus sequences. The same was true for *G*. *beliezeanus* and *G*. *pacificus*, whose consensus sequences contained small inserts/deletions relative to existing sequences which were otherwise equivalent (Fig 1). All of the divergent transcriptomic sequences fell into the terminal clusters of their respective species.

The D1-D3 phylogenies were also consistent with known species in the genera *Alexandrium*, *Tripos*, and *Fukuyoa*. The taxonomy of *Pyrodinium* species is currently in flux, but the data indicate *P*. *bahamense* and *P*. *var compressum* are possibly separate species. Further, the *P*. *bahamense* D1-D3 transcriptomic (MMETSP0796) consensus sequence is so divergent it probably represents another *Pyrodinium* species altogether. An even greater divergence was observed among the *Tripos* sequences, identified as *Tripos fuscus*, indicating these isolates also likely represent multiple species. These observations further illustrate the importance of careful vouchering as sequence data can be collected from strains that are poorly characterized or have been misidentified after they were collected. Consequently, species names of accessible libraries should not be taken at face value, particularly in genera whose taxonomy was unresolved at the time the library was constructed. Ribosomal and other genes could be used to resolve any uncertainties.

### ITS/5.8S phylogenies

**Fig 2:**
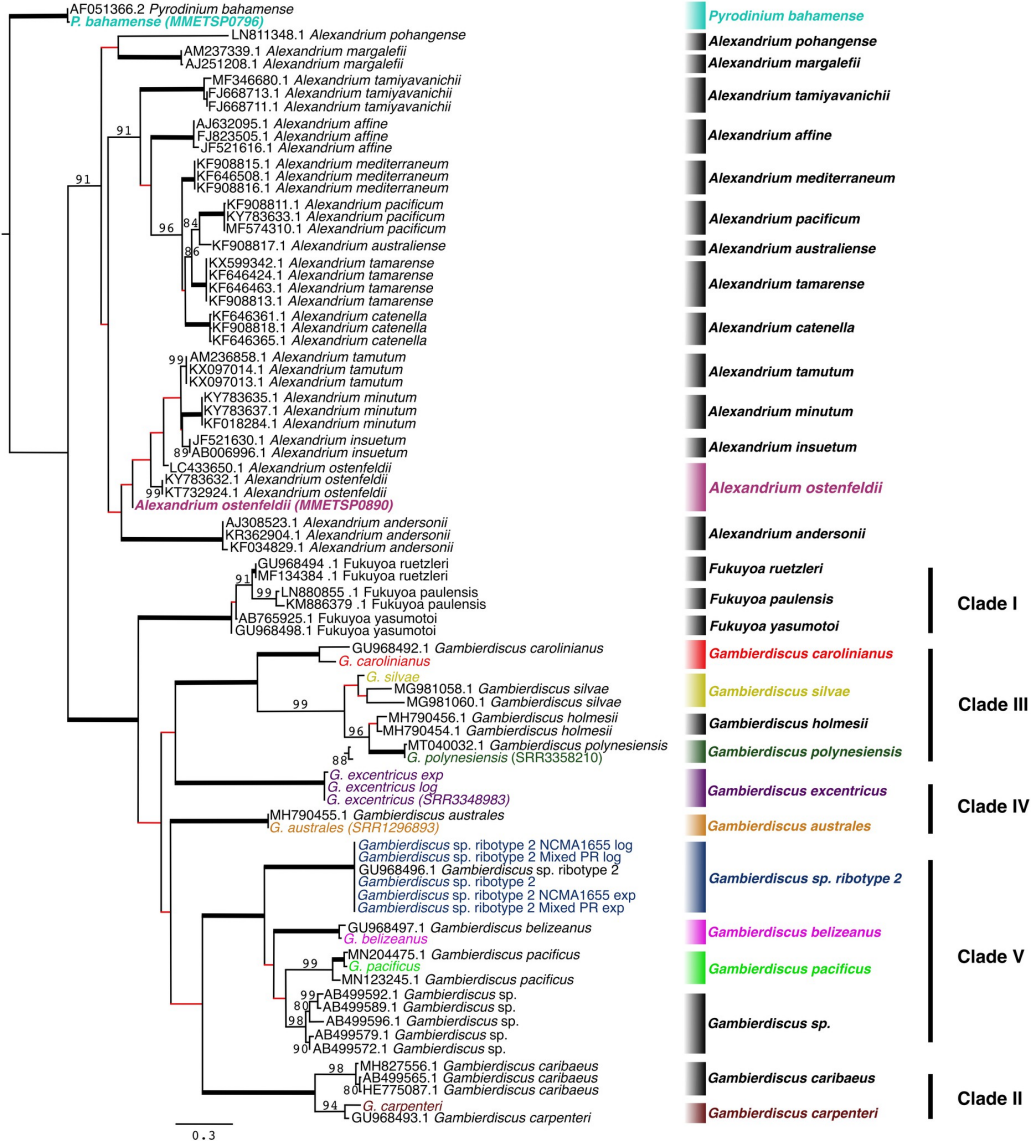
*Gambierdiscus*, *Fukuyoa and Alexandrium* ITS/5.8S phylogeny. Maximum likelihood tree (generated by RAxML). Branches indicated in red are not supported (ML bootstrap ≤80%). Bolded taxa are sequences obtained from high-throughput transcriptomics. Those with an identifier following the name resulted from transcriptomes pulled from NCBI. Nucleotide tree with 81 taxa. There are 0.3 substitutions per site.

The ITS/5.8S region was also examined to determine how well this region distinguished *Gambierdiscus* species [32]. The phylogeny (Fig 2) showed the ITS/5.8S sequences also clearly delineate *Gambierdiscus*, *Fukuyoa and Alexandriumn* species and confirm the species boundaries indicated in the D1-D3 phylogeny (Fig 1).

### Multi-gene phylogenies

**Fig 3:**
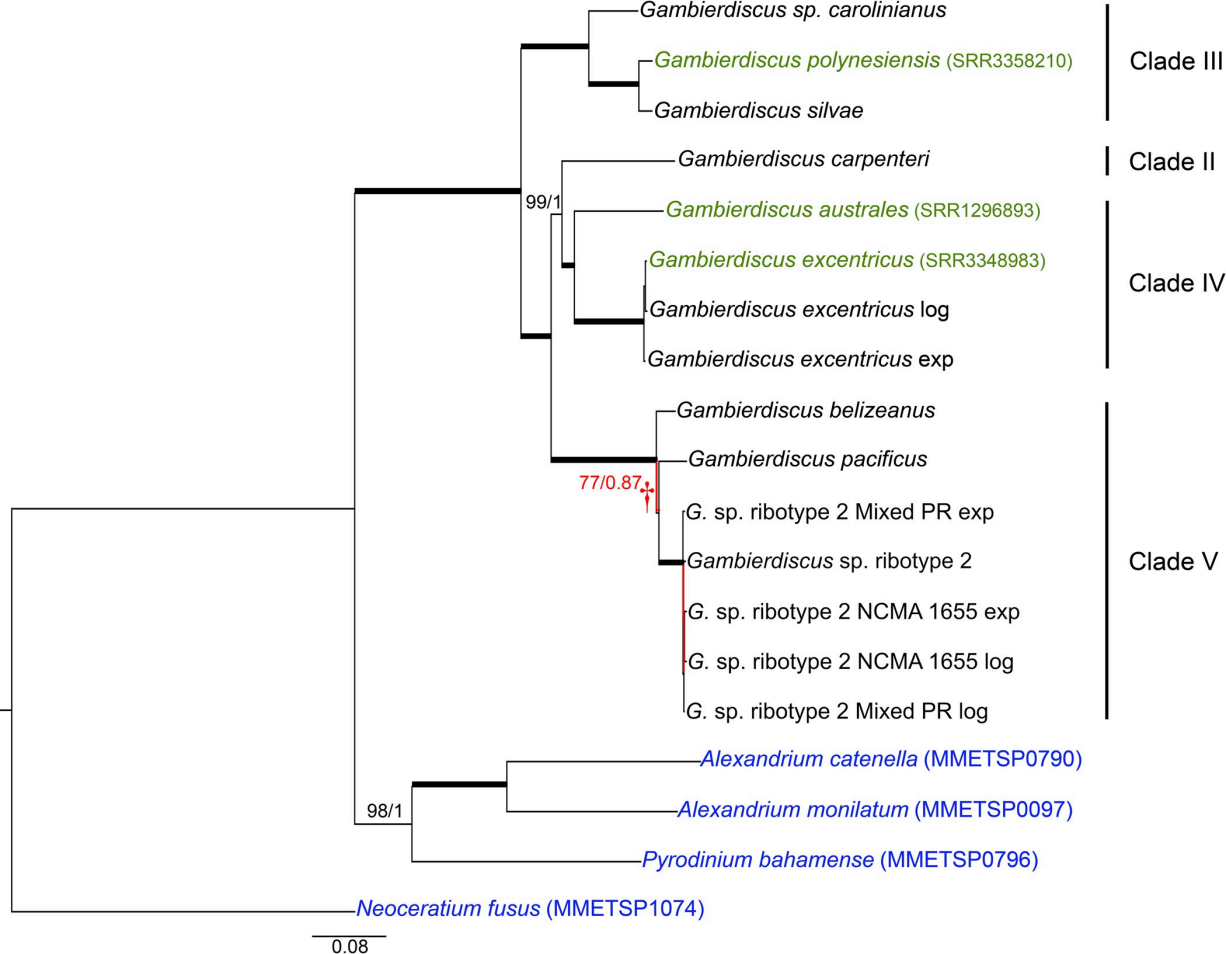
Phylogenetic tree of *Gambierdiscus* using transcriptomes and Core Eukaryotic reference genes. Maximum likelihood tree (found by RAxML), supported by Bayesian analysis. Branches indicated in red are not supported (ML bootstrap ≤80%; Bayesian posterior probability < 1). Taxa in green indicate *Gambierdiscus* transcriptomes obtained from NCBI. Taxa in blue indicate outgroups, whose transcriptomes were also obtained from NCBI. SRA identifiers are found in parentheses next to the taxon name. Branch with low support (†) indicates a possible single common ancestor of *G*. *belizeanus*, *G*. *pacificus*, and *G*. sp. ribotype 2. Genes were chosen using the Core Eukaryotic Gene dataset (BUSCO and CEGMA) as reference. Nucleotide tree: 28 genes; 45,828 bp. There are 0.08 substitutions per site.

**Fig 4:**
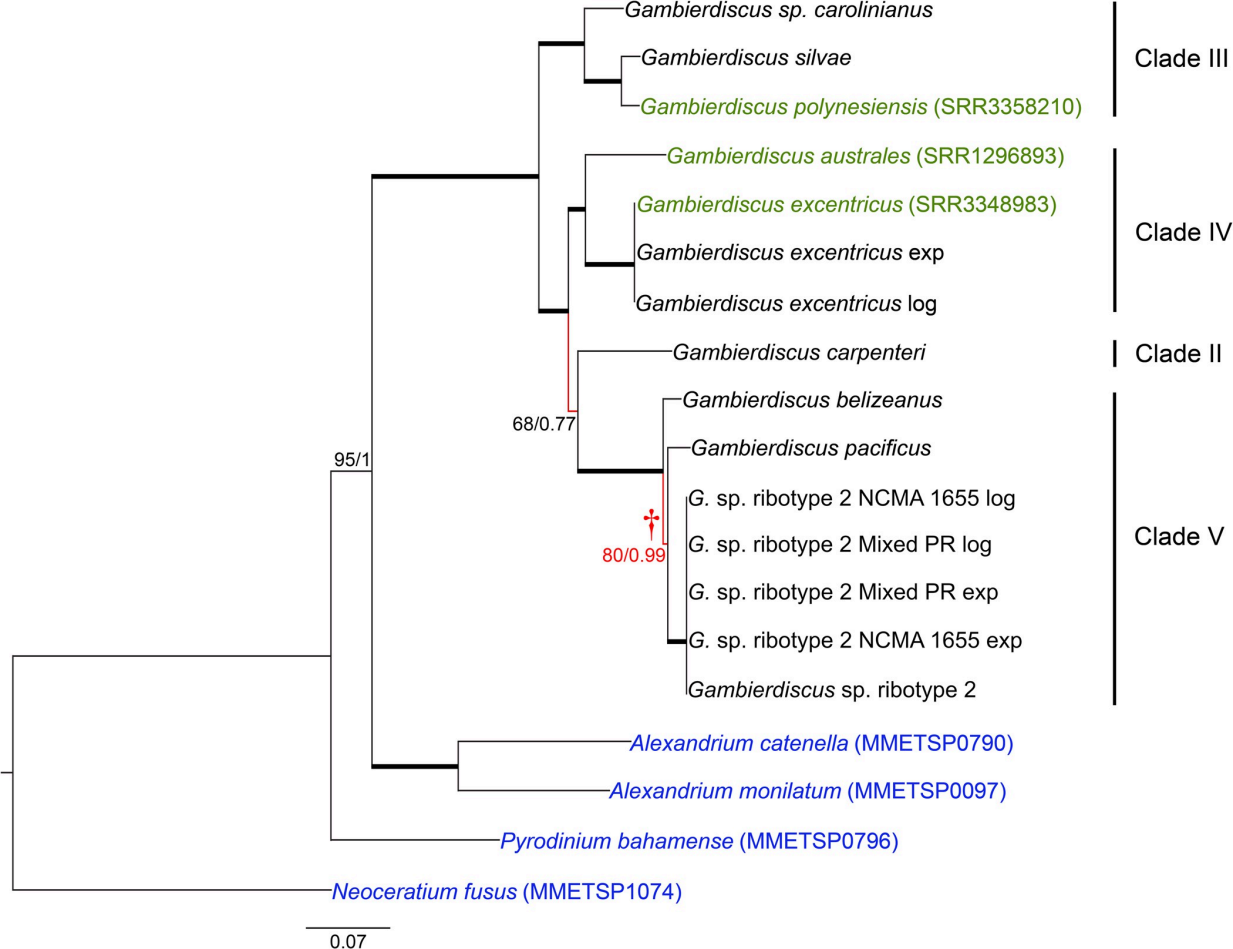
Phylogenetic tree of *Gambierdiscus* using transcriptomes and Janouškovec *et al*., 2017 reference genes. Maximum likelihood tree (found by RAxML), supported by Bayesian analysis. Branches indicated in red are not supported (ML bootstrap ≤80%; Bayesian posterior probability < 1). Taxa in green indicate *Gambierdiscus* transcriptomes obtained from NCBI. Taxa in blue indicate outgroups, whose transcriptomes were also obtained from NCBI. SRA identifiers are found in parentheses next to the taxon name. Branch with low support (†) indicates a possible single common ancestor of *G*. *belizeanus*, *G*. *pacificus*, and *G*. sp. ribotype 2. Genes were chosen using the genes described in Janouškovec *et al*., 2017 as reference. Nucleotide tree: 17 genes; 22,743 bp. There are 0.07 substitutions per site.

The *Gambierdiscus* phylogenies based on Core Eukaryotic Genes obtained from the BUSCO and CEGMA databases (28-gene phylogeny; Fig 3), and those using the genes selected by Janouškovec *et al*. (2017) (17-gene phylogeny; Fig 4) again yielded terminal clusters, with high support, equivalent to the same species observed in the D1-D3 phylogeny. Where multiple libraries for the same species were available, the intraspecific variation was much lower than what was observed between species (Figs 3 and 4). Furthermore, both the Core Eukaryotic Gene and JJ-PNAS dataset phylogenies indicate a potential rapid radiation event involving *G*. *belizeanus*, *G*. *pacificus* and *Gambierdiscus* ribotype 2 (clade V) (Figs 3 and 4).

The primary difference between the *Gambierdiscus* portion of the D1-D3 phylogeny and both the Core Eukaryotic and JJ-PNAS dataset phylogenies was the branching order among clades. In the D1-D3 phylogeny, Clades II and IV were on a co-equal branch separate from Clades III and V, either of which might be ancestral (Fig 1). In both the 28-gene and 17-gene phylogenies, Clade III was ancestral to clades II, IV and V with placement of Clade II being ambiguous. In 28-gene phylogeny, Clade II, represented by *G*. *carpenteri*, was most closely associated with Clade IV, consistent with the D1-D3 phylogeny (Fig 3). The 17-gene phylogeny showed Clade II as intermediate between IV and V but with weak branch support (Fig 4). The inclusion of transcriptomic data from *G*. *caribaeus*, the other Clade II species, may be required to more fully resolve the position of Clade II, but the stronger data indicate the association of Clades II and IV as in the D1-D3 phylogeny (Figs 3 & 4).

The multigene and D1-D3 phylogenies both showed a relatively recent divergence among Clade V species (*G*. *belizeanus*, *Gambierdicus ribotype 2* and *G*. *pacificus*), possibly indicating a recent radiation event (Figs 3 and 4). The potential recent divergence is also reflected in the 5.8S/ITS phylogeny (Fig 2).

### Literature survey results

The SSU, ITS/5.8S and 5’ LSU (mainly D1-D2 and D1-D3, but some D1-D6 regions) phylogenies from the survey articles were examined in detail. Where different articles included matching D1-D3 and D1-D6 phylogenies for a given species, they provided equivalent results. Similarly, with a few exceptions, the D1-D2-based phylogeny results were also congruent with those of D1-D3, indicating the D1-D2 region contained nearly as much information as the D1-D3 region. Consequently, the D1-D2, D1-D3, and D1-D6-based phylogeny results were treated as equivalent evidence of whether this region does or does resolve species This was done with the knowledge that a small number of D1-D2 results may not have been as fully resolved as those based on D1-D3 or D1-D6 regions.

In total, rDNA phylogenies for 863 described species based on one or more of the three rDNA regions examined were identified along with over 315 ribotypes that likely represent other distinct species. These described species and the various ribotypes spanned 233 genera. Overall, the SSU and D1-D3 sequences have similar success at delineating >93% of the dinoflagellate species surveyed, with the ITS/5.8S region capable of resolving >97% of species (Table 3).

**Table 3:** Summary of the results regarding how well the various rDNA regions resolved species boundaries.

	SSU			ITS/5.8S			5’ LSU		
	Not Resolved	Ambiguous	Resolved	Not Resolved	Ambiguous	Resolved	Not Resolved	Ambiguous	Resolved
**Number of Species**	34	8	591	6	5	379	27	15	618
**% of Total Species**	5.3	1.3	93.4	1.5	1.3	97.2	4.1	2.3	93.6

The full results of the literature survey are presented in S3 Table in S1 File. The genera containing one or more species for which rDNA sequences did not fully resolve species boundaries included *Amphidinium*, *Apocalathium*, *Archaeperidinium*, *Biecheleria*, *Centrodinium*, *Ceratocorys*, *Dinophysis*, *Gonyaulax*, *Gymnodinium*, *Prorocentrum*, *Scrippsiella*, and *Syltodinium*.

Of the 439 instances where there were both ITS/5.8S and D1-D3 data available for described species and ribotypes, these two regions either resolved or failed to resolve species boundaries equally well–i.e. gave they produced equivalent results >99% of the time (433 out of 439 times). Of the six instances where the two regions disagreed, five occurred in the genus *Dinophysis*. In each case the ITS region was able to distinguish species whereas the 5’ LSU (D1-D6) sequences failed to do so. These results indicate that when both the ITS/5.8S and D1-D3 show the same distinct species-specific clades, it is strong evidence that the sequences in question represent a single distinct species.

## Discussion

### Phylogenies using ITS/5.8 and D1-D3 rDNA genes identify *Gambierdiscus* species just as well as multigene phylogenies

The multigene phylogenies for *Gambierdisucs* based on 28 Core Eukaryotic genes and the subset of 17 genes identified by Janouškovec *et al*. (2017) yielded the same species clades as those phylogenies based solely on D1-D3 or ITS/5.8S LSU rDNA sequences (Figs 1–4). The consistency of these data demonstrates the D1-D3 LSU and the ITS/5.8S rDNA are capable of distinguishing *Gambierdiscus* species. This is important for efforts to better define the diversity of *Gambierdiscus* species, some of which produce ciguatoxins that bioaccumulate in the food chain resulting in ciguatera fish or shellfish poisoning (CP). Globally, CP is the leading cause of non-bacterial seafood toxicity. Given the toxicity of *Gambierdiscus* species varies dramatically, considerable effort has been made over the past decade to identify and describe new species and assess their toxicity. This research has been complicated because the morphologies exhibited by many *Gambierdiscus* species overlap. As a result, greater emphasis has been placed on using molecular characters, primarily rDNA gene regions, to define species [34, 77]. These descriptions were undertaken knowing the underlying concern that multigene phylogenies might prove better at delineating species than the rDNA sequences alone. The results of this study confirm for the first time D1-D3 and ITS/5.8S rDNA-based phylogenies, by themselves, can efficiently distinguish *Gambierdiscus* species without the requirement for using multigene phylogenies. However, it is important to note that, while these sequences can *distinguish* species within *Gambierdiscus*, defining a novel species likely requires more support from multigene phylogenetic data.

### Assessing the effectiveness of SSU, ITS/5.8S and the 5’ LSU (D1-D2, D1-D3, D1-D6) rDNA regions to distinguish other dinoflagellate species

The phylogenetic analyses carried out in this study showed the divergence in both the ITS/5.8S and D1-D3 rDNA were sufficient to identified closely related *Alexandrium* and *Fukuyoa* species and indicated the presence of potentially undescribed species in the genera *Pyrodinium* and *Tripos* as well (Figs 1 and 2). These observations raised the question as to what degree rDNA sequences can be used to distinguish dinoflagellate species in general. To address this question, a survey of rDNA phylogenies from 473 articles was undertaken (S3 Table in S1 File). The complied results revealed that of the 863 described dinoflagellate species identified, the SSU and 5’ LSU regions could distinguish >93% of the described species for which sequences were available (Table 3). The ITS/5.8S region in contrast could discriminate >97% of the species. This is an approximately 4% increase over using D1-D3 alone.

Though the same ability to discriminate species can be achieved using either a combination of ITS/5.8S plus D1-D3 LSU versus ITS/5.8S plus SSU rDNA phylogenies, we argue the former is the better choice. The reason is the greater sequence variation per base pair (higher information content) observed in the ITS/5.8S and 5’ LSU regions versus the SSU [78]. This difference in information content is reflected in efforts to design species-specific molecular assays over the past several decades. That work has shown the higher sequence variability in the ITS/5.8S and D1-D3 regions provide more closely spaced, species-specific sequences for use in constructing quantitative assays, than does the SSU region [79]. An additional benefit is that the D1-D3 is shorter, requiring less sequencing effort.

The greater ability of the ITS/5.8S region to delineate a higher percentage of species (97% versus 93%-94% as seen in the literature survey) is likely attributable to its function relative to the SSU and LSU rDNAs (Table 3). The entire rDNA complex is initially transcribed as a single long rRNA transcript. The SSU, LSU, and 5.8S are subsequently excised from this long transcript and serve as key structural components in the molecular complex which synthesizes proteins. Given their critical role in protein synthesis, they are under strong stabilizing selection. The ITS regions, though they play a key role in the excision of the SSU, LSU and 5.8S rDNA from the original transcript, are not under as strong as a stabilizing selection. Hence, the ITS1 and 2 regions diverge even more rapidly after speciation events than the corresponding structural genes. But despite the more rapid evolution rate in the ITS regions, any new divergences still remain species-specific, due to concerted evolution, the process where loci of homologous gene sequences are homogenized within a species [19, 75, 76, 80] (Fig 2). Though this process ensures even the most divergent rDNA alleles remain species-specific, it is not perfect. Consequently, some dinoflagellate species exhibit much higher inter-allelic sequence variation in the rDNA genes, with numerous pseudogene copies often observed, though they still segregate into species-specific clades [26, 76, 81, 82] (Figs 1 and 2).

This higher rate of divergence, coupled with homogenization of ITS/5.8S alleles, makes this gene region ideal for distinguishing recently separated species in which there has been either relatively little or no morphological differentiation [75, 76, 83–86] (S3 Table in S1 File). Some good examples are the coral and radiolarian endosymbiotic species within the *Symbiodinium* complex which have overlapping morphologies and where this region makes a particularly effective marker for species level divergences [19, 87].

Using ITS phylogenetic analyses is also less tedious than using genetic desistance as a means of distinguishing species [19, 75, 88]. Though genetic distances in this region work well for resolving most dinoflagellate species, its optimal application requires eliminates of pseudo-genes before calculating genetic distances [75]. Removing these non-functional alleles is not needed for phylogenetic analyses, because even the divergent genes generally fall into the same distinct species-specific clades [76, 86].

A final advantage of obtaining the ITS/5.8S data is it use in complementary base pair change (CBCs) analysis which examines if consistent base-pair substitutions have occurred in the ITS1 or ITS2 region [89–91]. Such substitutions are consistent with species level divergences [92, 93]. It should be noted that whereas the presence of a CBC correlates well with species boundaries, speciation can occur without the development of a CBC. Cumulatively, these data make a compelling argument for the ITS/5.8S and D1-D3 having the ability to distinguish most dinoflagellates species.

### Multigene versus rDNA phylogenies for assessing phylogenetic relationships

Although the *Gambierdiscus* multigene transcriptomic-based phylogenies grouped species into clades consistent with those observed in the D1-D3 phylogeny, there were differences in branching order. Both Core Eukaryotic and JJ-PNAS dataset multigene phylogenies indicated that Clade III was ancestral to Clades II, IV and V, whereas in the D1-D3 phylogeny Clades II and IV were on co-equal branches sister to Clades III and V (Figs 1, 3 and 4). Clades IV and V were on co-equal branches as in the D1-D3 phylogeny with the strongest support for placement of Clade II with Clade IV as in the D1-D3 phylogeny (Fig 1). Any ambiguity as to the placement of Clade II will likely be resolved when transcriptomic sequences become available for the other known Clade II species, *G*. *caribaeus*. Additionally, these transcriptomic-based phylogenies indicate a potential rapid radiation event that included *G*. *belizeanus*, *G*. *pacificus*, and *Gambierdiscus* sp. ribotype 2 (Clade V; Figs 3 and 4). Though there was a relative agreement in the evolutionary relationship revealed between the rDNA and multigene phylogenies, the higher number of genes (n = 27 genes; 45,828 nucleotides) in the core eukaryotic gene phylogeny and in the JJ-PNAS dataset (n = 17; 22,743 nucleotides) will undoubtedly produce more reliable phylogenies. This conclusion is supported by other studies also employing multiple genes [41, 94, 95]. As transcriptomic and genomic analyses become more standardized, multigene phylogenies will likely become the standard basis for assessing phylogenetic relationships.

### Proposal for use of rDNA sequence in describing dinoflagellate species

The literature survey results clearly indicate ITS/5.8S and D1-D3 LSU rDNA phylogenies can be concatenated and used in combination to distinguish most, but not all dinoflagellate species (Table 3). This raises the natural question of how to know when it is and is not appropriate to use the ITS/5.8S and D1-D3 phylogenies as definitive molecular characters when defining dinoflagellate species. To address this issue, we present a five-case decision tree, which provides defined rules govering the relative weight to assign morphological versus molecular characters when describing a species.

#### Detailed morphological description is the required starting point for any species description

An essential starting point for any description process is the continued practice of characterizing new species morphologically. This can be done with either single cell isolates or from single cells collected in the field and identified molecularly. Ideally, species descriptions should include both high-resolution light and scanning electron microscopy micrographs illustrating the full range of morphological variation exhibited by a species. The principle goal of taxonomy is to provide a framework upon which all other investigations depend. Defining species with molecular data without first providing morphological characterization has become increasingly common with the advent of metagenomic approaches, but unfortunately, no matter how strong the molecular evidence, identification of a species without morophological characterization strongly constrains this broader scientific work, particularly in regard to ecological field studies. That said, morphology alone frequently fails to delineate species boundaries without corresponding molecular corroboration, so ideally both data types would be considered.

It is also important to note that, unlike the situation with bacteria and archaea, there are many morphologically described dinoflagellate species for which there are not yet any corresponding molecular data. Consequently, many “novel” sequences in metagenomic datasets likely correspond to described, but unsequenced taxa. Thus, a major challenge moving forward will be to correlate and reconcile morphological and molecular data.

**Fig 5:**
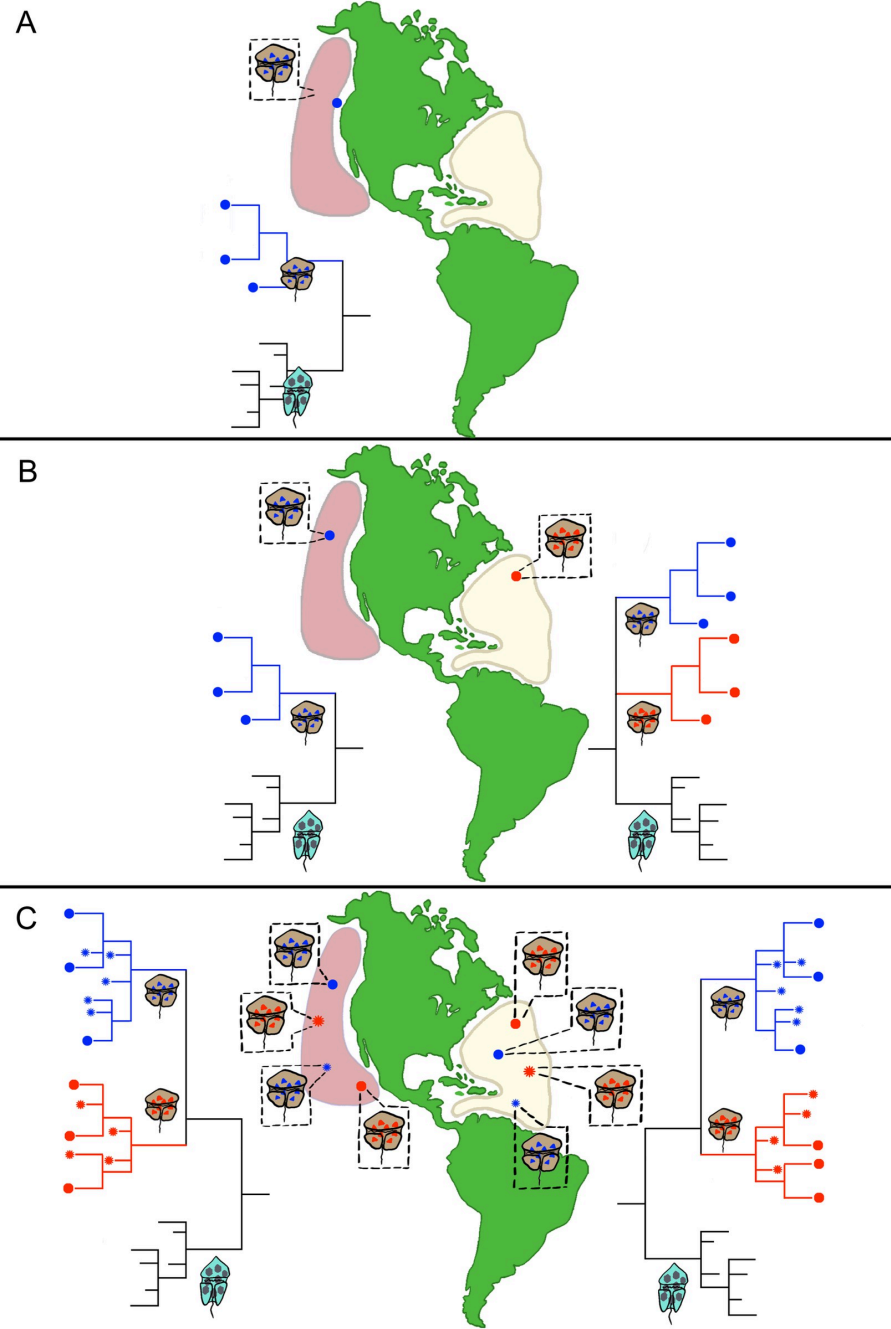
Diagram illustrating how various morphological and molecular phylogenetic information can be used in describing dinoflagellate species. Panel A) Example of a case where morphologically distinct isolates from the Pacific region (represented by the brown cell) compared to the morphology of a related, previously described, co-occurring species (represented by the bluish-green cell). The morphological differences were found to be distinct and non-overlapping with previously described species, supporting describing the new isolates as a separate species. Subsequent sequencing and phylogenetic analysis of the D1-D3 and ITS/5.8S regions from isolates of both species fell into distinct, non-overlapping clades. Here, description of the new species based on morphology and supported by the molecular data is warranted. Panel B) represents a situation where the isolates morphologically similar to those of newly described Pacific species (brown cell) were obtained from the Atlantic region. Morphometric analysis showed all the morphological features examined overlap to a significant degree. This state is indicated by the Atlantic isolates having the same shape cell as that shown for newly described Pacific species and a different coloration (pinkish purple). Here morphology alone does not unambiguously support the establishment of the new species. In contrast, the phylogenetic analysis of the D1-D3 and ITS/5.8S regions from the isolates consistently fall into distinct clades, clearly supporting establishment of the Atlantic isolates as a new species. Panel C) shows a situation where additional isolates from both the Atlantic and Pacific were sequenced. With our increasing capacity to carry out affordable sequencing, this will become an ever more common occurrence. In this example, subsequent phylogenetic analysis showed all three species occurred sympatrically in both regions and that in each regions the phylogenies yielded the same distinct species-specific clusters. Though not necessary for describing new species, the continued return of distinct species-specific clusters from regions where the species occur sympatrically provides additional evidence the described species are reproductively isolated.

**Fig 6:**
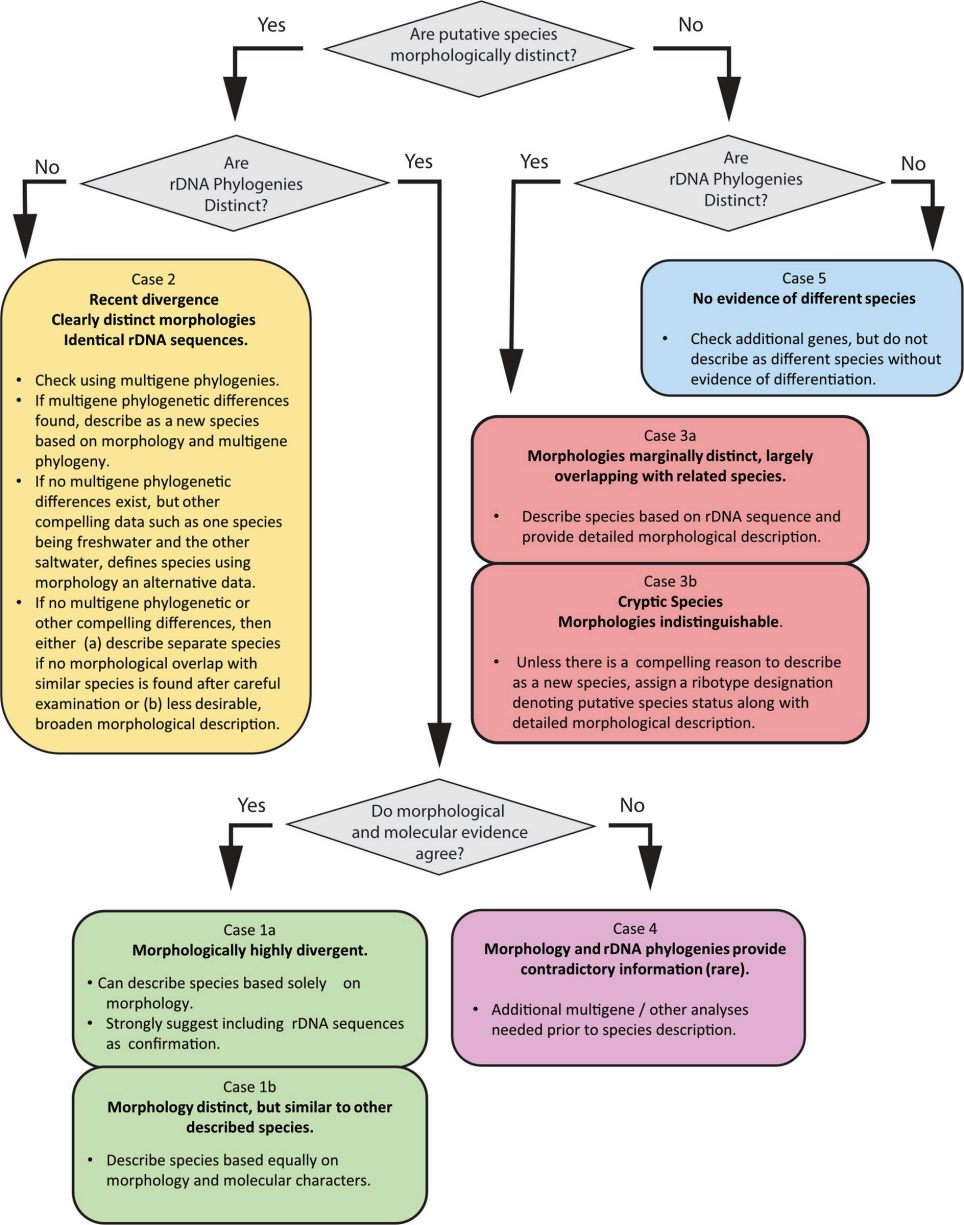
Schematic diagram showing a decision tree outlining how to weight morphological and rDNA phylogenies when defining dinoflagellate species. Here each distinct background color corresponds to one of five case studies illustrating the different nuisances in where and how to weight morphology versus D1-D3 and ITS/5.8S sequence information when defining dinoflagellate species. Cases 1a and b include situations where morphology differences between species are largely distinct and rDNA phylogeny supports morphologically the defined species (green background). Case 2 covers species where morphologies are distinct, but rDNA sequences are equivalent (yellow background). Case 3a encompasses species that are poorly defined morphologically, but whose rDNA phylogeny indicates presence of distinct species (salmon background). Case 3b includes those species where morphologies are indistinguishable, but the corresponding rDNA phylogenies show distinct species present (salmon background). Case 4 indicates the small number of species where the morphological and rDNA sequences provide contridictory information and can only be resolved by acquiring additional data (purple background). Case 5 (light blue background) includes those cases where neither the morphology nor rDNA sequences support establishment of a new species. This flow chart is intended for cases where there is at least some reliable data from both morphological and molecular studies; in cases where such evidence is absent or inconclusive, further study should be carried out before naming new species.

*Case 1a*. *Morphological and molecular evidence agree*, *and morphologies are highly divergent*. Case 1a represents species that are so morphologically distinct, they can be described solely on morphology alone (Figs 5A and 6). In these instances, it can be reasonably argued that there is no need to include ITS/5.8S and D1-D3 rDNA phylogenies in the description. Despite this fact, we contend that both ITS/5.8S and D1-D3 rDNA phylogenies should still be included in the species description. If these rDNA sequences fall into a discrete clades, their inclusion will strongly support the new species description and may subsequently facilitate the identification and description of unrecognized, morphologically similar species. Their inclusion will also facilitate tracking of subsequent nomenclatural changes.

An example of the latter situation is illustrated by the original descriptions of the species pairs *Alexandrium catenella* / *A*. *fundyense* [26] and *A*. *ostenfeldii* / *A*. *peruveanum* [25]. Though each species pair appeared morphologically distinct, reexamination of morphological, molecular, toxicological and other data collected after the original description allowed unabigous designation of *A*. *fundyense* as a synonym of *A*. *catenella* and *A*. *peruveanum* as a synonym of *A*. *ostenfeldii* [25, 26]. The original morphological descriptions were based on geographically distant populations exhibiting morphotypes representing the extreme ends of a morphological continuum. Only after additional sampling was conduced did this continuum become apparent. Had sufficient rDNA sequence data been available at the time the species were originally described, researchers would have recognized the apparently distinct morphotypes as potentially belonging to the same species.

The availability of D1-D3 and ITS/5.8S sequences for each newly described species will consequently make it easier to confirm if new isolates exhibiting unexpected morphologies do, or do not, belong to the same species. Similarly, having access to species-specific rDNA sequence data will facilitate identification and reporting of cases where new isolates, or field collected cells, belonging to the same species exhibit morphological variations not illustrated in the original description. This would allow faster and fuller characterization of the morphological variations beyond those in the original description, which can then be communicated to the broader scientific community. It is also important that, in all cases where molecular data are used as a primary character in defining a species, type genomic DNA for each new species be preserved in a solution designed to prevent degradation and be deposited in an established herbarium or similar collection.

*Case 1b*. *Morphology and molecular evidence agree*, *where morphology is distinct*, *but is found to be similar to other described species*. Case 1b indicates instances where the D1-D3 and ITS/5.8S rDNA phylogenetic analysis unambiguously supports describing a new species and the morphology of the new species is relatively distinct. This includes the key morphological feature(s) defining the species, such as when the size and shape of specific plates which are, on average, distinct, but whose measurements overlap to a small degree with the measurements obtained from morphologically similar, and often closely related, species. Here, cells with features falling in the morphological overlap zone could not be unambiguously assigned to one species or the other.

Species description in these situations would be based equally on morphology and the rDNA phylogenetic data, with more weight ascribed to the molecular data than in Case 1a (Fig 6). The degree of overlap in morphological characteristics with other morphologically similar species should be explicitly stated.

#### Case 2. Morphology is clear and distinct, but the rDNA sequences are identical

The literature survey presented in this study showed ITS/5.8S region phylogenies fail to delineate about 4% of the described species survey and 5’ LSU phylogenies about 7% of the time. These instances most likely represent recently evolved species exhibiting morphological differentiation, but whose rDNA sequences have not yet diverged [75]. Simulation studies have shown that a large number of generations after species diverge, potentially spanning millions of years, are required for there to be a high probability of observing monophyly in a given locus [96, 97]. This may be particularly true for marine species that have more recently reinvaded freshwater habitats [98–101]. A majority of these transitions occurred within the last 40 million years, with most of the diversification of species occurring in the last 25 million years [102].

One of the best documented examples illustrating this situation involves *Scrippsiella hangoei*, which is abundant in the Baltic Sea, and *Peridinum aciculiferum*, which is found in northern temperate lakes [100]. These species are morphologically distinct, occupy radically different habitats, but have identical SSU, ITS and LSU sequences. Laboratory experiments showed *S*. *hangoei* grew in a wide range of salinities (0–30), whereas *P*. *aciculiferum* only grew in low salinities (0–3). In addition, analysis of mitochondrial DNA data are consistent with *Scrippsiella hangoei* as a recent marine ancestor for *P*. *aciculiferum*. Morphology, habitat preference and physiological differences all unambiguously support these as distinct species despite their identical rDNA sequences. Annenkova et al. (2015) extended the previous study, documenting two more morphologically distinct freshwater species–*P*. *euryceps* and *P*. *baicalense*–with identical rDNA sequences to *P*. *aciculiferum*. These observations were consistent with the hypothesis that isolated lake environments foster rapid speciation events. This again supports the possibility that ITS/5.8S and D1-D3 phylogenies may fail to discriminate a greater proportion of freshwater compared to marine or brackish species.

Another subgroup of species falling into this group are those which are morphologically distinct, have indistinguishable rDNA sequences. A good example are some of the described species in the genus *Dinophysis* [19, 78, 80–82, 103–106] (S3 Table in S1 File). This is best illustrated in a recent study by Wolny et al. (2020) [107]. They found two morphologically and molecularly distinct *Dinophysis* species along the mid-Atlantic coast of the United States—*D*. *acuminata* and *D*. *norvegica*. *Dinophysis acuminata* was further found to be morphologically distinct from *D*. *ovum* from the Gulf of Mexico, and *D*. *sacculus* from the western Mediterranean Sea, yet all three of these morphologically distinct species yielded indistinguishable rDNA region sequences. Given the consistency in the morphological differences among *D*. *acuminata*, *D*. *ovum*, *D*. *sacculus*, a logical hypothesis is that members of the *D*. *acuminata* complex are indeed distinct species resulting form a relatively recent rapid radiation event.

Given these species cannot be resolved using rDNA sequences, the question then becomes what other means are required to describe the new species. A potential starting point is to conduct a multigene phylogenetic analysis. If the resulting multigene phylogeny indicate the morphotypes as distinct species, then the species should be described based primarily on morphology and secondarily on the multigene phylogeny results. Alternatively, if other compelling data which differentiate the morphologically distinct species, such as one species being marine and the other one freshwater, mating incompatibility studies, toxicity differences, etc., then one can define species based on morphology and the alternative corroborating data.

If none of the above data support separation, and there is no evidence of morphologically intermediate forms revealed by additional sampling, then two courses of action are possible. The first, and least desirable, is to describe a single species encompassing each of the distinct morphotypes. The other is to describe the distinct morphotypes as new species based solely on morphology and include the information as to which other species contain the equivalent rDNA sequences in the new species descriptions. This later approach, however, should only be employed after determining if the multigene phylogeny or other more extensive morphological studies also support describing a new species. This will help in distinguishing these cases from those such as *A*. *catenella* where relatively distinct morpholotype of the same species predominates in different regions [26]. As note above, sampling from these different populations accounted for why *A*. *catenella* and *A*. *fundyense* were originally designated as distinct species, rather than the single species of *A*. *catenella*. Only after the morphology of cells from many different populations were examined did the broad continuum of morphotytpes exhibited by this single species became apparent. This was bolstered by all the morphologically distinct cells yielding equivalent rDNA sequences.

*Case 3a*. *The rDNA phylogenies are disctinct*, *but morphologies largely overlap*. Under these circumstances, the distinct species-specific rDNA clusters present in the phylogeny can be used as the primary character upon which a new species is described, along with a detailed morphological description and the known overlap with other species (Figs 5B and 6). When only field collected cells are available for carrying out the morphological characterization, the corresponding rDNA or transcriptomic sequences can generally be obtained using single cell PCR amplification methods [108–112]. Once obtained, as in the case of achieving type genomic DNA in a herbarium, anytime rDNA sequences are used as a primary character for delineating species, representative rDNA sequences should be deposited in GenBank and listed in the species description paper as type DNA sequences.

When analyzing the phylogenetic clusters for identifying species in these situations, it is important to note that some species exhibit extensive inter-locus sequence variation as noted above. In haploid dinoflagellates, the number of rDNA loci can range from approximately 5 to >100,000 copies per cell. In a phylogenetic analysis, many of these pseudogenes can form sub-clusters within the main species-specific cluster [50, 53, 76, 113–118]. When this occurs, comparing the terminal nodes in the ITS and D1-D3 phylogenies will delineate the point at which inter-specific variation transitions to intra- specific variation. Another aspect of relying on molecular phylogenic analyses in describing species which bares emphasizing is that inclusion of multiple sequences from different isolates is preferable to using a single sequence to identify new species. Phylogenetic programs often have issues placing a single species due to long branch attraction issues. Thus, using multiple sequences provides information on the intraspecific rDNA variation in the new species and helps further confirm a given clade is distinct.

Though not required, further confirmation that molecularly defined species are valid can potentially be obtained over time in cases where the species occur sympatrically. As shown in Fig 5C, when isolates of correctly defined species from the same region are sequenced, the resulting ITS/5.8S and D1-D3 phylogenies will consistently produce unique, species-specific clades. The existence of these non-overlapping clades constitutes strong evidence that the species are reproductively isolated.

*Case 3b*. *Morphologies are indistinguishable (cryptic species)*, *though the rDNA phylogenies are distinct*. Here, new species would be described based on the molecular phylogenies alone [27, 119–121] (Fig 6). Though justified, this practice raises an interesting conundrum with regard to the ultimate goal of taxonomy, which is to serve the needs of the broader scientific community. Literally following the molecular-based description approach would result in a plethora of new cryptic species being described. Having so many morphologically identical species described may unnecessarily complicate how data from ecological or other studies are reported and interpreted. In most cases, there will be no known ecological, toxicological or other important functional difference between these cryptic species. The question then becomes: what practical reason would there be for describing these genetically distinct species? There is no unequivocal answer to this question.

We suggest for cases where there is no compelling ecological, toxicological or other reason for distinguishing the cryptic species, a more prudent approach would be to assign the cryptic species a unique ribotype identifier–e.g. “genus name cf. ribotype 1, 2… etc.”–without formally describing the species [28, 122]. Once the function of the ribotype is established, a full morphological and molecular description would be warranted. This use of ribotypes would alert the scientific community when cells with a given morphology may represent a complex of indistinguishable cryptic species. It would simultaneously discourage a proliferation of named species that may confuse more than enlighten work in other disciplines.

Alternatively, one could convincingly argue from a purely taxonomic standpoint that all species should be described regardless of their known functional significance. Definitive resolution of this issue is beyond the scope of this study, but is worth recognizing as a basis for the inevitable debate regarding the degree to which cryptic species are formally described based on molecular data. Some resolution of this issue is clearly needed as the literature survey identified more than 315 distinct ribotypes that could be identified as distinct species (S3 Table in S1 File). Regardless of which approach is taken, we again stress that the fullest morphological description possible should always accompany any new species or ribotype being described.

#### Case 4. Morphology and rDNA phylogenies provide contradictory information (rare)

In a small number of cases, neither morphology nor molecular rDNA phylogenies provide a consistent picture of where to draw species boundaries (Fig 6). A good example is the freshwater dinoflagellate *Peridinium cinctum*. This species is considered a widely dispersed generalist and the type species for the genus *Peridinium*. López et al. (2018) [123] obtained detailed morphological observations as well as ITS sequences from 15 strains collected from different freshwater reservoirs across central Europe. These 15 strains were representative of the variation across ribotypes and different localities. They identified three distinct and one less defined morphotype expressed among these isolates. When a hundred or more cells from cultures of the separate isolates were examined, each was found to express the 4 different morphotypes, but in different proportions. The study results also indicated five different ITS ribotypes were present, which failed to correlate with the variations in the dominant morphotypes observed in each isolate. Given these conflicting morphological and molecular datasets, it is currently unclear as to where species boundaries should be drawn. The efficacy of using more extensive multigene phylogenies to resolve such situations is unknown and will need to be tested using a broad sampling of isolates. In these types of cases, more isolates, preferentially from different locations, should be obtained, and multigene or other analyses such a mating studies, habitat preferences, etc. should be conducted to try and resolve the conflicting information prior to describing species.

#### Case 5. No evidence of different species

This represents those situations where neither morphology or rDNA sequences support the existence of separate species. In this case, other genes can be sequenced, but species should not be described without concrete evidence to support species level differentiation.

### Other advantages of including D1-D3 data as an integral aspect of dinoflagellate species descriptions

Another advantage for including D1-D3 sequence data as an integral part of dinoflagellate species description it that it can aid biogeographical studies. As high throughput sequencing becomes more affordable and common, it will be possible to screen the dinoflagellate species composition from an ever-increasing number of environments. If enough species-specific D1-D3 data are available, and this readily amplifiable region is targeted for study, it will simplify identification of known species present at the different locations. The major caveat is that though the vast majority of species can be identified this way, there will still be relatively small number of species that cannot.

Utilizing the D1-D3 (and ITS/5.8S) as a definitive character would also allow unprecedented freedom to publish more robust species descriptions emphasizing the full morphological variation observed within a species. Currently, the emphasis when describing a species morphologically is to find the character(s) that most clearly distinguish that species. This often limits the micrographs presented in the description to those best distinguishing the proposed differences between species. Those defining characters, however, may only be fully expressed in log phase growth or other conditions. Not showing the fuller representation of intraspecific morphological variation makes it more difficult for other non-taxonomists to identify which species they encounter in ecological or other related field studies. Relying primarily on the molecular character would remove the need to show the most dramatic morphological differences. It will also promote an understanding that particular morphologies may represent more than one species and that this must be accounted for when doing field or monitoring studies. Adopting this approach is particularly important in instances where closely-related, morphologically-indistinguishable species co-occur, some of which are toxic and others are not. Further, having the D1-D3 sequences will also make it easier for non-taxonomists to track and understand ongoing and historical nomenclatural revisions.

### Future directions

At present, the ITS/5.8S and rDNA D1-D3 genes represent relatively easy to acquire sequence data that, in combination, accurately delineate 93–97% of described dinoflagellates studied to date. In the future, multigene approaches are likely to provide even better delineation of species and superior insights to phylogenetic relationships. Such multi-locus approaches are starting to become more common and will most likely be the preferred method of molecularly delineating species in the future [40, 124, 125]. However, at present, these approaches are still relatively expensive and the metagenomic pipelines necessary for routinely eliminating paralogues and constructing unbiased multigene phylogenies have not been standardized [77]. During the interim period where the cost of doing multi-locus phylogenies declines to more affordable levels, and processing pipelines are standardized, we recommend a reliance on the ITS/5.8S and D1-D3 rDNA sequences as key characters in defining a majority of dinoflagellate species. If resources allow, the data in S3 Table in S1 File indicate including the SSU and D8-D10 regions in the analysis will only further strengthen the ability to delineate dinoflagellate species. This approach has been successfully employed by Gottschling *et al*., 2020, 2021 [126, 127].

Despite this success, an important caveat, regardless of what rDNA genes are used, is that these loci will not work universally. The case studies presented above, however, can provide a systematic guide for recognizing and evaluating when defining species using these loci is inappropriate and what next steps are needed to deal with this situation.

## Conclusions

Using transcriptomic data from numerous *Gambierdiscus* species, as well as transcriptomic data from related species, showed the D1-D3 rDNA and ITS/5.8S region phylogenies revealed the same species groups as obtained using multigene phylogenies. These findings support using the D1-D3 and ITS/5.8S regions as a rapid, reliable method for identifying and describing *Gambierdiscus* species. The multigene phylogenies, however, produced a more robust determination of the evolutionary relationship among species and indicated *G*. *belizeanus*, *G*. *pacificus* and *Gambierdiscus* sp. ribotype 2 arose from a rapid radiation event. Cumulatively, the data from the transcriptomic and single gene phylogenies indicated a high likelihood that D1-D3 and ITS/5.8S could successfully identify a broad range of dinoflagellate species. This possibility was tested and confirmed through a literature survey of 473 articles conducted to determine what percentage of described species could be identified using phylogenies based on either the SSU, ITS/5.8S and D1-D3 LSU rDNA regions. A total 863 species for which a phylogeny using at least one of these three rDNA regions were identified, as well as over 315 ribotypes that likely represent other distinct species. Overall, the SSU and D1-D3 sequences had similar success as delineating >93% of the dinoflagellate species surveyed, with the ITS/5.8S region capable of resolving >97% of species (Table 3). Arguments are presented for why D1-D3 in combination with ITS/5.8S phylogenies are the preferred sequences for use in describing new dinoflagellate species.

Given that the phylogenies were not completely consistent at delineating species, a systematic scheme for determining when and how the combined D1-D3 and ITS/5.8S rDNA regions can be employed as the primary character in identifying new dinoflagellate species is presented (Fig 6). Application of that protocol depends on the degree of morphological variation among species and the concordance with the molecular analyses, and will likely work for a majority of dinoflagellates species. The cases where the D1-D3 and ITS/5.8S phylogenies will most likely fail to distinguish species are in the relatively small number of recently evolved species, where sequence divergence in these genes is insufficient [75]. In these cases, multigene phylogenies, mating studies and even more detailed morphological studies will be required. In the future, as sequencing methods continue to advance, it is likely new dinoflagellate species descriptions will depend on both morphology and multigene phylogenies, including rDNA sequences from numerous isolates. In the interim, D1-D3 and ITS/5.8S rDNA sequences can serve to bridge this gap.

Whether the question at hand is environmental molecular (metagenomic) datasets, cryptic species among morphologically described strains, or morphologically divergent strains with no identifiable molecular characters, there will no doubt be discomfort with the idea of giving priority to molecular characters when there is discordance with (or a lack of information regarding) morphological characters. We argue, however, that this approach in no way diminishes the importance of morphological study of dinoflagellates. In fact, trusting the molecular data frees up morphological analysis to examine the environmental and evolutionary significance of structure. Rather than relying on morphological characters alone to identify species or other units of study, this approach will make it possible to determine when particular morphologies correlate with properties of interest (e.g., toxin production) and when they do not [39]. As such, morphological analysis will remain a vital and crucial part of the study of dinoflagellates, but as molecular data become increasingly widely available, having a clear understanding of the relationships among single-gene, multi-gene, and morphological diagnoses of species will be essential to accurately interpreting the information available.

## Supporting information

S1 File Singular file containing all supplementary information. This file contains a) S1-S45 Figs, individual gene trees obtained from CEGMA, BUSCO, and Janouškovec *et al*., 2017; b) S46 Fig, the results of the parametric bootstrapping for each individual gene used for the multigene phylogenies; c) S1 Table, the indices used for generating the Illumina transcriptome libraries; d) S2 Table, a list of all individual genes used for generating the multigene phylogenies; e) S3 Table, the literature survey for how well rDNA phylogenies distinguish species in different dinoflagellate genera, including all citations.
(PDF)

## Abbreviations

BUSCO: Benchmarking Universal Single-Copy Orthologs
CEGMA: Core Eukaryotic Gene Mapping Approach
ITS: Internal transcribed spacer region
LSU: Large subunit
rDNA: ribosomal genes
