# Supplementary material for: Using RDNA sequences to define dinoflagellate species
Source: PLoS One. 2022 Feb 25;17(2):e0264143. doi: 10.1371/journal.pone.0264143 (PMC8880924; doi:10.1371/journal.pone.0264143)
Supplement: S1 File — This file contains a) S1-S45 Figs, individual gene trees obtained from CEGMA, BUSCO, and Janouškovec et al., 2017; b) S46 Fig, the results of the parametric bootstrapping for each individual gene used for the multigene phylogenies; c) S1 Table, the indices used for generating the Illumina transcriptome libraries; d) S2 Table, a list of all individual genes used for generating the multigene phylogenies; e) S3 Table, the literature survey for how well rDNA phylogenies distinguish species in different dinoflagellate genera, including all citations. (PDF) [file pone.0264143.s001.pdf]

Supplemental Figures 1-45. Individual gene trees obtained from CEGMA, BUSCO, and Janouškovec *et al*., 2017, using the Maximum Likelihood algorithm with 500 bootstraps. Details regarding each gene can be found in Supplementary Table 2. This has also been posted to figshare: https://doi.org/10.6084/m9.figshare.12636017.v1

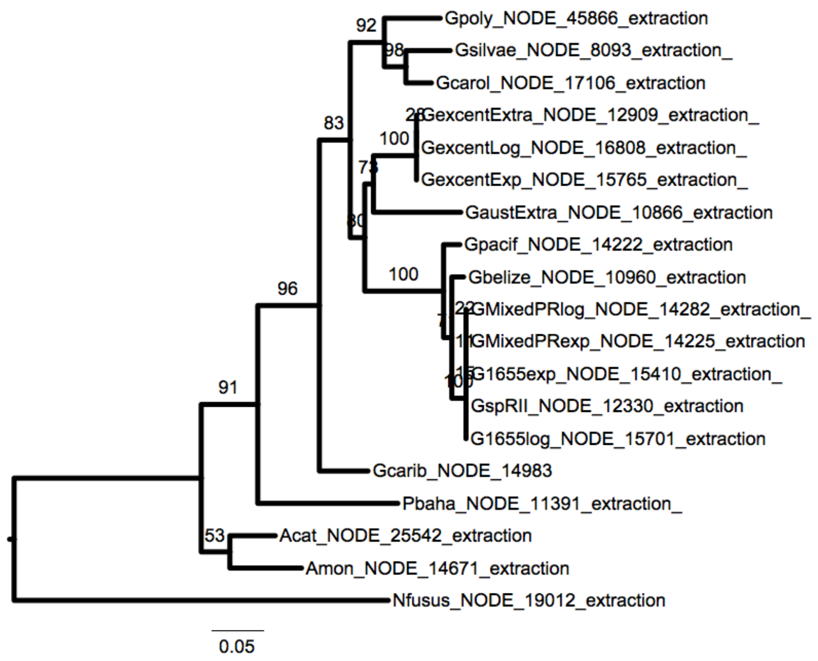

Figure 1: Gene atp6v1b

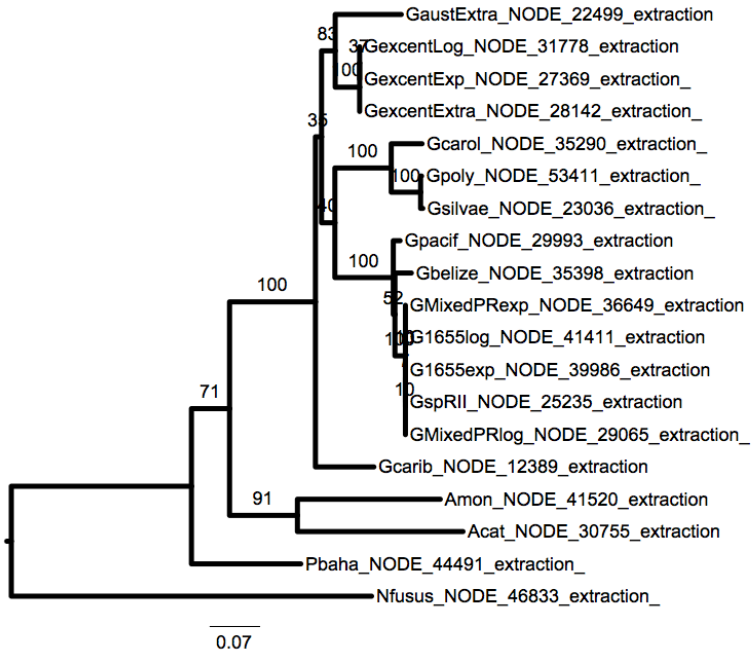

Figure 2. Gene atp6v1d

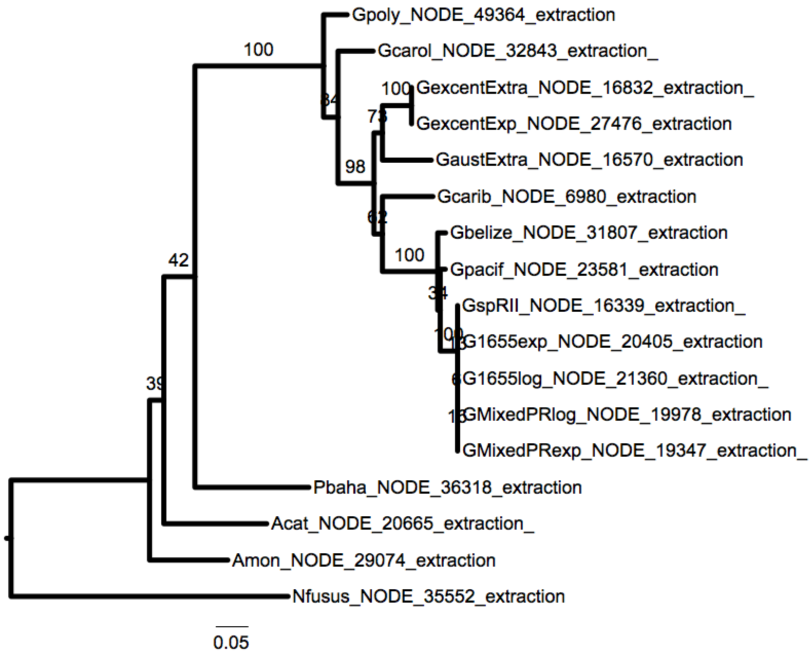

Figure 3. Gene dimt1l

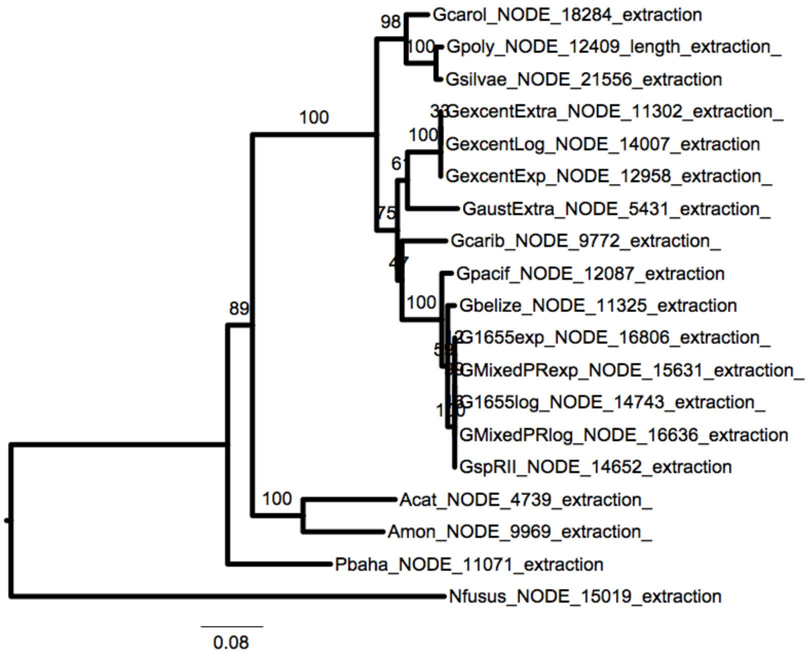

Figure 4. Gene dnai2

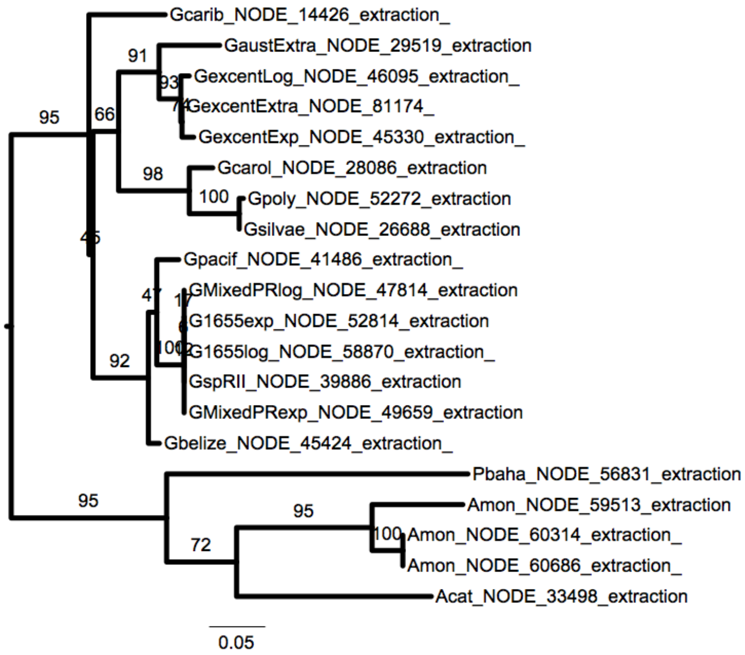

Figure 5. Gene eif2b

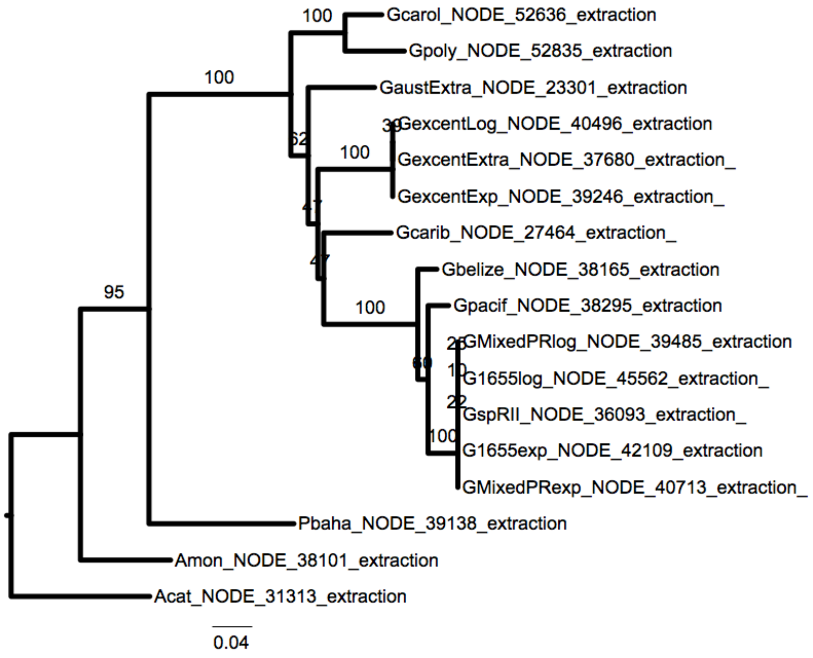

Figure 6. Gene emg1

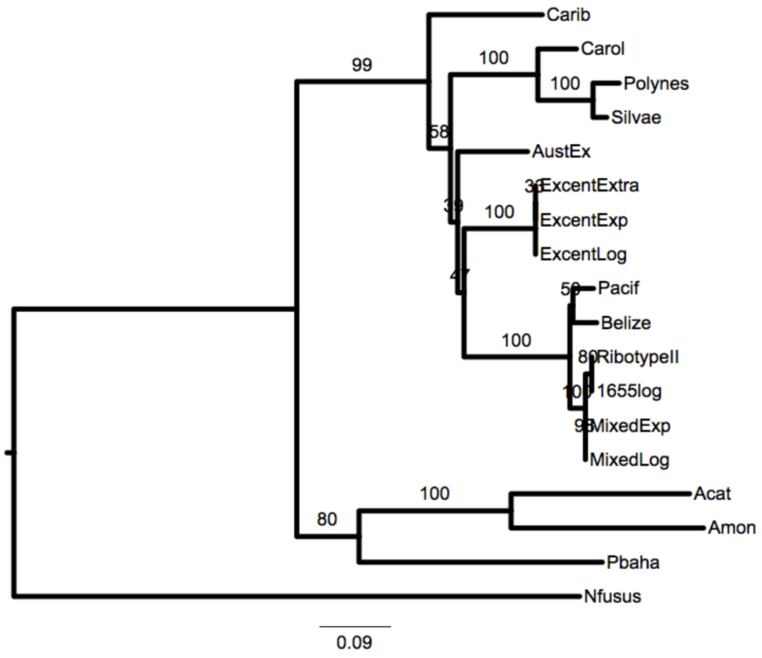

Figure 7. Gene EOG09370DP4

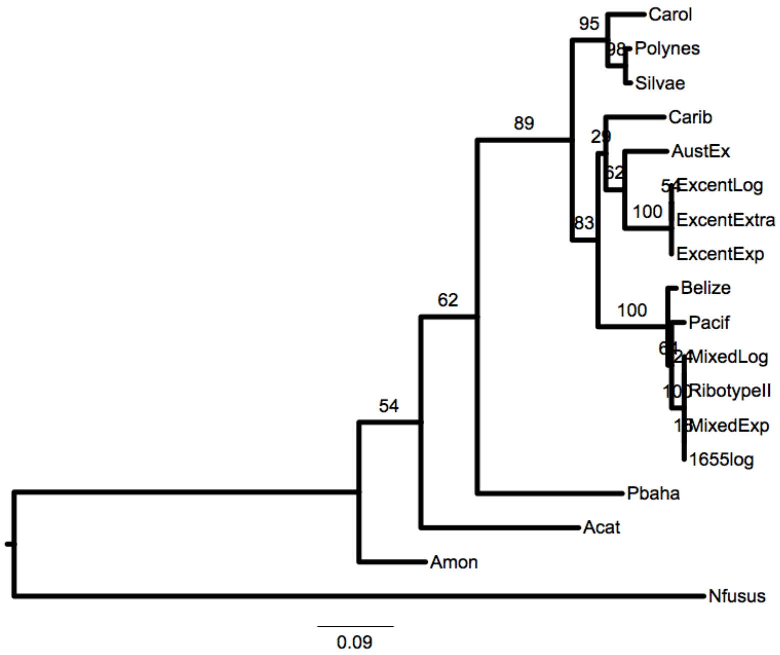

Figure 8. Gene EOG09370FLD

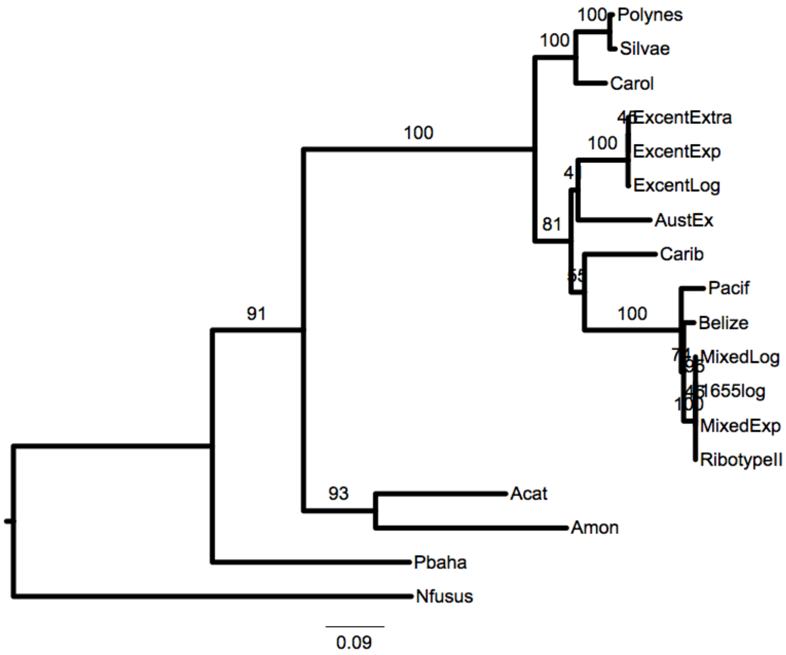

Figure 9. EOG09370FSS

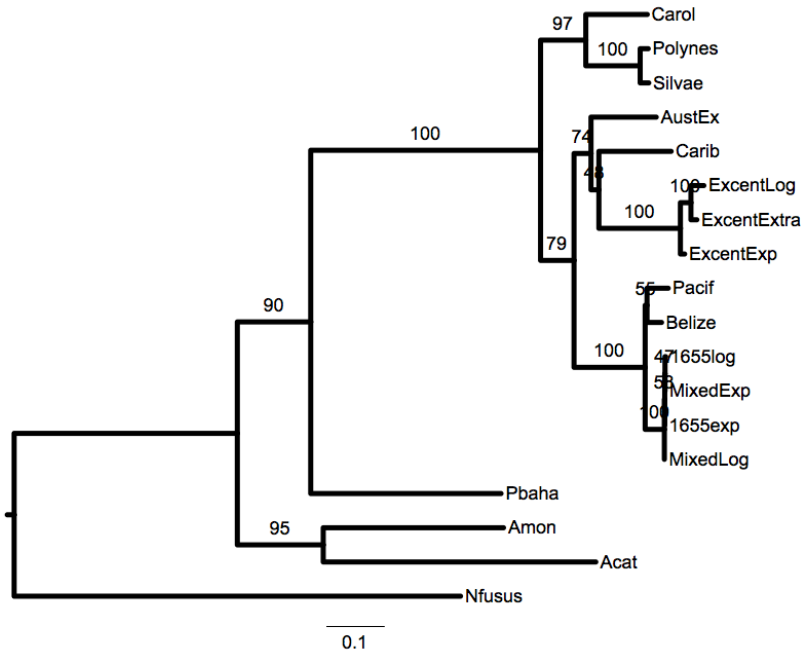

Figure 10. EOG09370IH5

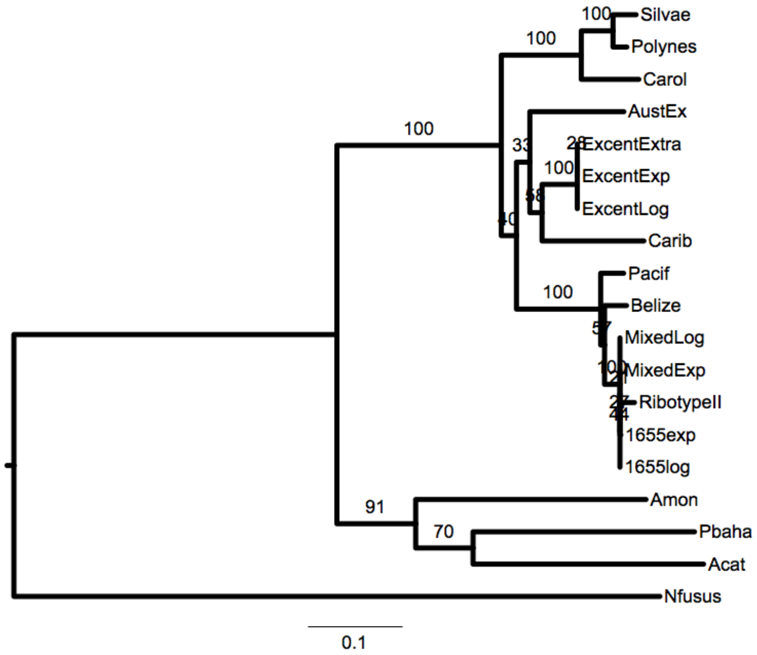

Figure 11. EOG09370IUA

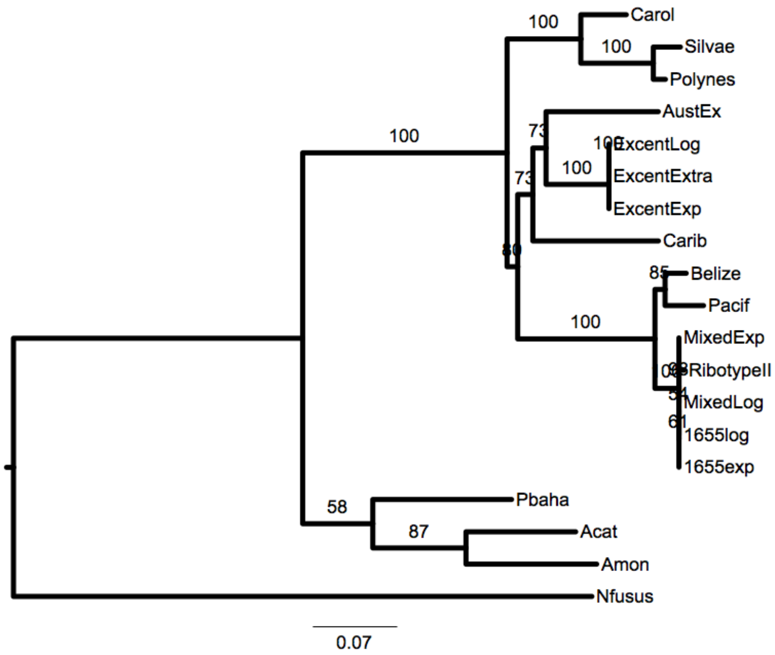

Figure 12. Gene EOG09370KD4

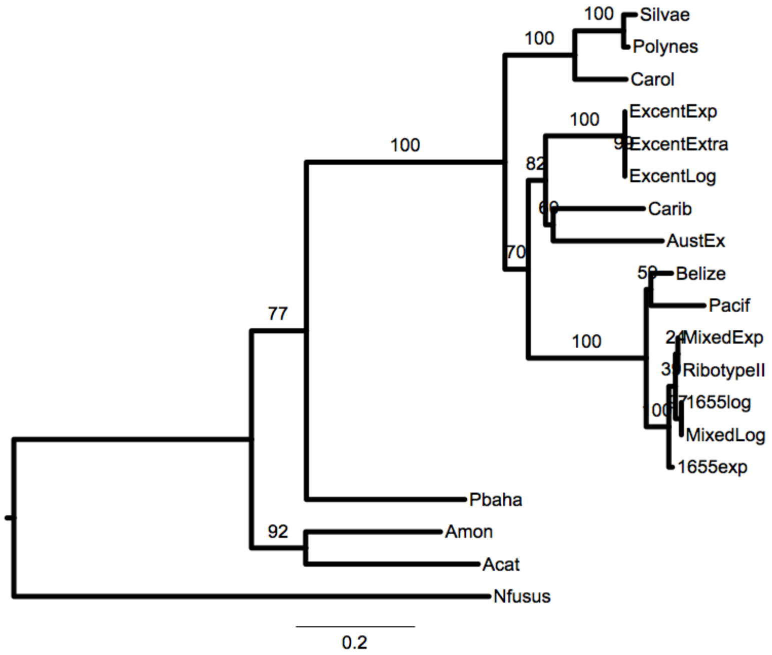

Figure 13. Gene EOG09370MQO

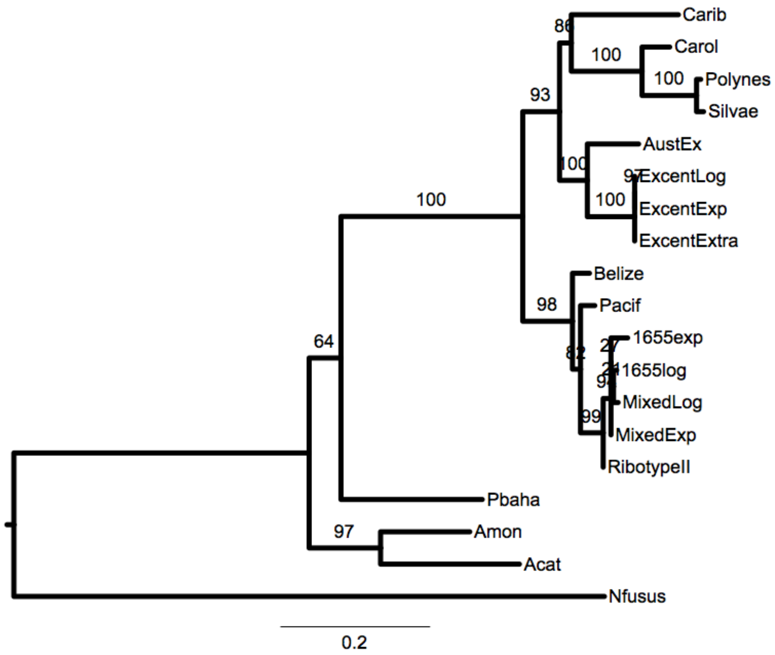

Figure 14. Gene EOG09370NBT

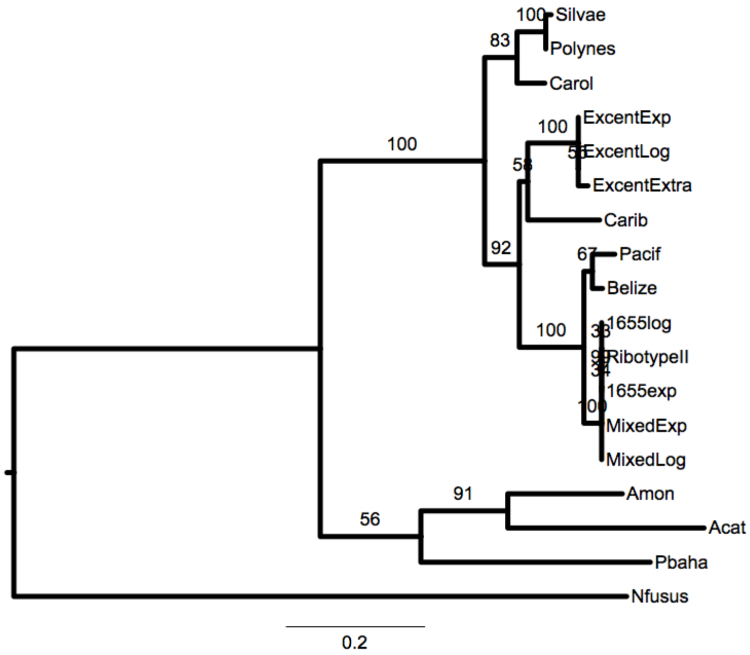

Figure 15. Gene EOG09370OLY

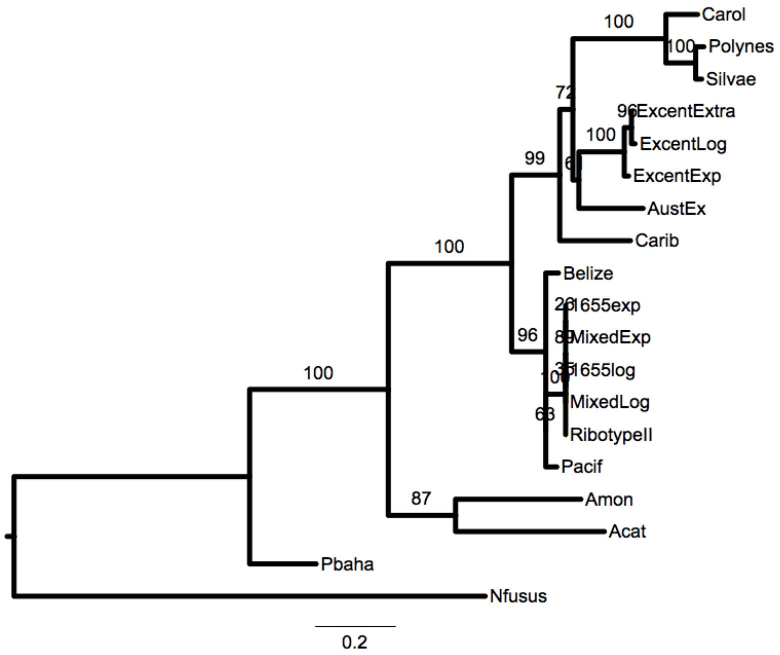

Figure 16. Gene EOG09370S0F

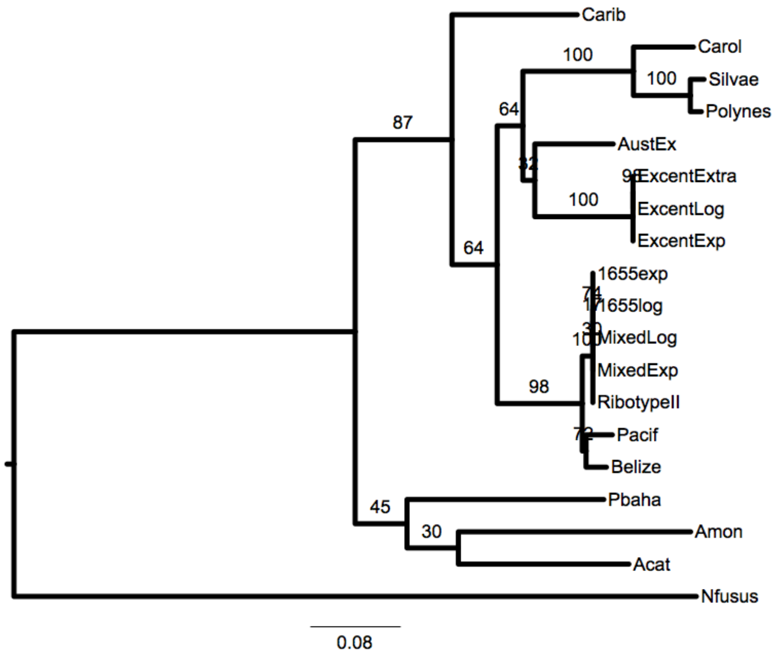

Figure 17. Gene EOG09370S0N

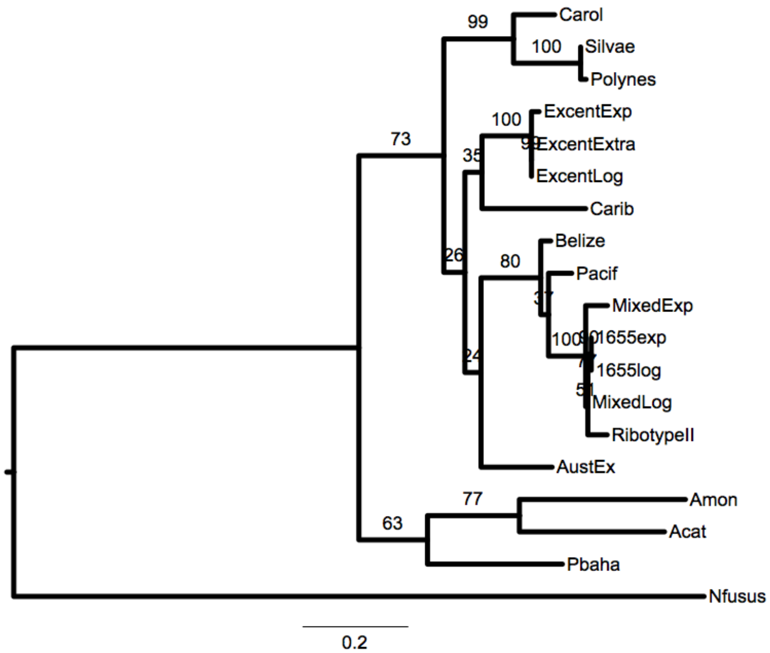

Figure 18. Gene EOG09370SV2

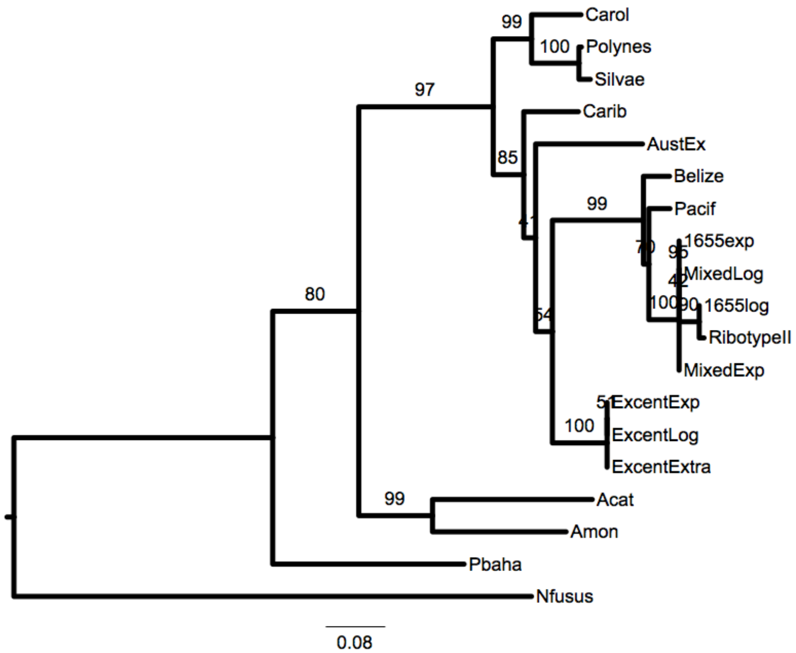

Figure 19. Gene EOG09370TCX

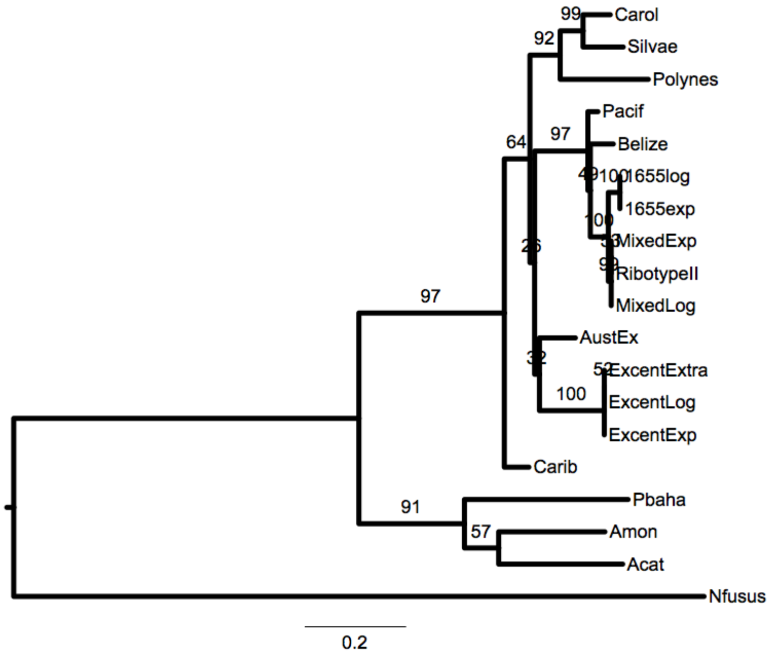

Figure 20. Gene EOG09370TH4

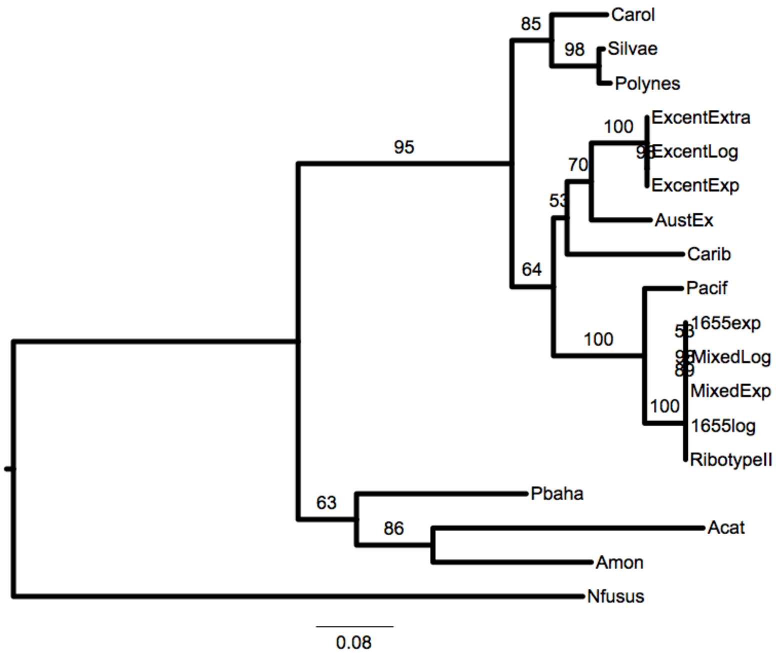

Figure 21. Gene EOG09370U0A

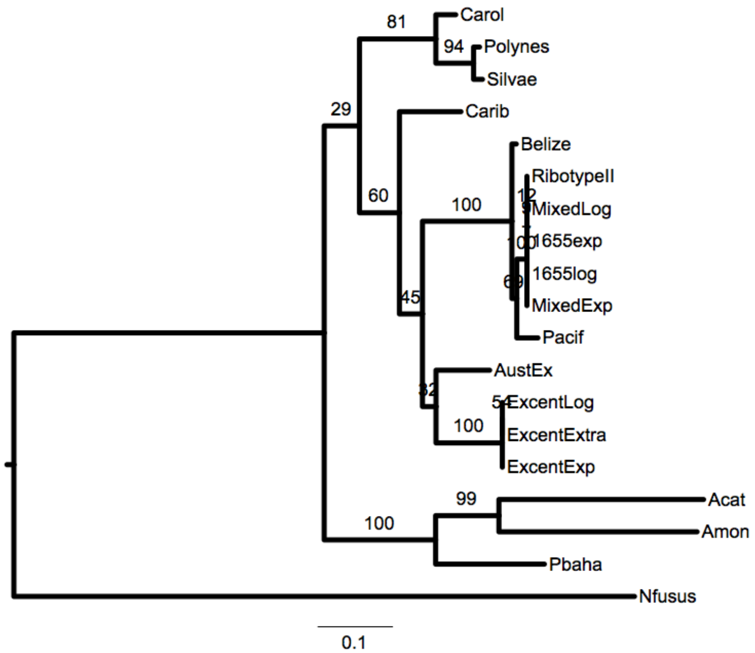

Figure 22. Gene EOG09370W6N

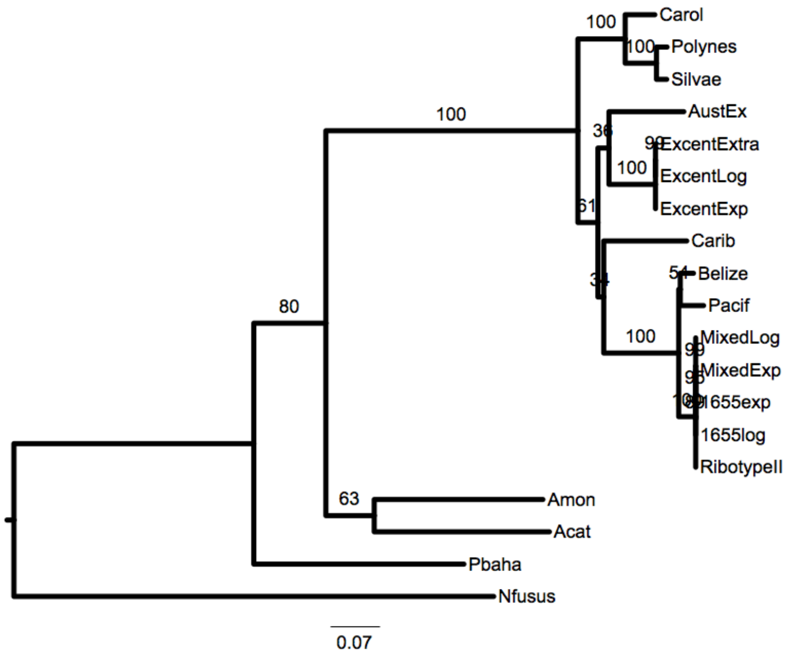

Figure 23. Gene EOG093702PE

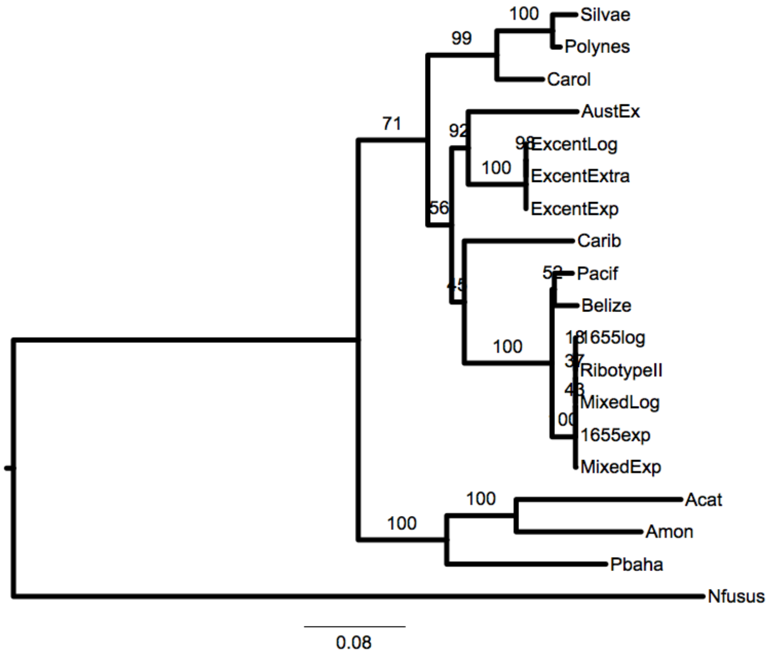

Figure 24. Gene EOG093703UX

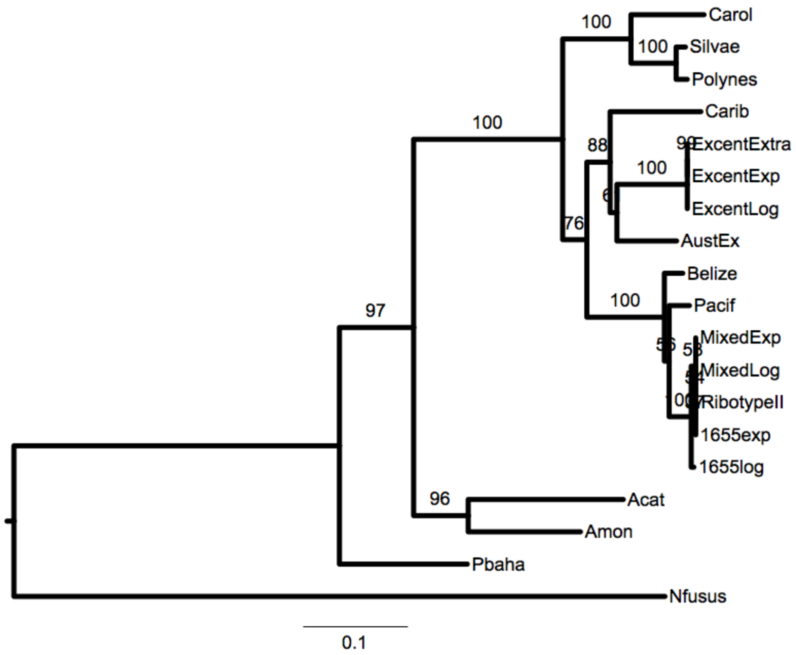

Figure 25. Gene EOG093705EY

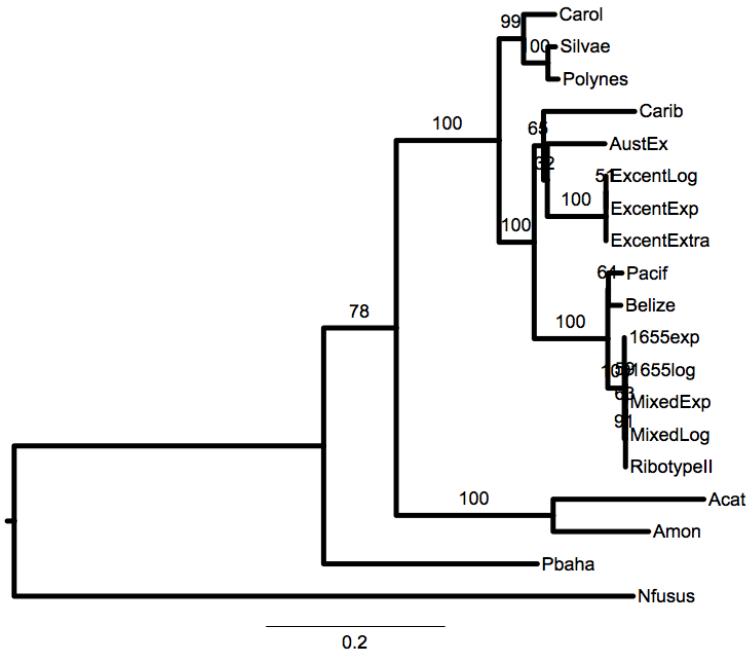

Figure 26. Gene EOG093706RN

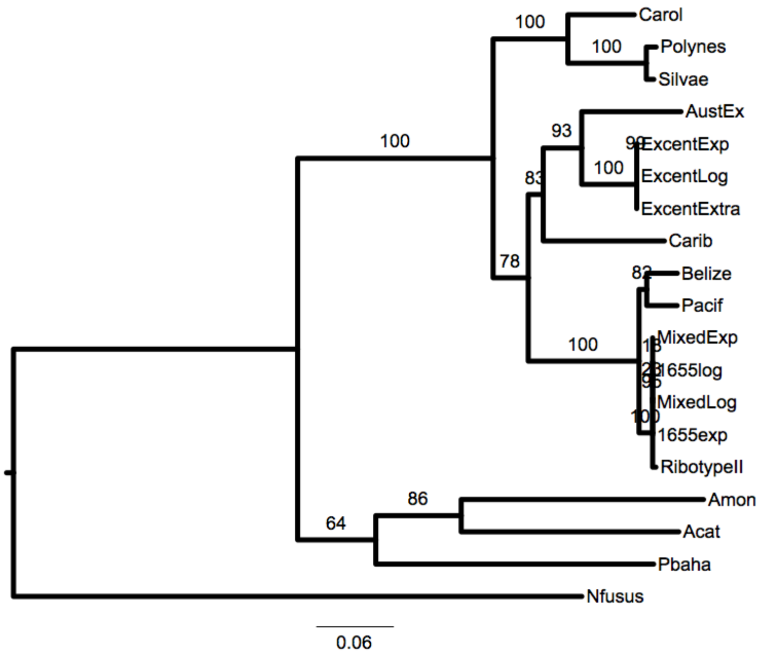

Figure 27. Gene EOG093707HK

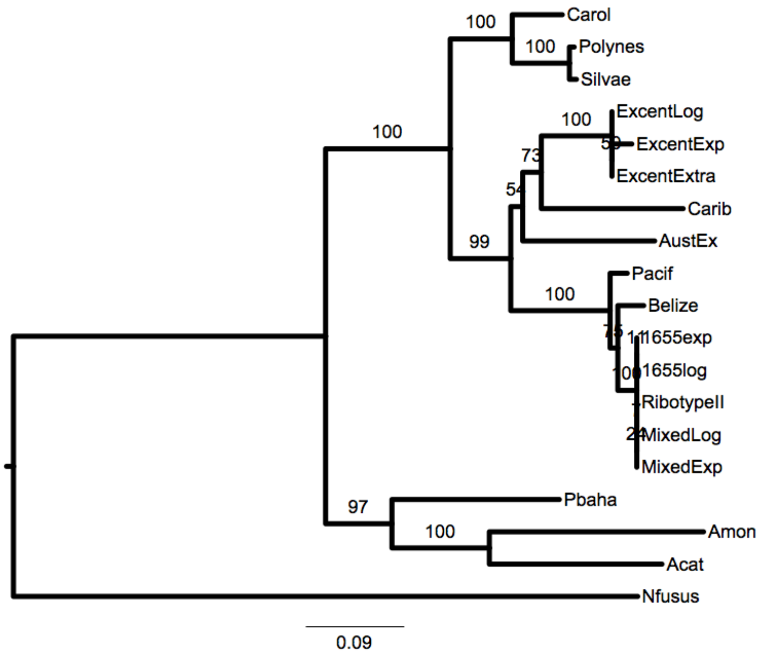

Figure 28. Gene EOG0937085E

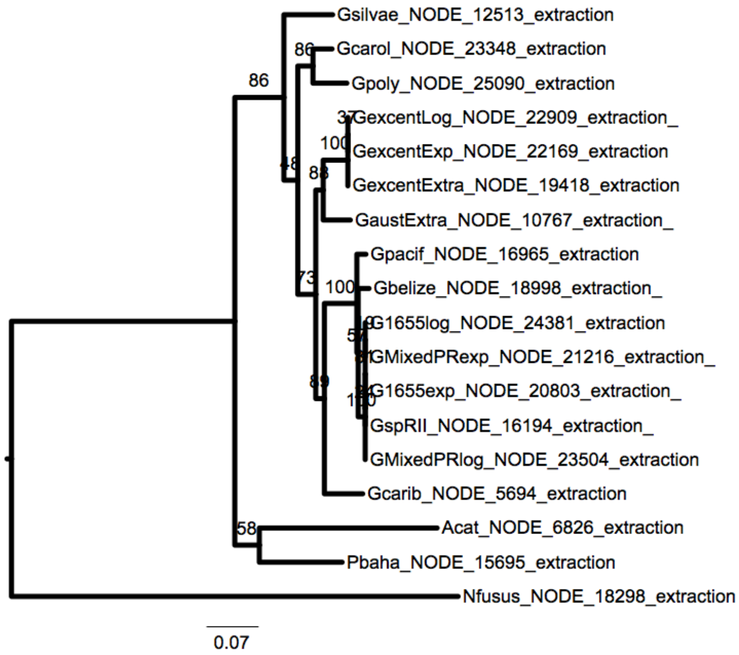

Figure 29. Gene etf1

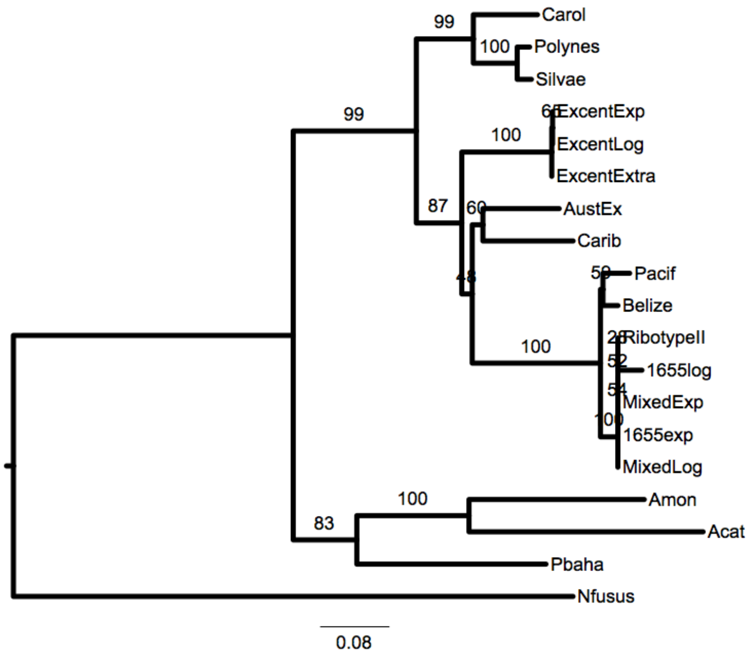

Figure 30. Gene KOG0530

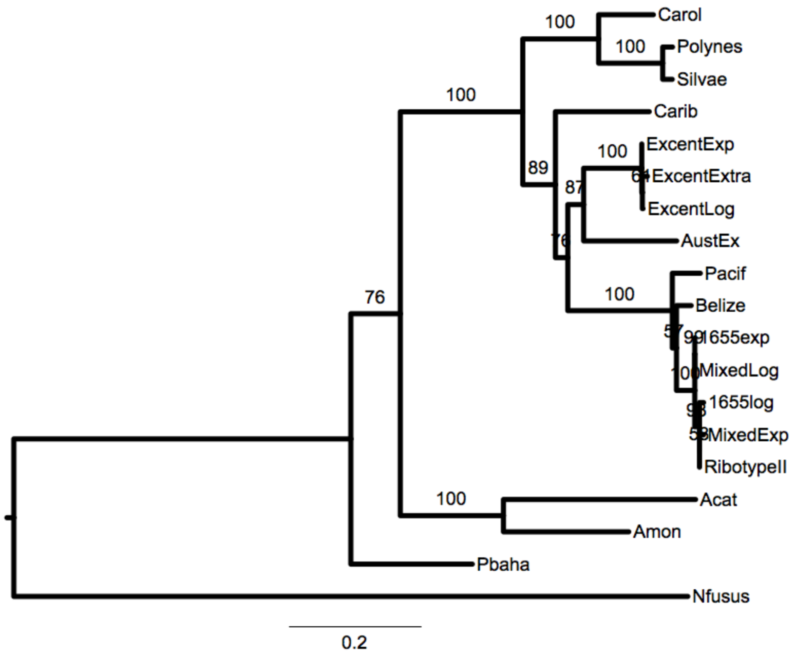

Figure 31. Gene KOG1272

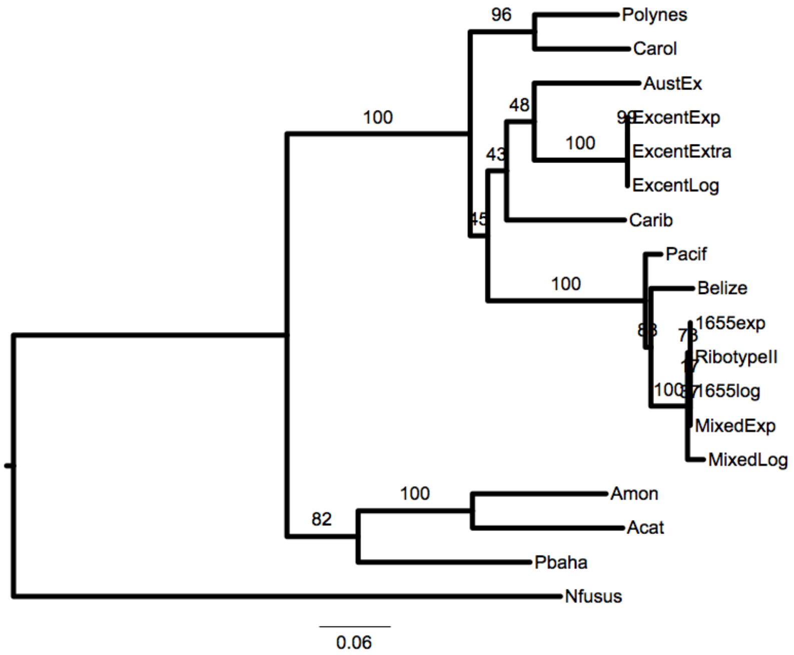

Figure 32. Gene KOG2781

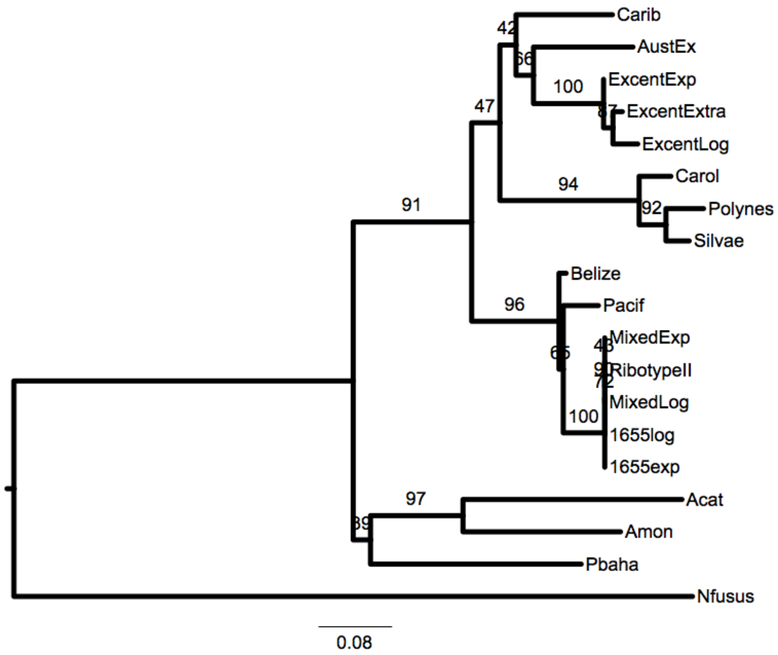

Figure 33. Gene KOG3048

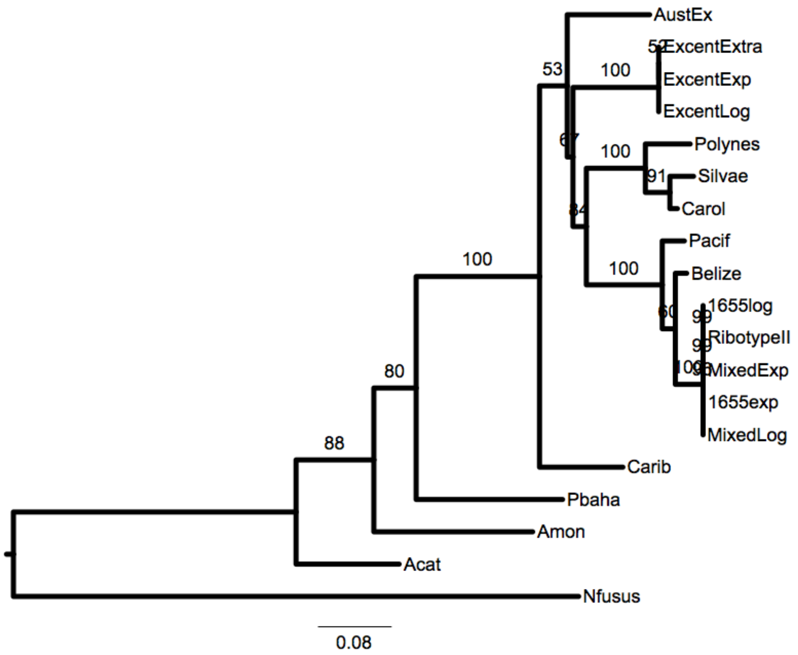

Figure 34. Gene KOG3189

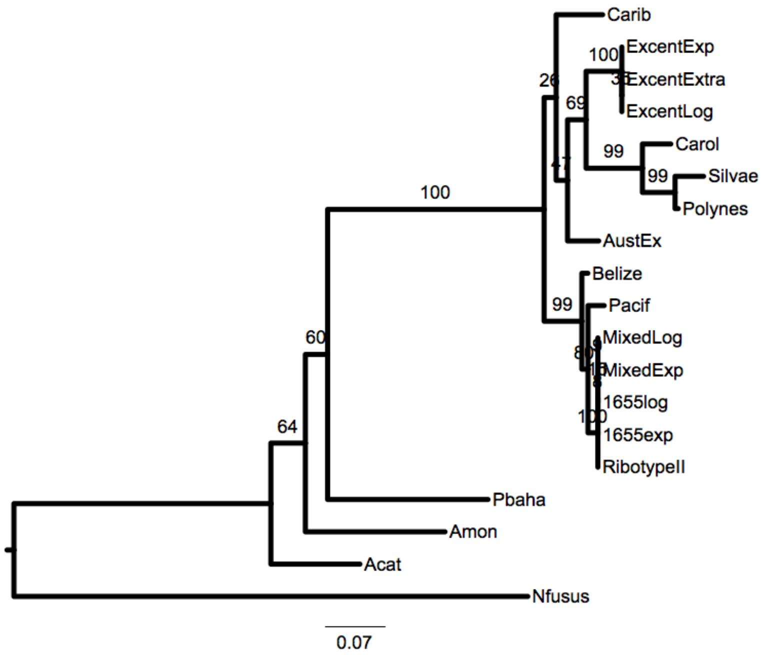

Figure 35. Gene KOG4392

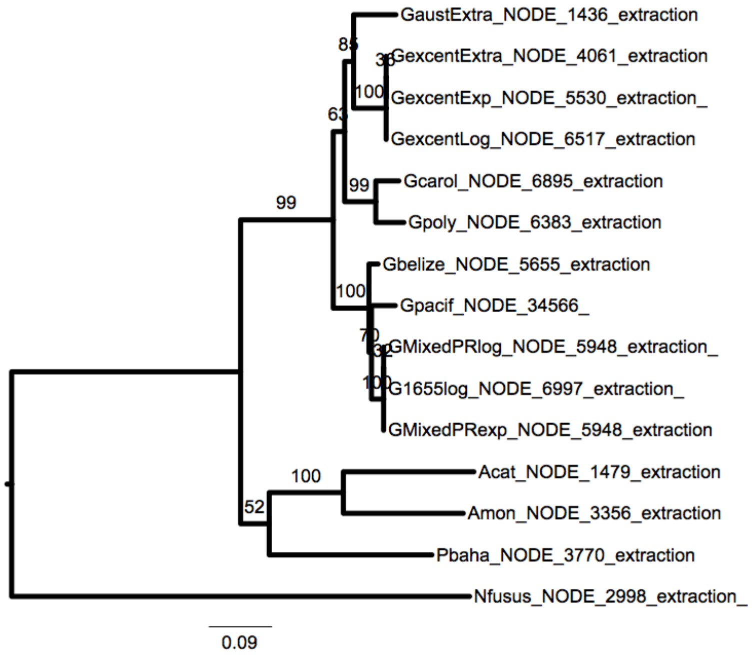

Figure 36. Gene mcm6

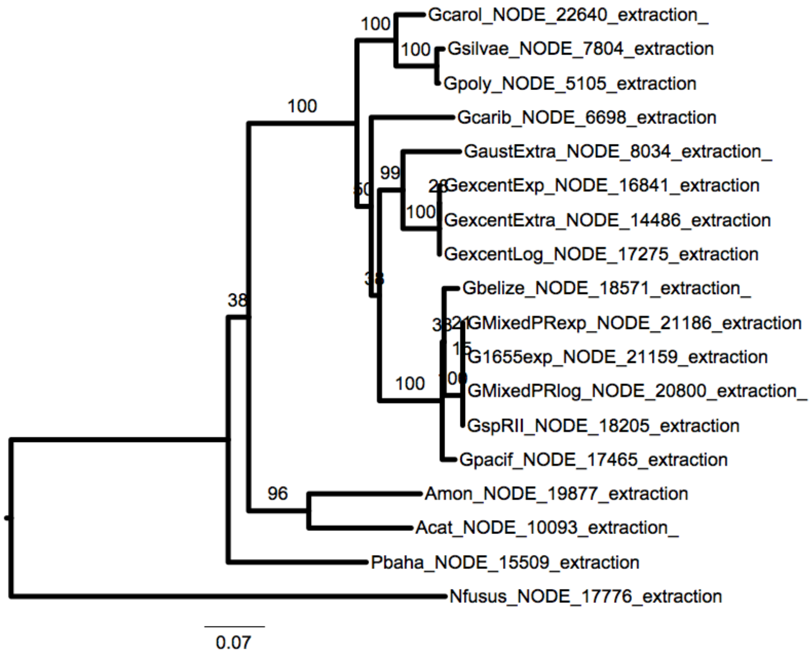

Figure 37. Gene nop56

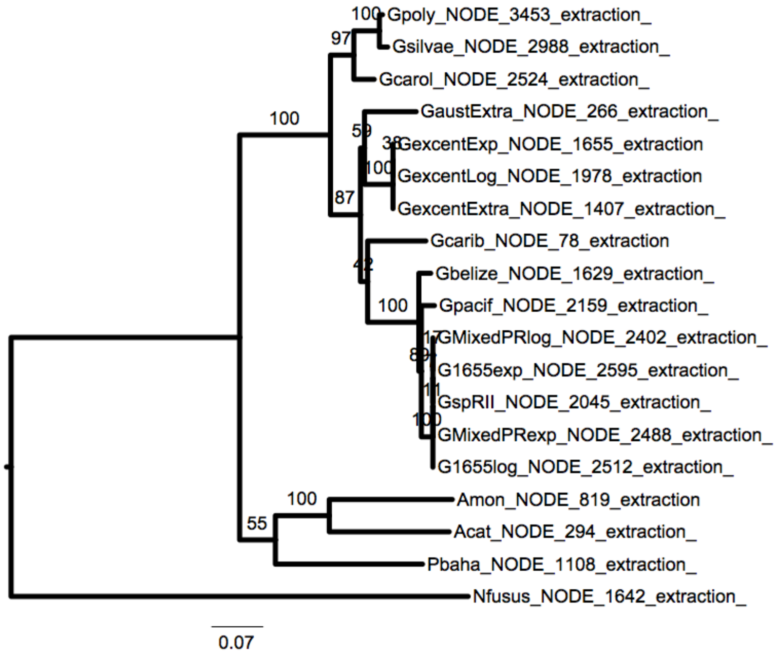

Figure38. Gene pmsd1

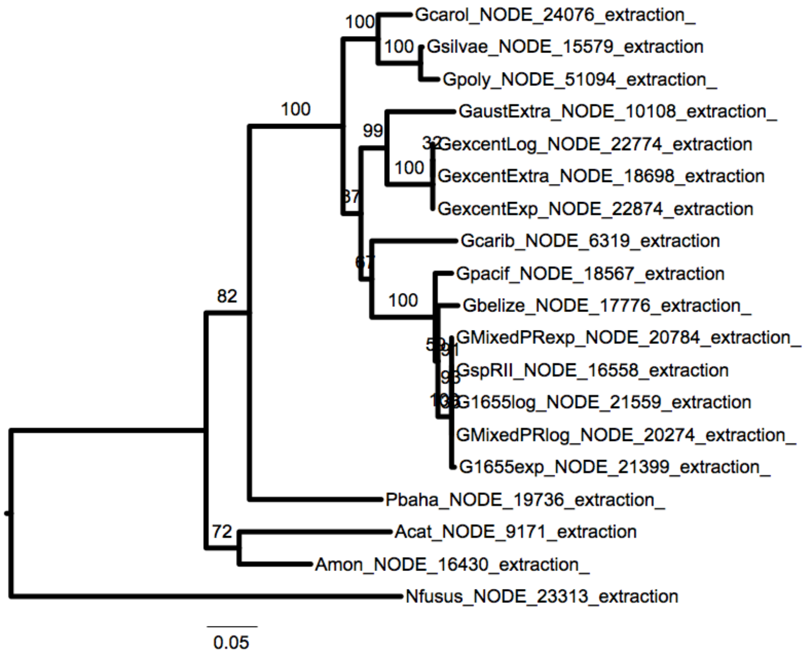

Figure39. Gene pmsd12

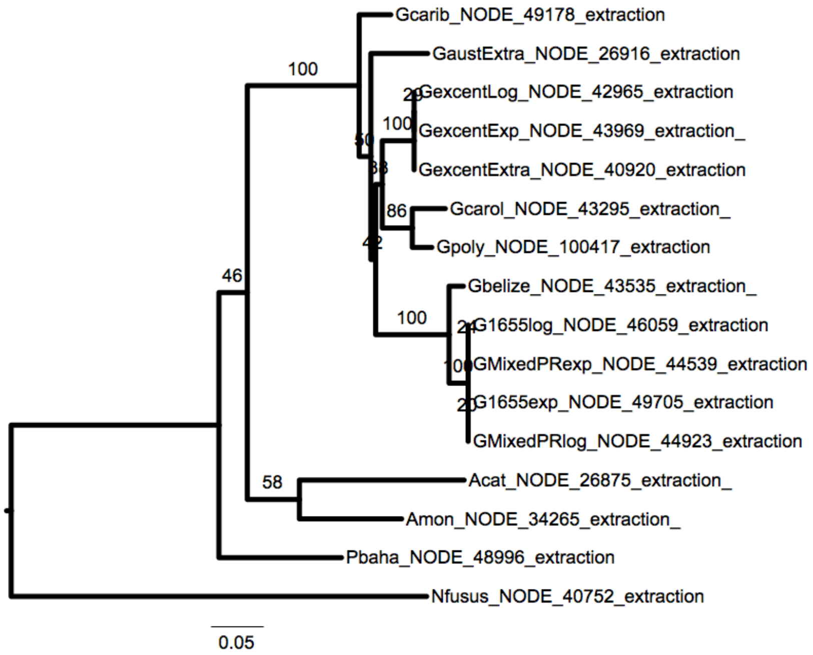

Figure 40. Gene psma5

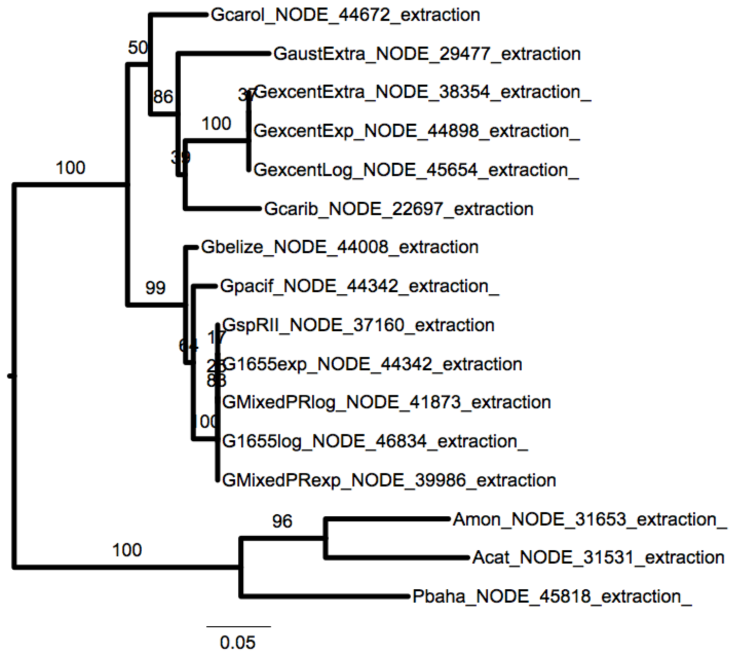

Figure 41. Gene psma7

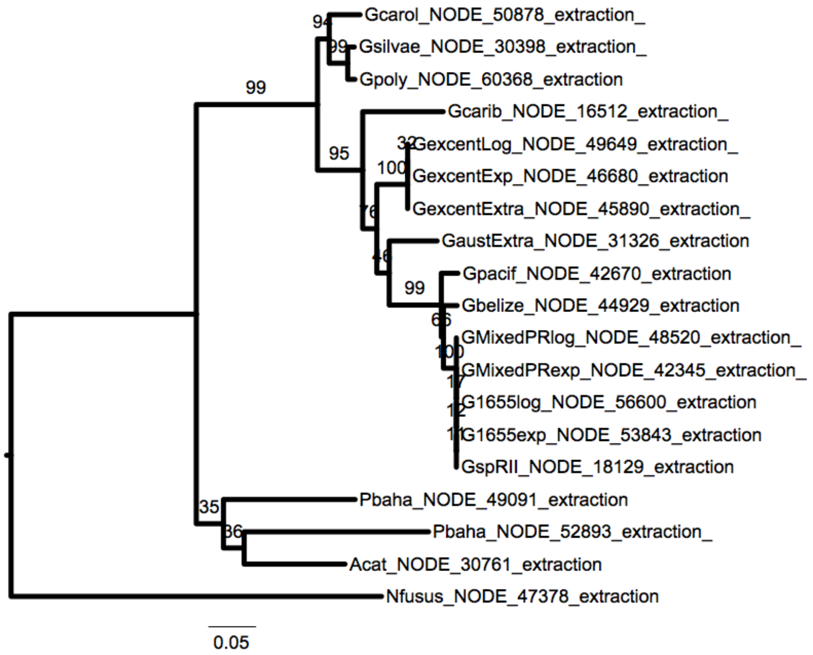

Figure 42. Gene psmb1

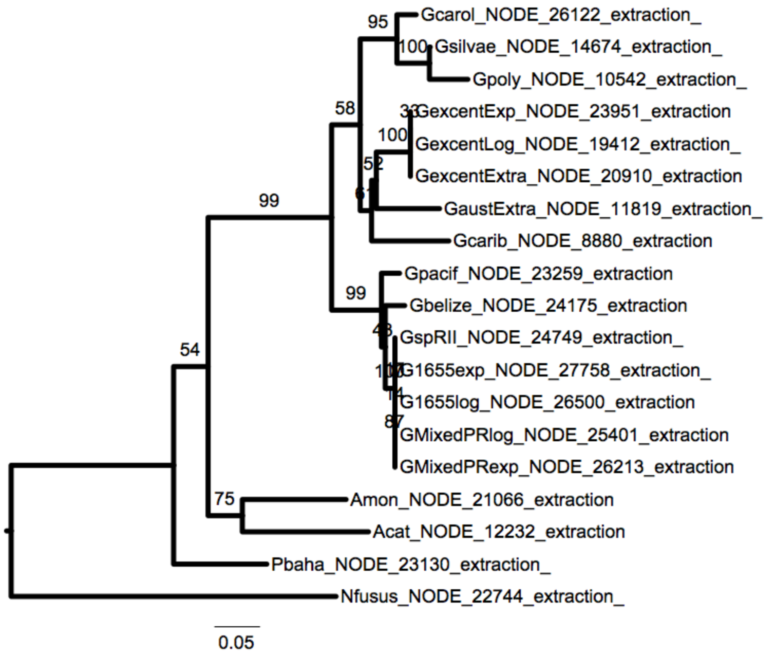

Figure 43. Gene psmc3

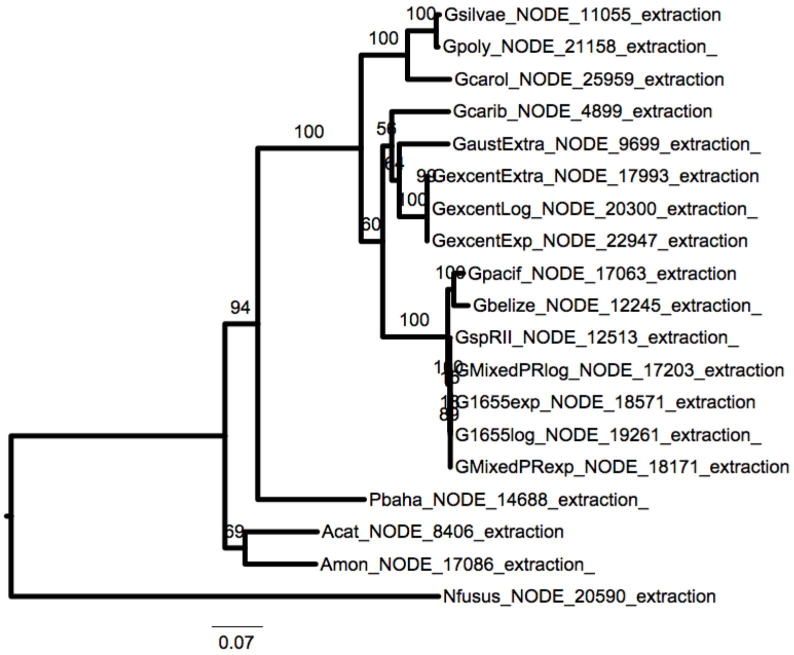

Figure 44. Gene sars

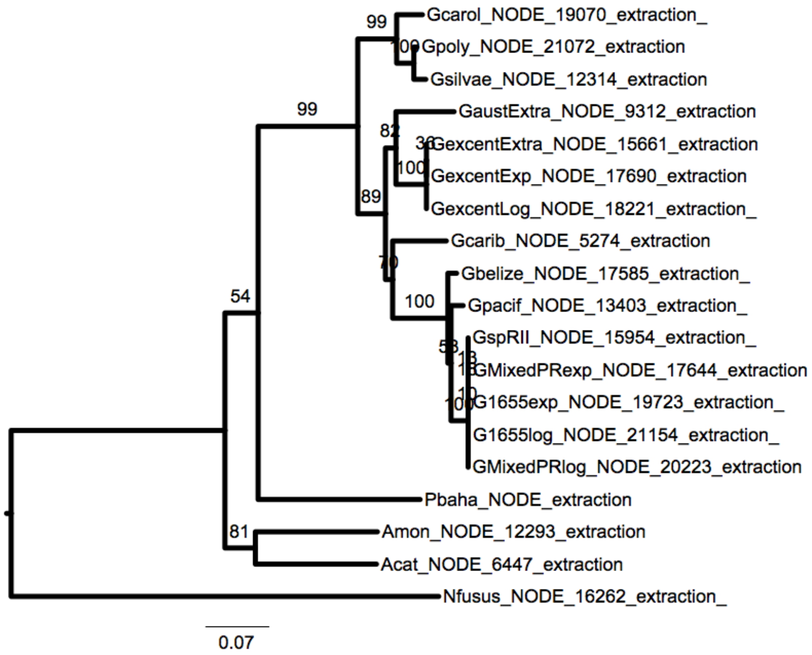

Figure 45. Gene tcp1-epsilon

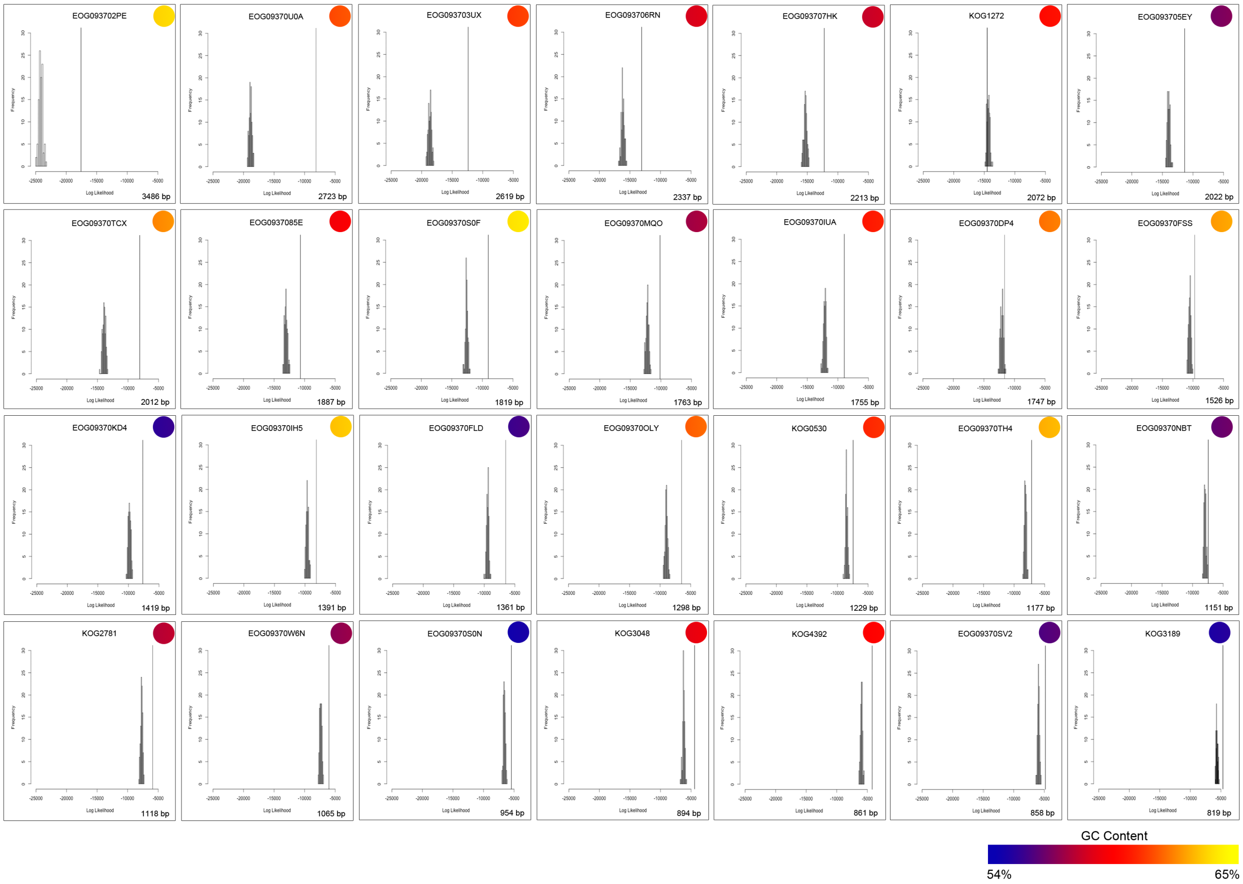

Supplemental Figure (46) for the Gambierdiscus phylogeny paper for describing a new dinoflagellate species. This figure shows the results of parametric bootstrapping for each individual gene used in the multigene phylogenies. Pseudo-replicates were generated for each gene with Seq-Gen using the GTR model. These simulated datasets were run through RAxML to obtain likelihood values; 100 times each. The values were plotted into histograms using R. Additionally, vertical lines were added to show two additional likelihood values: 1) the likelihood of the original gene alignment (with no reference to the final concatenated tree) and 2) the likelihood of the original gene alignment given the final, 28-gene concatenated tree. In the images, it looks like there is only one line shown, because both of the additional likelihood values are nearly identical relative to the histogram. In addition, the histograms are in order of gene length, from largest to smallest (with the actual bp count in the bottom right hand corner), and GC content is shown using a colored dot, with more blue indicating lower GC content and more yellow indicating greater GC content (a gradient is shown in the bottom right hand corner of the whole figure). This has also been posted to figshare: https://doi.org/10.6084/m9.figshare.12636395.v1

Supplementary Table 1. Indices used for generating Illumina libraries. Samples were submitted to the Institute for Genomic Sciences for sequencing.

Species Name	Sheared?	i7	i5
Gambierdiscus pacificus	Yes	N701 (TAAGGCGA)	S517 (GCGTAAGA)
Gambierdiscus sp. Ribotype II	Yes	N702 (CGTACTAG)	S517 (GCGTAAGA)
Gambierdiscus silvae	Yes	N703 (AGGCAGAA)	S517 (GCGTAAGA)
Gambierdiscus ruetzleri	Yes	N704 (TCCTGAGC)	S517 (GCGTAAGA)
Gambierdiscus caribaeus	Yes	N705 (GGACTCCT)	S517 (GCGTAAGA)
Gambierdiscus australes	Yes	N706 (TAGGCATG)	S517 (GCGTAAGA)
Gambierdiscus pacificus	No	N701 (TAAGGCGA)	S502 (CTCTCTAT)
Gambierdiscus sp. Ribotype II	No	N702 (CGTACTAG)	S502 (CTCTCTAT)
Gambierdiscus silvae	No	N703 (AGGCAGAA)	S503 (TATCCTTCT)
Gambierdiscus ruetzleri	No	N704 (TCCTGAGC)	S503 (TATCCTTCT)
Gambierdiscus caribaeus	No	N705 (GGACTCCT)	S504 (AGAGTAGA)
Gambierdiscus australes	No	N706 (TAGGCATG)	S504 (AGAGTAGA)

Supplementary Table 2. Individual Genes Used in This Study.

Gene ID	Gene Function	Source*	Gene Length#
**atp6v1b**	Vacuolar type ATPase B subunit	Jan	1486 bp
**atp6v1d**	Vacuolar type ATPase D subunit	Jan	781 bp
**dimt1l**	Dimethyladenosine transferase	Jan	734 bp
**dnai2**	Dynein intermediate chain 2	Jan	1627 bp
**eif2b**	Eukaryotic translation initiation factor 2 subunit beta	Jan	544 bp
**emg1**	rRNA small subunit methyltransferase NEP1	Jan	700 bp
**EOG09370DP4**	NADPH:adrenodoxin oxidoreductase, mitochondrial	BUSCO	1807 bp
**EOG09370FLD**	26S proteasome regulatory subunit RPN7	BUSCO	1421 bp
**EOG09370FSS**	Glycosyltransferase	BUSCO	1586 bp
**EOG09370IH5**	C21orf19-like protein (MEMO1)	BUSCO	1451 bp
**EOG09370IUA**	GPI-anchor transamidase	BUSCO	1755 bp
**EOG09370KD4**	KRR1 small subunit processome component	BUSCO	1359 bp
**EOG09370MQO**	Phosphopantothenate-cysteine ligase 1	BUSCO	1763 bp
**EOG09370NBT**	Ribosome production factor 2 (brix domain containing)	BUSCO	1151 bp
**EOG09370OLY**	Ribosome maturation protein SBDS	BUSCO	1298 bp
**EOG09370S0F**	Ribosome biogenesis protein TSR3	BUSCO	1819 bp
**EOG09370S0N**	Ran-binding protein	BUSCO	954 bp
**EOG09370SV2**	Vacuolar/vesicle protein sorting 29	BUSCO	858 bp
**EOG09370TCX**	tRNA methyltransferase-like protein 1	BUSCO	2012 bp
**EOG09370TH4**	thymidylate (dTMP) kinase	BUSCO	1177 bp
**EOG09370U0A**	26S proteasome non-ATPase regulatory subunit 4	BUSCO	2723 bp
**EOG09370W6N**	Prefoldin subunit 3	BUSCO	1065 bp
**EOG093702PE**	Nucleolar RNA-associated protein 6	BUSCO	3486 bp
**EOG093703UX**	Glutamine-dependent NAD(+) synthetase	BUSCO	2691 bp
**EOG093705EY**	Phenylalanyl-tRNA synthetase beta subunit	BUSCO	2022 bp
**EOG093706RN**	Conserved oligomeric golgi complex 4	BUSCO	2337 bp
**EOG093707HK**	SDA1 domain-containing protein	BUSCO	2213 bp
**EOG0937085E**	NMD3 ribosome export adaptor	BUSCO	1887 bp
**etf1**	Eukaryotic translation termination factor 1	Jan	1319 bp
**KOG0530**	Farnesyltransferase alpha subunit/ geranylgeranyltransferase type I alpha subunit	CEGMA	1229 bp
**KOG1272**	WD-40 repeat-containing subunit of 18S rRNA processing complex	CEGMA	2072 bp
**KOG2781**	U3 small nucleolar ribonucleoprotein component	CEGMA	1118 bp
**KOG3048**	Molecular chaperone prefoldin, subunit 5	CEGMA	894 bp
**KOG3189**	Phosphomannomutase	CEGMA	819 bp
**KOG4392**	RNA polymerase, subunit L	CEGMA	861 bp
**mcm6**	DNA replication licensing factor MCM6	Jan	2541 bp
**nop56**	Nucleolar protein 56	Jan	1456 bp
**pmsd1**	26S proteasome non-ATPase regulatory subunit 1	Jan	3614 bp
**pmsd12**	26S proteasome non-ATPase regulatory subunit 12	Jan	1396 bp
**psma5**	Proteasome subunit alpha type-5	Jan	636 bp
**psma7**	Proteasome subunit alpha type-7	Jan	761 bp
**psmb1**	Proteasome subunit beta type-1	Jan	676 bp
**psmc3**	26S proteasome regulatory subunit 6A	Jan	1383 bp
**sars**	Serine tRNA ligase, cytoplasmic	Jan	1432 bp
**tcp1-epsilon**	T-complex protein 1 subunit epsilon	Jan	1657 bp

* "Jan": Janouškovec *et al.*, 2017 ; # Alignment length (post-MUSCLE)

Supplementary Table 3. Literature survey of how well rDNA phylogenies distinguish species in different dinoflagellate genera.

The following table provides the results of a comprehensive review of rDNA phylogenies from 232 genera representing over 862 described species from 473 articles. The survey was completed to address how well phylogenies based on a single locus could discriminate dinoflagellate species. Specifically, the goal was to determine how well rDNA phylogenies based on either SSU, ITS/5.8S, or D1-D2 / D1-D3 / D1-D6 LSU rDNA clades distinguished dinoflagellates species/ribotypes (i.e. putative new species). Articles included in this survey were identified using Web of Science, Google searches, and from the reference sections of various taxonomic studies. The following information was recorded: (1) genus, species / ribotype; (2) whether a SSU, ITS/5.8S or D1-D2/D1-D3/D1-D6 LSU rDNA phylogeny exists for each species/ribotype entry and how well the particular rDNA regions found in the phylogenies was able to distinguishing the species/ribotype and (3) a representative listing of the articles containing phylogenies from which the data were extracted for each species or ribotype.

The nomenclatural changes that occurred over time were also reflected in the table with the most current name followed by previous homotypic genus and species names shown in parentheses. These nomenclatural changes were determined using AlgaeBase with the understanding that AlgaeBase is not a definitive taxonomic authority. However, AlgaeBase is relatively up to date and provides the best means of assessing nomenclatural changes over the past 25 years for such a diverse group of dinoflagellates.

Classifying how well species/ribotype(s) were delineated in a given phylogeny was nuanced and dependent on a combination of factors. The first factor was whether single or

multiple sequences were used to represent a given species/ribotype in a particular phylogeny. In cases where the single sequence fell on a well-separated branch, it was taken as tentatively representing a distinct species. Multiple sequences attributed to the same species falling into a distinct clade in the phylogeny was interpreted as definitive evidence that the clade represented a distinct species. The second factor was whether all the sequences falling into a given clade were properly identified given sequence entriesinto GenBank are sometimes assigned to the wrong species. As a result, some distinct clades contain sequences identified as belonging to different species. The question in each case is whether these are simply misidentified sequences belonging to a single species, or whether the rDNA region being examined failed to resolve closely related species. Given these particular factors, the specific species and ribotypes identified in the various individual phylogenies were evaluated and assigned to one of the following five designations:

- "Yes unconditionally" (= Y), cases where distinct, species-specific clades were present in one or more of the phylogenies reviewed were comprised of two or more sequences.
- "Yes provisionally" (= YP), indicated when a single sequence representing a given species fell on a branch distinct from other species.
- Yes, but ambiguous (= YA), designated situations where a distinct clade was observed containing sequences ascribed to multiple species, but likely belonged to the same species, however were misidentified when they were submitted to GenBank.
- No (= N) denotes cases where sequences from different known morphologically distinct species fall into the same clade. In these instances, phylogenies based on the rDNA gene segment fail to delineate what are morphologically distinct species.

- No, but ambiguous (=NA), represents a rarer version of the YA designation where there is some evidence the different sequences in the same clade are actually from distinct species, but this could not be fully resolved given the available data.

Because individual rDNA phylogenetic trees frequently utilized different species-specific sequences, the results were not always consistent as to whether the species / ribotypes should be assigned a Y, YP, YA, N or NA designation. In these cases where they disagreed, multiple designations used to represent the contradictory results among studies.

Genus and Species	SSU	ITS/5.8S	D1-D2 or D1-D3 LSU	Reference(s)
Abedinium (= Leptophyllus)				
dasypus	Y			Gómez et al. (2010b)<br>Cooney et al. (2020)
folium	YP			Cooney et al. (2020)
Achradina				
pulchra	Y			Gómez et al. 2017
Adenoides				
eludens<br>ADE2	Y	YP	Y	Hoppenrath et al. (2012b)<br>Gómez et al. (2015a)<br>You et al. (2015)<br>Mertens et al. (2015b)<br>Hoppenrath et al. (2017)<br>Gu et al. (2018)<br>Efimova et al. (2019)<br>Selina and Efimova (2020)<br>Gottschling et al. (2021a,b)
sinensis<br>sp. NIES-1402	Y		YP	You et al. (2015)<br>Hoppenrath et al. (2017)<br>Gu et al. (2018)
Aduncodinium				
glandula (species requires further investigation)	YP	YP	YP	Kang et al. (2015)<br>Saburova and Chomérat (2018)<br>Žerdoner Čalasan et al. (2019)<br>Gottschling et al. (2021a,b)
Ailadinium				
reticulatum	Y		Y	Saburova and Chomérat (2014)<br>You et al. (2015)<br>Gu et al. (2018)<br>Gómez et al. (2019b)
Akashiwo				
sanguinea (= Gymnodinium sanguineum)<br>CCMP1321 U41085<br>GnSg02<br>NCMA1837	Y	Y	Y	Grzebyk et al. (1998)<br>Daugbjerg et al. (2000)<br>Gast and Caron (2001)<br>Guillou et al. (2002)<br>Hansen and Daugbjerg (2004)<br>Kim, S.H. et al. (2004)<br>Shao et al. (2004)<br>Lindberg et al. (2005)<br>Murray et al. (2005)<br>Dolapsakis et al. (2006)<br>Iwataki et al. (2007)<br>Kim and Kim (2007)<br>Moestrup et al. (2008)<br>Howard et al. (2009)<br>Gottschling et al. (2012)<br>Hoppenrath et al. (2012a,b)

				Qiu et al. (2013)<br>Takano et al. (2014)<br>Gómez et al. (2015a)<br>Mertens et al. (2015b)<br>Reñé et al. (2015)<br>Hoppenrath et al. (2017)<br>Li et al. (2017)<br>Wakeman et al. (2018a)<br>Gottschling et al. (2021a,b)
Alexandrium				
acatenella			YP	Leaw et al. 2005
affine	Y	Y	Y	Zardoya et al. (1995)<br>Adachi et al. (1996)<br>Adachi et al. (1997)<br>Medlin et al. (1998)<br>Guillou et al. (2002)<br>Hansen and Daugbjerg (2004)<br>Kim, S.H. et al. (2004)<br>Kim, K.-Y. et al. (2005b)<br>Lilly et al. (2005)<br>Penna et al. (2005a)<br>Rogers et al. (2006)<br>Moestrup et al. (2008)<br>Penna et al. (2008)<br>Masseret et al. (2009)<br>Jedlicki et al. (2012)<br>Miranda et al. (2012)<br>Gu et al. (2013b)<br>Murray et al. (2014a)<br>Luo et al. (2016b)<br>Kim and Park (2017)<br>Menezes et al. (2018)<br>Lim et al. (2019)<br>Shikata et al. (2020)<br>Gottschling et al. (2021a)<br>Liu, Y. et al. (2021)
andersonii	YP	Y	Y	Medlin et al. (1998)<br>Guillou et al. (2002)<br>Penna et al. (2005a)<br>Dolapsakis et al. (2006)<br>Penna et al. (2008)<br>Amorim et al. (2013)<br>Gu et al. (2013b)<br>Menezes et al. (2018)<br>Efimova et al. (2019)<br>Li et al. (2019)<br>Lim et al. (2019)

australiense	YP	Y	Y	Medlin et al. (1998)<br>Guillou et al. (2002)<br>Lilly et al. (2007)<br>John et al. (2014)<br>Kim and Park (2017)<br>Menezes et al. (2018)<br>Li et al. (2019)<br>Shikata et al. (2020)<br>Liu, Y. et al. (2021)
balechii			YP	Leaw et al. 2005
catenella (= Alexandrium fundyense)	Y	Y	Y	Adachi et al. (1996)<br>Grzebyk et al. (1998)<br>Medlin et al. (1998)<br>Hansen et al. (2000)<br>Guillou et al. (2002)<br>Kim, S.H. et al. (2004)<br>Kim, K.-Y. et al. (2005b)<br>Murray et al. (2005)<br>Penna et al. (2005a)<br>Rogers et al. (2006)<br>Lilly et al. (2007)<br>Penna et al. (2008)<br>Jedlicki et al. (2012)<br>Gu et al. (2013b)<br>John et al. (2014)<br>Saburova and Chomérat (2014)<br>Kim and Park (2017)<br>Menezes et al. (2018)<br>Efimova et al. (2019)<br>Li et al. (2019)<br>Lim et al. (2019)<br>Shikata et al. (2020)<br>Liu, Y. et al. (2021)
cohorticula	Y	YP	Y	Guillou et al. (2002)<br>Masseret et al. (2009)<br>Gu et al. (2013b)<br>John et al. (2014)<br>Lim et al. (2019)<br>Liu, Y. et al. (2021)
compressum			YP	Leaw et al. (2005)
concavum			YP	Leaw et al. (2005)<br>Kim, K.-Y. et al. (2005b)<br>Gómez and Artigas (2019)
diversaporum	Y	Y	Y	Murray et al. (2014)<br>Menezes et al. (2018)<br>Gómez and Artigas (2019)<br>Lim et al. (2019)

foedum			YP	Leaw et al. 2005
fraterculus	Y	Y	Y	Kim, S.H. et al. (2004)<br>Kim, K.-Y. et al. (2005b)<br>Lilly et al. (2005)<br>Penna et al. (2008)<br>Masseret et al. (2009)<br>Miranda et al. (2012)<br>Gu et al. (2013b)<br>Murray et al. (2014)<br>Menezes et al. (2018)<br>Lim et al. (2019)<br>Shikata et al. (2020)<br>Liu, Y. et al. (2021)
hiranoi<br>(V4 rehgion SSU similar to Alexandrium taylori, Alexandrium pseudogoniaulax)	YP	YP	YP	Kim, K.-Y. et al. (2005b)<br>Leaw et al. 2005<br>Murray et al. (2014a)<br>Yamada et al. (2015)<br>Efimova et al. (2019)<br>Liu, Y. et al. (2021)
insuetum	YP	Y	Y	Adachi et al. (1997)<br>Guillou et al. (2002)<br>Lilly et al. (2005)<br>Penna et al. (2008)<br>Hoppenrath et al. (2017)<br>Menezes et al. (2018)<br>Efimova et al. (2019)<br>Gómez and Artigas (2019)<br>Li et al. (2019)<br>Lim et al. (2019)<br>Shikata et al. (2020)
kutnerae			YP	Leaw et al. (2005)
leei	Y	Y	Y	Kim, K.-Y. et al. (2005b)<br>Lilly et al. (2005)<br>John et al. (2014)<br>Gu et al. (2013b)<br>Murray et al. (2014a)<br>Menezes et al. (2018)<br>Lim et al. (2019)<br>Shikata et al. (2020)<br>Liu, Y. et al. (2021)
margalefii	YP	Y	Y	Grzebyk et al. (1998)<br>Guillou et al. (2002)<br>Hansen and Daugbjerg (2004)<br>Kim, K.-Y. et al. (2005b)<br>Lilly et al. (2005)<br>Penna et al. (2005a)<br>Dolapsakis et al. (2006)

				Gribble and Anderson (2006)<br>Rogers et al. (2006)<br>Moestrup et al. (2008)<br>Penna et al. (2008)<br>Howard et al. (2009)<br>Gu et al. (2013b)<br>John et al. (2014)<br>Murray et al. (2014a)<br>Kim and Park (2017)<br>Lim et al. (2019)<br>Shikata et al. (2020)
mediterraneum	Y	Y	Y	Lilly et al. (2007)<br>Penna et al. (2008)<br>John et al. 2014<br>Menezes et al. (2018)<br>Lim et al. (2019)<br>Shikata et al. (2020)<br>Liu, Y. et al. (2021)
minutum (= A. angustitabulatum and A. lusitanicum)	Y	Y	Y	Zardoya et al. (1995)<br>Grzebyk et al. (1998)<br>Medlin et al. (1998)<br>Guillou et al. (2002)<br>Hansen et al. (2003)<br>Lilly et al. (2005)<br>Penna et al. (2005a)<br>Dolapsakis et al. (2006)<br>Rogers et al. (2006)<br>Hoppenrath and Leander (2007)<br>Penna et al. (2008)<br>Gómez and López-García (2010b)<br>Gómez et al. (2010a,b)<br>Jedlicki et al. (2012)<br>Amorim et al. (2013)<br>Saburova and Chomérat (2014)<br>Hoppenrath et al. (2017)<br>Menezes et al. (2018)<br>Efimova et al. (2019)<br>Li et al. (2019)<br>Lim et al. (2019)<br>Shikata et al. (2020)<br>Gottschling et al. (2021a)
minutum like species<br>Daya Bay<br>OYU 792	Y			Liu, Y. et al. (2021)
monilatum	YP	Y	YP	Leaw et al. (2005)<br>Rogers et al. (2006)<br>Gómez et al. (2010a,b)<br>Murray et al. (2014)

				Liu, Y. et al. (2021)
ostenfeldii (= Alexandrium peruvianum)	Y	YP	Y	Grzebyk et al. (1998)<br>Guillou et al. (2002)<br>Lilly et al. (2005)<br>Dolapsakis et al. (2006)<br>Rogers et al. (2006)<br>Penna et al. (2008)<br>Gottschling et al. (2012)<br>Kremp et al. (2014)<br>Saburova and Chomérat (2014)<br>Hoppenrath et al. (2017)<br>Gu et al. (2018)<br>Menezes et al. (2018)<br>Efimova et al. (2019)<br>Li et al. (2019)<br>Lim et al. (2019)<br>Shikata et al. (2020)<br>Liu, Y. et al. (2021)
pacificum	Y	Y	Y	Adachi et al. (1996)<br>Medlin et al. (1998)<br>Hansen et al. (2000)<br>Murray et al. (2005)<br>Lilly et al. (2007)<br>Penna et al. (2008)<br>John et al. (2014)<br>Kim and Park (2017)<br>Menezes et al. (2018)<br>Li et al. (2019)<br>Lim et al. (2019)<br>Shikata et al. (2020)<br>Gottschling et al. (2021a)<br>Liu, Y. et al. (2021)
panamense	YP			Gu et al. (2018)
pohangense	YP		YP	Gómez and Artigas (2019)<br>Lim et al. (2019)
pseudogonyaulax	YP	Y	Y	Adachi et al. (1997)<br>Hansen and Daugbjerg (2004)<br>Kim, K.-Y. et al. (2005b)<br>Lilly et al. (2005)<br>Penna et al. (2005a)<br>Gribble and Anderson (2006)<br>Penna et al. (2008)<br>Howard et al. (2009)<br>Masseret et al. (2009)<br>John et al. 2014<br>Murray et al. (2014a)<br>Luo et al. (2016b)

				Efimova et al. (2019)<br>Gómez and Artigas (2019)<br>Li et al. (2019)<br>Lim et al. (2019)<br>Shikata et al. (2020)<br>Liu, Y. et al. (2021)
satoanum	YP	YP	YP	Leaw et al. (2005)<br>Lilly et al. (2005)<br>Murray et al. (2014a)<br>Kim, K.-Y. et al. (2005b)<br>Gómez and Artigas (2019)<br>Shikata et al. (2020)<br>Liu, Y. et al. (2021)
sp.<br>Ach01			Y	Jedlicki et al. (2012)
sp.<br>UFRJ-MN01<br>UFRJ-MN01		Y	Y	Menezes et al. (2018)
sp.<br>OTU 477	YP			Liu, Y. et al. (2021)
sp 1<br>HG222 LC056069	YP			Yamada et al. (2015)
sp 2<br>NY008 LC056068	YP			Yamada et al. (2015)
tamarense	YP	Y	Y	McNally et al. (1994)<br>Adachi et al. (1996)<br>Adachi et al. (1997)<br>Grzebyk et al. (1998)<br>Medlin et al. (1998)<br>Hansen et al. (2000)<br>Guillou et al. (2002)<br>Kim, S.H. et al. (2004)<br>Penna et al. (2005a)<br>Dolapsakis et al. (2006)<br>Kawami et al. (2006)<br>Rogers et al. (2006)<br>Litaker et al. (2007)<br>Gómez et al. (2010a,b)<br>Jedlicki et al. (2012)<br>Gu et al. (2013b)<br>John et al. (2014)<br>Kim and Park (2017)<br>Menezes et al. (2018)<br>Wakeman et al. (2018a)<br>Efimova et al. (2019)<br>Shikata et al. (2020)<br>Liu, Y. et al. (2021)

tamiyavanichii	Y	Y	Y	Lilly et al. (2005)<br>Kim, K.-Y. et al. (2005b)<br>Rogers et al. (2006)<br>Penna et al. (2008)<br>Jedlicki et al. (2012)<br>Miranda et al. (2012)<br>Gu et al. (2013b)<br>John et al. (2014)<br>Menezes et al. (2018)<br>Efimova et al. (2019)<br>Lim et al. (2019)<br>Shikata et al. (2020)
tamutum	Y	Y	Y	Kim, K.-Y. et al. (2005b)<br>Lilly et al. (2005)<br>Rogers et al. (2006)<br>Penna et al. (2008)<br>Jedlicki et al. (2012)<br>Miranda et al. (2012)<br>Menezes et al. (2018)<br>Gómez and Artigas (2019)<br>Lim et al. (2019)<br>Shikata et al. (2020)
taylorii	Y	Y	Y	Kim, K.-Y. et al. (2005b)<br>Lilly et al. (2005)<br>Penna et al. (2005a)<br>Rogers et al. (2006)<br>Masseret et al. (2009)<br>Anderson et al. (2012)<br>Miranda et al. (2012)<br>John et al. (2014)<br>Murray et al. (2014a)<br>Shikata et al. (2020)<br>Liu, Y. et al. (2021)
tropicale	YP	Y	Y	Adachi et al. (1996)<br>Anderson et al. (2012)<br>Murray et al. (2014)<br>Efimova et al. (2019<br>Shikata et al. (2020)
Amphidinium				
asymmetricum	YP			Gómez et al. (2009a)<br>Lee, K.H. et al. (2013)
belauense	YP/NA			McNally et al. (1994)<br>Grzebyk et al. (1998)<br>Gómez et al. (2009a)<br>Lee, K.H. et al. (2013)
carterae (= Amphidinium klebsii)	Y/YA	Y	Y	McNally et al. (1994)<br>Hansen and Daugbjerg (2004)

				Jørgensen et al. (2004a)<br>Murray et al. (2004)<br>Shao et al. (2004)<br>Murray et al. (2005)<br>Iwataki et al. (2007)<br>Litaker et al. (2007)<br>Kim and Kim (2007)<br>Moestrup et al. (2008)<br>Gómez et al. (2009a)<br>Tamura et al. (2009)<br>Hansen and Daugbjerg (2011)<br>Hoppenrath et al. (2012a)<br>Lee, K.H. et al. (2013)<br>Qiu et al. (2013)<br>Reñé et al. (2013)<br>Luo et al. (2016b)<br>Karafas et al. (2017)<br>Wakeman et al. (2018a)<br>Efimova et al. (2019)<br>Gottschling et al. (2021a,b)
cf. thermaeum		YP	YP	Karafas et al. (2017)
corpulentum	NA/YP			Gómez et al. (2009a)<br>Lee, K.H. et al. (2013)<br>Reñé et al. (2015)
crassum			YP	Reñé et al. (2015)
cupulatisquama	N/YP		YP	Tamura et al. (2009)<br>Lee, K.H. et al. (2013)<br>Yamada et al. (2015)<br>Moreira-González et al. (2019)
eilatiensis		Y		Shao et al. (2004)<br>Litaker et al. (2007)
fijiense		Y	Y	Karafas et al. (2017)<br>Moreira-González et al. (2019)
gibbosum<br>NY004 AB863027<br>L13719	Y		Y	Hansen and Daugbjerg (2004)<br>Jørgensen et al. (2004a)<br>Dolapsakis and Economou-Amilli (2009)<br>Tamura et al. (2009)<br>Hoppenrath et al. (2012a)<br>Murray et al. (2012)<br>Lee, K.H. et al. (2013)<br>Yamada et al. (2015)<br>Karafas et al. (2017)<br>Wakeman et al. (2018a)<br>Moreira-González et al. (2019)
globosum		Y	Y	Murray et al. (2004)<br>Karafas et al. (2017)

herdmanii	YA		Y	Hansen and Daugbjerg (2004)<br>Murray et al. (2004)<br>Murray et al. (2005)<br>Cyronak and Tomas (2008)<br>Hansen and Daugbjerg (2011)<br>Lee, K.H. et al. (2013)<br>Reñé et al. (2015)<br>Karafas et al. (2017)<br>Potvin et al. (2018)<br>Moreira-González et al. (2019)
incoloratum	YP		YP	Jørgensen et al. (2004a)<br>Murray et al. (2004)<br>Murray et al. (2005)<br>Kim and Kim (2007)<br>Dolapsakis and Economou-Amilli (2009)<br>Tamura et al. (2009)<br>Lee, K.H. et al. (2013)<br>Takano et al. (2014)<br>Karafas et al. (2017)<br>Moreira-González et al. (2019)
klebsii	NA		YP	Lee, K.H. et al. (2013)<br>Reñé et al. (2013)<br>Reñé et al. (2015)
longum	YP			Sparmann et al. (2008)<br>Lee, K.H. et al. (2013)
magnum		Y	Y	Karafas et al. (2017)<br>Moreira-González et al. (2019)
massartii<br>AMJJ1<br>CCCM 439 AF274255	Y	Y	Y	Jørgensen et al. (2004a)<br>Murray et al. (2004)<br>Leaw et al. (2005)<br>Moestrup et al. (2008)<br>Tamura et al. (2009)<br>Hansen and Daugbjerg (2011)<br>Lee, K.H. et al. (2013)<br>Takano et al. (2014)<br>Yamada et al. (2015)<br>Luo et al. (2016b)<br>Karafas et al. (2017)<br>Wakeman et al. (2018a)<br>Moreira-González et al. (2019)<br>Gottschling et al. (2021b)
cf. massartii		Y	Y	Karafas et al. (2017)<br>Moreira-González et al. (2019)
mootonorum	YP/NA		YA	Jørgensen et al. (2004a)<br>Murray et al. (2004)<br>Tamura et al. (2009)

				Lee, K.H. et al. (2013)<br>Moreira-González et al. (2019)
operculatum<br>TAK-0 AB704006	NP/Y		Y	Jørgensen et al. (2004a)<br>Murray et al. (2004)<br>Cyronak, T., Tomas, C. (2008)<br>Tamura et al. (2009)<br>Hoppenrath et al. (2012a)<br>Horiguchi et al. (2012)<br>Lee, K.H. et al. (2013)<br>Takano et al. (2014)<br>Gómez et al. (2015a)<br>Yamada et al. (2015)<br>Karafas et al. (2017)<br>Gu et al. (2018)<br>Efimova et al. (2019)<br>Moreira-González et al. (2019)
paucianulatum		Y	Y	Karafas et al. (2017)<br>Moreira-González et al. (2019)
pseudomassartii		YP	Y	Karafas et al. (2017)<br>Moreira-González et al. (2019)
rhynchocephalum / cf. rhynchocephalum<br>UTEX LB 1946 AY443012	YP			Yamada et al. (2015)
sp.<br>FA1-CMSTAC022			YP	Lee, K.H. et al. (2013)
sp.<br>FC2-CMSTAC023			YP	Lee, K.H. et al. (2013)
sp.<br>HG113 LC054922	YP			Yamada et al. (2015)
sp.<br>HG115 AB477347<br>(= sp. S1-CMSTAC025)	Y		YA	Lee, K.H. et al. (2013)<br>Yamada et al. (2015)
steinii<br>HG214 LC054920<br>HG220 LC054921	Y		Y	Jørgensen et al. (2004a)<br>Murray et al. (2004)<br>Murray et al. (2005)<br>Kim and Kim (2007)<br>Cyronak, T., Tomas, C. (2008)<br>Dolapsakis and Economou-Amilli (2009)<br>Tamura et al. (2009)<br>Hoppenrath et al. (2012a,b)<br>Lee, K.H. et al. (2013)<br>Reñé et al. (2015)<br>Karafas et al. (2017)<br>Wakeman et al. (2018a)<br>Efimova et al. (2019)<br>Moreira-González et al. (2019)

theodorei		YP	Y	Karafas et al. (2017)<br>Moreira-González et al. (2019)
thermaeum	Y	Y	Y	Dolapsakis and Economou-Amilli (2009)<br>Lee, K.H. et al. (2013)<br>Karafas et al. (2017)<br>Moreira-González et al. (2019)
tomasii		Y	YP	Karafas et al. (2017)<br>Moreira-González et al. (2019)
trulla	Y		Y	Jørgensen et al. (2004a)<br>Murray et al. (2004)<br>Leaw et al. (2005)<br>Iwataki et al. (2007)<br>Dolapsakis and Economou-Amilli (2009)<br>Tamura et al. (2009)<br>Lee, K.H. et al. (2013)<br>Karafas et al. (2017)<br>Moreira-González et al. (2019)
Amoebophrya				
sp. 1<br>ex Cochlodinium polykrikodies	YP			Kim and Park (2014)
sp. 2<br>ex Cochlodinium polykrikodies	YP			Kim and Park (2014)
sp.<br>'GS'	YP			Janson et al. (2000)
sp.<br>'DN'	YP			Janson et al. (2000)
sp. ex Akashiwo sanguinea (= Gymnodinium sanguinea)<br>AF069516<br>ex. Akashiwo sanguineaum<br>HM483395	YP	YP	YP	Gunderson et al. (1999)<br>Gunderson et al. (2002)<br>Skovgaard et al. (2005)<br>Harada et al. (2007)<br>Kim et al. (2008)<br>Jung et al. (2015)<br>Kim and Park (2017)<br>Wakeman et al. (2018b)<br>Gottschling et al. (2021a,b)
sp.<br>ex Alexandrium affine sp.<br>ex Levanderina fissa<br>ex Alexandrium affine<br>AY775284<br>ex Gymnodinium instriatum<br>AF472554<br>ex Gymnodinium instriatum<br>HM483394	Y	YP	YP	Kim et al. (2008)<br>Miranda et al. (2012)<br>Jung et al. (2015)<br>Gottschling et al. (2021a,b)
sp.	YP			Kim et al. (2008)

ex Ceratium lineatum AY260467				
sp.<br>ex Ceratium tripos AY208892<br>ex Prorocentrium micans AY208893	YP			Skovgaard et al. (2005)<br>Harada et al. (2007)<br>Kim et al. (2008)<br>Jung et al. (2015)<br>Horiguchi et al. (2017)
sp.<br>ex Ceratium tripos AY260468	YP			Kim et al. (2008)
sp.<br>ex Dinophysis norvegica AF239260	YP			Salomon et al. (2003)<br>Harada et al. (2007)<br>Kim et al. (2008)<br>Jung et al. (2015)
sp.<br>ex Dinophysis norvegica AY260469	YP			Gunderson et al. (2002)<br>Salomon et al. (2003)<br>Kim et al. (2008)
sp.<br>ex Gymnodinium instriatum AF472554	YP		YP	Gunderson et al. (2002)<br>Harada et al. (2007)<br>Gómez et al. (2010b)<br>Hoppenrath et al. (2012b)<br>Gómez et al. (2015a)<br>Horiguchi et al. (2017)<br>Kim and Park (2017)<br>Wakeman et al. (2018a)
sp.<br>ex Gonyaulax polygramma AY775285	YP			Kim et al. (2008)<br>Miranda et al. (2012)<br>Jung et al. (2015)<br>Reñé et al. (2019)
sp.<br>ex Karlodinium veneficum (=K. micrum) AF472553	YP			Gunderson et al. (2002)<br>Harada et al. (2007)<br>Kim et al. (2008)<br>Hoppenrath et al. (2012b)<br>Jung et al. (2015)<br>You et al. (2015)<br>Wakeman et al. (2018a)<br>Gómez et al. (2015a)
sp.<br>ex Prorocentrum minimum AY208894	YP			Harada et al. (2007)<br>Kim et al. (2008)<br>Jung et al. (2015)
sp.<br>ex Scripsiella sp. AF472555	YP			Gunderson et al. (2002)<br>Skovgaard et al. (2005)<br>Kim et al. (2008)<br>Skovgaard and Salomonsen (2009)<br>Gómez et al. (2010b)<br>Miranda et al. (2012)<br>Jung et al. (2015)

sp. Ribotype 1	N	Y		Cai et al. (2020)
sp. Ribotype 2	N	Y		Cai et al. (2020)
sp. Ribotype 3	N	Y		Cai et al. (2020)
sp. Ribotype 4	N	Y		Cai et al. (2020)
sp. Ribotype 5	N	Y		Cai et al. (2020)
sp. Ribotype 6	N	Y		Cai et al. (2020)
sp. Ribotype 7	N	Y		Cai et al. (2020)
sp. Ribotype 8	N	Y		Cai et al. (2020)
sp.	YP			Gómez et al. (2010a)
sp.<br>OLI11261 AJ402338	YP			Harada et al. (2007)
sp.<br>DH148-EKD27 AF290077	YP			Harada et al. (2007)
sp.<br>OLI11115 AJ402326	YP			Harada et al. (2007)
Amphidiniella				
sedentaria<br>HG156 LC057317<br>HG156 LC057317<br>AB212091	Y			Hoppenrath et al. (2007)<br>Saburova and Chomérat (2014)<br>Gómez et al. (2015a)<br>Yamada et al. (2015)<br>Wakeman et al. (2018a)
Amphidiniopsis				
arenaria			Y	Yamaguchi et al. (2016)
cf. arenaria<br>AY-2016	YP	YP		Gómez et al. (2019b)<br>Reñé et al. (2019)<br>Gottschling et al. (2021b)
bulla<br>AR44 MK940550			YP	Reñé et al. (2019)
dragescoi (= Thecadinium dragescoi)	YP			Hoppenrath et al. (2004)<br>Hoppenrath and Leander (2007)<br>Hoppenrath et al. (2012b)<br>Mertens et al. (2013)<br>Potvin et al. (2013)<br>Mertens et al. (2015b)<br>Gómez (2016b)<br>Hoppenrath et al. (2017)<br>Gómez et al. (2019b)<br>Reñé and Hoppenrath (2019)<br>Selina et al. (2019)
elongata<br>LC191239			YP	Reñé et al. (2019)
erinacea (= morphotype 1)<br>AR41 MK940551<br>AR48 MK940554<br>AR19 MK940556				Reñé et al. (2019)
hexagona (= morphotype 2)	Y			Gómez et al. (2019b)<br>Reñé et al. (2019)

hirsuta	Y			Gomez et al. (2011b)<br>Reñé et al. (2019)
cf. kofoidii<br>BB3	YP			Yamaguchi et al. (2016)<br>Gómez et al. (2019b)
korewalensis<br>kor 1 LC191242	Y		Y	Yamaguchi et al. (2016)<br>Saburova and Chomérat (2018)<br>Efimova et al. (2019)<br>Gómez et al. (2019b)<br>Reñé et al. (2019)<br>Selina et al. (2019)<br>Gottschling et al. (2021b)
A. bulla + A. elongate + A. korewalensis + A. uroensis (= morphopytpe 3. The relationship between these putative species requires futher investigation)	Y		YP	Saburova and Chomérat (2018)<br>Efimova et al. (2019)<br>Reñé et al. (2019)<br>Gottschling et al. (2021b)
rotundata (=morphotype 4)<br>rot1 LC191247	Y		YP	Hoppenrath et al. (2012b)<br>Saburova and Chomérat (2014)<br>SMertens et al. (2015b)<br>Gómez (2016b)<br>Yamaguchi et al. (2016)<br>Li et al. (2017)<br>Gómez et al. (2019b)<br>Reñé et al. (2019)<br>Selina et al. (2019)<br>Gottschling et al. (2021b)
sp.<br>AR19 MK940556			YP	Reñé et al. (2019)
swedmarkii	YP			Gomez et al. (2011b)<br>Reñé et al. (2019)
uroensis<br>uro4 LC191233	Y	YP	Y	Yamaguchi et al. (2016)<br>Reñé et al. (2019)<br>Gottschling et al. (2021a,b)
Amphidoma				
languida	Y	Y	Y	Nézan et al. (2012)<br>Tillmann et al. (2012)<br>Tillmann et al. (2014)<br>You et al. (2015)<br>Anglès et al. (2017)<br>Hoppenrath et al. (2017)<br>Gu et al. (2018)<br>Luo et al. (2018a)<br>Selina and Efimova (2020)<br>Tillman et al. (2020)<br>Gottschling et al. (2021a,b)<br>Takahashi et al. (2021)

parvula	YP	YP	YP	Selina and Efimova (2020)<br>Tillmann et al. (2020)<br>Takahashi et al. (2021)
Amphisolenia				
bidentata<br>LE392	YP	YP	Y	Jensen and Daugbjerg (2009)<br>Gómez et al. (2011a)<br>Park et al (2011)<br>Li et al. (2017)<br>Gottschling et al. (2021a,b)
clavipes<br>MR14.6 AB473708<br>May be misidentified A. schauinslandii seqeuence			YA	Park et al (2011)
globifera	YP			Gómez et al. (2011a)<br>Gómez et al. (2015b)
palaeotheroides			YP	Park et al (2011)
schauinslandii	YP			Gómez et al. (2011a)<br>Park et al (2011)
sp.<br>FG281	YP			Gómez et al. (2011a)
thrinax			Y	Jensen and Daugbjerg (2009)<br>Park et al (2011)
Amylax				
triacantha (= Amylax triacantha var. buxus)	Y		Y	Kim and Kim (2007)<br>Mertens et al. (2017a)<br>Efimova et al. (2019)<br>Selina and Efimova (2020)<br>Zhang et al. (2020)
Amyloodinium				
ocellatum	Y	Y	YP	Litaker et al. (2007)<br>Coats et al. (2010)<br>Jung et al. (2015)<br>Gómez and Gast (2018)<br>Kretschmann et al. (2018b)<br>Gottschling et al. (2021a,b)
Ankistrodinium				
armigerum	Y		Y	Watamabe et al. (2014)
semilunatum (= Amphidinium semilunatum, Thecadinium semilunatum)	Y	YP	Y	Murray et al. (2005)<br>Gribble and Anderson (2006)<br>Sparmann et al. (2008)<br>Dolapsakis and Economou-Amilli (2009)<br>Hoppenrath et al. (2012a)<br>Miranda et al. (2012)<br>Yamada et al. (2013)<br>Watamabe et al. (2014)<br>Gómez et al. (2015b)

				Gómez et al. (2016b)<br>Li et al. (2017)<br>Gottschling et al. (2021a)
Ansanella				
granifera<br>AGSW10	Y	YP	Y	Gottschling et al. (2005b)<br>Jeong et al. (2014a)<br>Takahashi et al. (2015)<br>Luo et al. (2016b)<br>Jang et al. (2017a,b)<br>LaJeunesse et al. (2018)<br>Gottschling et al. (2021a,b)
natalensis (= Biecheleria natalensis)	Y			Yamada et al. (2015)
Apicoporus				
glaber	Y	YP	YP	Sparmann et al. (2008)<br>Gómez et al. (2011a)<br>Hoppenrath et al. (2012a)<br>Okamoto et al. (2012)<br>Reñé et al. (2015)<br>Gómez et al. (2016b)<br>Yuasa et al. (2016)<br>Gottschling et al. (2021a)
parvidiaboli	Y			Sparmann et al. (2008)<br>Gómez et al. (2011a)<br>Reñé et al. (2015)<br>Hoppenrath et al. (2017)
sp.	Y		Y	Reñé et al. (2015)
Apocalathium (likely species complex)				
aciculiferum (= Peridinium aciculiferum) Likely that A. aciculiferum and A. malmogiense sequences were miss identified causing sequence overlap. I.E. the ITS1, ITS2, 5.8S, SSU, and partial LSU were the same as S. hangoei<br>AY970653<br>PAER1<br>PAER2<br>PAER3	N	N/YA	N	Gottschling et al. (2005a)<br>Logares et al. (2007)<br>Gu et al. (2013a)<br>Annenkova et al. (2015)<br>Kang et al. (2015)<br>Luo et al. (2015)<br>Craveiro et al. (2016)<br>Luo, Z. et al. (2016a)<br>Saburova and Chomérat (2018)<br>Lee, S.Y. et al. (2019)<br>Luo et al. (2019)<br>Žerdoner Čalasan et al. (2019)<br>Li, Z. et al. (2020)<br>Luo, Z. et al. (2020)<br>Gottschling et al. (2021b)
baicalense (= Peridinium baicalense)	N	Y	Y	Annenkova et al. (2011)<br>Annenkova et al. (2015)<br>Luo et al. (2015)<br>Craveiro et al. (2016)

				Saburova and Chomérat (2018)<br>Luo et al. (2019)<br>Luo, Z. et al. (2020)
euryceps (= Peridinium euryceps)	N	Y	Y	Annenkova et al. (2015)<br>Luo et al. (2015)<br>Craveiro et al. (2016)
malmogiense (= Peridinium malmogiense) (not clear if separate species from Apocalathium aciculiferum)<br>SHTV1<br>SHTV2<br>SHTV5	YA	YP	YA	Craveiro et al. (2016)<br>Hansen et al. (2018)<br>Saburova and Chomérat (2018)<br>Lee, S.Y. et al. (2019)<br>Luo et al. (2019)<br>Žerdoner Čalasan et al. (2019)<br>Li, Z. et al. (2020)<br>Luo, Z. et al. (2020)<br>Tillman et al. (2020)<br>Gottschling et al. (2021a,b)
Archaeperidinium				
bailongense	YP		YP	Liu et al. (2015a,b)<br>Gu et al. (2016)<br>Potvin et al. (2018)
constrictum<br>Yellow Sea KM042419			YA	Liu et al. (2015a,b)<br>Gu et al. (2016)
minutum (= Protoperidinium minutum -<br>in some of the phylogenies a single sequence for A. constrictum and A. saanichi fall into this group, one of which is likely misidentified).<br>Isolate 1 AB564308<br>Isolate 1 AB564310<br>Mondego Estuary GQ227502	Y/YP	Y	Y/YP	Ribeiro, et al. (2010)<br>Yamaguchi et al. (2011)<br>Hoppenrath et al. (2012b)<br>Mertens et al. (2012)<br>Nézan et al. (2012)<br>Gottschling and McLean (2013)<br>Mertens et al. (2013)<br>Potvin et al. (2013)<br>Chomérat and Bilien (2014)<br>Saburova and Chomérat (2014)<br>Kang et al. (2015)<br>Liu et al. (2015a,b)<br>Yamada et al. (2015)<br>Gómez (2016b)<br>Anglès et al. (2017)<br>Gómez et al. (2017a)<br>Potvin et al. (2018)<br>Reñé et al. (2019)<br>Žerdoner Čalasan et al. (2019)<br>Gottschling et al. (2021a,b)
saanichi<br>AY theca4	N/Y		YA	Mertens et al. (2012)<br>Mertens et al. (2013)<br>Liu et al. (2015a,b)<br>Mertens et al. (2015b)<br>Gu et al. (2016)

				Hoppenrath et al. (2017)<br>Gottschling et al. (2021b)
Asterodinium				
gracile			YP	Benico et al. (2019)
Asulcocephalium				
miricentonis<br>mi11-8kt	Y	YP	Y	Takahashi et al. (2015)<br>Hehenberger et al. (2017)<br>Jang et al. (2017a,b)<br>Gottschling et al. (2021a,b)
Ataxiodinium				
choane	YP		YP	Mertens et al. (2017a)<br>Zhang et al. (2020)
Azadinium				
caudatum	YP	Y	Y	Saburova and Chomérat (2014)<br>Boutrup et al. (2016)<br>Gómez (2016b)<br>Gómez et al. (2017a)<br>Hoppenrath et al. (2017)<br>Gottschling et al. (2021a)<br>Takahashi et al. (2021)
var caudatum / var margalefii	Y	Y	Y	Nézan et al. (2012)<br>Tillmann et al. (2012)<br>Gu et al. (2013a)<br>Luo, Z. et al. (2013)<br>Percopo et al. (2013)<br>Baytut et al. (2016)<br>Kim, H.-J. et al. (2017)<br>Luo, Z. et al. (2017b)<br>Tillman et al. (2020)<br>Gottschling et al. (2021b)
concinnum	YP	YP	YP	Tillmann et al. (2014)<br>Baytut et al. (2016)<br>Efimova et al. (2019)<br>Selina and Efimova (2020)<br>Tillman et al. (2020)<br>Takahashi et al. (2021)<br>Gottschling et al. (2021a,b)
cuneatum	Y	Y	Y	Tillmann et al. (2014)<br>Baytut et al. (2016)<br>Kim, H.-J. et al. (2017)<br>Luo et al. (2017b)<br>Tillman et al. (2020)<br>Takahashi et al. (2021)
dalianense Ribotype A<br>121F6<br>962B8<br>AZCH02 KF543359		Y	Y	Luo, Z. et al. (2013)<br>Tillmann et al. (2014<br>Baytut et al. (2016)<br>Kim, H.-J. et al. (2017)

N-38-03<br>Takahashi et al. (2021) showed less distinct ITS clades so ribotypes A and B may be same species				Luo, Z. et al. (2017b)<br>Tillmann et al. (2017)<br>Tillman et al. (2020)<br>Takahashi et al. (2021)
dalianense Ribotype B<br>481F8<br>962B3<br>H-2-G7<br>IFR-ADA-01C<br>LF-14-F07<br>N12-12-04		Y	Y	Kim, H.-J. et al. (2017)<br>Luo, Z. et al. (2017b)<br>Tillman et al. (2020)<br>Takahashi et al. (2021)
dexteroporum	Y	Y	Y	Luo, Z. et al. (2013)<br>Percopo et al. (2013)<br>Tillmann et al. (2014)<br>Gómez et al. (2017a)<br>Hoppenrath et al. (2017)<br>Kim, H.-J. et al. (2017)<br>Luo, Z. et al. (2017b)<br>Tillmann et al. (2019)<br>Tillmann et al. (2020)<br>Gottschling et al. (2021b)<br>Takahashi et al. (2021)
obesum	Y	Y	Y	Tillmann et al. (2010)<br>Tillmann et al. (2011)<br>Gottschling et al. (2012)<br>Nézan et al. (2012)<br>Tillmann et al. (2012)<br>Percopo et al. (2013)<br>Tillmann et al. (2014)<br>Baytut et al. (2016)<br>Kim, H.-J. et al. (2017)<br>Luo, Z. et al. (2017b)<br>Tillmann et al. (2019)<br>Selina and Efimova (2020)<br>Tillmann et al. (2020)<br>Takahashi et al. (2021)
perforatum	Y	Y	Y	Tillmann et al. (2019)<br>Tillmann et al. (2020)<br>Takahashi et al. (2021)
polongum	Y	Y	Y	Tillmann et al. (2012)<br>Gu et al. (2013a)<br>Luo, Z. et al. (2013)<br>Percopo et al. (2013)<br>Anglès et al. (2017)<br>Kim, H.-J. et al. (2017)<br>Luo, Z. et al. (2017b)

				Selina and Efimova (2020)<br>Tillman et al. (2020)<br>Takahashi et al. (2021)
poporum Ribotypes A1 and A2<br>1-C11<br>1-D5<br>2-B9<br>968B7<br>Brest Bay, France<br>CAWD230 KR534888<br>Kerros, France<br>LF-14-E12<br>N-39-13<br>TIO256<br>TIO420<br>TIO431<br>UTHC5 HQ324893<br>UTHC8 HQ324894<br>UTHD4 HQ324895	Y	Y	Y	Percopo et al. (2013)<br>Luo, Z. et al. (2013)<br>Tillman et al. (2014)<br>Smith et al. (2016)<br>Kim, H.-J. et al. (2017)<br>Luo, Z. et al. (2017b)<br>Tillmann et al. (2017)<br>Li, Z. et al. (2020)<br>Tillman et al. (2020)<br>Gottschling et al. (2021a,b)<br>Takahashi et al. (2021)
poporum Ribotype B<br>AZDH04<br>AZDH05<br>AZDH11<br>AZDH16<br>AZDH23<br>AZFC07<br>AZFC12<br>AZFC15<br>AZFC16<br>AZFC18<br>G14 KC286551<br>G25 KC286552<br>G57 KC286554<br>G60 KC286553<br>G62<br>G66 KC286560<br>G68<br>GM29<br>HJ-2011 FR877580<br>TIO429		Y	Y	Luo, Z. et al. (2013<br>Percopo et al. (2013)<br>Tillman et al. (2014)<br>Kim, H.-J. et al. (2017)<br>Luo, Z. et al. (2017b)<br>Tillmann et al. (2017)<br>Tillman et al. (2020)<br>Takahashi et al. (2021)
poporum Ribotype C1 and C2<br>18A1 KT383009<br>18B2 KT383010<br>18B4 KT383011<br>22C1 KT383017<br>18C3 KT383012<br>18C4 KT383013<br>18C5 KT383014		Y	Y	Luo, Z. et al. (2013)<br>Tillman et al. (2014)<br>Baytut et al. (2016)<br>Kim, H.-J. et al. (2017)<br>Luo, Z. et al. (2017b)<br>Tillmann et al. (2017)<br>Tillman et al. (2020)<br>Takahashi et al. (2021)

18D2 KT383015<br>18D4 KT383016<br>26B5 KT383018<br>AZFC13<br>AZFC14<br>AZFC16 KC286556<br>G37 KC286557<br>G42 KC286558<br>G58 KC286555<br>G59 KC286559<br>G64				
poporum Ribotype D<br>GM29		YP	YP	Luo, Z. et al. (2013)<br>Kim, H.-J. et al. (2017)<br>Tillmann et al. (2017)
poporum<br>(includes ITS ribotypes A1, A2, B, C1 and C2)	Y	Y	Y	Tillmann et al. (2011)<br>Nézan et al. (2012)<br>Gu et al. (2013a)<br>Tillmann et al. (2014)<br>You et al. (2015)<br>Hoppenrath et al. (2017)<br>Gu et al. (2018)<br>Tillman et al. (2020)<br>Takahashi et al. (2021)
spinosum	Y	Y	Y	Tillmann et al. (2009)<br>Tillmann et al. (2010)<br>Tillmann et al. (2011)<br>Nézan et al. (2012)<br>Tillmann et al. (2012)<br>Gu et al. (2013a)<br>Luo, Z. et al. (2013)<br>Percopo et al. (2013)<br>Tillmann et al. (2014)<br>You et al. (2015)<br>Gómez (2016a,b)<br>Smith et al. (2016)<br>Gómez et al. (2017a)<br>Kim, H.-J. et al. (2017)<br>Luo, Z. et al. (2017b)<br>Gu et al. (2018)<br>Tillman et al. (2020)<br>Selina and Efimova (2020)<br>Tillmann et al. (2020)<br>Takahashi et al. (2021)<br>Gottschling et al. (2021a,b)
trinitatum	Y	Y	Y	Tillmann et al. (2014)<br>Baytut et al. (2016)<br>Gómez et al. (2016a)<br>Gómez et al. (2017a)

				Kim, H.-J. et al. (2017)<br>Efimova et al. (2019)<br>Tillman et al. (2020)<br>Gottschling et al. (2021b)<br>Takahashi et al. (2021)
zhuanum	Y	Y	Y	Kim, H.-J. et al. (2017)<br>Luo, Z. et al. (2017b)<br>Efimova et al. (2019)<br>Tillman et al. (2020)<br>Takahashi et al. (2021)
Balechina				
pachydermata (= Gymnodinium amphora)	Y			Gómez et al. (2015a,b)<br>Gómez et al. (2016b)<br>Li et al. (2017)
Baldinia				
anauniensis<br>greenGS	YP		Y	Hansen et al. (2007a)<br>Moestrup et al. (2008)<br>Hansen and Daugbjerg (2011)<br>Hoppenrath et al. (2012a)<br>Mertens et al. (2012)<br>Gottschling and McLean (2013)<br>Jeong et al. (2014c)<br>Moestrup et al. (2014)<br>Pandeirada et al. (2014)<br>Takahashi et al. (2015)<br>Boutrup et al. (2016)<br>Luo et al. (2016b)<br>Zhang et al. (2016)<br>Jang et al. (2017a,b)<br>Hansen et al. (2018)<br>LaJeunesse et al. (2018)<br>Wakeman et al. (2018a)<br>Gottschling et al. (2021a,b)
bernardiensis (= Glenodinium bernardinense)			YP	Moestrup et al. (2009a)<br>Jeong et al. (2014c)
Barrufeta				
bravensis	Y		Y	Reñé et al. (2011)<br>Sampedro et al. (2011)<br>Nézan et al. (2014)<br>Reñé et al. (2014)<br>Gu et al. (2015a)<br>Reñé et al. (2015)<br>Annenkova (2018)<br>Luo et al. (2018b)<br>Romeikat et al. (2019)
resplendens<br>GM17	YP	YP	Y	Gu et al. (2015a)<br>Na et al. (2017)

				Annenkova (2018)<br>Luo et al. (2018b)<br>Gómez et al. (2019a)<br>Romeikat et al. (2019)<br>Gottschling et al. (2021a,b)
Bernardinium				
bernardinense (= Hemidinium bernardinense)			YP	Moestrup et al. (2009a)<br>Jeong et al. (2014c)<br>Zhang et al. (2016)
sp.	YP			Zhang et al. (2016)
Biecheleria				
baltica	Y		Y	Moestrup et al. (2009b)<br>Siano et al. (2010)<br>Hansen and Daugbjerg (2011)<br>Balzano et al. (2012)<br>Jeong et al. (2014a,c)<br>Takahashi et al. (2015)<br>Luo et al. (2016b)<br>Jang et al. (2017a,b)<br>Hehenberger et al. (2017)<br>Li et al. (2017)<br>Raho et al. (2018)<br>Selina and Efimova (2020)
brevisulcata<br>trd276-kt	Y/YA		Y	Jeong et al. (2014a)<br>Takahashi et al. (2014)<br>Takahashi et al. (2015)<br>Hehenberger et al. (2017)<br>Jang et al. (2017a,b)<br>Kang and Wang (2018)<br>Raho et al. (2018)<br>Ribeiro et al. (2019)<br>Gottschling et al. (2021b)
cestocoetes	N			Raho et al. (2018)
cincta (= Woloszynskia cincta)	Y/YA	YP	Y	Jørgensen et al. (2004a)<br>Balzano et al. (2012)<br>Gottschling et al. (2012)<br>Jeong et al. (2014a,c)<br>Saburova and Chomérat (2014)<br>Takahashi et al. (2014)<br>Takahashi et al. (2015)<br>Anglès et al. (2017)<br>Hehenberger et al. (2017)<br>Jang et al. (2017a,b)<br>Li et al. (2017)<br>Na et al. (2017)<br>Gu et al. (2018)<br>LaJeunesse et al. (2018)

				Luo et al. (2018b)<br>Raho et al. (2018)<br>Ribeiro et al. (2019)
halophila (= Woloszynskia halophile)	Y		YP	Kremp et al. (2005)<br>Gottschling et al. (2008)<br>Moestrup et al. (2008)<br>Gómez et al. (2009a)<br>Gómez et al. (2010a,b)<br>Annenkova et al. (2011)<br>Miranda et al. (2012)<br>Gómez et al. (2015b)<br>LaJeunesse et al. (2018)<br>Wakeman et al. (2018a)
natalensis<br>Cx7 LC054923	YP			Yamada et al. (2015)<br>Raho et al. (2018)
pesudopalustris (= Woloszynskia pseudopalustris) In many of the D1-D2 phylogenies, B. brevisulcata, baltica, cincta and Woloszynskia, Gymnodinium sp – one sequence each – are not resolved. Likely wrong IDs on sequences.			YP/YA	Daugbjerg et al. (2000)<br>Murray et al. (2005)<br>Gribble and Anderson (2006)<br>Kim and Kim (2007)<br>Moestrup et al. (2007)<br>Moestrup et al. (2008)<br>Moestrup et al. (2009b)<br>Siano et al. (2009)<br>Siano et al. (2010)<br>Hansen and Daugbjerg (2011)<br>Jeong et al. (2014a,c)<br>Takahashi et al. (2015)<br>Boutrup et al. (2016)<br>Luo et al. (2016b)<br>Jang et al. (2017a,b)<br>Kang and Wang (2018)<br>LaJeunesse et al. (2018)
tirezensis	YP	YP	YP	Raho et al. (2018)
Biecheleriopsis				
adriatica<br>trd278-kt	Y	Y	Y	Moestrup et al. (2009b)<br>Hansen and Daugbjerg (2011)<br>Balzano et al. (2012)<br>Jeong et al. (2014a,c)<br>Takahashi et al. (2014)<br>Jang et al. (2015)<br>Takahashi et al. (2015)<br>Boutrup et al. (2016)<br>Hehenberger et al. (2017)<br>Jang et al. (2017a,b)<br>Li et al. (2017)<br>Na et al. (2017)<br>Kang and Wang (2018)

				LaJeunesse et al. (2018)<br>Luo et al. (2018b)<br>Raho et al. (2018)<br>Gottschling et al. (2021a,b)
Bispinodinium				
angelaceum<br>HG236 AB762397	YP	YP	YP	Yamada et al. (2013)<br>Saburova and Chomérat (2014)<br>Reñé et al. (2015)<br>Yamada et al. (2015)<br>Gómez et al. (2016a)<br>Gottschling et al. (2021a,b)
Blixaea				
quinquecornis (= Peridinium quinquecorne)	YP			Ki et al. (2011)<br>Saburova et al. (2012)<br>Saburova and Chomérat (2014)<br>Kang et al. (2015)<br>Luo et al. (2015)<br>Horiguchi et al. (2017)<br>Pinto et al. (2017)<br>Yamada et al. (2017)<br>Dawut et al. (2018)<br>Satta et al. (2020)
Blastodinium				
contortum	Y	Y	YP	Skovgaard et al. (2007)<br>Skovgaard and Salomonsen (2009)<br>Coats et al. (2010)<br>Alves-De-Souza et al. (2011)<br>Qiu et al. (2011)<br>Okamoto et al. (2012)<br>Skovgaard et al. (2012)<br>Gu et al. (2013a)<br>Hoppenrath et al. (2013)<br>Kim and Park (2014)<br>Gómez and Skovgaard (2015)<br>Jung et al. (2015)<br>Gómez (2016b)<br>Anglès et al. (2017)<br>Kretschmann et al. (2018b)<br>Yamamoto et al. (2020)<br>Gottschling et al. (2021a,b)
crassum	YP	YP	YP	Skovgaard and Salomonsen (2009)<br>Coats et al. (2010)<br>Gu et al. (2013a)<br>Anglès et al. (2017)<br>Kretschmann et al. (2018b)<br>Žerdoner Čalasan et al. (2019)<br>Gottschling et al. (2021a,b)

galatheanum	Y	Y		Skovgaard and Salomonsen (2009)<br>Coats et al. (2010)<br>Skovgaard et al. (2012)<br>Gómez and Skovgaard (2015)<br>Gómez (2016b)
mangini	Y	Y		Alves-De-Souza et al. (2011)<br>Skovgaard et al. (2012)
cf. mangini	Y	Y		Skovgaard et al. (2012)
mangini var oncaea	Y	Y		Skovgaard et al. (2012)
navicula	Y	Y		Skovgaard et al. (2007)<br>Skovgaard and Salomonsen (2009)<br>Coats et al. (2010)<br>Gómez et al. (2010a,b)<br>Skovgaard et al. (2012)<br>Gómez and Skovgaard (2015)<br>Gómez (2016b)<br>Yamamoto et al. (2020)
oviforme	YP	Y		Skovgaard et al. (2012)<br>Hoppenrath et al. (2017)<br>Selina et al. (2019)
pruvoti	YP			Skovgaard and Salomonsen (2009)
spinulosum Group I (includes seqs. from B. spinulosum, B. cassum, B. inornatum, cf. pruvoti)	Y	Y		Alves-De-Souza et al. (2011)<br>Okamoto et al. (2012)<br>Skovgaard et al. (2012)
spinulosum Group 2 (undescribed species)	Y	Y		Alves-De-Souza et al. (2011)<br>Skovgaard et al. (2012)
spinulosum Group 3 (seqs. from B. spinulosum and B. cf. spinulosum)	Y	Y		Skovgaard et al. (2012)
Blepharocysta				
sp. FG216	YP			Gómez et al. (2010a,b)<br>Li et al. (2017)<br>Hoppenrath et al. (2020)
Boreadinium				
breve	YP	YP	Y	Liu et al. (2015a)<br>Mertens et al. (2015b)<br>Yamaguchi et al. (2016)<br>Reñé and Hoppenrath (2019)<br>Žerdoner Čalasan et al. (2019)<br>Gurdebek et al. (2020)
Borghiella				
anderseni	YP		YP	Takahashi et al. (2015)<br>Boutrup et al. (2016)<br>Boutrup et al. (2017)<br>Jang et al. (2017a)<br>Moestrup et al. (2018)

dodgei<br>CCAC0075 MG851594	YP		YP	Moestrup et al. (2008)<br>Siano et al. (2010)<br>Hansen and Daugbjerg (2011)<br>Jeong et al. (2014a,c)<br>Pandeirada et al. (2014)<br>Takahashi et al. (2014)<br>Takahashi et al. (2015)<br>Boutrup et al. (2016)<br>Luo et al. (2016b)<br>Zhang et al. (2016)<br>Boutrup et al. (2017)<br>Jang et al. (2017a)<br>LaJeunesse et al. (2018)<br>Moestrup et al. (2018)<br>Gottschling et al. (2021b)
pascheri (= Woloszynskia pascheri)	YP	YP	YP/YA	Gómez et al. (2009a)<br>Takahashi et al. (2015)<br>Gómez et al. (2015b)<br>Jang et al. (2017a,b)<br>Li et al. (2017)<br>Moestrup et al. (2018)
sp.<br>CCAC0075<br>GeoM*542 MK405487<br>MK405488	YP	YP	YP	Gottschling et al. (2021a,b)
tenuissima (= Woloszynskia tenuissima)<br>A NIES 619 Y443025	YP		YP	Hansen and Daugbjerg (2004)<br>Iwataki et al. (2007)<br>Kim and Kim (2007)<br>Moestrup et al. (2007)<br>Moestrup et al. (2008)<br>Hoppenrath et al. (2009)<br>Siano et al. (2010)<br>Hansen and Daugbjerg (2011)<br>Jeong et al. (2014a,c)<br>Moestrup et al. (2014)<br>Pandeirada et al. (2014)<br>Takahashi et al. (2014)<br>Takahashi et al. (2015)<br>Yamada et al. (2015)<br>Boutrup et al. (2016)<br>Luo et al. (2016b)<br>Zhang et al. (2016)<br>Boutrup et al. (2017)<br>Jang et al. (2017a,b)<br>LaJeunesse et al. (2018)<br>Moestrup et al. (2018)
Brachidiniaceae				

sp.<br>GrAr01	YP			Gottschling et al. (2021b)
Brachidinium				
capitatum	YP	YP	YP	Henrichs et al. (2011)<br>Nézan et al. (2014)<br>Gómez et al. (2015b)<br>Gómez et al. (2016a,b)<br>Li et al. (2017)<br>Wang et al. (2018)<br>Gómez et al. (2019a)<br>Takahashi et al. (2019)
Brandtodinium				
nutricula (= Zooxanthella nutricula)<br>BBSR323	Y	YP	Y	Gottschling and McLean (2013)<br>Gu et al. (2013a)<br>Probert et al. (2014)<br>Gómez (2016b)<br>Kretschmann et al. (2018b)<br>Žerdoner Čalasan et al. (2019)<br>Gottschling et al. (2021a,b)
Breviolum (Symbiodinium clade B)				
Symbiodinium sp. clade B (SSU sequences do not correspond directly with LSU sequences)	Y	Y		Takishita et al. (2003)<br>Strychar et al. (2005)<br>Jeong et al. (2014a)<br>Hehenberger et al. (2017)<br>Jang et al. (2017b)<br>Rodríguez et al. (2019)<br>Shi et al. (2020)
aenigmaticum<br>B19 lineage variant		Y	Y	LaJeunesse (2005)<br>Parkinson et al. (2015)
antillogorgium (= Symbiodinium antillogorgium, Zooxanthella antillogorgia)<br>CCMP3449 (=mac08-0689)<br>ITS2 KT149341<br>LSU KT149346		Y	Y	LaJeunesse (2001)<br>Parkinson et al. (2015)<br>LaJeunesse et al. (2018)
dendrogyrum<br>ITS2 B1 MH974805<br>B1k GU907638<br>LSU rDNA MH728999		Y	Y	LaJeunesse (2005)<br>LaJeunesse et al. (2018)<br>Lewis et al. (2019)
endomadracis (clade B7) (= Symbiodinium endomadracis, Zooxanthella endomadracis)<br>CCMP3448 =mac04-180<br>ITS2 B13 or B15<br>ITS2 KT149342-KT149343<br>LSU KT149347-KT149348		Y	Y	Parkinson et al. (2015)<br>LaJeunesse et al. (2018)<br>Lewis et al. (2019)
faviinorum		Y	Y	LaJeunesse et al. (2018)

ITS2 B1 EU449064<br>B14, B14a EU449084-EU449086<br>B24 GU907643<br>LSU MH728997				Lewis et al. (2019)
meandrinium<br>ITS2 B1 MH974804<br>B20 GU907642<br>LSU MH728998		Y	Y	LaJeunesse et al. (2018)<br>Lewis et al. (2019)
minutum (= Symbiodinium minutum, Zooxanthella minuta)<br>B1 variant<br>Mf 1.05b.01<br>NCMA830<br>Type B1 AF424559	YP	Y	Y	LaJeunesse et al. (2012)<br>Jeong et al. (2014b)<br>Parkinson et al. (2015)<br>LaJeunesse et al. (2018)<br>Gottschling et al. (2021a,b)
pseudominutum<br>CCMP3450 (=rt146)<br>B1 ITS ribotype<br>ITS2 KT149344<br>LSU KT149350		Y	Y	Parkinson et al. (2015)<br>LaJeunesse et al. (2018)
psygmophilum (= Symbiodinium pseudominutum, Zooxanthella pseudominuta) B1/B19 lineange variant<br>Type B2 AF427460		Y	Y	LaJeunesse (2005)<br>LaJeunesse et al. (2012)<br>Jeong et al. (2014b)<br>Parkinson et al. (2015)<br>LaJeunesse et al. (2018)
Bysmatrum				
arenicola	YA		YA	Dawut et al. (2018)<br>Luo et al. (2019)
austrafrum	YP			Dawut et al. (2018)
granulosum	Y	Y	Y	Luo et al. (2018a)<br>Gómez et al. (2019b)
gregarium (= Bysmatrum caponii)<br>BCGRL1	Y	Y	Y	Anglès et al. (2017)<br>Dawut et al. (2018)<br>Luo et al. (2018a)<br>Gómez et al. (2019b)<br>Gottschling et al. (2021b)
subsalsum (clade 1)<br>FB1<br>FB2 KY593819<br>FB3<br>Mediterranean Sea<br>-2 Mediterranean Sea HQ593830<br>-4 Mediterranean Sea HQ593827	Y	Y	Y	Anglès et al. (2017)<br>Dawut et al. (2018)<br>Luo et al. (2018a)

-5 Mediterranean Sea HQ593833<br>-8 Mediterranean Sea HQ593816 HQ845326<br>-9 Mediterranean Sea HQ593827<br>-21 Mediterranean Sea HQ593822				
subsalsum (clade 2)<br>CI<br>D323 HQ845326<br>KC32CCAUTH<br>PL1 KY593827<br>PL2<br>PL3<br>PL wild cells<br>TBBYS02<br>TBBYS02<br>TIO406	Y	Y	Y	Gottschling et al. (2012)<br>Anglès et al. (2017)<br>Dawut et al. (2018)<br>Luo et al. (2018a)<br>Gottschling et al. (2021a,b)
Cabra				
matta	YP			Yamaguchi et al. (2018)<br>Gómez et al. (2019b)
Caladoa				
arcachonensis	Y	Y	Y	Luo et al. (2019)<br>Luo, Z. et al. (2020)<br>Gottschling et al. (2021a)
Calcicarpinum				
bivalvum<br>GeoB*230	YP	YP	YP	Gottschling et al. (2012)<br>Craveiro et al. (2013)<br>Gottschling and McLean (2013)<br>Gu et al. (2013a)<br>Tillmann et al. (2014)<br>Kretschmann et al. (2018b)<br>Gottschling et al. (2021b)
Calciodinellum				
albatrosianum (= Sphaerodinella albatrosiana, Thoracosphaera albatrosiana)	Y	Y	Y	Gottschling et al. (2005a)<br>Attaran-Fariman and Bolch (2007)<br>Gottschling et al. (2008)<br>Zinssmeister et al. (2011)<br>Zinssmeister et al. (2012)<br>Craveiro et al. (2013)<br>Gu et al. (2013c)<br>Kretschmann et al. (2014)<br>Craveiro et al. (2015)<br>Efimova et al. (2019)<br>Lee, S.Y. et al. (2019)

				Kim, H.J. et al. (2021)
elongatum (= Sphaerodinella tuberosa f. elongate)<br>GeoB*161 D066 MH497039<br>GeoB 335 D307 JN982397<br>HQ729489<br>KF751926	YP	YP	YP	Kretschmann et al. (2014)<br>Lee, S.Y. et al. (2019)<br>Žerdoner Čalasan et al. (2019)<br>Gottschling et al. (2021b)
levantinum<br>GeoB 122 D052 MH497035	YP	Y	Y	Gottschling et al. (2005a)<br>Attaran-Fariman and Bolch (2007)<br>Gottschling et al. (2008)<br>Soehner et al (2012)<br>Craveiro et al. (2013)<br>Kretschmann et al. (2014)<br>Craveiro et al. (2015)<br>Li et al. (2015)<br>Žerdoner Čalasan et al. (2019)<br>Kim, H.J. et al. (2021)
operosum / aff. operosum (= Scrippsiella operosa – placement in genus Calciodinellum or Scrippsiella unresolved. Requires further investigation.)<br>GeoB*31<br>GeoB 34<br>M23*26/4<br>SZN74	YP	YP	YP	D'Onofrio et al. (1999)<br>Montresor et al. (2003)<br>Gottschling et al. (2005a,b)<br>Attaran-Fariman and Bolch (2007)<br>Gottschling et al. (2008)<br>Zinssmeister et al. (2011)<br>Zinssmeister et al. (2012)<br>Craveiro et al. (2013)<br>Gu et al. (2013c)<br>Kretschmann et al. (2014)<br>Craveiro et al. (2015)<br>Craveiro et al. (2016)<br>Efimova et al. (2019)<br>Lee, S.Y. et al. (2019)<br>Žerdoner Čalasan et al. (2019)<br>Li, Z. et al. (2020)<br>Gottschling et al. (2021a,b)<br>Kim, H.J. et al. (2021)
sp.		Y		Soehner et al (2012)
sp. tub*2	YP	YP	YP	Kretschmann et al. (2014)
Centrodinium				
eminens	YA		YA	Gómez and Artigas (2019)<br>Selina and Efimova (2020)
intermedium	YA		YA	Gómez and Artigas (2019)<br>Selina and Efimova (2020)
punctatum (= C. splendidum)	Y	Y	Y	Gómez and Artigas (2019)<br>Li et al. (2019)<br>Selina and Efimova (2020)
Ceratium				
furcoides	Y	YP	YP	Accattatis et al. (2020)

HBI:SC201002a				Selina and Efimova (2020)<br>Gottschling et al. (2021a,b)
hirundinella<br>WA28-13	Y			Gómez et al. (2010a,b,c)<br>Gómez et al. (2015a)<br>Mertens et al. (2015b)<br>Accattatis et al. (2020)<br>Selina and Efimova (2020)<br>Gottschling et al. (2021b)
sp.<br>HCB-2005	YP			Gómez et al. (2010c)
Ceratocorys				
armata	N		NA	Selina and Efimova (2020)<br>Zhang et al. (2020)
gourretii			NA	Zhang et al. (2020)
horrida<br>GeoB 183	YP	YP	NA/YP	Grzebyk et al. (1998)<br>Gómez et al. (2010a,b,c)<br>Miranda et al. (2012)<br>Reñé and Hoppenrath (2019)<br>Luo, Z. et al. (2020a)<br>Zhang et al. (2020)<br>Gottschling et al. (2021a,b)
malayensis (not clear whether or not if Ceratocrys horrida, C. gourretii, and C armata LSU sequences are from misidentified isolates – see Luo, Z. et al. 2020a)		Y	Y/YA	Luo, Z. et al. (2020a)<br>Selina and Efimova (2020)<br>Zhang et al. (2020)
mariaovidiorum		Y		Salgado et al. (2018)
sp.<br>NY002 LC054924	YP			Yamada et al. (2015)
Ceratoperidinium				
margalefii			Y	Reñé et al. (2013)<br>Reñé et al. (2015)<br>Boutrup et al. (2017)<br>Gómez (2018)<br>Hu et al. (2020)<br>Shin and Matsuoka (2020)
Citharistes				
apsteinii - in Park et al (2011) there are 2 distinct LSU clusters consistne with separate C. apsteinii and C. regius species suggesting several isolates were originally misidentified.			YP	Park et al (2011)
regius			Y	Jensen and Daugbjerg (2009)<br>Park et al (2011)

Cladocopium				
goreaui (C1)<br>113c<br>152c FJ529523<br>NCMA2466	Y	Y	Y	LaJeunesse (2001)<br>LaJeunesse (2005)<br>Sampayo et al. (2009)<br>Gottschling et al. (2012)<br>Zinssmeister et al. (2012)<br>Gottschling and McLean (2013)<br>Jeong et al. (2014b)<br>LaJeunesse et al. (2018)<br>Teschima et al. (2019)<br>Boo et al. (2020)<br>Shi et al. (2020)<br>Gottschling et al. (2021a,b)
thermophilum (C3b-Gulf C3)			YP	Sampayo et al. (2009)<br>Hume et al. (2015)<br>LaJeunesse et al. (2018)<br>Shi et al. (2020)
sp.<br>C1c KF740674		Y		Jeong et al. (2014b)<br>Shi et al. (2020)
sp.<br>Type C1d KF740675			YP	Jeong et al. (2014b)
sp.<br>C2 203wp<br>LaJeunesse 2001 KF740672	Y	Y	Y	LaJeunesse (2001)<br>Takishita et al. (2003)<br>Strychar et al. (2005) (SSU sequences not mapped in detail to specific species)<br>Siano et al. (2010)<br>Jeong et al. (2014a,b)<br>Probert et al. (2014)<br>Hehenberger et al. (2017)<br>Jang et al. (2017b)<br>LaJeunesse et al. (2018)<br>Rodríguez et al. (2019)<br>Teschima et al. (2019)
sp.<br>C3<br>C3b<br>C3u KF740676<br>C3n-t FJ529530		Y		LaJeunesse (2005)<br>Jeong et al. (2014b)<br>Hume et al. (2015)<br>Shi et al. (2020)
sp.<br>clade C15		Y		Boo et al. (2020)<br>Shi et al. (2020)
sp.<br>C7/C7a KF740677		Y	YP	Jeong et al. (2014a)<br>Hume et al. (2015)
sp.<br>C8		Y		LaJeunesse (2005)
sp.<br>C8a FJ529526			YP	Jeong et al. (2014b)

sp.<br>C15 KF740678		Y	YP	LaJeunesse (2005)<br>Jeong et al. (2014b)<br>Shi et al. (2020)
sp.<br>C17a		Y		Hume et al. (2015)<br>Shi et al. (2020)
sp.<br>C21 KF740679		Y	YP	LaJeunesse (2005)<br>Jeong et al. (2014b)
sp.<br>C26a		Y		Hume et al. (2015)
sp.<br>C27		Y		Hume et al. (2015)
sp.<br>C27a		Y		Hume et al. (2015)
sp.<br>C31 KF740680		Y	YP	LaJeunesse (2005)<br>Jeong et al. (2014b)<br>Hume et al. (2015)<br>Shi et al. (2020)
sp.<br>C33 FJ529532			YP	Jeong et al. (2014b)
sp.<br>C33a FJ529531			YP	Jeong et al. (2014b)
sp.<br>C35a FJ529529			YP	Jeong et al. (2014b)
sp.<br>C40 KF740681		Y	YP	Jeong et al. (2014b)<br>Hume et al. (2015)
sp.<br>C42		Y		LaJeunesse (2005)
sp.<br>C42a FJ529525			YP	Jeong et al. (2014b)
sp. C45		Y		LaJeunesse (2005)
sp.<br>C57 KF740682				Jeong et al. (2014b)
sp.<br>C62		Y		Shi et al. (2020)
sp.<br>C66		Y		LaJeunesse (2005)
sp.<br>C71a KF740683			YP	Jeong et al. (2014b)
sp.<br>C78a FJ529527			YP	Jeong et al. (2014b)
sp.<br>C79 FJ529528			YP	Jeong et al. (2014b)
sp.<br>C91		Y		Shi et al. (2020)
sp.<br>C130		Y		Teschima et al. (2019)
sp.			YP	Jeong et al. (2014b)

C140 KF740684				
sp.<br>C1143		Y		Shi et al. (2020)
sp.<br>C1169		Y		Shi et al. (2020)
Chimonodinium				
lomnickii (= Peridinium lomnickii)	YP	YP	YP	Craveiro et al. (2011)<br>Moestrup et al. (2014)<br>Annenkova et al. (2015)<br>Zhang et al. (2016)<br>Boutrup et al. (2017)<br>Hoppenrath et al. (2017)<br>Saburova and Chomérat (2018)<br>Luo et al. (2019)<br>Žerdoner Čalasan et al. (2019)<br>Hoppenrath et al. (2020)
lomnickii var. wierzejskii<br>GeoM*715	YP	YP	YP	Kretschmann et al. (2018b)<br>Žerdoner Čalasan et al. (2019)<br>Luo, Z. et al. (2020)<br>Gottschling et al. (2021b)
Chytriodinium				
affine	Y	YP	YP	Gomez et al. (2009b)<br>Coats et al. (2010)<br>Gómez and Skovgaard (2014)<br>Gómez et al. (2016b)<br>Strassert et al. (2018)<br>Žerdoner Čalasan et al. (2019)
roseum<br>FG192	YP	YP	YP	Gomez et al. (2009b)<br>Coats et al. (2010)<br>Gómez and Skovgaard (2014)<br>Gómez et al. (2015b)<br>Gómez et al. (2016b)<br>Strassert et al. (2018)<br>Žerdoner Čalasan et al. (2019)
sp.<br>Atlantic isolates 1-7<br>KM245128	Y	YP	Y	Gómez and Skovgaard (2014)<br>Gómez and Skovgaard (2015)<br>Na et al. (2017)<br>Luo et al. (2018b)<br>Strassert et al. (2018)<br>Gómez et al. (2019a)<br>Romeikat et al. (2019)<br>Gottschling et al. (2021a)
sp.<br>KJ22-3-14 KT389895	YP		YP	Romeikat et al. (2019)<br>Žerdoner Čalasan et al. (2019)
Cladocopium				
sp.<br>genotype 01		Y		Mizuyama et al. (2020)

sp.<br>genotype 02		Y		Mizuyama et al. (2020)
sp.<br>clade C (537 seq)		Y		Qin et al. (2019)
Cochlodinium				
sp. 1			YP	Reñé et al. (2013)<br>Reñé et al. (2015)
sp.<br>AR300 KP790181			YP	Reñé et al. (2015)
sp.<br>FG12_clone3 KY468922			YP	Hu et al. (2019)
strangulatum (= Cochlodinium convolutum)			YP	Reñé et al. (2013)<br>Reñé et al. (2015)
Coolia				
canariensis phylogroup I<br>NQAIF252 HQ897282<br>VGO786 AM902737<br>VGO787 AM902738	YP		Y	Fraga et al. (2008)<br>Leaw et al. (2010)<br>Laza-Martinez et al. (2011)<br>Jeong et al. (2012)<br>Mohammad-Noor et al. (2013)<br>David et al. (2014b)<br>Rhodes et al. (2014)<br>Wakeman et al. (2015)<br>Gómez, et al. (2016d)<br>Leaw et al. (2016)<br>Leung et al. (2017)<br>Nascimento et al. (2019)<br>Tibiriçá et al. (2020)<br>Gottschling et al. (2021b)
canariensis phylogroup II<br>UNR-25 MK109023		YP	YP	Nascimento et al. (2019)<br>Tibiriçá et al. (2020)
canariensis phylogroup III<br>Dn137EHU KF956847<br>CMJJ1 FR847224<br>UNR-25 MK109023<br>NQAIF252 HQ897278<br>Dn137EHU KF896838<br>SKLMP_Ve011 KX589150<br>Dn206EHU<br>SKLMP S037<br>CMJJ1	Y	Y	Y	Fraga et al. (2008)<br>Leaw et al. (2010)<br>Laza-Martinez et al. (2011)<br>Jeong et al. (2012)<br>Mohammad-Noor et al. (2013)<br>David et al. (2014b)<br>Rhodes et al. (2014)<br>Wakeman et al. (2015)<br>Gómez, et al. (2016d)<br>Leaw et al. (2016)<br>Shin et al. (2016)<br>Leung et al. (2017)<br>Zhang, H. et al. (2017)<br>Moreira-González et al. (2019)<br>Nascimento et al. (2019)<br>Reñé and Hoppenrath (2019)<br>David et al. (2020)

				Zhang, H. et al. (2020)
guanchica		Y	YP	David et al. (2020)<br>Tibiriçá et al. (2020)
malayensis	Y	Y	Y	Penna et al. (2005b)<br>Litaker et al. (2007)<br>Fraga et al. (2008)<br>Leaw et al. (2010)<br>Laza-Martinez et al. (2011)<br>Jeong et al. (2012)<br>David et al. (2014b)<br>Rhodes et al. (2014)<br>Tawong et al. (2015)<br>Wakeman et al. (2015)<br>Gómez, et al. (2016d)<br>Leaw et al. (2016)<br>Verma et al. (2016a)<br>Kim and Park (2017)<br>Leung et al. (2017)<br>Efimova et al. (2019)<br>Moreira-González et al. (2019)<br>Nascimento et al. (2019)<br>David et al. (2020)<br>Tibiriçá et al. (2020)<br>Zhang, H. et al. (2020)<br>Gottschling et al. (2021b)
cf. malayensis			Y	Gómez, et al. (2016d)
monotis (= Ostreopsis monotis)	1. Y	Y	Y	Penna et al. (2005b)<br>Dolapsakis et al. (2006)<br>Litaker et al. (2007)<br>Fraga et al. (2008)<br>Gómez et al. (2010a,b)<br>Leaw et al. (2010)<br>Laza-Martinez et al. (2011)<br>Gottschling et al. (2012)<br>Jeong et al. (2012)<br>Amorim et al. (2013)<br>Mohammad-Noor et al. (2013)<br>David et al. (2014b)<br>Efimova et al. (2014)<br>Rhodes et al. (2014)<br>Wakeman et al. (2015)<br>Gómez, et al. (2016d)<br>Leaw et al. (2016)<br>Verma et al. (2016a)<br>Kim and Park (2017)<br>Leung et al. (2017)<br>Gu et al. (2018)<br>Wakeman et al. (2018a)

				Moreira-González et al. (2019)<br>Nascimento et al. (2019)<br>Reñé and Hoppenrath (2019)<br>David et al. (2020)<br>Selina and Efimova (2020)<br>Tibiriçá et al. (2020)<br>Gottschling et al. (2021b)
palmyrensis		Y	Y	Gómez, et al. (2016d)<br>Leaw et al. (2016)<br>Leung et al. (2017)<br>Moreira-González et al. (2019)<br>Nascimento et al. (2019)<br>David et al. (2020)<br>Tibiriçá et al. (2020)
santacroce		Y	YP	Gómez, et al. (2016d)<br>Leaw et al. (2016)<br>Leung et al. (2017)<br>Moreira-González et al. (2019)<br>Nascimento et al. (2019)<br>David et al. (2020)<br>Tibiriçá et al. (2020)
sp. 1<br>NQAIF103 HQ897277	YP		YP	David et al. (2014b)
tropicalis<br>CCMP 1744<br>VGO923		Y	Y	Jeong et al. (2012)<br>Mohammad-Noor et al. (2013)<br>David et al. (2014b)<br>Rhodes et al. (2014)<br>Wakeman et al. (2015)<br>Gómez, et al. (2016d)<br>Leaw et al. (2016)<br>Moreira-González et al. (2019)<br>Nascimento et al. (2019)<br>David et al. (2020)<br>Tibiriçá et al. (2020)<br>Zhang, H. et al. (2020)
Corythodinium				
cristatum	Y			Gómez (2016b)
frenguellii	Y			Gómez (2016b)
tesselatum	Y			Gómez (2016b)<br>Gómez and Artigas (2019)
Crypthecodinium				
cohnii (= Crypthecodinium setense)	Y	YP	YP	McNally et al. (1994)<br>Grzebyk et al. (1998)<br>Gómez et al. (2010b)<br>Yamaguchi et al. (2011)<br>Miranda et al. (2012)<br>Prabowo, et al. (2013)

				Cooney et al. (2020)<br>Selina and Efimova (2020)
sp.<br>CAAE CL2	YP			Gómez et al. (2010b)<br>Cooney et al. (2020)<br>Gottschling et al. (2021b)
sp.<br>clade 1	Y	Y	Y	Prabowo, et al. (2013)
sp.<br>clade 2	Y	Y	Y	Prabowo, et al. (2013)
Cryptoperidiniopsis				
brodyi<br>CBDE1 DQ991372<br>CBDE2 DQ991373<br>CBDE10 DQ991374<br>H/V14 AY245690	Y	Y	Y	Saito et al. (2002)<br>Litaker et al. (2005)<br>Seaborn et al. (2006)<br>Litaker et al. (2007)<br>Mason et al. (2007)<br>Park et al. (2007)<br>Coats et al. (2010)<br>Gottschling et al. (2012)<br>Miranda et al. (2012)<br>Craveiro et al. (2013)<br>Gottschling and McLean (2013)<br>Gu et al. (2013a)<br>Jeong et al. (2014c)<br>Kretschmann et al. (2014)<br>Tillmann et al. (2014)<br>Kang et al. (2015)<br>Jung et al. (2015)<br>Kretschmann et al. (2018b)<br>Hoppenrath et al. (2020)<br>Li, Z. et al. (2020)<br>Kim, H.J. et al. (2021)
sp.<br>F525Jul02 AY590480	Y	Y	Y	Mason et al. (2007)<br>Craveiro et al. (2013)<br>Tillmann et al. (2014)<br>Žerdoner Čalasan et al. (2019)<br>Li, Z. et al. (2020)
sp.<br>NOAA Beach AY590486	Y	Y	Y	Mason et al. (2007)<br>Craveiro et al. (2013)<br>Kretschmann et al. (2014)<br>Tillmann et al. (2014)<br>Hoppenrath et al. (2020)
sp.<br>PLO21 AY245691	Y	Y	Y	Mason et al. (2007)<br>Craveiro et al. (2013)<br>Kretschmann et al. (2014)<br>Tillmann et al. (2014)
sp.<br>NCMA (CCMP) 1828	Y	Y	Y	Mason et al. (2007)<br>Craveiro et al. (2013

AY590476				Kretschmann et al. (2014)<br>Tillmann et al. (2014)<br>Žerdoner Čalasan et al. (2019)<br>Hoppenrath et al. (2020)
sp.<br>Folly C5 AY590481		YP	YP	Mason et al. (2007)<br>Craveiro et al. (2013)<br>Kretschmann et al. (2014)<br>Tillmann et al. (2014)<br>Hoppenrath et al. (2020)
Cystodinium				
phaseolus	YP			Qiu et al. (2011)<br>Takahashi et al. (2015)<br>Jang et al. (2017b)
Cucumeridinium				
coeruleum (= Gymnodinium coeruleum, Balechina coerulea)	Y			Qiu et al. (2013)<br>Gómez et al. (2015b)<br>Gómez et al. (2016b)<br>Li et al. (2017)
cucumis	YP			Gómez et al. (2015b)<br>Li et al. (2017)
lira (= Gymnodinium lira)	YP			Gómez et al. (2015b)<br>Gómez et al. (2016b)<br>Li et al. (2017)
Dactylodinium				
arachnoides			Y	Lum et al. (2019)
pterobelotum<br>vnd255-kt	YP	YP	YP	Takahashi et al. (2017)<br>LaJeunesse et al. (2018)<br>Gottschling et al. (2021a,b)
Dapsilidinium				
pastielsii			YP	Mertens et al. (2017a)<br>Selina and Efimova (2020)<br>Zhang et al. (2020)
Dinophysis				
acuminata clade 1 complex (= Dinophysis acuminata f. lachmannii, Dinophysis borealis, Dinophysis ellipsoids, Dinophysis lachmannii, Dinophysis skagii) DARU-2013 clone 2<br>SSU sequences equivalent for D. acuminata, D. caudata, D. miles, D. norvegica, Dinophysis sp. FPIP, D. tripos	N	N	N	Guillou et al. (2002)<br>Rehnstam-Holm et al. (2002)<br>Edvardsen et al. (2003)<br>Litaker et al. (2007)<br>Kim and Kim (2007)<br>Raho et al. (2008)<br>Handy et al. (2009)<br>Gómez et al. (2010a,b)<br>Gómez et al. (2011a)<br>Qiu et al. (2011)<br>Reñé et al. (2013)<br>Saburova and Chomérat (2014)<br>Takano et al. (2014)<br>Wolny et al. (2020)

				Gottschling et al. (2021b)
acuta clade 3 (= Dinophysis dens)<br>SSU sequences equivalent for D. acuta, D. fortii, D. infundibulus	N	N	N	Rehnstam-Holm et al. (2002)<br>Edvardsen et al. (2003)<br>Gottschling et al. (2005a)<br>Kim and Kim (2007)<br>Handy et al. (2009)<br>Gómez et al. (2011a)<br>Qiu et al. (2011)<br>Saburova and Chomérat (2014)<br>Takano et al. (2014)<br>Mertens et al. (2015b)<br>Baytut et al. (2016)<br>Gómez and Artigas (2019)
cf. acuta	YP			Qiu et al. (2011)
argus (= Phalacroma argus)			YP	Jensen and Daugbjerg (2009)
braarudii			YP	Jensen and Daugbjerg (2009)<br>Park et al (2011)
caudata (clade 5)<br>FPIP EU780641<br>FTL69	N	Y	N	Guillou et al. (2002)<br>Edvardsen et al. (2003)<br>Gottschling et al. (2005a)<br>Raho et al. (2008)<br>Handy et al. (2009)<br>Gómez et al. (2011a)<br>Qiu et al. (2011)<br>Gottschling et al. (2012)<br>Rodríguez et al. (2012)<br>You et al. (2015)<br>Wolny et al. (2020)<br>Gottschling et al. (2021a,b)
fortii	N	Y	N	Guillou et al. (2002)<br>Edvardsen et al. (2003)<br>Gómez et al. (2011a)<br>Qiu et al. (2011)<br>Rodríguez et al. (2012)<br>Saburova and Chomérat (2014)<br>Takano et al. (2014)
hastata	Y			Gómez et al. (2011a)
hastata f. phalacromides	YP			Gómez et al. (2011a)
infundibulum	N	Y	Y	Gómez et al. (2011a)<br>Qiu et al. (2011)<br>Rodríguez et al. (2012)<br>Takano et al. (2014)
miles	N	YP	N	Jensen and Daugbjerg (2009)<br>Qiu et al. (2011)
monacantha	YP			Gómez et al. (2011a)
norvegica clade 2	N	Y	N	Rehnstam-Holm et al. (2002)<br>Edvardsen et al. (2003)

				Hansen and Daugbjerg (2004)<br>Murray et al. (2005)<br>Kim and Kim (2007)<br>Litaker et al. (2007)<br>Moestrup et al. (2008)<br>Raho et al. (2008)<br>Sparmann et al. (2008)<br>Handy et al. (2009)<br>Gómez et al. (2011a)<br>Hansen and Daugbjerg (2011)<br>Qiu et al. (2011)<br>Hoppenrath et al. (2012b)<br>Rodríguez et al. (2012)<br>Takano et al. (2014)<br>Baytut et al. (2016)<br>Boutrup et al. (2016)<br>Jang et al. (2016)<br>Jang et al. (2017a,b)<br>Wolny et al. (2020)
odiosa (= Phalacroma odiosum)	NP		N	Handy et al. (2009)<br>Jensen and Daugbjerg (2009)<br>Gómez et al. (2011a)<br>Baytut et al. (2016)
ovum (= D. brevisulcus)	N	N	N	Jensen and Daugbjerg (2009)<br>Hoppenrath et al. (2012a)<br>Qiu et al. (2011)
parvula (= Phalacroma parvulum)	Y		YP	Jensen and Daugbjerg (2009)<br>Gómez et al. (2011a)<br>Baytut et al. (2016)
pusilla	Y		YP	Jensen and Daugbjerg (2009)<br>Gómez et al. (2011a)<br>Park et al (2011)<br>Hoppenrath et al. (2017)
similis			YP	Jensen and Daugbjerg (2009)
sp.<br>clade 4<br>CBC4 L3<br>CBC4 L10	Y	Y	Y	Handy et al. (2009)<br>Wolny et al. (2020)
sp.<br>CBC4 L124	YP	YP	YP	Handy et al. (2009)<br>Qiu et al. (2011)
sp.<br>CBC4 500	YP	YP	YP	Handy et al. (2009)<br>Qiu et al. (2011)
rotundata	YP		YP	Edvardsen et al. (2003)<br>Gómez et al. (2010a,b)<br>Takano et al. (2014)<br>Gómez et al. (2015b)
sacculus (= D. pavillardii)	N	N	N	Edvardsen et al. (2003)

				Handy et al. (2009)<br>Qiu et al. (2011)<br>Rodríguez et al. (2012)
schuettii			YP	Jensen and Daugbjerg (2009)<br>Park et al (2011)
tripos	N	Y	N	Guillou et al. (2002)<br>Edvardsen et al. (2003)<br>Raho et al. (2008)<br>Jensen and Daugbjerg (2009)<br>Gómez et al. (2011a)<br>Hansen and Daugbjerg (2011)<br>Qiu et al. (2011)<br>Reñé et al. (2013)<br>Boutrup et al. (2016)
Dinothrix				
paradoxa<br>2020-HS02 LC583318				Yamada et al. (2020)
phymatodea<br>HG180 LC054946	YP			Yamada et al. (2020)
pseudoparadoxa<br>HG204 LC054947	YP			Yamada et al. (2020)
quadrilobata (= Gymnodinium quadrilobatum)<br>2020-RO01 LC583319	YP			Yamada et al. (2020)
rugata (= Galeidinium rugatum - taxon requires further investigation)<br>HG249 AB195668	YP	YP	YP	Tamura et al. (2005)<br>Gómez et al. (2010b)<br>Alves-De-Souza et al. (2011)<br>Gottschling et al. (2012)<br>Saburova et al. (2012)<br>Saburova and Chomérat (2014)<br>Luo et al. (2015)<br>Gómez et al. (2015b)<br>Horiguchi et al. (2017)<br>Yamada et al. (2017)<br>Žerdoner Čalasan et al. (2019)<br>Yamada et al. (2020)
Diplopelta				
bomba	YP		YP	Hoppenrath et al. (2012b)<br>Mertens et al. (2015b)<br>Gu et al. (2016)<br>Gómez et al. (2019b)<br>Reñé et al. (2019)<br>Gurdebek et al. (2020)
globula			YP	Liu et al. (2015a)<br>Gu et al. (2016)
pusilla (= Lebouraia pusilla)<br>cell 1			Y	Mertens et al. (2015)<br>Gu et al. (2016)

cell 2				Yamaguchi et al. (2016)<br>Efimova et al. (2019)<br>Žerdoner Čalasan et al. (2019)
Diplopsalis				
caspica	YP		YP	Zhang, Q. et al. (2015)
lenticula<br>040619-3 AB716909<br>cell 2<br>M2	YP	Y	Y	Gribble and Anderson (2006)<br>Kim and Kim (2007)<br>Hoppenrath et al. (2012a)<br>Nézan et al. (2012)<br>Potvin et al. (2013)<br>Liu et al. (2015a)<br>Mertens et al. (2015b)<br>Boutrup et al. (2016)<br>Gu et al. (2016)<br>Hoppenrath et al. (2017)<br>Reñé et al. (2019)<br>Žerdoner Čalasan et al. (2019)
Diplopsalopsis				
bomba (= Diplosalis lenticulata, Dissodium asymmetricum, Diplopsalopsis asymmetrica)<br>040913-3 AB716930	YP		YP	Kawami et al. (2006)<br>Gómez et al. (2010a,b)<br>Mertens et al. (2015b)<br>Yamaguchi et al. (2016)<br>Efimova et al. (2019)<br>Reñé et al. (2019)
globula (= Diplopsalopsis sphaerica)			YP	Gurdebek et al. (2020)
ovata<br>cell 2 KP702711			Y	Liu et al. (2015a)<br>Mertens et al. (2015b)<br>Gu et al. (2016)<br>Yamaguchi et al. (2016)<br>Reñé et al. (2019)<br>Gurdebek et al. (2020)
Dissodinium				
pseudolunula<br>FG59<br>IFR992	Y	YP	Y	Kim and Kim (2007)<br>Gomez et al. (2009b)<br>Coats et al. (2010)<br>Gómez and Skovgaard (2014)<br>Nézan et al. (2014)<br>Takano et al. (2014)<br>Gómez et al. (2015b)<br>Gu et al. (2015a)<br>Reñé et al. (2015)<br>Hoppenrath et al. (2017)<br>Li et al. (2017)<br>Na et al. (2017)<br>Gómez et al. (2019a)<br>Romeikat et al. (2019)

				Žerdoner Čalasan et al. (2019)
Duboscquodinium				
collinii<br>VSM11	Y	Y	Y	Coats et al. (2010)<br>Zinssmeister et al. (2011)<br>Craveiro et al. (2013)<br>Gu et al. (2013a)<br>Kim and Park (2014)<br>Kretschmann et al. (2014)<br>Saburova and Chomérat (2014)<br>Craveiro et al. (2015)<br>Gómez and Skovgaard (2015)<br>Gómez et al. (2015a)<br>Kang et al. (2015)<br>You et al. (2015)<br>Kretschmann et al. (2018a,b)<br>Žerdoner Čalasan et al. (2019)<br>Li, Z. et al. (2020)<br>Gottschling et al. (2021a,b)<br>Kim, H.J. et al. (2021)
Duboscquella				
sp.<br>ex Flavella ehrenbergii sp.<br>Ishikari/2003 AB295040	YP			Harada et al. (2007)<br>Skovgaard and Salomonsen (2009)
sp.<br>ex Flavella ehrenbergii sp.<br>Hamana/2003 AB295041	YP			Harada et al. (2007)<br>Gómez et al. (2010b)
Durinskia				
agilis<br>IFR10-453 JF514516	Y	Y	YP	Saburova et al. (2012)<br>Gómez et al. (2015a)<br>Luo et al. (2015)<br>Gómez et al. (2017a)<br>Yamada et al. (2017)<br>Kretschmann et al. (2018b)<br>Saburova and Chomérat (2018)<br>Hoppenrath et al. (2020)<br>Satta et al. (2020)
baltica<br>AF231803<br>HG171 LC054925<br>HG265 LC054926	Y	Y		Tamura et al. (2005)<br>Pienaar et al. (2007)<br>Zhang, Q. et al. (2011a)<br>Saburova et al. (2012)<br>Yamada et al. (2015)<br>Yamada et al. (2017)<br>Kretschmann et al. (2018b)
cf. baltica<br>CS38<br>DBFH2<br>HG171 LC054925	Y	Y		Yamada et al. (2015)<br>Yamada et al. (2017)<br>Kretschmann et al. (2018b)<br>Žerdoner Čalasan et al. (2019)

HG265 LC054926				Satta et al. (2020)
capensis<br>Lamberts Bay AB271107	Y	Y		Pienaar et al. (2007)<br>Gómez et al. (2015b)<br>Yamada et al. (2015)<br>Yamada et al. (2017)<br>Kretschmann et al. (2018b)<br>Satta et al. (2020)
dybowskii (= Durinskia baltica, Peridinium balticum, Peridinium dybowskii, Glenodinium dybowskii)	Y		YP	Hoppenrath and Leander (2007)<br>Alves-De-Souza et al. (2011)<br>Hoppenrath et al. (2012b)<br>Gómez et al. (2015b)<br>Kang et al. (2015)<br>Luo et al. (2015)<br>Mertens et al. (2015b)<br>Gómez et al. (2017a)<br>Saburova and Chomérat (2018)<br>Gómez and Artigas (2019)<br>Gómez et al. (2019b)<br>Reñé et al. (2019)
kwazulunatalensis	Y	Y		Yamada et al. (2017)<br>Kretschmann et al. (2018b)<br>Satta et al. (2020)
oculata<br>GeoM*662<br>I2804<br>K05039	Y	Y	Y	Kretschmann et al. (2018b)<br>Žerdoner Čalasan et al. (2019)<br>Satta et al. (2020)<br>Gottschling et al. (2021a,b)
Durusdinium				
(Symbiodinium clade D temperate species)	YP/YA	Y	Y	Takishita et al. (2003)<br>Siano et al. (2010)<br>Probert et al. (2014)<br>Hehenberger et al. (2017)<br>Jang et al. (2017b)<br>Shi et al. (2020)
boreum (= Symbiodinium borea, Zooxanthella borea)<br>D8 KF740686<br>D13 KF740687<br>D15 KF740688		Y	Y	Jeong et al. (2014b)<br>LaJeunesse et al. (2014)<br>LaJeunesse et al. (2018)
eurythalpos (= Symbiodinium eurythalpos, Zooxanthella eurythalpos)<br>D8<br>D12<br>D13			Y	LaJeunesse et al. (2014)<br>LaJeunesse et al. (2018)
glynnii (= Symbiodinium glynnii, Zooxanthella glynnii)		Y	Y	Wham et al. (2017)<br>LaJeunesse et al. (2018)

trenchii (= Symbiodinium trenchii, Zooxanthella trenchii)<br>D2-D6<br>D1a KF740689		Y	Y	Jeong et al. (2014b)<br>Wham et al. (2017)<br>LaJeunesse et al. (2018)<br>Qin et al. (2019)
sp.<br>DS17A01		Y		Boo et al. (2020)
sp.<br>PSP1-05	YP	YP	YP	Gottschling et al. (2021a,b)
Echinidinium				
karaense<br>EKAR1 KY129813<br>EKAR2 KY129814	Y		Y	Potvin et al. (2018)
Effrenium (Symbiodinium clade E)				
sp.<br>Type E clone E:0-2 AF238262	YP			Jeong et al. (2014a)
sp.<br>Type E clone E:0-3 AF238263	YP			Jeong et al. (2014a)
sp.<br>clade E AB546599		Y		Rodríguez et al. (2019)<br>Boo et al. (2020)
voratum<br>CCMP 421 KF364603<br>MJa-B6-Sy<br>RCC 1521 KF364606<br>rt-383 KF364605<br>SMFL1 HE653238<br>SvFL1<br>SvIC 1<br>TSP-C2-Sy KF364604	YP		Y	Siano et al. (2010)<br>Jeong et al. (2014a,b,c)<br>Lee, S.Y. et al. (2015)<br>Hehenberger et al. (2017)<br>LaJeunesse et al. (2018)<br>Shi et al. (2020)<br>Gottschling et al. (2021a,b)
Ensiculifera				
carinata	YP	Y	YP	Li, Z. et al. (2020)
jinhaensis (= "Pentapharsodinium" jinhaensis)		Y	YP	Li et al. (2015)<br>Žerdoner Čalasan et al. (2019)<br>Li, Z. et al. (2020)
mexicana	Y	Y	Y	Li, Z. et al. (2020)
sp.<br>SSND22 EU728696	YP	YP	YP	Li et al. (2015)<br>Li, Z. et al. (2020)
tyrrhenica (= Pentapharsodinium tyrrhenicum)<br>MUCC097 AF022201	Y	Y	Y	Grzebyk et al. (1998)<br>D'Onofrio et al. (1999)<br>Montresor et al. (2003)<br>Gottschling et al. (2005a)<br>Dolapsakis et al. (2006)<br>Hoppenrath and Leander (2007)<br>Gottschling et al. (2008)<br>Gómez et al. (2010a,b)<br>Hoppenrath et al. (2012b)<br>Craveiro et al. (2013)

				Craveiro et al. (2015)<br>Li et al. (2015)<br>Luo et al. (2015)<br>Mertens et al. (2015b)<br>Yamada et al. (2015)<br>Gómez et al. (2017a)<br>Efimova et al. (2019)<br>Hoppenrath et al. (2020)<br>Li, Z. et al. (2020)<br>Satta et al. (2020)<br>Gottschling et al. (2021a)<br>Kim, H.J. et al. (2021)
Erythropsidinium				
agile<br>FG117	YP	YP	YP	Gomez et al. (2009a,b)<br>Reñé et al. (2015)<br>Žerdoner Čalasan et al. (2019)
Esoptrodinium				
gemma (= Bernardinium bernardinense)			YP	Calado et al. (2006)<br>Moestrup et al. (2008)<br>Hansen and Daugbjerg (2011)<br>Fawcett and Parrow (2012)<br>Takahashi et al. (2015)<br>Boutrup et al. (2016)<br>Luo et al. (2016b)<br>Boutrup et al. (2017)<br>Pandeirada et al. (2017)<br>Takahashi et al. (2017)
sp. A<br>CCP1 JQ439938<br>CCP2<br>RP<br>UNCC	YP	YP	YP	Fawcett and Parrow (2012)<br>Anglès et al. (2017)<br>Gottschling et al. (2021a,b)
sp. B<br>PPT			YP	Fawcett and Parrow (2012)
sp. D<br>HP JQ439943	YP	YP	YP	Fawcett and Parrow (2012)<br>Anglès et al. (2017)
Euduboscquella				
cachonii sp. clade C<br>sp. ex Eutintinus tenuis<br>SERC1 JN934988<br>SERC12 JN934987	YP			Bachvaroff, et al. (2012)<br>Kim and Park (2014)<br>Jung et al. (2015)<br>Jung et al. (2018)<br>Wakeman et al. (2018b)
costata sp. clade D<br>ex Favella arculata KP749831<br>ex Schmidingerella arcuata<br>JN934989	Y			Bachvaroff, et al. (2012)<br>Jung et al. (2015)<br>Gómez and Gast (2018)<br>Jung et al. (2018)
crenulata sp. clade A	Y			Bachvaroff, et al. (2012)

ex Favella erenbergii<br>Hamana/2003 AB295041<br>ex Favella panamensis<br>JN606065<br>ex Tintinnipsis sp. JN934984<br>ex Tintinnopsis cf. subacuta<br>JN934992				Kim and Park (2014)<br>Jung et al. (2015)<br>Gómez and Gast (2018)<br>Jung et al. (2018)
sp. clade B<br>ex Favella erenbergii<br>Ishikari/2003 AB295040<br>sp. Favella markusovszkyi<br>JN934985<br>sp. Favella panamensis<br>JN934986	Y			Bachvaroff, et al. (2012)<br>Kim and Park (2014)<br>Jung et al. (2015)<br>Gómez and Gast (2018)<br>Jung et al. (2018)
sp. clade E<br>JP12-1 MG209087	YP			Jung et al. (2018)
sp. clade F<br>JP12-4-1 MG209098	YP			Jung et al. (2018)
sp. clade G<br>JP12-4-5 MG209094	Y			Jung et al. (2018)
Fensomea				
setacea	Y	Y	Y	Gottschling et al. (2021a)
Fragilidium				
duplocampanaeforme<br>Fd-LOHABE01	YA		Y	Kim and Park (2017)<br>Gómez and Artigas (2019)<br>Li and Shin (2019)<br>Selina and Efimova (2020)<br>Gottschling et al. (2021b)
cf. duplocampanaeforme			Y	Amorim et al. (2013)<br>Kim and Park (2017)
fissile			YP	Kim and Park (2017)
mexicanum	Y		Y	Li and Shin (2019)
sp.<br>G02 FJ405356	YP			Li and Shin (2019)
sp.<br>FIU49 EU165297			YP	Amorim et al. (2013)
subglobosum	YA		Y	Leaw et al. 2005<br>Murray et al. (2005)<br>Dolapsakis et al. (2006)<br>Hoppenrath and Leander (2007)<br>Kim and Kim (2007)<br>Amorim et al. (2013)<br>Saburova and Chomérat (2014)<br>Gómez et al. (2017a)<br>Kim and Park (2017)<br>Li and Shin (2019)
Fugacium (Symbiodinium clade F)				

kawagutii (clade Fr5)<br>135cp AF333517<br>clade_C AF427462<br>NCMA2468 LK934666	YP	Y	Y	LaJeunesse (2001)<br>Jeong et al. (2014b)<br>LaJeunesse et al. (2018)<br>Rodríguez et al. (2019)<br>Teschima et al. (2019)<br>Shi et al. (2020)<br>Gottschling et al. (2021a,b)<br>Macher et al. (2021)
sp. clade Fr2<br>133c		Y		LaJeunesse (2001)<br>Jeong et al. (2014b)<br>Shi et al. (2020)
sp.<br>CS-156 AB016594	YP			Hehenberger et al. (2017)
sp.<br>NCMA2455	YP			Gottschling et al. (2021b)
Fukuyoa				
paulensis	Y		Y	Gómez et al. (2015c)<br>Shin et al. (2016)<br>Smith et al. (2016)<br>Kretschmann et al. (2017)<br>Rhodes et al. (2017)<br>Leung et al. (2018)<br>Gómez and Artigas (2019)<br>Kretzschmar (2019)<br>Reñé and Hoppenrath (2019)
ruetzleri (= Gambierdiscus ruetzleri) (Though genetic clusters are distinct, Leung et al. 2018 argue that genetic distance between F. ruetzleri and G. yasumotoi is too small to be different species given overlap in their morphological characteristics)<br>SKLMP_S044	Y		Y	Litaker et al. (2009)<br>Gómez et al. (2015c)<br>Shin et al. (2016)<br>Smith et al. (2016)<br>Kretschmann et al. (2017)<br>Rodríguez et al. (2017)<br>Rhodes et al. (2017)<br>Kretzschmar (2019)<br>Reñé and Hoppenrath (2019)<br>Selina and Efimova (2020)<br>Gottschling et al. (2021b)
sp.<br>DQ-2015	YP			Gottschling et al. (2021b)
sp.<br>HK Type 1	YP		YP	Leung et al. (2018)
yasumotoi (= Gambierdiscus yasumotoi)	YA		Y	Litaker et al. (2009)<br>Gómez et al. (2010a,b)<br>Murray et al. (2014b)<br>Gómez et al. (2015c)<br>Smith et al. (2016)<br>Kretschmann et al. (2017)<br>Rhodes et al. (2017)

				Leung et al. (2018)<br>Hoppenrath et al. (2019)<br>Kretzschmar (2019)<br>Reñé and Hoppenrath (2019)<br>Selina and Efimova (2020)
Fusiperidinium				
wisconsinense (= Peridinium wisconsinense, taxon requires further investigation)	YP	YP	YP	Luo, Z. et al. (2016a)<br>McCarthy et al. (2018)<br>Luo et al. (2018a)<br>Luo et al. (2019)<br>Žerdoner Čalasan et al. (2019)<br>Hoppenrath et al. (2020)<br>Li, Z. et al. (2020)<br>Luo, Z. et al. (2020)<br>Gottschling et al. (2021b)<br>Kim, H.J. et al. (2021)
Galeidinium				
sp.<br>HG180 LC054946	YP	YP		Kretschmann et al. (2018b)<br>Satta et al. (2020)
sp.<br>HG204 LC054947	YP	YP		Kretschmann et al. (2018b)<br>Satta et al. (2020)
Gambierdiscus				
australes	Y		Y	Chinain et al. (1999)<br>Litaker et al. (2009)<br>Fraga et al. (2011)<br>Fraga and Rodríguez (2014)<br>Gómez et al. (2015c)<br>Fraga et al. (2016)<br>Shin et al. (2016)<br>Smith et al. (2016)<br>Kim and Park (2017)<br>Kretschmann et al. (2017)<br>Rhodes et al. (2017)<br>Rodríguez et al. (2017)<br>Larsson et al. (2018)<br>Efimova et al. (2019)<br>Hoppenrath et al. (2019)<br>Kretzschmar (2019)<br>Reñé and Hoppenrath (2019)
balechii	Y			Rhodes et al. (2017)<br>Jang et al. (2018)<br>Hoppenrath et al. (2019)
belizeanus	Y	YP	Y	Litaker et al. (2009)<br>Fraga et al. (2011)<br>Gómez et al. (2015c)<br>Fraga et al. (2016)<br>Shin et al. (2016)

				Smith et al. (2016)<br>Kretschmann et al. (2017)<br>Rhodes et al. (2017)<br>Rodríguez et al. (2017)<br>Larsson et al. (2018)<br>Kretzschmar (2019)<br>Li et al. (2019)<br>Reñé and Hoppenrath (2019)
caribaeus	Y	YP	Y	Litaker et al. (2009)<br>Fraga et al. (2011)<br>Fraga and Rodríguez (2014)<br>Gómez et al. (2015c)<br>Fraga et al. (2016)<br>Smith et al. (2016)<br>Kretschmann et al. (2017)<br>Rodríguez et al. (2017)<br>Rhodes et al. (2017)<br>Larsson et al. (2018)<br>Hoppenrath et al. (2019)<br>Kretzschmar (2019)<br>Reñé and Hoppenrath (2019)
carolinianus	Y	YP	Y	Litaker et al. (2009)<br>Fraga et al. (2011)<br>Fraga and Rodríguez (2014)<br>Gómez et al. (2015c)<br>Fraga et al. (2016)<br>Shin et al. (2016)<br>Smith et al. (2016)<br>Kretschmann et al. (2017)<br>Rodríguez et al. (2017)<br>Rhodes et al. (2017)<br>Jang et al. (2018)<br>Larsson et al. (2018)<br>Hoppenrath et al. (2019)<br>Li et al. (2019)<br>Reñé and Hoppenrath (2019)<br>Gottschling et al. (2021b)
carpenteri	Y		Y	Litaker et al. (2009)<br>Fraga et al. (2011)<br>Fraga and Rodríguez (2014)<br>Gómez et al. (2015c)<br>Fraga et al. (2016)<br>Smith et al. (2016)<br>Kretschmann et al. (2017)<br>Rhodes et al. (2017)<br>Rodríguez et al. (2017)<br>Jang et al. (2018)<br>Larsson et al. (2018)

				Hoppenrath et al. (2019)<br>Kretzschmar (2019)<br>Reñé and Hoppenrath (2019)
cheloniae	Y		Y	Smith et al. (2016)<br>Jang et al. (2018)<br>Larsson et al. (2018)<br>Hoppenrath et al. (2019)<br>Kretzschmar (2019)<br>Rhodes et al. (2017)
excentricus	Y		Y	Fraga et al. (2011)<br>Fraga and Rodríguez (2014)<br>Smith et al. (2016)<br>Rhodes et al. (2017)<br>Rodríguez et al. (2017)<br>Jang et al. (2018)<br>Larsson et al. (2018)<br>Hoppenrath et al. (2019)<br>Kretzschmar (2019)<br>Reñé and Hoppenrath (2019)<br>Selina and Efimova (2020)
holmesii	Y		Y	Kretzschmar (2019)
honu	YP		Y	Rhodes et al. (2017)<br>Larsson et al. (2018)<br>Hoppenrath et al. (2019)<br>Kretzschmar (2019)
jejuensis	Y		Y	Jang et al. (2018)
lapillus	Y			Rhodes et al. (2017)<br>Hoppenrath et al. (2019)
lewisii<br>UTSHI6A6	YP		YP	Larsson et al. (2018)<br>Kretzschmar (2019)
pacificus	Y		Y	Chinain et al. (1999)<br>Litaker et al. (2009)<br>Fraga et al. (2011)<br>Fraga and Rodríguez (2014)<br>Gómez et al. (2015c)<br>Fraga et al. (2016)<br>Smith et al. (2016)<br>Kretschmann et al. (2017)<br>Rhodes et al. (2017)<br>Jang et al. (2018)<br>Larsson et al. (2018)<br>Hoppenrath et al. (2019)<br>Kretzschmar (2019)<br>Reñé and Hoppenrath (2019)
polynesiensis	Y		Y	Chinain et al. (1999)<br>Litaker et al. (2009)<br>Amorim et al. (2013)

				Fraga and Rodríguez (2014)<br>Gómez et al. (2015c)<br>Fraga et al. (2016)<br>Shin et al. (2016)<br>Smith et al. (2016)<br>Kim and Park (2017)<br>Kretschmann et al. (2017)<br>Rhodes et al. (2017)<br>Rodríguez et al. (2017)<br>Jang et al. (2018)<br>Larsson et al. (2018)<br>Efimova et al. (2019)<br>Hoppenrath et al. (2019)<br>Kretzschmar (2019)<br>Reñé and Hoppenrath (2019)
scabrosus	Y		YP	Nishimura et al. (2014)<br>Gómez et al. (2015c)<br>Shin et al. (2016)<br>Smith et al. (2016)<br>Kretschmann et al. (2017)<br>Rodríguez et al. (2017)<br>Jang et al. (2018)<br>Larsson et al. (2018)<br>Hoppenrath et al. (2019)<br>Kretzschmar (2019)<br>Reñé and Hoppenrath (2019)
silvae	YP		Y	Fraga and Rodríguez (2014)<br>Rhodes et al. (2017)<br>Rodríguez et al. (2017)<br>Jang et al. (2018)<br>Larsson et al. (2018)<br>Hoppenrath et al. (2019)<br>Kretzschmar (2019)
scabrosus	Y		Y	Fraga and Rodríguez (2014)<br>Fraga et al. (2016)<br>Kretschmann et al. (2017)<br>Rodríguez et al. (2017)<br>Jang et al. (2018)<br>Kretzschmar (2019)
sp. ribotype 2	Y		Y	Litaker et al. (2009)<br>Fraga et al. (2011)<br>Fraga and Rodríguez (2014)<br>Fraga et al. (2016)<br>Smith et al. (2016)<br>Kretschmann et al. (2017)<br>Jang et al. (2018)<br>Larsson et al. (2018)<br>Kretzschmar (2019)

				Hoppenrath et al. (2019)
sp. type 2	Y		Y	Fraga et al. (2016)<br>Smith et al. (2016)<br>Kretschmann et al. (2017)<br>Rhodes et al. (2017)<br>Rodríguez et al. (2017)<br>Larsson et al. (2018)<br>Hoppenrath et al. (2019)
sp. type 3	Y		Y	Fraga et al. (2016)<br>Smith et al. (2016)<br>Kretschmann et al. (2017)<br>Rhodes et al. (2017)<br>Jang et al. (2018)<br>Hoppenrath et al. (2019)
toxicus	Y		Y	Litaker et al. (2009)<br>Gómez et al. (2010a)<br>Fraga et al. (2011)<br>Fraga and Rodríguez (2014)<br>Gómez et al. (2015c)<br>Shin et al. (2016)<br>Smith et al. (2016)<br>Kretschmann et al. (2017)<br>Rhodes et al. (2017)<br>Rodríguez et al. (2017)<br>Jang et al. (2018)<br>Larsson et al. (2018)<br>Efimova et al. (2019)<br>Hoppenrath et al. (2019)<br>Kretzschmar (2019)<br>Reñé and Hoppenrath (2019)
Gerakladium (Symbiodinium Clade G temperate species)				
endoclionum<br>OI16_orient6 MF322789<br>JP16_27_6 MF322790<br>JP16_31 23S MF322793	YA		Y	Siano et al. (2010)<br>Ramsby et al. (2017)<br>LaJeunesse et al (2018)<br>Shi et al. (2020)
spongiolum<br>Floridakeys17_3 MF322787<br>Stthom17_01 MF322788	YA		Y	Siano et al. (2010)<br>LaJeunesse et al (2018)<br>Shi et al. (2020)
Gertia				
stigmatica<br>mdd472-kt LC490696	YP	YP	YP	Takahashi et al. (2019)<br>Benico et al. (2020)<br>Ok et al. (2020)
Glenoaulax				
inaequalis (= Glenodinium inaequale)	YP			Saburova and Chomérat (2014)
Glenodiniopsis				

steinii<br>NIES463 EF058255<br>NIES463 EF274257	YP	YP	YP	Gottschling et al. (2021a,b)
Glenodinium				
hallii (= Cachonina hallii)	Y		YA	Nézan et al. (2012)<br>Kim and Park (2014)
Gloeodinium				
montanum<br>CCAC0066	YP	YP	YP	Hansen et al. (2018)<br>Wakeman et al. (2018a)<br>Žerdoner Čalasan et al. (2019)<br>Gottschling et al. (2021a,b)
viscum	YP			McNally et al. (1994)<br>Grzebyk et al. (1998)<br>Gómez et al. (2015a)<br>Wakeman et al. (2018a)
Goniodoma				
polyedricum			Y	Nézan et al. (2012)<br>Gómez et al. (2015)<br>Boutrup et al. (2016)<br>Kim and Park (2017)
Gonyaulax				
baltica			Y	Ellegaard et al. (2003)<br>Hansen and Daugbjerg (2004)<br>Dolapsakis et al. (2006)<br>Gribble and Anderson (2006)<br>Kim and Kim (2007)<br>Moestrup et al. (2008)<br>Howard et al. (2009)<br>Mertens et al. (2012)<br>Mertens et al. (2015b)<br>Boutrup et al. (2016)<br>Luo et al. (2016b)<br>Mertens et al. (2017a)<br>Ellegaard et al. (2018)<br>Efimova et al. (2019)
cochlea	YP			Gómez et al. (2010a)<br>Zhang et al. (2020)
digitalis (= Protoperidinium digitale)			YP	Ellegaard et al. (2003)<br>Hansen and Daugbjerg (2004)<br>Kim and Kim (2007)<br>Howard et al. (2009)<br>Saburova and Chomérat (2014)<br>Hoppenrath et al. (2017)<br>Kim and Park (2017)<br>Ellegaard et al. (2018)<br>Efimova et al. (2019)<br>Selina and Efimova (2020)

				Zhang et al. (2020)
elongata (= Spiniferites elongates)<br>cyst 6	YP	YP	YP	Ellegaard et al. (2003)<br>Hansen and Daugbjerg (2004)<br>Howard et al. (2009)<br>Ellegaard et al. (2018)<br>Efimova et al. (2019)<br>Selina and Efimova (2020)<br>Zhang et al. (2020)<br>Gottschling et al. (2021a,b)
fragilis	YP		Y/YA	Gómez et al. (2010a,b)<br>Efimova et al. (2019)<br>Zhang et al. (2020)
membranacea / cf. membranacea (= Spiniferites membranaceus)	YP	YP	Y	Ellegaard et al. (2003)<br>Kim and Kim (2007)<br>Howard et al. (2009)<br>Ellegaard et al. (2018)<br>Efimova et al. (2019)<br>Zhang et al. (2020)<br>Gottschling et al. (2021a)
polygramma	YP		YP/YA	Howard et al. (2009)<br>Gómez et al. (2010a,b)<br>Kim and Park (2014)<br>Li et al. (2017)<br>Gómez and Artigas (2019)<br>Zhang et al. (2020)
spinifera (= Spiniferites ramosus)<br>CCMP409 AF022155<br>KC51	YP	YP	Y/YA	Grzebyk et al. (1998)<br>Ellegaard et al. (2003)<br>Murray et al. (2005)<br>Dolapsakis et al. (2006)<br>Rogers et al. (2006)<br>Hoppenrath and Leander (2007)<br>Penna et al. (2008)<br>Howard et al. (2009)<br>Gómez et al. (2010a,b)<br>Hoppenrath et al. (2012b)<br>Yamada et al. (2015)<br>Kim and Park (2017)<br>Li et al. (2017)<br>Mertens et al. (2017a)<br>Gu et al. (2018)<br>Salgado et al. (2018)<br>Efimova et al. (2019)<br>Gómez and Artigas (2019)<br>Selina and Efimova (2020)<br>Zhang et al. (2020)<br>Gottschling et al. (2021a)
cf. spinifera			Y	Ellegaard et al. (2003)

				Gribble and Anderson (2006)<br>Ellegaard et al. (2018)
whaseongensis	YP		YP	Zhang et al. (2020)
Gotoius				
abei				
excentricus (= Diplopsalis excentrica, Dissodium excentricum)<br>040603-11 AB716923	YP		YP	Kawami et al. (2006)<br>Gómez et al. (2010a,b)<br>Liu et al. (2015a)<br>Mertens et al. (2015b)<br>Gu et al. (2016)<br>Yamaguchi et al. (2016)<br>Reñé et al. (2019)<br>Gurdebek et al. (2020)
ostenfeldii (= Diplopsalis ostenfeldii)			YP	Liu et al. (2015a)
Grammatodinium				
tongyeonginum<br>LIM-PS2334	YP		YP	Li et al. (2017)<br>Shin et al. (2019)<br>Selina and Efimova (2020)<br>Gottschling et al. (2021b)
Gymnodiniaceae unidentified species				
sp. isolated from Spongotrochus glacialis<br>AB860180	YP	YP	YP	Romeikat et al. (2019)
sp. KJ56-0.2-19	YP	YP	YP	Romeikat et al. (2019)
sp. KJ47-3-57	YP	YP	YP	Romeikat et al. (2019)
sp. KJ22-3-19	YP	YP	YP	Romeikat et al. (2019)
sp. KJ22-3-58	YP	YP	YP	Romeikat et al. (2019)
sp. N420T_95	YP	YP	YP	Žerdoner Čalasan et al. (2019)
sp. HBI:HB201110a (QZ-2012)	YP	YP	YP	Žerdoner Čalasan et al. (2019)
sp. W8eB2	YP			Gottschling et al. (2021b)
Gymnodinium				
aureolum<br>MUCC284 AF022196<br>SWA 16 AY999082<br>GeoB 232 KJ481834<br>IFR10-059 KJ508392<br>GASMK0803 FN392226	Y	Y	Y	Daugbjerg et al. (2000)<br>Hansen et al. (2000)<br>de Salas et al. (2005b)<br>Kim and Kim (2007)<br>Murray et al. (2007b)<br>Gómez et al. (2010a,b)<br>Reñé et al. (2011)<br>Sampedro et al. (2011)<br>Gottschling et al. (2012)<br>Gu et al. (2013e)<br>Lee, K.H. et al. (2013)<br>Qiu et al. (2013)<br>Gómez and Skovgaard (2014)<br>Nézan et al. (2014)<br>Takano et al. (2014)

				Kretschmann et al. (2015)<br>Reñé et al. (2015)<br>Na et al. (2017)<br>Annenkova (2018)<br>Gu et al. (2018)<br>Luo et al. (2018a,b)<br>Wakeman et al. (2018a)<br>Efimova et al. (2019)<br>Gómez et al. (2019a)<br>Li et al. (2019)<br>Romeikat et al. (2019)<br>Tillman et al. (2020)<br>Gottschling et al. (2021a,b)
baicalense<br>LA084 (NA-2008)<br>LA0810 (NA-2008)<br>LM081 (NA-2008)<br>LM086 (NA-2008)<br>LM0802 (NA-2008)	Y	YP	Y	Gómez and Skovgaard (2014)<br>Na et al. (2017)<br>Annenkova (2018)<br>Romeikat et al. (2019)<br>Žerdoner Čalasan et al. (2019)<br>Gottschling et al. (2021b)
catenatum<br>GnCt01	Y	Y	Y	Zardoya et al. (1995)<br>Adachi et al. (1997)<br>Grzebyk et al. (1998)<br>Hansen et al. (2000)<br>Hansen and Daugbjerg (2004)<br>Kim, S.H. et al. (2004)<br>Ordás et al. (2004)<br>Bergholtz et al. (2005)<br>Lindberg et al. (2005)<br>Murray et al. (2005)<br>Kawami et al. (2006)<br>Hoppenrath and Leander (2007)<br>Iwataki et al. (2007)<br>Kim and Kim (2007)<br>Litaker et al. (2007)<br>Murray et al. (2007a,b)<br>Moestrup et al. (2008)<br>Sparmann et al. (2008)<br>Sampedro et al. (2011)<br>Gottschling et al. (2012)<br>Hoppenrath et al. (2012b)<br>Yoon et al. (2012)<br>Gu et al. (2013e)<br>Qiu et al. (2013)<br>Reñé et al. (2013)<br>Saburova and Chomérat (2014)<br>Takano et al. (2014)<br>Gómez et al. (2015a)<br>Kretschmann et al. (2015)

				Reñé et al. (2015)<br>Yamada et al. (2015)<br>Gómez et al. (2017a)<br>Na et al. (2017)<br>Annenkova (2018)<br>Luo et al. (2018a,b)<br>Efimova et al. (2019)<br>Gómez et al. (2019a)<br>Romeikat et al. (2019)<br>Žerdoner Čalasan et al. (2019)<br>Shin et al. (2019)<br>Gottschling et al. (2021a,b)
corii		YA/YP	YA	Guillou et al. (2002)<br>Shao et al. (2004)<br>Litaker et al. (2007)<br>Fodor (2014)
corollarium<br>K-0983	Y	YP	Y	Gu et al. (2013e)<br>Nézan et al. (2014)<br>Na et al. (2017)<br>Luo et al. (2018b)<br>Romeikat et al. (2019)
dominans	YP	YP	YP	Gottschling et al. (2012)<br>Reñé et al. (2015)<br>Luo et al. (2016b)
dorsalisulcum<br>HG154 LC054930<br>KDAAD DQ837534<br>SM28	Y	Y	Y	Murray et al. (2007b)<br>Gómez et al. (2009a)<br>Gómez et al. (2010a,b)<br>Reñé et al. (2011)<br>Sampedro et al. (2011)<br>Hoppenrath et al. (2012a)<br>Gu et al. (2013e)<br>Qiu et al. (2013)<br>Gómez and Skovgaard (2014)<br>Nézan et al. (2014)<br>Gómez et al. (2015b)<br>Reñé et al. (2015)<br>Yamada et al. (2015)<br>Boutrup et al. (2016)<br>Na et al. (2017)<br>Annenkova (2018)<br>Luo et al. (2018b)<br>Efimova et al. (2019)<br>Gómez et al. (2019a)<br>Romeikat et al. (2019)
eucyaneum	YP			Xia et al. (2013)<br>Takano et al. (2014)
fuscum	Y	YP	Y	Grzebyk et al. (1998)

CCMP1677 AF200676<br>LO2281-09<br>MUCC282D AF022194				Hansen et al. (2000)<br>Hansen and Daugbjerg (2004)<br>Bergholtz et al. (2005)<br>Lindberg et al. (2005)<br>Murray et al. (2005)<br>Kawami et al. (2006)<br>Kim and Kim (2007)<br>Murray et al. (2007b)<br>Moestrup et al. (2008)<br>Sparmann et al. (2008)<br>Gómez et al. (2010a,b)<br>Sampedro et al. (2011)<br>Hoppenrath et al. (2012b)<br>Gu et al. (2013e)<br>Reñé et al. (2013)<br>Gómez and Skovgaard (2014)<br>Takano et al. (2014)<br>Mertens et al. (2015b)<br>Reñé et al. (2015)<br>Gómez et al. (2017a)<br>Hoppenrath et al. (2017)<br>Horiguchi et al. (2017)<br>Na et al. (2017)<br>Annenkova (2018)<br>Efimova et al. (2019)<br>Gómez et al. (2019a)<br>Romeikat et al. (2019)<br>Shin et al. (2019)<br>Žerdoner Čalasan et al. (2019)<br>Gottschling et al. (2021a,b)
heterogrammum	Y			Reñé et al. (2015)
"impatiens"<br>CCAV0025	YP	YP	YP	Gottschling et al. (2021a)
impudicum<br>MUCC276D AF022197<br>Gi-1cp	Y	Y	Y	Grzebyk et al. (1998)<br>Daugbjerg et al. (2000)<br>Hansen et al. (2000)<br>Hansen and Daugbjerg (2004)<br>Lindberg et al. (2005)<br>Hoppenrath and Leander (2007)<br>Murray et al. (2007b)<br>Moestrup et al. (2008)<br>Sparmann et al. (2008)<br>Reñé et al. (2011)<br>Sampedro et al. (2011)<br>Gottschling et al. (2012)<br>Hoppenrath et al. (2012a)<br>Yoon et al. (2012)<br>Qiu et al. (2013)

				Reñé et al. (2013)<br>Nézan et al. (2014)<br>Takano et al. (2014)<br>Kretschmann et al. (2015)<br>Reñé et al. (2015)<br>Yamada et al. (2015)<br>Hoppenrath et al. (2017)<br>Annenkova (2018)<br>Gu et al. (2018)<br>Gómez et al. (2019a)<br>Romeikat et al. (2019)<br>Žerdoner Čalasan et al. (2019)<br>Shin et al. (2019)<br>Žerdoner Čalasan et al. (2019)<br>Gottschling et al. (2021a,b)
inusitatum			Y	Gu et al. (2013e)<br>Romeikat et al. (2019)
limneticum	YP			Efimova et al. (2019)
litoralis<br>TLCA12062007 KT359532	YP	YP	Y	Reñé et al. (2011)<br>Gu et al. (2013e)<br>Reñé et al. (2013)<br>Nézan et al. (2014)<br>Reñé et al. (2015)<br>Na et al. (2017)<br>Luo et al. (2018b)<br>Gómez et al. (2019a)<br>Reñé et al. (2019)<br>Romeikat et al. (2019)<br>Shin et al. (2019)<br>Žerdoner Čalasan et al. (2019)<br>Gottschling et al. (2021b)
maguelonnense		Y		Shao et al. (2004)
microreticulatum	Y	YP	Y	Reñé et al. (2011)<br>Sampedro et al. (2011)<br>Gu et al. (2013e)<br>Takano et al. (2014)<br>Reñé et al. (2015)<br>Na et al. (2017)<br>Romeikat et al. (2019)
nolleri<br>VGO9221	Y		Y	Daugbjerg et al. (2000)<br>Hansen et al. (2000)<br>Hansen and Daugbjerg (2004)<br>Bergholtz et al. (2005)<br>Kim and Kim (2007)<br>Murray et al. (2007b)<br>Moestrup et al. (2008)<br>Reñé et al. (2011)

				Sampedro et al. (2011)<br>Gu et al. (2013e)<br>Takano et al. (2014)<br>Boutrup et al. (2016)<br>Luo et al. (2018b)<br>Romeikat et al. (2019)<br>Žerdoner Čalasan et al. (2019)<br>Ok et al. (2020)
cf. nolleri	YP	YP	YP	Na et al. (2017)<br>Gómez et al. (2019a)
plasticum	Y	Y	Y	Gu et al. (2013e)<br>Na et al. (2017)<br>Gómez et al. (2019a)<br>Romeikat et al. (2019)<br>Gottschling et al. (2021a,b)
cf placidum<br>K-0308	YP	YP	YP	Daugbjerg et al. (2000)<br>Kretschmann et al. (2015)<br>Žerdoner Čalasan et al. (2019)
palustre (= Spiniferodinium palustre)<br>AJC14-732<br>GeoM*719	Y	YP	Y	Daugbjerg et al. (2000)<br>Murray et al. (2007b)<br>Reñé et al. (2011)<br>Gu et al. (2013e)<br>Takano et al. (2014)<br>Liu et al. (2015a)<br>Reñé et al. (2015)<br>Annenkova (2018)<br>Efimova et al. (2019)<br>Gómez et al. (2019a)<br>Žerdoner Čalasan et al. (2019)<br>Gottschling et al. (2021a)
pygmaeum			YA	Fodor (2014)
smaydae<br>GSSW10	YP	YP	YP	Kretschmann et al. (2015)<br>Boutrup et al. (2016)<br>Na et al. (2017)<br>Luo et al. (2018a,b)<br>Romeikat et al. (2019)<br>Žerdoner Čalasan et al. (2019)<br>Tillman et al. (2020)<br>Gottschling et al. (2021b)
sp.<br>ZX28-3-45 Isolated from Spongotrochus glacialis KT390099	YP	YP	YP	Romeikat et al. (2019)
sp.<br>Isolated from Spongotrochus glacialis AB860180	YP	YP	YP	Romeikat et al. (2019)
sp.	YP			Gómez et al. (2019a)

QZ-2012 JQ639761				
sp.<br>Corsica_2 AF318248	YP			Guillou et al. (2002)
sp. 1<br>Isolate AR86 KP790186<br>Isolate AR199 KP790188<br>Isolate AR344 KP790178	YP	YP	Y	Reñé et al. (2013)<br>Reñé et al. (2015)<br>Na et al. (2017)<br>Luo et al. (2018b)
sp. 2<br>Isolate 2 KF245463<br>Isolate AR251 KP790189<br>Isolate AR252 KP790190	YP	YP	Y	Reñé et al. (2013)<br>Reñé et al. (2015)<br>Na et al. (2017)<br>Luo et al. (2018b)<br>Shin et al. (2019)
sp.<br>NVA/RUS/2008 clone 7<br>NVA/RUS/2008 clone 44	Y	Y	Y	Romeikat et al. (2019)
trapeziforme			YP	Reñé et al. (2011)<br>Gu et al. (2013e)<br>Reñé et al. (2015)
varians<br>CCMP421 AF060899			YP	Guillou et al. (2002)
venator (= Gymnodinium pellucidum)	YP		YP	Murray et al. (2005)<br>Gribble and Anderson (2006)<br>Iwataki et al. (2007)<br>Murray et al. (2007b)<br>Hoppenrath et al. (2012a)<br>Gu et al. (2013e)<br>Takano et al. (2014)<br>Reñé et al. (2015)
Gymnoxanthella				
radiolariae	Y		Y	Yuasa et al. (2016)<br>Gómez et al. (2017a)<br>Luo et al. (2018b)<br>Gómez et al. (2019a)
sp.	YP	YP	YP	Gottschling et al. (2021a)
Gyrodiniellum				
shiwhaense	YP	YP	YP	Gómez and Skovgaard (2014)<br>Jeong et al. (2014c)<br>Gu et al. (2015a)<br>Kretschmann et al. (2015)<br>Na et al. (2017)<br>Luo et al. (2018b)<br>Gómez et al. (2019a)<br>Romeikat et al. (2019)<br>Žerdoner Čalasan et al. (2019)<br>Gottschling et al. (2021a,b)
sp.	YP		YP	Romeikat et al. (2019)

DH114_3A10				
Gyrodinium				
britannia			YP	Reñé et al. (2015)<br>Annenkova (2018)
corallinum			YP	Reñé et al. (2015)<br>Annenkova (2018)
dominans	YP	YP	Y	Hansen and Daugbjerg (2004)<br>Lindberg et al. (2005)<br>Iwataki et al. (2007)<br>Kim and Kim (2007)<br>Moestrup et al. (2008)<br>Hansen and Daugbjerg (2011)<br>Hoppenrath et al. (2012a)<br>Yoon et al. (2012)<br>Reñé et al. (2013)<br>Nézan et al. (2014)<br>Reñé et al. (2015)<br>Boutrup et al. (2016)<br>Luo et al. (2016b)<br>Annenkova (2018)<br>Efimova et al. (2019)<br>Shin et al. (2019)<br>Ok et al. (2020)<br>Gottschling et al. (2021a)
dorsum	YP			Sparmann et al. (2008)<br>Hoppenrath et al. (2012a,b)
fusiforme	YP		YP	Takano and Horiguchi (2004)<br>Sparmann et al. (2008)<br>Hoppenrath et al. (2012b)<br>Yoon et al. (2012)<br>Takano et al. (2014)<br>Gómez et al. (2017a)<br>Annenkova (2018)<br>Gómez et al. (2019a)<br>Shin et al. (2019)
gutrula	YP			Annenkova (2018)
helveticum	YP	YP	Y	Takano and Horiguchi (2004)<br>Hoppenrath et al. (2012a)<br>Yoon et al. (2012)<br>Reñé et al. (2015)<br>Pinto et al. (2017)<br>Annenkova (2018)<br>Gottschling et al. (2021a)
heterogrammum (sp. 3)	YP		Y	Reñé et al. (2015)<br>Annenkova (2018)
moestrupii	YP	YP	YP	Yoon et al. (2012)<br>Nézan et al. (2014)

				Reñé et al. (2015)<br>Annenkova (2018)
cf. ochraceum			Y	Reñé et al. (2015)<br>Annenkova (2018)
rubrum	YP		YP	Hansen and Daugbjerg (2004)<br>Takano and Horiguchi (2004)<br>Lindberg et al. (2005)<br>Iwataki et al. (2007)<br>Kim and Kim (2007)<br>Moestrup et al. (2008)<br>Hansen and Daugbjerg (2011)<br>Hoppenrath et al. (2012a)<br>Yoon et al. (2012)<br>Qiu et al. (2013)<br>Reñé et al. (2013)<br>Nézan et al. (2014)<br>Saburova and Chomérat (2014)<br>Takano et al. (2014)<br>Reñé et al. (2015)<br>Boutrup et al. (2016)<br>Luo et al. (2016b)<br>Annenkova (2018)<br>Shin et al. (2019)<br>Ok et al. (2020)
sp. 1			YP	Reñé et al. (2015)<br>Annenkova (2018)
sp. 2			YP	Reñé et al. (2015)
sp. 4			Y	Reñé et al. (2015)<br>Annenkova (2018)
sp. 5			YP	Reñé et al. (2015)
spirale (= Gymnodinium spirale)	N/YA/Y		Y	Hansen and Daugbjerg (2004)<br>Takano and Horiguchi (2004)<br>Lindberg et al. (2005)<br>Iwataki et al. (2007)<br>Kim and Kim (2007)<br>Moestrup et al. (2008)<br>Sparmann et al. (2008)<br>Hansen and Daugbjerg (2011)<br>Sampedro et al. (2011)<br>Hoppenrath et al. (2012a,b)<br>Stock et al. (2012)<br>Yoon et al. (2012)<br>Qiu et al. (2013)<br>Reñé et al. (2013)<br>Saburova and Chomérat (2014)<br>Takano et al. (2014)<br>Mertens et al. (2015b)

				Reñé et al. (2015)<br>Gómez et al. (2017a)<br>Hoppenrath et al. (2017)<br>Li et al. (2017)<br>Annenkova (2018)<br>Shin et al. (2019)<br>Efimova et al. (2019)<br>Gómez et al. (2019a)<br>Shin et al. (2019)
undulans	YP			Reñé et al. (2015)
viridescens			Y	Reñé et al. (2015)<br>Annenkova (2018)
Haidadinium				
ichthyophilum<br>ex Gasterosteus aculeatus	YP			Hehenberger et al. (2017)
Halostylodinium				
arenarium<br>HG2 LC054931	YP			Horiguchi et al. (2000)<br>Saburova and Chomérat (2014)<br>Efimova et al. (2019)
Haplozoon				
axiothellae	YP			Coats et al. (2010)<br>Gómez et al. (2010b)<br>Yamaguchi et al. (2011)<br>Gómez and Skovgaard (2015)<br>Wakeman et al. (2018b)<br>Yamamoto et al. (2020)
exoense	YP			Wakeman et al. (2018b)<br>Yamamoto et al. (2020)
gracile	Y			Yamamoto et al. (2020)
praxillellae	YP			Coats et al. (2010)<br>Gómez and Skovgaard (2015)<br>Jung et al. (2015)<br>Wakeman et al. (2018b)<br>Yamamoto et al. (2020)
pungens	Y			Yamamoto et al. (2020)
Hematodinium				
nastum			YP	Boutrup et al. (2017)
perezi<br>4-2121 FJ844430<br>4-2122 FJ844431<br>ChincoteagueHemat<br>HM067680-HM067683<br>CsH1 DQ925237<br>ex Callinectes sapidus MF-2000<br>AF286023	Y	Y	YP	Skovgaard et al. (2005)<br>Harada et al. (2007)<br>Skovgaard et al. (2007)<br>Guillou et al. (2008)<br>Gómez et al. (2010a)<br>Small et al. (2011)<br>Okamoto et al. (2012)<br>Kim and Park (2014)<br>Gómez and Skovgaard (2015)<br>Gómez et al. (2017a)

ex Liocarcinus depurator<br>EF065717<br>ex Liocarcinus depurator<br>EF065718<br>ITS gentotype III<br>DQ925227 - DQ925236				Li et al. (2017)<br>Gómez et al. (2019a)
perezi ITS gentotype I<br>ex Liocarcinus depurator<br>EF065708-EF0657011<br>EF065713-EF065716<br>EF153724-EF153729		Y		Small et al. (2011)
perezi ITS gentotype II<br>ex Portunus trituberculatus and Scylla serrata<br>EF173451-EF173454		Y		Small et al. (2011)
sp.<br>ex Chionoecetes baird<br>FJ844414<br>ex Nephrops norvegicus<br>FJ844428	Y			Gómez and Gast (2018)
sp.<br>ex Chionoecetes angulatus<br>U52357	YP			Wakeman et al. (2018b)
sp.<br>ex Chionoecetes bairdi<br>FJ844416<br>ex Chionoecetes opilio<br>FJ844422<br>ex Chionoecetes tanneri<br>FJ844425<br>ex Nephrops norvegicus<br>FJ844429	Y			Small et al. (2011)
sp.<br>JQ928405	YP			Gómez and Gast (2018)
All NE and NW Atlantic Host species<br>ex Chionoecetes opilio<br>EF032004<br>many other sequences		Y		Hamilton et al. (2010)
NE Atalantic Langoustines<br>ex Nephrops norvegicus<br>EU096209<br>ex Nephrops norvegicus<br>EF032010<br>many other sequences		Y		Hamilton et al. (2010)
NE Atlantic: Crabs C. meanas and C. pagurus		Y		Hamilton et al. (2010)

ex Carcinus maenas Cm887 EF675761<br>many other sequences				
Hemidinium				
nasutum	YP	YP	YP	Mertens et al. (2012)<br>Mertens et al. (2013)<br>Boutrup et al. (2016)<br>Hansen et al. (2018)<br>Gottschling et al. (2021a)
Herdmania				
litoralis morphogroup 5 (taxon requires further investigation, may be multispecies complex)<br>single-cell PCR AB564300<br>single-cell PCR AB564306<br>single-cell PCR AB564307	Y	Y	Y	Yamaguchi et al. (2011)<br>Hoppenrath et al. (2012b)<br>Mertens et al. (2013)<br>Potvin et al. (2013)<br>Saburova and Chomérat (2014)<br>Kang et al. (2015)<br>Mertens et al. (2015b)<br>Gómez et al. (2017a)<br>Li et al. (2017)<br>Potvin et al. (2018)<br>Saburova and Chomérat (2018)<br>Efimova et al. (2019)<br>Gómez et al. (2019b)<br>Reñé et al. (2019)<br>Žerdoner Čalasan et al. (2019)<br>Gottschling et al. (2021a,b)
Heterocapsa (= Cachonina)				
arctica /subsp. artica / subsp. frigida<br>CCMP445 AY571372	YP	Y	YP	Yoshida et al. (2003)<br>Hansen and Daugbjerg (2004)<br>Kim and Kim (2007)<br>Hoppenrath et al. (2012a)<br>Potvin et al. (2013)<br>Salas et al. (2014)<br>Gu et al. (2016)<br>Kretschmann et al. (2018a,b)<br>Efimova et al. (2019)<br>Gómez and Artigas (2019)<br>Gottschling et al. (2021a,b)
circularisquama<br>HA92-1<br>HG17 LC054932<br>OK1<br>OK2<br>OK3	YP	Y	YP	Yoshida et al. (2003)<br>Iwataki et al. (2004)<br>Litaker et al. (2007)<br>Salas et al. (2014)<br>Yamada et al. (2015)<br>Pinto et al. (2017)<br>Efimova et al. (2019)<br>Tillman et al. (2020)
horiguchii	YP	Y	YP	Yoshida et al. (2003)

FK6-D47					Salas et al. (2014)
huensis			YP		Salas et al. (2014)
illdefina		YP	YP	YP	Yoshida et al. (2003)<br>Salas et al. (2014)<br>Gómez et al. (2015a)
lanceolate<br>TK6-D57		YP	YP	YP	Yoshida et al. (2003)<br>Salas et al. (2014)
minima<br>JK2<br>JK2<br>JD-2012	 KF031311<br>KF031312<br>JX661019	YP	Y	YP	Salas et al. (2014)
niei<br>CCMP 447	 AF274265	Y	YP	YP/YA	Gómez et al. (2010a,b)<br>Hoppenrath et al. (2012b)<br>Nézan et al. (2012)<br>Salas et al. (2014)<br>Yamada et al. (2015)<br>Gómez et al. (2017a)<br>Hoppenrath et al. (2017)<br>Li et al. (2017)<br>Efimova et al. (2019)
ovata		YP	YP	YP	Salas et al. (2014)
psammophila<br>TM43	 LC054933	YP			Yamada et al. (2015)
pseudotriquetra<br>GeoB 222		YP	YP	YP	Iwataki et al. (2004)<br>Salas et al. (2014)<br>Žerdoner Čalasan et al. (2019)<br>Gottschling et al. (2021a,b)
pygmaea<br>CCCM 681<br>CCMP1322<br>CCMP1490<br>CCMP1734<br>CCMP2770<br>UTEX1653	 AF274266<br>FJ939577<br>AB084094<br>EU165312<br>EU165271<br>EF492494	Y	Y	Y	Yoshida et al. (2003)<br>Gottschling et al. (2005a)<br>Attaran-Fariman and Bolch (2007)<br>Litaker et al. (2007)<br>Gómez et al. (2010a,b)<br>Gottschling et al. (2012)<br>Potvin et al. (2013)<br>Salas et al. (2014)<br>Yamada et al. (2015)<br>Kretschmann et al. (2018a,b)<br>Gottschling et al. (2021b)
rotundata		Y	YP	YP	Hansen and Daugbjerg (2004)<br>Iwataki et al. (2004)<br>Hoppenrath and Leander (2007)<br>Kim and Kim (2007)<br>Moestrup et al. (2008)<br>Sparmann et al. (2008)<br>Gómez et al. (2010b)<br>Yamaguchi et al. (2011)<br>Hoppenrath et al. (2012b)

				Salas et al. (2014)<br>Kang et al. (2015)<br>Yamada et al. (2015)<br>Gómez et al. (2017a)<br>Kretschmann et al. (2018a,b)<br>Efimova et al. (2019)
sp.<br>FIU11 EU165273			YP	Salas et al. (2014)
sp.<br>CCMP(NCMA) 424 AY371082<br>FIU 12R EU165274<br>HZS-2011 JN020164	YP	YP	Y	Salas et al. (2014)<br>Kretschmann et al. (2018a,b)<br>Žerdoner Čalasan et al. (2019)
sp.<br>GeoB 222 AY499509		YP		Salas et al. (2014)
sp.<br>AF260399			YP	Daugbjerg et al. (200)<br>Salas et al. (2014)
sp.<br>HCBC88 JN119844			YP	Salas et al. (2014)
sp. 3<br>NIES614 AB084099		YP		Yoshida et al. (2003)
sp. 5<br>NIES473 AB084100		YP		Yoshida et al. (2003)
steinii<br>UTKG4<br>UTKG5<br>UTKG7	Y	Y	Y	Kretschmann et al. (2018a,b)<br>Žerdoner Čalasan et al. (2019)<br>Tillman et al. (2020)<br>Gottschling et al. (2021a,b)
triquetra<br>CCMP448<br>MUCC285 AF022198<br>NIES7	Y	Y	Y	Grzebyk et al. (1998)<br>D'Onofrio et al. (1999)<br>Yoshida et al. (2003)<br>Hansen and Daugbjerg (2004)<br>Iwataki et al. (2004)<br>Kim, S.H. et al. (2004)<br>Gottschling et al. (2005a)<br>Murray et al. (2005)<br>Dolapsakis et al. (2006)<br>Kawami et al. (2006)<br>Attaran-Fariman and Bolch (2007)<br>Hoppenrath and Leander (2007)<br>Iwataki et al. (2007)<br>Kim and Kim (2007)<br>Litaker et al. (2007)<br>Moestrup et al. (2008)<br>Gómez et al. (2010a.b)<br>Yamaguchi et al. (2011)<br>Hoppenrath et al. (2012b)<br>Potvin et al. (2013)<br>Salas et al. (2014)

				Kang et al. (2015)<br>Mertens et al. (2015b)<br>Yamada et al. (2015)<br>Gu et al. (2016)<br>Gómez et al. (2017a)<br>Li et al. (2017)<br>Efimova et al. (2019)<br>Li, Z. et al. (2020)
Heterodinium				
doma	YP			Gómez et al. (2012a)
globosum (= Centrodinium globosum)	YA			Gómez et al. (2012a)
milneri	Y			Gómez et al. (2012a)
pavillardii	YP			Gómez et al. (2012a)
rigdeniae	YA			Gómez et al. (2012a)
scrippsii	Y			Gómez et al. (2012a)
Histioneis				
elongata			YP	Jensen and Daugbjerg (2009)
cymbalaria	YP			Gómez et al. (2011a)
gubernans	YP			Gómez et al. (2011a)
milneri			YP	Jensen and Daugbjerg (2009)<br>Park et al (2011)
longicollis	Y			Gómez et al. (2011a)
sp.<br>FTL62 EU780646	YP	YP	YP	Handy et al. (2009)<br>Park et al (2011)<br>Qiu et al. (2011)<br>Gottschling et al. (2012)<br>Gottschling et al. (2021a,b)
sp.<br>FTL70 EU780645	YP	YP	YP	Handy et al. (2009)<br>Park et al (2011)<br>Qiu et al. (2011)
sp.<br>L334 FJ808696			YP	Park et al (2011)
Huia				
caspica (= Diplopsalis caspica)<br>Cell_1<br>HBI:SD201204a	YP		YP	Liu et al. (2015a)<br>Mertens et al. (2015b)<br>Gu et al. (2016)<br>Efimova et al. (2019)<br>Reñé et al. (2019)<br>Gurdebek et al. (2020)<br>Gottschling et al. (2021b)
Ichthyodinium				
chabelardi AB264776	Y			Coats et al. (2010)<br>Kim and Park (2014)<br>Jung et al. (2015)<br>Gómez and Gast (2018)
sp.	YP			Kim and Park (2014)

ex Gadus morhus				
sp.<br>ex Plectropomus leopardus	YP			Skovgaard and Salomonsen (2009)
sp.<br>PL AB276368	YP			Gómez et al. (2010a,b)
Impagidinium				
caspienense	YP	YP	YP	Mertens et al. (2017a)<br>Zhang et al. (2020)<br>Gottschling et al. (2021a,b)
pallidum	YP		YP	Mertens et al. (2017a)<br>Zhang et al. (2020)
sp.<br>GeoB 1007	YP			Gottschling et al. (2021b)
Islandinium				
minutum<br>Barrow Strait AB780843<br>Isolate 3<br>Isolate 4	YP	YP	Y	Liu et al. (2013)<br>Mertens et al. (2013)<br>Potvin et al. (2013)<br>Liu et al. (2015a)<br>Mertens et al. (2015b)<br>Yamaguchi et al. (2016)<br>Žerdoner Čalasan et al. (2019)<br>Gurdebek et al. (2020)<br>Gottschling et al. (2021a,b)
minutum subsp. barbatum<br>IMINCS1 KY129806<br>IMINCS2 KY129807<br>IMINLS1 KY129808<br>IMINLS2 KY129809	Y		Y	Potvin etr al. (2018)
tricingulatum (= Protoperidinium tricingulatum)	YP	Y	YP	Liu et al. (2013)<br>Mertens et al. (2013)<br>Potvin et al. (2013)<br>Liu et al. (2015a,b)<br>Mertens et al. (2015b)<br>Yamaguchi et al. (2016)<br>Luo et al. (2018a)<br>Potvin et al. (2018)<br>Gurdebek et al. (2020)
Jadwigia				
applanata<br>CCAC0021 EF058240	Y	YP	Y	Lindberg et al. (2005)<br>Calado et al. (2006)<br>Moestrup et al. (2007)<br>Moestrup et al. (2008)<br>Hansen and Daugbjerg (2011)<br>Hoppenrath et al. (2012a)<br>Yamada et al. (2013)<br>Jeong et al. (2014c)<br>Moestrup et al. (2014)

				Yamada et al. (2015)<br>Boutrup et al. (2016)<br>Luo et al. (2016b)<br>Pinto et al. (2017)<br>Takahashi et al. (2017)<br>Wakeman et al. (2018a)<br>Gottschling et al. (2021a,b)
Johsia				
chumphonensis	Y	Y	Y	Luo et al. (2020b)<br>Gottschling et al. (2021a)
Kapelodinium				
vestifici	YP	YP	YP	Boutrup et al. (2016)<br>Boutrup et al. (2017)<br>Takahashi et al. (2019)
Kareniaceae				
kleptoplastic sp.<br>IFR11-001 KJ508389<br>IFR11-015 KJ508390<br>IFR10-474 KJ858681<br>RS-24 AY434686<br>RS-24 FJ823536	YP	YP	YP	Nézan et al. (2014)<br>Li and Shin (2018)<br>Takahashi et al. (2019)
Karenia				
asterichroma			YP	de Salas et al. (2005a)<br>Luo, Z. et al. (2018c)<br>Wang et al. (2018)<br>Benico et al. (2019)<br>Cen et al. (2021)
bicuneiformis (= Karenia bidigitata)	YP	YP	YP	Guillou et al. (2002)<br>Botes and Pitcher (2003)<br>Bergholtz et al. (2005)<br>de Salas et al. (2005a,b)<br>Bergholtz et al. (2006)<br>Siano et al. (2009)<br>Nézan et al. (2014)<br>Gómez et al. (2015b)<br>Luo, Z. et al. (2018c)<br>Wang et al. (2018)<br>Li et al. (2017)<br>Benico et al. (2019)<br>Efimova et al. (2019)<br>Cen et al. (2021)
brevis (= Gymnodinium breve, Ptychodiscus brevis)<br>CCMP718 AF172714	Y	Y	Y	Hansen et al. (2000)<br>Guillou et al. (2002)<br>Hansen and Daugbjerg (2004)<br>Haywood et al. (2004)<br>Bergholtz et al. (2005)<br>de Salas et al. (2005a)

				Gottschling et al. (2005a,b)<br>Murray et al. (2005)<br>Bergholtz et al. (2006)<br>Garcés et al. (2006)<br>Kim and Kim (2007)<br>Litaker et al. (2007)<br>Murray et al. (2007a)<br>Moestrup et al. (2008)<br>Howard et al. (2009)<br>Siano et al. (2009)<br>Hoppenrath et al. (2012a)<br>Qiu et al. (2013)<br>Mertens et al. (2015b)<br>Yamada et al. (2015)<br>Gómez et al. (2017a)<br>Li et al. (2017)<br>Gu et al. (2018)<br>Wakeman et al. (2018a)<br>Wang et al. (2018)<br>Cen et al. (2021)<br>Gottschling et al. (2021b)
brevisulcata (= Gymnodinium brevisulcata)			Y	Bergholtz et al. (2005)<br>de Salas et al. (2005a)<br>Bergholtz et al. (2006)<br>Siano et al. (2009)<br>Nézan et al. (2014)<br>Luo, Z. et al. (2018c)<br>Wang et al. (2018)<br>Benico et al. (2019)<br>Mardones et al. (2020)<br>Ok et al. (2020)<br>Cen et al. (2021)
cristata			Y	Botes and Pitcher (2003)<br>de Salas et al. (2005a)<br>Nézan et al. (2014)<br>Luo, Z. et al. (2018c)<br>Wang et al. (2018)<br>Mardones et al. (2020)<br>Ok et al. (2020)<br>Cen et al. (2021)
longicanalis (= Karenia umbella)	Y	Y	Y	Yang et al. (2004)<br>Bergholtz et al. (2005)<br>de Salas et al. (2005a,b)<br>Bergholtz et al. (2006)<br>Kim and Kim (2007)<br>Siano et al. (2009)<br>Hoppenrath et al. (2012a)<br>Mertens et al. (2012)

				Nézan et al. (2012)<br>Nézan et al. (2014)<br>Reñé et al. (2015)<br>Boutrup et al. (2016)<br>Luo, Z. et al. (2018c)<br>Wang et al. (2018)<br>Benico et al. (2019)<br>Mardones et al. (2020)<br>Ok et al. (2020)<br>Cen et al. (2021)
mikimotoi (= Gymnodinium mikimotoi)	YP	Y	Y	Grzebyk et al. (1998)<br>Hansen et al. (2000)<br>Guillou et al. (2002)<br>Guillou et al. (2002)<br>Hansen and Daugbjerg (2004)<br>Haywood et al. (2004)<br>de Salas et al. (2005a)<br>Gottschling et al. (2005b)<br>Murray et al. (2005)<br>Gottschling et al. (2005a)<br>Bergholtz et al. (2006)<br>Dolapsakis et al. (2006)<br>Garcés et al. (2006)<br>Litaker et al. (2007)<br>Moestrup et al. (2008)<br>Howard et al. (2009)<br>Siano et al. (2009)<br>Gómez et al. (2010b)<br>Gottschling et al. (2012)<br>Hoppenrath et al. (2012a)<br>Nézan et al. (2012)<br>Qiu et al. (2013)<br>Nézan et al. (2014)<br>Saburova and Chomérat (2014)<br>Reñé et al. (2015)<br>You et al. (2015)<br>Gómez et al. (2017a)<br>Li et al. (2017)<br>Luo, Z. et al. (2018c)<br>Benico et al. (2019)<br>Efimova et al. (2019)<br>Mardones et al. (2020)<br>Cen et al. (2021)<br>Gottschling et al. (2021a,b)
papilionacea	YP	Y	Y	Guillou et al. (2002)<br>Haywood et al. (2004)<br>Bergholtz et al. (2005)<br>de Salas et al. (2005a)

				Bergholtz et al. (2006)<br>Siano et al. (2009)<br>Qiu et al. (2013)<br>Nézan et al. (2014)<br>Gómez et al. (2015b)<br>Hoppenrath et al. (2017)<br>Li et al. (2017)<br>Luo, Z. et al. (2018c)<br>Wang et al. (2018)<br>Benico et al. (2019)<br>Efimova et al. (2019)<br>Mardones et al. (2020)<br>Ok et al. (2020)<br>Cen et al. (2021)
selliformis (phylotypes I, II and III)	Y	Y	Y	Bergholtz et al. (2005)<br>de Salas et al. (2005a)<br>Bergholtz et al. (2006)<br>Siano et al. (2009)<br>Nézan et al. (2014)<br>Pandeirada et al. (2014)<br>Boutrup et al. (2016)<br>Wang et al. (2018)<br>Efimova et al. (2019)<br>Benico et al. (2019)<br>Mardones et al. (2020)<br>Ok et al. (2020)<br>Cen et al. (2021)<br>Gottschling et al. (2021b)
sp.			YP	Benico et al. (2019)
sp 1 France, Atlantic<br>IFR11-001 KJ508369<br>IFR11-015 KJ508390<br>IFR868 KJ508369			Y	Nézan et al. (2014)<br>Luo, Z. et al. (2018c)<br>Cen et al. (2021)
sp France Mediterranean			YP	Luo, Z. et al. (2018c)
Karlodinium				
antarcticum	YP		YP	Haywood et al. (2004)<br>de Salas et al. (2008)<br>Siano et al. (2009)<br>Hoppenrath et al. (2012a)<br>Nézan et al. (2014)<br>Wang et al. (2018)<br>Ok et al. (2020)<br>Cen et al. (2021)
armiger	YP	Y	Y	Haywood et al. (2004)<br>Bergholtz et al. (2005)<br>Bergholtz et al. (2006)<br>Garcés et al. (2006)

				de Salas et al. (2008)<br>Hansen and Daugbjerg (2011)<br>Wang et al. (2011)<br>Hoppenrath et al. (2012a)<br>Nézan et al. (2012)<br>Nézan et al. (2014)<br>Mertens et al. (2015b)<br>Reñé et al. (2015)<br>Boutrup et al. (2016)<br>Li and Shin (2018)<br>Luo, Z. et al. (2018c)<br>Wang et al. (2018)<br>Benico et al. (2019)<br>Moreira-González et al. (2019)<br>Benico et al. (2020)<br>Ok et al. (2020)<br>Cen et al. (2021)
australe		Y	Y	Haywood et al. (2004)<br>Bergholtz et al. (2005)<br>de Salas et al. (2005a)<br>Bergholtz et al. (2006)<br>de Salas et al. (2008)<br>Li and Shin (2018)<br>Luo, Z. et al. (2018c)<br>Salgado et al. (2018)<br>Benico et al. (2019)<br>Benico et al. (2020)<br>Ok et al. (2020)<br>Cen et al. (2021)
azanzae			YP	Benico et al. (2020)
ballantinum	YP		Y	Haywood et al. (2004)<br>de Salas et al. (2008)<br>Siano et al. (2009)<br>Hoppenrath et al. (2012a)<br>Nézan et al. (2014)<br>Qiu et al. (2013)<br>Luo, Z. et al. (2018c)<br>Shin et al. (2019)<br>Benico et al. (2019)<br>Gómez et al. (2019a)<br>Benico et al. (2020)<br>Ok et al. (2020)<br>Cen et al. (2021)
conicum	YP		YP	Haywood et al. (2004)<br>de Salas et al. (2008)<br>Siano et al. (2009)<br>Hoppenrath et al. (2012a)<br>Qiu et al. (2013)

				Luo, Z. et al. (2018c)<br>Benico et al. (2019)<br>Benico et al. (2020)<br>Ok et al. (2020)<br>Cen et al. (2021)
corrugatum	YP		YP	Haywood et al. (2004)<br>de Salas et al. (2008)<br>Siano et al. (2009)<br>Hoppenrath et al. (2012a)<br>Nézan et al. (2014)<br>Luo, Z. et al. (2018c)<br>Benico et al. (2019)<br>Ok et al. (2020)
decipiens	YP	Y	Y	Haywood et al. (2004)<br>de Salas et al. (2008)<br>Siano et al. (2009)<br>Hoppenrath et al. (2012a)<br>Nézan et al. (2012)<br>Nézan et al. (2014)<br>Reñé et al. (2015)<br>Benico et al. (2019)<br>Cen et al. (2021)<br>Ok et al. (2020)
digitatum (= Karenia digitatum)		Y	YA	Luo, Z. et al. (2018c)<br>Benico et al. (2019)<br>Benico et al. (2020)<br>Cen et al. (2021)
elegans		Y	Y	Cen et al. (2021)
gentienii			Y	Nézan et al. (2014)<br>Luo, Z. et al. (2018c)<br>Benico et al. (2019)<br>Ok et al. (2020)<br>Cen et al. (2021)
jejuense<br>KAMS0708 FN357291<br>LMBEV135 MG365893<br>LMBEV136 MG365894	Y	Y	Y/YA	Li and Shin (2018)<br>Cen et al. (2021)
sp.<br>IFR981 KJ508386			Y	Benico et al. (2019)<br>Benico et al. (2020)<br>Ok et al. (2020)<br>Cen et al. (2021)
sp.<br>KAMS0708 FN357291	YP	YP	YP	Gottschling et al. (2012)<br>Nézan et al. (2014)<br>Cen et al. (2021)
sp. France<br>IFR981 KJ508386			Y	Nézan et al. (2014)<br>Luo, Z. et al. (2018c)

veneficum (42) (= K. micrum, Gymnodinium galatheanum , Gyrodinium galatheanum)<br>KT-77B AF172712<br>GgaITSC35 AF352366<br>GgaITSC36 AF352367<br>NMBjah047 DQ459434<br>LAMB090611 HQ434334<br>QUCCCM_SS2-14 KX853202	YP	Y	Y	Hansen et al. (2000)<br>Guillou et al. (2002)<br>Hansen and Daugbjerg (2004)<br>Haywood et al. (2004)<br>Shao et al. (2004)<br>Bergholtz et al. (2005)<br>de Salas et al. (2005b)<br>Gottschling et al. (2005a)<br>Murray et al. (2005)<br>Bergholtz et al. (2006)<br>Garcés et al. (2006)<br>Litaker et al. (2007)<br>de Salas et al. (2008)<br>Moestrup et al. (2008)<br>Howard et al. (2009)<br>Siano et al. (2009)<br>Hansen and Daugbjerg (2011)<br>Wang et al. (2011)<br>Gottschling et al. (2012)<br>Hoppenrath et al. (2012a)<br>Qiu et al. (2013)<br>Nézan et al. (2014)<br>Saburova and Chomérat (2014)<br>Takano et al. (2014)<br>Gómez et al. (2015a)<br>Yamada et al. (2015)<br>Gu et al. (2018)<br>Luo, Z. et al. (2018c)<br>Wang et al. (2018)<br>Benico et al. (2019)<br>Benico et al. (2020)<br>Cen et al. (2021)<br>Ok et al. (2020)<br>Gottschling et al. (2021b)
zhouanum		YA	Y	Luo, Z. et al. (2018c)<br>Benico et al. (2019)<br>Benico et al. (2020)<br>Ok et al. (2020)<br>Cen et al. (2021)
Kofoidinium				
cf. pavillardii<br>FG540	Y			Gómez et al. (2010b)<br>Okamoto et al. (2012)<br>Cooney et al. (2020)
sp.<br>FG256	YP			Gómez et al. (2010b)<br>Cooney et al. (2020)
Kolkwitziella				

acuta (= Kolkwitziella gibbera, Kolkwitziella salebrosa)			Y	Mertens et al. (2015b)<br>Gu et al. (2016)
Kirithra				
asteri	YP	YP	Y	Boutrup et al. (2017)<br>Gómez (2018)<br>Hu et al. (2019)<br>Hu et al. (2020)<br>Shin and Matsuoka (2020)<br>Gottschling et al. (2021a)
sigma			Y	Hu et al. (2020)
Kryptoperidinium				
foliaceum<br>UTEX LB1688 KY693721<br>UTEX LB 1688 AF274268	Y	Y	Y	Tamura et al. (2005)<br>Hoppenrath and Leander (2007)<br>Pienaar et al. (2007)<br>Gómez et al. (2010b)<br>Alves-De-Souza et al. (2011)<br>Zhang, Q. et al. (2011b)<br>Hoppenrath et al. (2012b)<br>Gottschling et al. (2012)<br>Saburova et al. (2012)<br>Saburova and Chomérat (2014)<br>Luo et al. (2015)<br>Boutrup et al. (2016)<br>Gómez et al. (2017a)<br>Horiguchi et al. (2017)<br>Li et al. (2017)<br>Yamada et al. (2017)<br>Kretschmann et al. (2018b)<br>Saburova and Chomérat (2018)<br>Gottschling et al. (2019b)
triquetrum (= Glenodinium triquetrum, Heterocapsa triquetra, Peridinium triquetra)	Y	Y		Kretschmann et al. (2018b)<br>Gottschling et al. (2019a,b)
sp.<br>GeoB459 KY693721<br>KryCA MN963958<br>KrySG MN963957<br>KFF-1001 LT906378	Y	Y	Y	Satta et al. (2020)
sp.<br>UTEX1688 AF274268<br>UTEX1688 EF492508<br>UTEX1688 DQ847436<br>UTEX1688 AF231804	YP	YP	YP	Satta et al. (2020)
Leiocephalium				
pseudosanguineum	Y	YP	Y	Takahashi et al. (2015)<br>Hehenberger et al. (2017)

				Jang et al. (2017a,b)<br>LaJeunesse et al. (2018)<br>Raho et al. (2018)
Lepidodinium				
chlorophorum (= Gymnodinium chlorophorum)<br>NIES 1867<br>DIN3	Y	Y	Y	Daugbjerg et al. (2000)<br>Hansen et al. (2000)<br>Hansen and Daugbjerg (2004)<br>Bergholtz et al. (2005)<br>Lindberg et al. (2005)<br>Murray et al. (2005)<br>Hansen et al. (2007b)<br>Kim and Kim (2007)<br>Murray et al. (2007b)<br>Moestrup et al. (2008)<br>Hansen and Daugbjerg (2011)<br>Reñé et al. (2011)<br>Gottschling et al. (2012)<br>Hoppenrath et al. (2012a)<br>Gu et al. (2013e)<br>Nézan et al. (2014)<br>Takano et al. (2014)<br>Gómez et al. (2015b)<br>Gu et al. (2015a)<br>Kretschmann et al. (2015)<br>Reñé et al. (2015)<br>Boutrup et al. (2016)<br>Luo et al. (2016b)<br>Li et al. (2017)<br>Na et al. (2017)<br>Luo et al. (2018b)<br>Romeikat et al. (2019)<br>Shin et al. (2019)<br>Žerdoner Čalasan et al. (2019)
sp.<br>LJ47-3-58	YP		YP	Romeikat et al. (2019)
viride (taxon requires further investigation)<br>MUCC247D AF022199	YP/YA	YP	Y	Grzebyk et al. (1998)<br>Hansen et al. (2007b)<br>Sparmann et al. (2008)<br>Gómez et al. (2010a,b)<br>Hansen and Daugbjerg (2011)<br>Reñé et al. (2011)<br>Gottschling et al. (2012)<br>Hoppenrath et al. (2012a,b)<br>Gu et al. (2013e)<br>Nézan et al. (2014)<br>Takano et al. (2014)<br>Gómez et al. (2015a)<br>Gu et al. (2015a)

				Kretschmann et al. (2015)<br>Reñé et al. (2015)<br>Yamada et al. (2015)<br>You et al. (2015)<br>Horiguchi et al. (2017)<br>Li et al. (2017)<br>Na et al. (2017)<br>Gu et al. (2018)<br>Luo et al. (2018b)<br>Shin et al. (2019)<br>Wakeman et al. (2018a)<br>Gómez et al. (2019a)<br>Romeikat et al. (2019)<br>Žerdoner Čalasan et al. (2019)<br>Gottschling et al. (2021a,b)
Laciniporus				
arabicus	YP	YP	YP	Saburova and Chomérat (2018)<br>Li, Z. et al. (2020)
Lebouridinium				
glaucum (= Katodinium glaucum, Spirodinium glaucum, Gyrodinium glaucum, Massartia glauca)	Y		Y	Reñé et al. (2013)<br>Kim and Kim (2007)<br>Kang et al. (2015)<br>Reñé et al. (2015)<br>Gómez et al. (2016a,b)
sp.<br>SGUH758 KJ763303	YP			Gómez et al. (2016a)
Leonella				
granifera<br>D002<br>GeoB 38<br>GeoB 132	Y	Y	YP	Gottschling et al. (2005a,b)<br>Gottschling et al. (2008)<br>Zinssmeister et al. (2012)<br>Gu et al. (2013a)<br>Reñé and Hoppenrath (2019)<br>Žerdoner Čalasan et al. (2019)<br>Li, Z. et al. (2020)<br>Gottschling et al. (2021b)
Lessardia				
elongata	YP			Saldarriaga et al. (2003)<br>Hoppenrath and Leander (2007)<br>Sparmann et al. (2008)<br>Gómez et al. (2010b)<br>Hoppenrath et al. (2012b)<br>Saburova and Chomérat (2014)<br>Gómez et al. (2015a)<br>Gómez et al. (2017a)<br>Hoppenrath et al. (2020)
Levanderina				

fissa (= Gyrodinium fissum, Gymnodinium nstriatum, Gyrodinium instriatum, Gyrodinium uncatenum) NCMA431	Y	Y	Y	Kim, S.H. et al. (2004)<br>Shao et al. (2004)<br>Gottschling et al. (2005a)<br>Garcés et al. (2006)<br>Kim and Kim (2007)<br>Litaker et al. (2007)<br>Sparmann et al. (2008)<br>Gómez et al. (2010a)<br>Hansen and Daugbjerg (2011)<br>Sampedro et al. (2011)<br>Hoppenrath et al. (2012a)<br>Miranda et al. (2012)<br>Yoon et al. (2012)<br>Reñé et al. (2013)<br>Moestrup et al. (2014)<br>Nézan et al. (2014)<br>Reñé et al. (2015)<br>Luo et al. (2016b)<br>Hoppenrath et al. (2017)<br>Li et al. (2017)<br>Annenkova (2018)<br>Shin et al. (2019)<br>Selina and Efimova (2020)<br>Gottschling et al. (2021a,b)
Lingulodinium				Gu et al. (2018)
polyedra (= Gonyaulax polyedra, Lingulodinium polyedrum in many phylogenies)	Y	Y	Y	Lee et al. (2001)<br>Kim, S.H. et al. (2004)<br>Hoppenrath and Leander (2007)<br>Kim and Kim (2007)<br>Howard et al. (2009)<br>Gómez et al. (2010a,b)<br>Gottschling et al. (2012)<br>Hoppenrath et al. (2012b)<br>Miranda et al. (2012)<br>Akselman et al. (2015)<br>Mertens et al. (2015b)<br>You et al. (2015)<br>Saburova and Chomérat (2014)<br>Salgado et al. (2018)<br>Efimova et al. (2019)<br>Selina and Efimova (2020)<br>Zhang et al. (2020)<br>Gottschling et al. (2021a,b)
Luciella				
atlantis NCMA (CCMP1838, 1839, 1840)	YP	Y	YP	Gottschling et al. (2005a)<br>Litaker et al. (2007)<br>Mason et al. (2007)<br>Gu et al. (2013a)

				Kretschmann et al. (2014)<br>Jeong et al. (2014c)<br>Tillmann et al. (2014)<br>Kang et al. (2015)<br>Kretschmann et al. (2018b)
masanensis<br>Masan Lucy-200505<br>NC Lucy-V27 AY590485<br>NCMA(CCMP)1873<br>NCMA(CCMP)1873<br>NCMA(CCMP)1877<br>NCMA(CCMP)3035<br>VIMS 1050 EU048553<br>VIMS 1041 EU048552	Y	Y	Y	Litaker et al. (2005)<br>Mason et al. (2007)<br>Coats et al. (2010)<br>Gottschling et al. (2012)<br>Gu et al. (2013a)<br>Craveiro et al. (2013)<br>Gottschling and McLean (2013)<br>Jeong et al. (2014c)<br>Saburova and Chomérat (2014)<br>Tillmann et al. (2014)<br>Kang et al. (2015)<br>Kretschmann et al. (2018b)<br>Saburova and Chomérat (2018)<br>Li, Z. et al. (2020)<br>Hoppenrath et al. (2020)<br>Tillmann et al. (2020)<br>Gottschling et al. (2021b)
sp. ribotype 2<br>Florida Lucy AY245689	YP	YP	YP	Mason et al. (2007)<br>Gottschling et al. (2012)<br>Craveiro et al. (2013)<br>Gottschling and McLean (2013)<br>Kretschmann et al. (2014)<br>Tillmann et al. (2014)<br>Žerdoner Čalasan et al. (2019)<br>Li, Z. et al. (2020)<br>Gottschling et al. (2021b)
sp. ribotype 3<br>NCMA(CCMP)1835 AY590477	YP	YP	YP	Mason et al. (2007)<br>Gottschling et al. (2012)<br>Craveiro et al. (2013)<br>Gottschling and McLean (2013)<br>Kretschmann et al. (2014)<br>Žerdoner Čalasan et al. (2019)
sp. ribotype 4<br>HR1SSeptA5 AY590483	YP	YP	YP	Mason et al. (2007)<br>Gottschling et al. (2012)<br>Craveiro et al. (2013)<br>Gottschling and McLean (2013)<br>Tillmann et al. (2014)<br>Žerdoner Čalasan et al. (2019)
Madanidinium				
loirii	Y		Y	Chomérat and Bilien (2014)<br>Chomérat et al. (2014)<br>You et al. (2015)

				Saburova and Chomérat (2018)<br>Wakeman et al. (2018a)<br>Gómez et al. (2019b)
Margalefidinium (subsection of Cochlodinium)				
fulvescens (= Cochlodinium fulvescens)	Y		Y	Iwataki et al. (2007)<br>Iwataki et al. (2008)<br>Hansen and Daugbjerg (2011)<br>Stock et al. (2012)<br>Qiu et al. (2013)<br>Boutrup et al. (2016)<br>Luo et al. (2016b)<br>Hu, Z. et al. (2018)<br>Shin et al. (2019)<br>Lin et al. (2020)
polykrikoides (= Cochlodinium polykrikoides)	Y	Y	Y	Kim, S.H. et al. (2004)<br>Shao et al. (2004)<br>Iwataki et al. (2007)<br>Iwataki et al. (2008)<br>Gómez et al. (2009a)<br>Howard et al. (2009)<br>Hansen and Daugbjerg (2011)<br>Gottschling et al. (2012)<br>Qiu et al. (2013)<br>Reñé et al. (2015)<br>Yamada et al. (2015)<br>Boutrup et al. (2016)<br>Luo et al. (2016b)<br>Hoppenrath et al. (2017)<br>Hu, Z. et al. (2018)<br>Shin et al. (2019)
Marine Alveolate Group I				
sp.<br>DH144-EKD3 AF290064	YP			Skovgaard et al. (2005)
sp.<br>DH145-EKD20 AF290067	YP			Skovgaard et al. (2005)
sp.<br>DH148-EKD22 AF290078	YP			Skovgaard et al. (2005)
sp.<br>OLI11001 AJ402327	YP			Skovgaard et al. (2005)
sp.<br>OLI11511 AJ402343	YP			Skovgaard et al. (2005)
sp.<br>OLI11029 AJ402352	YP			Skovgaard et al. (2005)
sp.<br>OLI11033 AJ402353	YP			Skovgaard et al. (2005)
sp.	YP			Skovgaard et al. (2005)

OLI11038 AJ402328				
Marine Alveolate Group II				
sp.<br>DH147-EKD3 AF290069	YP			Skovgaard et al. (2005)
sp.<br>DH148-EKD AF290079	YP			Skovgaard et al. (2005)
sp.<br>DH148-EKD27 AF290077	YP			Skovgaard et al. (2005)
sp.<br>OLI11009 AJ402348	YP			Skovgaard et al. (2005)
sp.<br>OLI11012 AJ402330	YP			Skovgaard et al. (2005)
sp.<br>OLI11115 AJ402326	YP			Skovgaard et al. (2005)
sp.<br>OLI11023 AJ402335	YP			Skovgaard et al. (2005)
sp.<br>OLI11055 AJ402344	YP			Skovgaard et al. (2005)
sp.<br>OLI11261 AJ402338	YP			Skovgaard et al. (2005)
Matsuokaea (=Ensiculifera)				
loeblichii (=Ensiculifera loeblichii, Pentapharsodinium trachodium, Pentapharsodinium ambiguous)	YP	Y	Y	D’Onofrio et al. (1999)<br>Gottschling et al. (2005a)<br>Attaran-Fariman and Bolch (2007)<br>Gottschling et al. (2008)<br>Craveiro et al. (2013)<br>Craveiro et al. (2015)<br>Li et al. (2015)<br>You et al. (2015)<br>Hoppenrath et al. (2017)<br>Gu et al. (2018)<br>Kretschmann et al. (2018b)<br>Li, Z. et al. (2020)<br>Hoppenrath et al. (2020)<br>Selina and Efimova (2020)<br>Kim, H.J. et al. (2021)
aff. loeblichii<br>GeoB*220	YP	YP	YP	Gottschling et al. (2005a)<br>Žerdoner Čalasan et al. (2019)<br>Luo, Z. et al. (2020)<br>Satta et al. (2020)<br>Gottschling et al. (2021a)
Miliolidium (from Symbiodinium complex)				
leii<br>sp. free-living; Psp1-05<br>sp. D1.1 Marginopora		Y	Y	Pochon and LaJeunesse (2021)
sp.		YP		Pochon and LaJeunesse (2021)

Marginopora, Papua New Guinea; Momigliano and Uthicke 2013				
sp.<br>sediment, Ogasawara Isl.; (Reimer et al. 2010)			YP	Pochon and LaJeunesse (2021)
sp.<br>Psp1-05; free-living, Palau, (Carlos et al. 1999)			YP	Pochon and LaJeunesse (2021)
Moestrupia				
oblonga<br>Spain JF272764			YP	Hansen and Daugbjerg (2011)<br>Moestrup et al. (2014)<br>Pandeirada et al. (2014)<br>Boutrup et al. (2016)<br>Annenkova (2018)<br>Wakeman et al. (2018a)
sp. Clade A<br>G4M4<br>Gyob8<br>G5M5<br>G7M7<br>M15	Y	Y	Y	Prabowo et al. (2016)<br>Gottschling et al. (2021a,b)
Clade B<br>AW3<br>AW4<br>AW22-8<br>AW22-13<br>AW22-15<br>Aw22-17<br>Odo23-1 LC025931<br>SW22-11	Y	Y	Y	Prabowo et al. (2016)<br>Annenkova (2018)<br>Gottschling et al. (2021a,b)
Clade C<br>AW22-12<br>B74<br>B78<br>Odo23-1<br>Odo23-11<br>Odo23-1	Y	Y	Y	Prabowo et al. (2016)<br>Gottschling et al. (2021a,b)
sp.<br>HG228 LC054934	YP			Yamada et al. (2015)
Naiadinium				
polonicum (= Peridiniopsis polonica, Peridinium polonicum)<br>HBI:MG200823a<br>GeoB*683<br>NIES500 AY443017	Y	Y	Y	Craveiro et al. (2011)<br>Hansen and Daugbjerg (2011)<br>Ki et al. (2011)<br>Hoppenrath et al. (2012a)<br>Gu et al. (2013a)<br>Kretschmann et al. (2014)

				Craveiro et al. (2015)<br>Kang et al. (2015)<br>Luo et al. (2015)<br>Boutrup et al. (2016)<br>Luo et al. (2016a,b)<br>Zhang et al. (2016)<br>Boutrup et al. (2017)<br>Hoppenrath et al. (2017)<br>Kretschmann et al. (2018a)<br>Saburova and Chomérat (2018)<br>Efimova et al. (2019)<br>Luo et al. (2019)<br>Žerdoner Čalasan et al. (2019)<br>Luo, Z. et al. (2020)<br>Gottschling et al. (2021a,b)<br>Kim, H.J. et al. (2021)
Nematodinium				
sp<br>isolate 1, UBC3 FJ947041<br>isolate 2, UBC4 FJ947039	YP	YP	YP	Hoppenrath et al. (2009)<br>Hoppenrath et al. (2012a)<br>Gu et al. (2015a)<br>Reñé et al. (2015)Yamada et al. (2015)<br>Luo et al. (2016b)<br>Luo et al. (2018b)<br>Gómez et al. (2019a)<br>Romeikat et al. (2019)<br>Žerdoner Čalasan et al. (2019)<br>Gottschling et al. (2021a,b)
Niea				
acanthocysta (= Oblea acanthocysta)<br>LC005410	YP		Y	Kawami et al. (2006)<br>Potvin et al. (2013)<br>Liu et al. (2015a)<br>Mertens et al. (2015a,b)<br>Gu et al. (2016)<br>Yamaguchi et al. (2016)<br>Gómez et al. (2019b)<br>Reñé et al. (2019)<br>Gurdebek et al. (2020)
chinensis			Y	Liu et al. (2015a)<br>Mertens et al. (2015b)<br>Gu et al. (2016)<br>Yamaguchi et al. (2016)<br>Gurdebek et al. (2020)
torta (= Oblea torta)<br>040817-3 AB716924	YP		Y	Kawami et al. (2006)<br>Gómez et al. (2010a,b)<br>Potvin et al. (2013)<br>Liu et al. (2015a)<br>Mertens et al. (2015b)

				Gu et al. (2016)<br>Yamaguchi et al. (2016)<br>Reñé et al. (2019)<br>Gurdebek et al. (2020)
Noctiluca				
scintillans	Y	YP	YP	Kawami et al. (2006)<br>Hoppenrath and Leander (2007)<br>Kim and Kim (2007)<br>Sparmann et al. (2008)<br>Gómez et al. (2010b)<br>Ki, J.-S. (2010)<br>Ki et al. (2011)<br>Yamaguchi et al. (2011)<br>Gottschling et al. (2012)<br>Kim et al. (2013)<br>Takano et al. (2014)<br>Mertens et al. (2015b)<br>Boutrup et al. (2016)<br>Gu et al. (2018)<br>Cooney et al. (2020)<br>Gottschling et al. (2021a,b)
Nottbeckia				
ochracea (= Hemidinium ochraceum)	YP	YP	YP	Hansen et al. (2018)<br>Gottschling et al. (2021a)
Nusuttodinium				
acidotum (= Gymnodinium acidotum)<br>Gaci-Japan #1<br>Gaci-Japan #2<br>HBI:HB201009a	Y	YP	Y	Takano et al. (2014)<br>Gu et al. (2015a)<br>Kretschmann et al. (2015)<br>Onuma and Horiguchi (2016)<br>Na et al. (2017)<br>Luo et al. (2018b)<br>Gómez et al. (2019a)<br>Romeikat et al. (2019)<br>Žerdoner Čalasan et al. (2019)
aeruginosum (= Gymnodinium aeruginosum)<br>Ainosato<br>Denmark AB921317<br>Docho<br>Gaer-Japan #1<br>Gaer-Japan #2<br>Gaer-Japan #3<br>Gaer-Japan #4<br>Gaer-Japan #5	Y	Y	Y	Takano et al. (2014)<br>Kretschmann et al. (2015)<br>Onuma and Horiguchi (2016)<br>Na et al. (2017)<br>Luo et al. (2018b)<br>Romeikat et al. (2019)<br>Žerdoner Čalasan et al. (2019)<br>Gottschling et al. (2021a,b)
amphidinioides (= Amphidinium amphidinioides, Amphidinium bourrellyi,	Y	Y	Y	Takano et al. (2014)<br>Kretschmann et al. (2015)<br>Onuma and Horiguchi (2016)

Gymnodinium amphidinioides)<br>Aamp-Japan #b<br>Ainosato<br>Ono				Luo et al. (2018b)<br>Gómez et al. (2019a)<br>Romeikat et al. (2019)<br>Žerdoner Čalasan et al. (2019)<br>Gottschling et al. (2021b)
desymbiontum<br>Abuta<br>Fukara<br>Ishikari	Y	Y	Y	Kretschmann et al. (2015)<br>Romeikat et al. (2019)<br>Žerdoner Čalasan et al. (2019)<br>Gottschling et al. (2021b)
eucyaneum (= Gymnodinium eucyaneum	YP		YA	Gómez and Skovgaard (2014)<br>Takano et al. (2014)
latum (= Amphidinium latum)<br>SA no43	Y	Y	Y	Takano et al. (2014)<br>Kretschmann et al. (2015)<br>Annenkova (2018)<br>Romeikat et al. (2019)<br>Žerdoner Čalasan et al. (2019)
limneticum (= Gymnodinium limneticum)	Y		Y	
myriopyrenoides (= Gymnodinium myriopyrenoides)	YP	YP	YP	Gu et al. (2013e)<br>Takano et al. (2014)<br>Kretschmann et al. (2015)<br>Gu et al. (2015a)<br>Reñé et al. (2015)<br>Romeikat et al. (2019)
poecilochroum (= Amphidinium poecilochroum)<br>Aininkappu<br>Hokuto<br>Shibagaki Beach<br>Yakamoshi	Y	YP	Y	Lee, K.H. et al. (2013)<br>Takano et al. (2014)<br>Gu et al. (2015a)<br>Kretschmann et al. (2015)<br>Reñé et al. (2015)<br>Na et al. (2017)<br>Annenkova (2018)<br>Luo et al. (2018b)<br>Gómez et al. (2019a)<br>Romeikat et al. (2019)<br>Žerdoner Čalasan et al. (2019)<br>Gottschling et al. (2021b)
Oblea				
rotunda (= Peridiniopsis rotunda, Glenodinium rotundum - taxon requires further investigation)			YP	Hoppenrath et al. (2017)<br>Liu et al. (2015a)<br>Mertens et al. (2015b)<br>Gu et al. (2016)<br>Yamaguchi et al. (2016)<br>Efimova et al. (2019)<br>Žerdoner Čalasan et al. (2019)
Oodinium				
pouchetii	Y			Gómez and Skovgaard (2015)
Ornithocercus				

heteroporus (= Ornithocercus triclavatus)	Y		YP	Jensen and Daugbjerg (2009)<br>Gómez et al. (2011a)
magnificus (= Ornithocercus minor)<br>CBC4L7	Y	Y	Y	Handy et al. (2009)<br>Jensen and Daugbjerg (2009)<br>Gómez et al. (2011a)<br>Gottschling et al. (2012)<br>Hoppenrath et al. (2012a)<br>Qiu et al. (2011)<br>You et al. (2015)<br>Gu et al. (2018)<br>Luo et al. (2019<br>Gottschling et al. (2021a,b)
quadratus	NA/YA	YP	YP	Handy et al. (2009)<br>Jensen and Daugbjerg (2009)<br>Gómez et al. (2011a)<br>Hansen and Daugbjerg (2011)<br>Qiu et al. (2011)<br>Boutrup et al. (2016)<br>Luo et al. (2016b)
steinii (= O. orbiculatus)	YP	YP	YA	Handy et al. (2009)<br>Jensen and Daugbjerg (2009)
Ostreopsis				
fattorussoi<br>CBA L1020 LT555467<br>KC86 JX065559<br>VGO881 FM994895		Y	Y	Chomérat et al. (2019b)<br>Borsato et al. (2020)<br>Carnicer et al. (2020)<br>Chomérat et al. (2020a)<br>Nascimento et al. (2020)
labens<br>(currently have single identical Ostreopsis labens and Ostreopsis lenticularis sequences)		YA		Ramos et al. (2015)<br>Gómez et al. (2017b)
lenticularis (= sp. 5)<br>CBA0203 JX065552<br>MB80828_4 AB674917<br>O70421_1 AB674918<br>s0587 AB674922<br>Tub10 MK227231<br>UNRFN-P-01 MT336217	YP	Y	Y	Dolapsakis et al. (2006)<br>Leaw et al. (2010)<br>Sato et al. (2011)<br>Kang et al. (2013)<br>Efimova et al. (2014)<br>Carnicer et al. (2015)<br>Ramos et al. (2015)<br>Verma et al. (2016a,b)<br>Zhang, H. et al. (2017)<br>Gómez et al. (2017b)<br>Zhang, H. et al. (2017)<br>Chomérat et al. (2019b)<br>Reñé and Hoppenrath (2019)<br>Borsato et al. (2020)<br>Carnicer et al. (2020)

				Chomérat et al. (2020a)
				Nascimento et al. (2020)
mascarenensis		Y	Y	Borsato et al. (2020)
MN545659				Chomérat et al. (2020a)
MN545660				
MN545663				
sp. cf. ovata clade A	Y	Y	Y	Penna et al. (2005b)
1-AE KF612639				Laza-Martinez et al. (2011)
10BA KF612635				Sato et al. (2011)
10BD KF612633				Kang et al. (2013)
10BE KF612626				Efimova et al. (2014)
A1 AJ311520				Carnicer et al. (2015)
CBA1502 JX065562				Ramos et al. (2015)
CBA1823 JX065564				Gómez, et al. (2016d)
CBA-A FM946082				Verma et al. (2016a,b)
CBA-D FM946077				Gómez et al. (2017b)
CBA-N FM244631				Zhang, H. et al. (2017)
CIM_BA1 MG310157				Chomérat et al. (2019b)
CIM_K1 MG310154				Borsato et al. (2020)
Geo-2 FM244627				Carnicer et al. (2020)
IEO-01BR AJ420006				Nascimento et al. (2020)
IEO-02BR AJ318461				
IEO-03BR AJ491311				
IEO-06BR AJ491312				
IFR-OSTO1MO KJ439619				
IRTA-SMM-15-13 MG551865				
IshiOst50 AB605824				
IshiOst61 AB605818				
KabO13 AB605817				
KAC85 AB674906				
KC17 FM244736				
KC17 FM946100				
KC68 FM946096				
KC70 KM244734				
KC70 FM946098				
KC71 FM946099				
LCA-E7 JF682767				
OIK2 AB605821				
OOAB0801 JX065568				
OOBZT14 KX845008				
OS-12BR FN256423				
Oso.1 KJ781415				
OS-04BR FM244664				
OS-04BR FM994898				
OS-05BR FM244665				
OS-05BR FM994899				
OS-10BR FM997914				
OS-15BR FM997916				

OS-16BR FM997917				
OS-16BR FM244669				
OS-18BR FM244670				
OS-19BR FM997918				
OS-20BR FM997919				
OS-20BR FM244671				
S-19BR FM997920				
S70828 AB674907				
SKLMP_Ve02 MG914029				
T60730 AB674902				
T70828 AB674903				
TroOstF AB605819				
TroOstC AB605823				
UBA-BR KX781270				
UFBA013 KY628450				
UFBA013 KY865734				
UNR-03 NM560105				
UNR-05 NM560106				
UNR-60 NM560108				
UNR-03 NM560112				
UNR-05 NM560113				
VGO693 FM994894				
VGO820 FM244634				
VGO820 FM994892				
VGO822 FM244635				
VGO822 FM994893				
VGO883 FM994930				
VGO883 FM244638				
VGO884 FM994931				
VGO884 FM244639				
VGO886 FM244640				
VGO887 FM244641				
VGO887 FM994932				
VGO960 JX065567				
VGO964 HG972965				
VGO964 JX065566				
VGO1001 JX065551				
sp. cf. ovata clade B		Y	Y	Nascimento et al. (2020)
KC17 FM244736				
VGO611 FM994896				
VGO614 FM244642				
VGO614 FM994897				
UNR-10 MN560107				
UNR-10 MN560114				
sp. cf. ovata clade C		Y	Y	Leaw et al. (2005)
"Indonesia"				Penna et al. (2005b)
1S1D2 KR230007				Fraga et al. (2008)
1S1D2 KX129869				Penna et al. (2010)

1S1D4	KR230008				Laza-Martinez et al. (2011)
1S1D4	KX129870				Kang et al. (2013)
1S1D6	KR230009				Efimova et al. (2014)
1S1D6	KX129871				Rhodes et al. (2014)
CAWD174	AB674904				Carnicer et al. (2015)
HER27	KX055881				Ramos et al. (2015)
OOTL0602	GQ380660				Gómez, et al. (2016d)
OvPR01	AF218457				Gómez et al. (2017b)
OVPT2	AB674905				Reñé and Hoppenrath (2019)
OvPR04	AF218458				Nascimento et al. (2020)
OvPR04	AF244940				
OvSA02	AF218460				
OvSA06	AF218463				
OvSA10	AF218464				
P-0117	KM032202				
TF4OS	AB841250				
TF5OS	AB841251				
sp. cf. ovata clade D			Y	Y	Sato et al. (2011)
LCH001	JX065569				Zhang, H. et al. (2017)
QB06	KC900891				Gómez et al. (2017b)
T145	AB841232				Borsato et al. (2020)
T152	AB841235				Nascimento et al. (2020)
T153	AB841236				
T163	AB841237				
T163	AB841257				
T170	AB841238				
T193	AB841241				
T197	AB841245				
T198	AB841246				
T207	AB841248				
VGO1056	JX065586				
sp. cf. ovata clade E			Y	Y	Zhang, H. et al. (2017)
1G	MH844087				Borsato et al. (2020)
CBA_4	FM244724				Nascimento et al. (2020)
CBA_4	FM997921				
CBA_6	FM244725				
CBA_6	FM997922				
CBA_9	FM244726				
CBA_9	FM997923				
CBA_10	FM244727				
CBA_10	FM997924				
OvPD04	AF218456				
OvPD06	AF218455				
OvPD07	AF076217				
OvPD07	AF218456				
rhodesae		Y	Y	Y	Verma et al. (2016a)
HER2	KX055844				Chomérat et al. (2019b)
HER7	KX055878				Reñé and Hoppenrath (2019)

HER25	KX055874				Borsato et al. (2020)
HER30	KX055843				Carnicer et al. (2020)
HER32	KX055842				Chomérat et al. (2020a)
HER301	KX055841				Nascimento et al. (2020)
siamensis / cf. siamensis		YP	Y	Y	Leaw et al. (2005a)
CBA CNR-T5					Penna et al. (2005b)
CAWD96	AB674915				Penna et al. (2010)
CAWD203	KT868526				Laza-Martinez et al. (2011)
CAWD203	KT868527				David et al. (2013)
CAWD206	KJ422869				Kang et al. (2013)
CSIC-D	FN256431				Efimova et al. (2014)
CSIC-D1	AJ491333				Rhodes et al. (2014)
CSICD7					Carnicer et al. (2015)
Dn18EHU	HQ414222				Ramos et al. (2015)
Dn19EHU	HQ414223				Gómez, et al. (2016d)
Dn20EHU	HQ414224				Verma et al. (2016a,b)
Dn86EHU	JX987675				Gómez et al. (2017b)
VGO-OS5V	FN256430				Zhang, H. et al. (2017)
					Chomérat et al. (2019b)
					Reñé and Hoppenrath (2019)
					Borsato et al. (2020)
					Carnicer et al. (2020)
					Chomérat et al. (2020a)
					David et al. (2020)
					Nascimento et al. (2020)
					Selina and Efimova (2020)
sp. 1 clade B1		YP	Y	Y	Dolapsakis et al. (2006)
J2	KC848728				Sato et al. (2011)
M1	KC848731				Kang et al. (2013)
MB61007_3	AB674909				Efimova et al. (2014)
OVJJ1	HE793379				Carnicer et al. (2015)
s0617	AB674910				Ramos et al. (2015)
s0716	AB841256				Verma et al. (2016a,b)
T70828-5	AB674908				Chomérat et al. (2019b)
					Borsato et al. (2020)
					Carnicer et al. (2020)
					Nascimento et al. (2020)
sp. 2 clade B2			Y	Y	Sato et al. (2011)
clone C	KC848717				Kang et al. (2013)
OdoOst6	AB674913				Efimova et al. (2014)
UrGmb6	AB605815				Carnicer et al. (2015)
					Verma et al. (2016a,b)
					Chomérat et al. (2019b)
					Borsato et al. (2020)
					Carnicer et al. (2020)
					Chomérat et al. (2020a)
					Nascimento et al. (2020)
sp. 3			Y	Y	Sato et al. (2011)

CAWD184 AB674914<br>CAWD221 KM360088<br>CAWD241 KY069060				Carnicer et al. (2015)<br>Ramos et al. (2015)<br>Chomérat et al. (2019b)<br>Carnicer et al. (2020)<br>Borsato et al. (2020)<br>Chomérat et al. (2020a)<br>Nascimento et al. (2020)
sp. 4<br>CAWD179 AB674916		Y		Sato et al. (2011)<br>Carnicer et al. (2015)<br>Ramos et al. (2015)<br>Chomérat et al. (2019b)<br>Borsato et al. (2020)<br>Carnicer et al. (2020)<br>Chomérat et al. (2020a)<br>Nascimento et al. (2020)
sp. 6<br>IR33 AB674920<br>OU11 AB674921<br>OA21 C9 AB605827<br>OA21 C8 AB605826<br>OA21 C10 AB605828<br>OIPR01 AF244941<br>NT013 JX065570		Y	Y	Sato et al. (2011)<br>Carnicer et al. (2015)<br>Ramos et al. (2015)<br>Verma et al. (2016a,b)<br>Zhang, H. et al. (2017)<br>Borsato et al. (2020)<br>Carnicer et al. (2020)<br>Chomérat et al. (2020a,b)
sp. 7<br>TB34OS AB841214<br>TB38OS AB841216<br>TB30OS AB841218<br>TB38OS AB841258		Y	YP	Verma et al. (2016a,b)<br>Chomérat et al. (2019b)<br>Borsato et al. (2020)<br>Carnicer et al. (2020)<br>Nascimento et al. (2020)
sp. 8<br>Reunion Island small cells<br>P-0105 KM032216<br>P-0121 KM032219<br>P-0127 KM032214		Y		Carnicer et al. (2015)<br>Chomérat et al. (2019b)<br>Borsato et al. (2020)<br>Carnicer et al. (2020)<br>Nascimento et al. (2020)
sp. VGO881<br>VGO881 FM244637		YP		Penna et al. (2010)<br>Ramos et al. (2015)
Oxyrrhis				
marina	YP		YP	Murray et al. (2005)<br>Kim and Kim (2007)<br>Ki, J.-S. (2010)<br>Shin et al. (2019)
Oxytoxum				
scolopax (= Corythodinium michaelsarsii)	YP			Gómez and Artigas (2019)
Pachena				
abriliae	Y	YP	YP	Hoppenrath et al. (2020)<br>Gottschling et al. (2021a)
leibnizii	YP			Hoppenrath et al. (2020)

meriddae	YP			Hoppenrath et al. (2020)
sp.			YP	Hoppenrath et al. (2020)
Palatinus				
apiculatus (= Peridinium palatinum)<br>GeoM*762<br>HBI:HB201101a JQ639763<br>HBI:HB201101a JQ639753	Y	Y	Y	Kim and Kim (2007)<br>Craveiro et al. (2009)<br>Craveiro et al. (2009)<br>Hansen and Daugbjerg (2011)<br>Zhang, Q. et al. (2011b)<br>Gu et al. (2013a)<br>Kang et al. (2015)<br>Luo et al. (2015)<br>Boutrup et al. (2016)<br>Luo et al. (2016b)<br>Anglès et al. (2017)<br>Hansen et al. (2018)<br>Kretschmann et al. (2018a)<br>Saburova and Chomérat (2018)<br>Luo et al. (2019)<br>Reñé and Hoppenrath (2019)<br>Žerdoner Čalasan et al. (2019)<br>Li, Z. et al. (2020)<br>Luo, Z. et al. (2020)<br>Gottschling et al. (2021a,b)<br>Kim, H.J. et al. (2021)
laevis<br>AJC6-798 AF260395	YP	YP	YP	Kretschmann et al. (2018a)<br>Luo, Z. et al. (2020)
pseudolaevis (=Peridinium pseudolaeve)			YP	Kim and Kim (2007)<br>Craveiro et al. (2009)<br>Zhang, Q. et al. (2011b)
Paragymnodinium				
shiwhaense<br>Jeong2006-1 AM408889	YP	YP	YP	Hansen and Daugbjerg (2011)<br>Gottschling et al. (2012)<br>Gómez and Skovgaard (2014)<br>Jeong et al. (2014c)<br>Reñé et al. (2014)<br>Gu et al. (2015a)<br>Yamada et al. (2015)<br>Na et al. (2017)<br>Pinto et al. (2017)<br>Annenkova (2018)<br>Dawut et al. (2018)<br>Luo et al. (2018a)<br>Yokouchi et al. (2018)<br>Romeikat et al. (2019)
stigmaticum	YP		YP	Yokouchi et al. (2018)<br>Yokouchi and Horiguchi (2021)
verecundum	YP		YP	Yokouchi and Horiguchi (2021)

Parvodinium				
centenniale (= Procentrum centennial)<br>CCAC002	YP	YP	YP	Gómez et al. (2017a)<br>Kretschmann et al. (2018a)<br>Gottschling et al. (2019a)<br>Žerdoner Čalasan et al. (2019)<br>Li, Z. et al. (2020)<br>Gottschling et al. (2021b)
cf centennial (= Procentrum cf. centennial, Peridinium centenniale)	YP		YP	Zhang, Q. et al. (2011b)<br>Kang et al. (2015)<br>Gómez et al. (2017a)
elpatiewskyi<br>NM604293	YP	YP	YP	Luo, Z. et al. (2020)
inconspicuum (= Peridinium inconspicuum)<br>CCAP1140/3	Y	YP	YP	Ki et al. (2011)<br>Gottschling and McLean (2013)<br>Gu et al. (2013a)<br>Luo et al. (2015)<br>Anglès et al. (2017)<br>Luo et al. (2019)<br>Žerdoner Čalasan et al. (2019)<br>Li, Z. et al. (2020)<br>Luo, Z. et al. (2020)
marciniakii<br>GeoM*701<br>GeoM*709<br>GeoM*750	Y	Y	Y	Kretschmann et al. (2018a)<br>Efimova et al. (2019)<br>Gottschling et al. (2019a)<br>Luo, Z. et al. (2020)<br>Gottschling et al. (2021b)
mixtum (= var. remotum and var. conjuctum)	Y	Y	Y	Kretschmann et al. (2018a)<br>Luo, Z. et al. (2020)
parvulum (= Peridinium parvulum)	YP	YP	YP	Luo, Z. et al. (2020)
sp.<br>UTEX2255	YP	YP	YP	Kretschmann et al. (2018a,b)<br>Žerdoner Čalasan et al. (2019)
trawinskii<br>GeoM*702<br>GeoM*703<br>GeoM*704<br>GeoM*753 MG255427	Y	Y	Y	Kretschmann et al. (2018a)<br>Gottschling et al. (2019a)<br>Luo, Z. et al. (2020)<br>Gottschling et al. (2021a,b)
umbonatum (= Peridinium umbonatum)<br>FACHB329	Y	Y	Y	Hoppenrath and Leander (2007)<br>Gómez et al. (2010b)<br>Ki et al. (2011)<br>Hoppenrath et al. (2012b)<br>Saburova et al. (2012)<br>Gottschling and McLean (2013)<br>Gu et al. (2013a)<br>Kang et al. (2015)<br>Zhang et al. (2016)<br>Anglès et al. (2017)

				Hoppenrath et al. (2017)<br>Kretschmann et al. (2018a)<br>Efimova et al. (2019)<br>Gottschling et al. (2019a)<br>Luo et al. (2019)<br>Žerdoner Čalasan et al. (2019)<br>Li, Z. et al. (2020)<br>Luo, Z. et al. (2020)<br>Gottschling et al. (2021a,b)
cf. umbonatum<br>GeoM*791<br>GeoM*792<br>GeoM*795	Y	Y	Y	Kretschmann et al. (2018a)
Paulsenella				
vonstoschii	YP			Kühn and Medlin (2005)<br>Coats et al. (2010)<br>Jeong et al. (2014c)<br>Kim and Park (2014)<br>Gómez and Skovgaard (2015)<br>Kang et al. (2015)<br>Jung et al. (2015)
Pelagodinium				
beii (= Gymnodinium bei)<br>clone 7963 JF791066<br>ex Globigerinoides ruber U41087<br>Globigerinoides conglobatus U37365<br>RCC:1491 KF422623	Y	Y	Y	Grzebyk et al. (1998)<br>Gast and Caron (2001)<br>Guillou et al. (2002)<br>Siano et al. (2010)<br>Fodor (2014)<br>Jeong et al. (2014a)<br>Saburova and Chomérat (2014)<br>Takahashi et al. (2014)<br>Gómez et al. (2015a)<br>Takahashi et al. (2015)<br>Luo et al. (2016b)<br>Jang et al. (2017a,b)<br>Li et al. (2017)<br>LaJeunesse et al. (2018)<br>Wakeman et al. (2018a)<br>Raho et al. (2018)
sp. P1<br>AC24 JX661024<br>DOi_325R DQ195282<br>DT5_174 DQ195344<br>DE_33 DQ195346<br>DT8_801 DQ195347<br>DE_32 DQ195353<br>DOi_211 DQ195355<br>DT8_34 DQ195357<br>DT8_32 DQ195358		Y	Y	Takahashi et al. (2014)<br>Potvin et al. (2015)<br>LaJeunesse et al. (2018)<br>Gottschling et al. (2021b)

DT8_170 DQ195359<br>DT8_644 DQ198021<br>DT8_492 DQ198023<br>DRe_2105 DQ198028<br>DT8_679 DQ198029<br>KP342301 EP-2015 GQ422124				
sp. P2<br>DOi_139 DQ198058<br>DOi_489R DQ195284<br>DQ195361 DT5_325<br>DQ195362 DT5_240<br>DQ195363 DT8_33<br>DQ195364 DVf_13<br>DQ195365 DT5_292<br>DRe_954 DQ198067<br>DRe_1064 DQ198062<br>DRe_1065 DQ195370<br>DRe_1219 DQ195369<br>DRe_1567 DQ198059<br>DT5_212 DQ198069<br>DT5-394R DQ195283		Y	Y	Potvin et al. (2015)<br>LaJeunesse et al. (2018)
sp.<br>AR1 JX661023<br>AR1 JX661025	YP	YP	Y	Potvin et al. (2015)<br>Takahashi et al. (2015)<br>LaJeunesse et al. (2018)
sp.<br>AC 24-2 JX661022<br>AC 24-2 JX661027	YP	YP		Potvin et al. (2015)<br>Takahashi et al. (2015)
Pellucidodinium				
psammophilum<br>Aininkappu<br>Nosappu	Y	YP	Y	Na et al. (2017)<br>Luo et al. (2018b)<br>Gómez et al. (2019a)<br>Romeikat et al. (2019)<br>Žerdoner Čalasan et al. (2019)
Pentaplacodinium				
saltonense (= Ceratocorys mariaovidiorum)		Y	Y	Mertens et al. (2018)<br>Luo, Z. et al. (2020a)<br>Selina and Efimova (2020)<br>Zhang et al. (2020)
usupianum	YP	Y	Y	Luo, Z. et al. (2020a)<br>Zhang et al. (2020)
Pentapharsodinium				
imariensis (= “Ensiculifera” imariensis)	YP	Y	Y	D’Onofrio et al. (1999)<br>Montresor et al. (2003)<br>Gottschling et al. (2005a)<br>Attaran-Fariman and Bolch (2007)<br>Gottschling et al. (2008)<br>Li et al. (2015)

				Hoppenrath et al. (2017)<br>Žerdoner Čalasan et al. (2019)<br>Li, Z. et al. (2020)
cf. imariensis (= "Ensiculifera" cf. imariensis)<br>GeoB 284<br>D207	YP	YP		Gottschling et al. (2005a)<br>Kretschmann et al. (2018b)<br>Gottschling et al. (2021a,b)
sp.<br>CCMP771 AF274270	YP			Gómez et al. (2017a)<br>Satta et al. (2020)
sp.<br>SSND22 EU728696		Y		Li, Z. et al. (2020)
Peridiniale s				
sp.<br>PRS2c_4E_04	YP	YP	YP	Žerdoner Čalasan et al. (2019)
Peridiniella				
catenata	YP	YP	YP	Daugbjerg et al. (2000)<br>Hansen and Daugbjerg (2004)<br>Murray et al. (2005)<br>Moestrup et al. (2008)<br>Hansen and Daugbjerg (2011)<br>Yamaguchi et al. (2011)<br>Nézan et al. (2012)<br>Baytut et al. (2016)<br>Boutrup et al. (2016)<br>Luo et al. (2016b)<br>Gottschling et al. (2021a)
sp.<br>NC-2011 isolate IFR10-078<br>JQ247714	YP	YP	YP	Nézan et al. (2012)<br>Baytut et al. (2016)<br>Hoppenrath et al. (2017)<br>Chomérat et al. (2019)<br>Gottschling et al. (2021a)
sp.<br>DAB09			YP	Baytut et al. (2016)
Peridiniopsis				
borgei (= Peridinium borgei)<br>PBSK-A EF058241	YP	YP	YP	Zhang, Q. et al. (2011b)<br>Craveiro et al. (2011)<br>Hansen and Daugbjerg (2011)<br>Zhang, Q. et al. (2011a)<br>Gottschling and McLean (2013)<br>Gu et al. (2013a)<br>Luo et al. (2015)<br>You et al. (2015)<br>Yamada et al. (2015)<br>Boutrup et al. (2016)<br>Luo et al. (2016b)<br>Zhang et al. (2016)<br>Anglès et al. (2017)

				Gu et al. (2018)<br>Hansen et al. (2018)<br>Žerdoner Čalasan et al. (2019)<br>Li, Z. et al. (2020)<br>Gottschling et al. (2021a,b)
Peridinium				
bipes	YP	YP	YP	Daugbjerg et al. (2000)<br>Hansen and Daugbjerg (2004)<br>Murray et al. (2005)<br>Dolapsakis et al. (2006)<br>Kawami et al. (2006)<br>Ki et al. (2011)<br>Hoppenrath et al. (2012a,b)<br>Yoon et al. (2012)<br>Gu et al. (2013a)<br>Potvin et al. (2013)<br>Reñé et al. (2013)<br>Saburova and Chomérat (2014)<br>Zhang et al. (2016)<br>Gottschling et al. (2021a)
bipes f. globosum<br>NIES495	Y	Y	Y	Ki et al. (2011)<br>Gottschling and McLean (2013)<br>Gu et al. (2013a)<br>Luo et al. (2015)<br>Kretschmann et al. (2018b)<br>Gottschling et al. (2019a)<br>Žerdoner Čalasan et al. (2019)<br>Li, Z. et al. (2020)<br>Gottschling et al. (2021b)
bipes var. / f. occultatum<br>HYJA0310<br>NIES497	Y	Y	Y	Gu et al. (2013a)<br>Luo et al. (2015)<br>Kretschmann et al. (2018b)<br>Gottschling et al. (2019a)<br>Žerdoner Čalasan et al. (2019)<br>Gottschling et al. (2021b)
cinctum / cf. cinctum<br>CCAC0102<br>D136<br>GeoM*778<br>PCGY-4<br>PCINC-CCAP DQ166209<br>SAG2017	Y	Y	Y	Hansen and Daugbjerg (2004)<br>Gottschling et al. (2005a)<br>Murray et al. (2005)<br>Kawami et al. (2006)<br>Attaran-Fariman and Bolch (2007)<br>Kim and Kim (2007)<br>Moestrup et al. (2008)<br>Gómez et al. (2010a,b)<br>Hansen and Daugbjerg (2011)<br>Nézan et al. (2012)<br>Craveiro et al. (2013)<br>Gu et al. (2013a)

				Potvin et al. (2013)<br>Reñé et al. (2013)<br>Kang et al. (2015)<br>Luo et al. (2015)<br>Mertens et al. (2015b)<br>Yamada et al. (2015)<br>Gu et al. (2016)<br>Zhang et al. (2016)<br>Gómez et al. (2017a)<br>Li et al. (2017)<br>Kretschmann et al. (2018b)<br>Gómez et al. (2019b)<br>Gottschling et al. (2019a)<br>Reñé et al. (2019)<br>Žerdoner Čalasan et al. (2019)<br>Li, Z. et al. (2020)<br>Gottschling et al. (2021a,b)
fusiformis<br>050701-1 AB716912			YP	Reñé et al. (2019)
dalei (= "Pentapharsodinium" dalei)<br>SCCAP K-1100 JX262492	Y	Y	Y	D'Onofrio et al. (1999)<br>Montresor et al. (2003)<br>Gottschling et al. (2005a)<br>Attaran-Fariman and Bolch (2007)<br>Gu et al. (2013d)<br>Saburova and Chomérat (2014)<br>Li et al. (2015)<br>Boutrup et al. (2016)<br>Zhang et al. (2016)<br>Hoppenrath et al. (2017)<br>Kretschmann et al. (2018b)<br>Efimova et al. (2019)<br>Luo et al. (2019)<br>Žerdoner Čalasan et al. (2019)<br>Li, Z. et al. (2020)<br>Satta et al. (2020)<br>Gottschling et al. (2021b)<br>Kim, H.J. et al. (2021)
dalei var. aciculiferum (= Pentapharsodinium dalei var. aciculiferum)		Y		Gu et al. (2013d)
gatunense<br>PGDA-1	Y	YP	YP	Ki et al. (2011)<br>Gottschling and McLean (2013)<br>Luo et al. (2015)<br>You et al. (2015)<br>Zhang et al. (2016)<br>Gu et al. (2018)<br>Gottschling et al. (2019a)<br>Žerdoner Čalasan et al. (2019)

				Gottschling et al. (2021b)
limbatum<br>plssu1<br>plssu2<br>plssu3	Y			Ki et al. (2011)<br>Luo et al. (2015)<br>Gottschling et al. (2019a)<br>Gottschling et al. (2021b)
pseudo-laeve			YP	Daugbjerg et al. (2000)<br>Murray et al. (2005)
quadricorne<br>AB246744	YP			Luo et al. (2015)<br>Yamada et al. (2015)
sociale	YP			Luo et al. (2015)<br>Gómez (2016b)<br>Zhang et al. (2016)
volzii<br>NIES501	YP	YP	YP	Ki et al. (2011)<br>Kang et al. (2015)<br>Luo et al. (2015)<br>Gottschling et al. (2019a)<br>Žerdoner Čalasan et al. (2019)<br>Gottschling et al. (2021b)
wierzejski	Y			Gómez et al. (2010b)<br>Ki et al. (2011)<br>Zhang et al. (2016)<br>Hoppenrath et al. (2017)<br>Gómez et al. (2017a)
willei<br>Lund1<br>NIES 304 AF274272<br>PCWA-1<br>TK007	Y	Y	Y	Daugbjerg et al. (2000)<br>Hansen and Daugbjerg (2004)<br>Gottschling et al. (2005a,b)<br>Murray et al. (2005)<br>Kim and Kim (2007)<br>Moestrup et al. (2008)<br>Sparmann et al. (2008)<br>Gómez et al. (2010a,b)<br>Hansen and Daugbjerg (2011)<br>Ki et al. (2011)<br>Hoppenrath et al. (2012b)<br>Yoon et al. (2012)<br>Gu et al. (2013a)<br>Potvin et al. (2013)<br>Reñé et al. (2013)<br>Jeong et al. (2014c)<br>Kang et al. (2015)<br>Mertens et al. (2015b)<br>Gómez et al. (2015a)<br>Yamada et al. (2015)<br>Gu et al. (2016)<br>Luo et al. (2016b)<br>Yamaguchi et al. (2016)<br>Li et al. (2017)

				Gottschling et al. (2019a)<br>Žerdoner Čalasan et al. (2019)<br>Gottschling et al. (2021a,b)
cf. willei<br>Lund 1	YP			Gottschling et al. (2021b)
Pernambugia				
tuberosa<br>GeoB*61	YP	YP	YP	Gottschling et al. (2005a,b)<br>Attaran-Fariman and Bolch (2007)<br>Gottschling et al. (2008)<br>Zinssmeister et al. (2011)<br>Zinssmeister et al. (2012)<br>Gu et al. (2013c)<br>Craveiro et al. (2015)<br>Craveiro et al. (2016)<br>Luo, Z. et al. (2016a)<br>Kretschmann et al. (2018a)<br>Gottschling et al. (2019a)<br>Lee, S.Y. et al. (2019)<br>Žerdoner Čalasan et al. (2019)<br>Li, Z. et al. (2020)<br>Gottschling et al. (2021a,b)<br>Kim, H.J. et al. (2021)
Pfiesteria				
piscicida	Y	Y	Y	Saito et al. (2002)<br>Gottschling et al. (2005a)<br>Litaker et al. (2005)<br>Murray et al. (2005)<br>Seaborn et al. (2006)<br>Litaker et al. (2007)<br>Logares et al. (2007)<br>Coats et al. (2010)<br>Gómez et al. (2010a,b)<br>Gottschling et al. (2012)<br>Hoppenrath et al. (2012b)<br>Nézan et al. (2012)<br>Craveiro et al. (2013)<br>Gottschling and McLean (2013)<br>Gu et al. (2013a)<br>Hoppenrath et al. (2013)<br>Jeong et al. (2014c)<br>Tillmann et al. (2014)<br>Gómez et al. (2015a)<br>Jung et al. (2015)<br>Kang et al. (2015)<br>Li et al. (2017)<br>Kretschmann et al. (2018b)<br>Reñé and Hoppenrath (2019)<br>Žerdoner Čalasan et al. (2019)

				Hoppenrath et al. (2020)<br>Li, Z. et al. (2020)<br>Gottschling et al. (2021a,b)<br>Kim, H.J. et al. (2021)
Pfiesteria-like species				
sp.<br>Pfiesteria-like dinoflatellate<br>AY245689			YP	Hoppenrath et al. (2020)
sp.<br>Pfiesteria-like dinoflatellate<br>AY590478			YP	Hoppenrath et al. (2020)
sp.<br>Pfiesteria-like species Bullet<br>AY251288	YP			Mason et al. (2007)
Pheopolykrikos				
beauchampii<br>isolate 1 DQ371294<br>isolate 2 DQ371295	Y	YP	YP	Sparmann et al. (2008)<br>Gómez et al. (2009a)<br>Gómez et al. (2010a,b)<br>Hoppenrath et al. (2012b)<br>Tang, Y.Z. et al. (2013)<br>Reñé et al. (2014)<br>Saburova and Chomérat (2014)<br>Gómez et al. (2015b)<br>Reñé et al. (2015)<br>Li et al. (2017)<br>Na et al. (2017)<br>Žerdoner Čalasan et al. (2019)
Phalacroma				
acutum (LSU seqeunec sme as P. poroictyum sequences – may be misidentified isolate)			YA	Park et al (2011)<br>Baytut et al. (2016)
apicatum			YP	Jensen and Daugbjerg (2009)
cuneus			Y	Jensen and Daugbjerg (2009)<br>Park et al (2011)<br>Baytut et al. (2016)<br>Daugbjerg et al. (2019)
doryphorum	Y		YP	Jensen and Daugbjerg (2009)<br>Gómez et al. (2011a)<br>Park et al (2011)<br>Baytut et al. (2016)<br>Daugbjerg et al. (2019)
favus	YA			Gómez et al. (2011a)
mitra	Y		YP	Jensen and Daugbjerg (2009)<br>Gómez et al. (2011a)<br>Saburova and Chomérat (2014)<br>Baytut et al. (2016)

oxytoxoides (= Oxyphysis oxytoxoides)	YP		YP	Kim and Kim (2007)<br>Gómez et al. (2011a)<br>Park et al (2011)<br>Saburova and Chomérat (2014)<br>Baytut et al. (2016)<br>Daugbjerg et al. (2019)
"ovum" invalid species - LSU sequence groups with P. doryphorum			YP	Park et al (2011)<br>Baytut et al. (2016)<br>Daugbjerg et al. (2019)
cf. parvalum<br>FJ808708			YP	Daugbjerg et al. (2019)
porodictyum	Y		YP	Jensen and Daugbjerg (2009)<br>Gómez et al. (2011a)<br>Hansen and Daugbjerg (2011)<br>Boutrup et al. (2016)<br>Luo et al. (2016b)<br>Boutrup et al. (2017)<br>Daugbjerg et al. (2019)
rapa<br>CBC4 L5I<br>CBC4 L201<br>FTL 67	Y	Y	Y	Handy et al. (2009)<br>Gómez et al. (2011a)<br>Qiu et al. (2011)<br>Gottschling et al. (2012)<br>You et al. (2015)<br>Gu et al. (2018)<br>Wakeman et al. (2018a)<br>Gómez and Artigas (2019)<br>Gottschling et al. (2021a,b)
rotundatum / cf. rotundatum<br>DrotO8-22 AJ506975<br>DrotO11-3 AJ506979<br>FTLL 10<br>FTL71<br>FTL121	Y	Y	Y	Handy et al. (2009)<br>Gómez et al. (2011a)<br>Park et al (2011)<br>Qiu et al. (2011)<br>Gottschling et al. (2012)<br>Saburova and Chomérat (2014)<br>Baytut et al. (2016)<br>Gómez et al. (2017a)<br>Daugbjerg et al. (2019)<br>Gottschling et al. (2021a,b)
sp. ribotype 1<br>Cell 41 MK192804			YP	Daugbjerg et al. (2019)
sp. ribotype 2<br>Cell 43 MK192805			YP	Daugbjerg et al. (2019)
sp. ribotype 3<br>Cell 37 MK192806			YP	Daugbjerg et al. (2019)
sp.<br>L78 FJ808711				Daugbjerg et al. (2019)
sp.<br>CBC4L5 EU780655	YP	YP	YP	Gottschling et al. (2012)

sp.<br>CBC4L128	YP	YP	YP	Handy et al. (2009)
sp.<br>FTL1 10 EU789056	YP			Qiu et al. (2011)
Phytodinium				
sp.	YP			Hehenberger et al. (2017)
Pileidinium				
ciceropse	YP			Hoppenrath et al. (2007)<br>Saburova and Chomérat (2014)<br>Gómez et al. (2015a)<br>Wakeman et al. (2018a)<br>Gómez et al. (2019b)<br>Yamamoto et al. (2020)
Piscinoodinium				
sp.<br>RWL-2007 clone 4 EF016918<br>clone 626 7 062900 EF016921<br>clone 84 D 081001 EF016922<br>clone 3 03-0321 EF016923	Y			Levy et al. (2007)<br>Coats et al. (2010)<br>Annenkova et al. (2011)<br>Gómez and Skovgaard (2015)<br>Takahashi et al. (2015)<br>Hehenberger et al. (2017)<br>Jang et al. (2017b)
piscinoodinium related sp.<br>M5-9BR FJ923469<br>M5-4BI FJ923471	Y			Annenkova et al. (2011)
Plagiodinium				
ballux	YP			Gómez et al. (2019b)<br>Yamada et al. (2019)
belizeanum<br>HG225 LC054937	YP		YP	Yamada et al. (2015)<br>Wakeman et al. (2018a)<br>Gómez et al. (2019b)<br>Yamada et al. (2019)
sp.<br>HG177 LC054937	YP			Yamada et al. (2015)<br>Gómez et al. (2019b)
Planodinium				
striatum	Y			Gómez et al. (2019b)
Podolampas				
bipes<br>FG91 FJ888595<br>(either species misidentified or resolution between P. elegans and P. palmipes is poor)	YP			Gómez et al. (2010a,b)<br>Gómez et al. (2015b)<br>Li et al. (2017)<br>Yamaguchi et al (2018)<br>Gómez et al. (2019b)<br>Hoppenrath et al. (2020)
elegans<br>FG265 FJ888596	YA			Gómez et al. (2010a)<br>Yamaguchi et al (2018)<br>Gómez et al. (2019b)<br>Hoppenrath et al. (2020)
palmipes	YA			Gómez et al. (2010a)

FG51 FJ888594				Yamaguchi et al (2018)<br>Gómez et al. (2019b)<br>Hoppenrath et al. (2020)
spinifera<br>FG267 FJ888597	YP			Gómez et al. (2010a)<br>Yamaguchi et al (2018)<br>Gómez et al. (2019b)<br>Hoppenrath et al. (2020)
Polarella				
glacialis<br>NCMA1383<br>ss5 AF099183	Y	Y	Y	Hansen and Daugbjerg (2004)<br>Murray et al. (2005)<br>Gribble and Anderson (2006)<br>Iwataki et al. (2007)<br>Murray et al. (2007a)<br>Moestrup et al. (2008)<br>Sparmann et al. (2008)<br>Moestrup et al. (2009b)<br>Gómez et al. (2010a,b)<br>Gottschling et al. (2012)<br>Hoppenrath et al. (2012b)<br>Saburova and Chomérat (2014)<br>Mertens et al. (2015b)<br>Gómez et al. (2017a)<br>Jang et al. (2017a,b)<br>Li et al. (2017)<br>LaJeunesse et al. (2018)<br>Raho et al. (2018)<br>Reñé et al. (2019)<br>Gottschling et al. (2021a,b)
Polykrikos				
geminatum<br>(taxon requires further investigation)	Y	YP	Y	Qiu et al. (2013)<br>Gómez and Skovgaard (2014)<br>Kretschmann et al. (2015)<br>Reñé et al. (2015)<br>Na et al. (2017)<br>Luo et al. (2019)<br>Shin et al. (2019)<br>Romeikat et al. (2019)<br>Žerdoner Čalasan et al. (2019)<br>Gottschling et al. (2021b)
hartmannii (= Pheopolykrikos hartmannii)<br>FR4	Y	YP	Y	Kim, S.H. et al. (2004)<br>Iwataki et al. (2007)<br>Kim and Kim (2007)<br>Sparmann et al. (2008)<br>Hoppenrath et al. (2009)<br>Hoppenrath et al. (2012a)<br>Qiu et al. (2013)<br>Tang, Y.Z. et al. (2013)

				Reñé et al. (2014)<br>Takano et al. (2014)<br>Gómez et al. (2015a)<br>Reñé et al. (2015)<br>Boutrup et al. (2016)<br>Na et al. (2017)<br>Annenkova (2018)<br>Wakeman et al. (2018a)<br>Gómez et al. (2019a)<br>Romeikat et al. (2019)<br>Žerdoner Čalasan et al. (2019)
herdmaiae	YP	YP		Gómez et al. (2009a)<br>Gómez et al. (2010a,b)<br>Gómez and Skovgaard (2014)<br>Na et al. (2017)
kofoidii	Y	YP	Y	Kim and Kim (2007)<br>Sparmann et al. (2008)<br>Gómez et al. (2009a)<br>Hoppenrath et al. (2009)<br>Gómez et al. (2010a,b)<br>Hansen and Daugbjerg (2011)<br>Hoppenrath et al. (2012a,b)<br>Qiu et al. (2013)<br>Gómez and Skovgaard (2014)<br>Reñé et al. (2014)<br>Reñé et al. (2015)<br>Gómez et al. (2015b)<br>Gu et al. (2015a)<br>Mertens et al. (2015b)<br>Boutrup et al. (2016)<br>Na et al. (2017)<br>Annenkova (2018)<br>Romeikat et al. (2019)<br>Shin et al. (2019)
lebourae	Y	YP	Y	Sparmann et al. (2008)<br>Gómez et al. (2009a)<br>Hoppenrath et al. (2012a)<br>Qiu et al. (2013)<br>Gómez and Skovgaard (2014)<br>Reñé et al. (2014)<br>Reñé et al. (2015)<br>Boutrup et al. (2016)<br>Na et al. (2017)<br>Gómez et al. (2019a)<br>Reñé et al. (2019)
“quadratus”<br>- may not belong to genus Polykrikos	Y			Potvin et al. (2018)

sp.<br>GD1590bp2 EU418966	YP			Gómez and Skovgaard (2014)
schwartzii	Y	YP	Y	Hoppenrath et al. (2009)<br>Hansen and Daugbjerg (2011)<br>Hoppenrath et al. (2012a)<br>Qiu et al. (2013)<br>Kim and Park (2014)<br>Reñé et al. (2014)<br>Gu et al. (2015a)<br>Reñé et al. (2015)<br>Boutrup et al. (2016)<br>Na et al. (2017)<br>Annenkova (2018)<br>Hansen et al. (2018)<br>Romeikat et al. (2019)<br>Shin et al. (2019)
tanit<br>(taxon requires further investigation)	YP	YP	Y	Reñé et al. (2014)<br>Reñé et al. (2015)<br>Na et al. (2017)<br>Žerdoner Čalasan et al. (2019)
Posoniella				
tricarinelloides<br>PTLY01	Y	Y	Y	Gu et al. (2013a)<br>Anglès et al. (2017)<br>Kretschmann et al. (2018b)<br>Žerdoner Čalasan et al. (2019)<br>Li, Z. et al. (2020)<br>Selina and Efimova (2020)<br>Gottschling et al. (2021a,b)
Preperidinium				
meunieri (= Diplopsalopsis minor - taxon requires further investigation)<br>field isolate DQ444232	YP	YP	YP	Gribble and Anderson (2006)<br>Kim and Kim (2007)<br>Ribeiro, et al. (2010)<br>Yamaguchi et al. (2011)<br>Potvin et al. (2013)<br>Liu et al. (2015a)<br>Mertens et al. (2015b)<br>Gu et al. (2016)<br>Yamaguchi et al. (2016)<br>Boutrup et al. (2016)<br>Reñé et al. (2019)<br>Gottschling et al. (2021a)
Prorocentrum				
balticum		Y	YP	Delmail et al. (2011)<br>de Queiroz Mendes et al. (2019)<br>Verma et al. (2019)<br>Sunesen et al. (2020)
bimaculatum			YP	Chomérat et al. (2012)

				David et al. (2014a)<br>Saburova and Chomérat (2014)<br>Luo et al. (2017a)<br>Nascimento et al. (2017)<br>Chomérat et al. (2019)<br>Moreira-González et al. (2019)<br>Verma et al. (2019)<br>Zou et al. (2020)
borbonicum			YP	Murray et al. (2007a)<br>Delmail et al. (2011)<br>David et al. (2014a)<br>Luo et al. (2017a)<br>Chomérat et al. (2019)<br>Verma et al. (2019)<br>Zou et al. (2020)
caipirignum (= Prorocentrum lima morphotype 4 from Zhang et al. 2015)<br>AS4F8 KP063216<br>DS4G4 KP063220<br>K-0625 EF566747<br>PMHV-1 JQ638940<br>SE10 KP063231<br>TIO11 KY010252<br>TIO138 KY010254<br>TIO180 JY010256<br>Y16235		Y	Y	Zhang, H. et al. (2015)<br>Nascimento et al. (2017)<br>Chomérat et al. (2019)<br>de Queiroz Mendes et al. (2019)<br>Nishimura et al. (2020)<br>Sunesen et al. (2020)<br>Zou et al. (2020)
cassubicum		YP	YP	Lim et al. (2013)<br>Verma et al. (2019)<br>Sunesen et al. (2020)
clipeus			Y	Saburova and Chomérat (2014)<br>Hoppenrath et al. (2017)<br>Luo et al. (2017a)<br>Verma et al. (2019)
concavum (= Procentrum arabianumm, Prorocentrum faustiae) relationship to Prorocentrum faustiae was not conclusively determined.	YP	Y	Y	Grzebyk et al. (1998)<br>Hoppenrath and Leander (2007)<br>Murray et al. (2007a)<br>Chomérat et al. (2010)<br>Chomérat et al. (2011)<br>Delmail et al. (2011)<br>Lim et al. (2013)<br>David et al. (2014a)<br>Hoppenrath et al. (2017)<br>Luo et al. (2017a)<br>Nascimento et al. (2017)<br>Wakeman et al. (2018a)<br>Chomérat et al. (2019)

				de Queiroz Mendes et al. (2019)<br>Moreira-González et al. (2019)<br>Verma et al. (2019)<br>Sunesen et al. (2020)<br>Zou et al. (2020)
consutum	Y		YP	Chomérat et al. (2010)<br>Chomérat et al. (2011)<br>Chomérat et al. (2012)<br>Lim et al. (2013)<br>Saburova and Chomérat (2014)<br>Hoppenrath et al. (2017)<br>Luo et al. (2017a)<br>Nascimento et al. (2017)<br>Chomérat et al. (2019)<br>Verma et al. (2019)<br>Sunesen et al. (2020)<br>Zou et al. (2020)
cordatum (= Procentrum minimum)<br>PmiPrMu21 AY421791	Y	Y	Y	Zardoya et al. (1995)<br>Grzebyk et al. (1998)<br>Hansen and Daugbjerg (2004)<br>Kim, S.H. et al. (2004)<br>Shao et al. (2004)<br>Gottschling et al. (2005a)<br>Murray et al. (2005)<br>Kawami et al. (2006)<br>Hoppenrath and Leander (2007)<br>Kim and Kim (2007)<br>Litaker et al. (2007)<br>Murray et al. (2007a)<br>Moestrup et al. (2008)<br>Howard et al. (2009)<br>Chomérat et al. (2010)<br>Cohen-Fernández et al (2010)<br>Leblond et al. (2010)<br>Chomérat et al. (2011)<br>Delmail et al. (2011)<br>Lim et al. (2013)<br>Saburova and Chomérat (2014)<br>Salas et al. (2014)<br>Takano et al. (2014)<br>Gómez et al. (2015a)<br>Yamada et al. (2015)<br>Gómez et al. (2017a,c)<br>Hoppenrath et al. (2017)<br>Luo et al. (2017a)<br>Nascimento et al. (2017)<br>Chomérat et al. (2019)<br>de Queiroz Mendes et al. (2019)

				Verma et al. (2019)<br>Sunesen et al. (2020)<br>Gottschling et al. (2021a,b)
dentatum	YP		Y	Hoppenrath and Leander (2007)<br>Murray et al. (2007a)<br>Chomérat et al. (2010)<br>Gómez et al. (2010a,b)<br>Leblond et al. (2010)<br>Chomérat et al. (2011)<br>Delmail et al. (2011)<br>David et al. (2014a)<br>You et al. (2015)<br>Gómez et al. (2017c)<br>Luo et al. (2017a)<br>Nascimento et al. (2017)<br>Gu et al. (2018)<br>McCarthy et al. (2018)<br>Chomérat et al. (2019)<br>Verma et al. (2019)
elegans		YP	YP	David et al. (2014a)<br>Luo et al. (2017a)<br>de Queiroz Mendes et al. (2019)<br>Verma et al. 2019
emarginatum	YP	Y	Y	Grzebyk et al. (1998)<br>Hoppenrath and Leander (2007)<br>Murray et al. (2007a)<br>Chomérat et al. (2010)<br>Chomérat et al. (2011)<br>Delmail et al. (2011)<br>Laza-Martinez et al. (2011)<br>Lim et al. (2013)<br>Gómez et al. (2015a)<br>You et al. (2015)<br>Hoppenrath et al. (2017)<br>Luo et al. (2017a)<br>Gu et al. (2018)<br>de Queiroz Mendes et al. (2019)<br>Verma et al. (2019)<br>Sunesen et al. (2020)
faustiae	YP		YP/YA	Chomérat et al. (2010)<br>Delmail et al. (2011)<br>Chomérat et al. (2012)<br>David et al. (2014a)<br>Zou et al. (2020)
foraminosum	YP	Y	Y	David et al. (2014a)<br>Hoppenrath et al. (2017)<br>Luo et al. (2017a)

				Nascimento et al. (2017)<br>Moreira-González et al. (2019)<br>Sunesen et al. (2020)<br>Zou et al. (2020)
cf. foraminosum<br>IFR12-17 MF784643<br>IFR12-183 MF784642<br>IFR12-198 MF784657<br>IFR15-049 MF784646<br>IFR15-260 MF784644<br>IFR15-263 MF784645			Y	Chomérat et al. (2019)<br>Verma et al. (2019)
aff. foraminosum<br>IFR11-185 JX912178<br>IFR11-210 JX912176<br>IFR469 JX912177<br>Pf16IMB-AB13 KT203867<br>Pf7IMB-AB13 KT203866			Y	Chomérat et al. (2019)<br>Verma et al. (2019)
foveolatum			YP	Bolch and Campbell (2004)<br>Murray et al. (2007a)<br>Delmail et al. (2011)<br>Lim et al. (2013)<br>David et al. (2014a)<br>Verma et al. (2019)
fukuyoi (may represent multispecies complex)	YP	Y	Y	Murray et al. (2007a)<br>Chomérat et al. (2010)<br>Chomérat et al. (2011)<br>Delmail et al. (2011)<br>Lim et al. (2013)<br>David et al. (2014a)<br>Gómez et al. (2015a)<br>Hoppenrath et al. (2017)<br>Luo et al. (2017a)<br>Chomérat et al. (2019)<br>de Queiroz Mendes et al. (2019)<br>Verma et al. (2019)<br>Sunesen et al. (2020)
glenanicum	Y		YP	Chomérat et al. (2011)<br>Lim et al. (2013)<br>Hoppenrath et al. (2017)<br>Luo et al. (2017a)<br>Dawut et al. (2018)<br>Verma et al. (2019)
gracile (= Procentrum sigmoides)	N/NA		Y	Hoppenrath and Leander (2007)<br>Murray et al. (2007a)<br>Chomérat et al. (2010)<br>Cohen-Fernández et al (2010)<br>Gómez et al. (2010a,b)

				Chomérat et al. (2011)<br>Delmail et al. (2011)<br>Lim et al. (2013)<br>David et al. (2014a)<br>Hoppenrath et al. (2017)<br>Li et al. (2017)<br>Luo et al. (2017a)<br>Nascimento et al. (2017)<br>Chomérat et al. (2019)<br>Verma et al. (2019)<br>Sunesen et al. (2020)
hoffmannianum (= Procentrum belizeanum, Prorocentrum maculosum – may be multi-species complex as there are distinct LSU subclades)<br>PPAN20 Y16236	Y	Y	Y	Hoppenrath and Leander (2007)<br>Murray et al. (2007a)<br>Chomérat et al. (2010)<br>Chomérat et al. (2011)<br>Delmail et al. (2011)<br>Lim et al. (2013)<br>David et al. (2014a)<br>Saburova and Chomérat (2014)<br>You et al. (2015)<br>Luo et al. (2017a)<br>Nascimento et al. (2017)<br>Dawut et al. (2018)<br>Gu et al. (2018)<br>Chomérat et al. (2019)<br>de Queiroz Mendes et al. (2019)<br>Verma et al. (2019)<br>Nishimura et al. (2020)<br>Sunesen et al. (2020)<br>Zou et al. (2020)<br>Gottschling et al. (2021a,b)
koreanum	Y	Y	Y	You et al. (2015)<br>Gómez et al. (2017c)<br>Luo et al. (2017a)<br>Gu et al. (2018)<br>McCarthy et al. (2018)<br>de Queiroz Mendes et al. (2019)<br>Verma et al. (2019)<br>Sunesen et al. (2020)<br>Zou et al. (2020)<br>Gottschling et al. (2021b)
leve<br>NCMA2634	YP	Y	Y	Chomérat et al. (2010)<br>Chomérat et al. (2011)<br>Delmail et al. (2011)<br>Lim et al. (2013)<br>David et al. (2014a)<br>Saburova and Chomérat (2014)<br>Luo et al. (2017a)

				Nascimento et al. (2017)<br>Chomérat et al. (2019)<br>Moreira-González et al. (2019)<br>Verma et al. (2019)<br>Zou et al. (2020)<br>Gottschling et al. (2021a,b)
lima (= Prorcentrum arenarium)<br>PLLS01 AY259170<br>CCMP685 DQ336179<br>Sorrento1 DQ336189<br>Dn35EHU HQ414228<br>Dn38EHU HQ414229<br>Dn37EHU HQ414231<br>Work by Nishimura et al. (2020) and others indicate that this is a species complex that includes more species than initially identified by idenfied by Zhang et al. (2015)	Y	Y	Y	Zardoya et al. (1995)<br>Grzebyk et al. (1998)<br>Hoppenrath and Leander (2007)<br>Murray et al. (2007a)<br>Chomérat et al. (2010)<br>Cohen-Fernández et al (2010)<br>Chomérat et al. (2011)<br>Delmail et al. (2011)<br>Laza-Martinez et al. (2011)<br>Hoppenrath et al. (2012b)<br>Lim et al. (2013)<br>Saburova and Chomérat (2014)<br>You et al. (2015)<br>Zhang, H. et al. (2015)<br>Luo et al. (2017a)<br>Nascimento et al. (2017)<br>Gu et al. (2018)<br>Chomérat et al. (2019)<br>de Queiroz Mendes et al. (2019)<br>Moreira-González et al. (2019)<br>Sunesen et al. (2020)<br>Zou et al. (2020)<br>Gottschling et al. (2021a,b)
cf. lima morphotype 1<br>UFR11-063 AJ567456<br>SM24 DQ336182<br>NMN07 EF566748<br>MNN07 MG701857<br>regarded as Prorocentrum arenarium by Nascimento et al. (2017)		Y	Y	Zhang, H. et al. (2015)<br>Nascimento et al. (2017)<br>Chomérat et al. (2019)<br>Nishimura et al. (2020)
cf. lima morphotype 2<br>DNS-3 DQ336187<br>SM29 DQ336181<br>S4 DQ336187<br>TIO155a KY010251		Y	Y	Zhang, H. et al. (2015)<br>Nishimura et al. (2020)
cf. lima morphotype 3<br>DNS0-3v KP063225<br>SC7v KP063224		Y	Y	Zhang, H. et al. (2015)<br>Chomérat et al. (2019)<br>Nishimura et al. (2020)
cf. lima morphotype 5<br>AS4F8 JP063217		Y	Y	Zhang, H. et al. (2015)<br>Nascimento et al. (2017)

DS4G4 KP063220<br>DS4D9 KP063219<br>TIO179 KY010255<br>TIO180 KY010256				Chomérat et al. (2019)<br>Nishimura et al. (2020)
cf. lima tropical morphotype		Y		Nascimento et al. (2017)
malayense		YP	Y	Verma et al. 2019<br>Sunesen et al. (2020)
mexicanum<br>NMN16	N	Y	Y	Grzebyk et al. (1998)<br>Murray et al. (2005)<br>Hoppenrath and Leander (2007)<br>Kim and Kim (2007)<br>Murray et al. (2007a)<br>Chomérat et al. (2010)<br>Cohen-Fernández et al (2010)<br>Gómez et al. (2010a,b)<br>Leblond et al. (2010)<br>Chomérat et al. (2011)<br>Delmail et al. (2011)<br>Lim et al. (2013)<br>You et al. (2015)<br>Gómez et al. (2017c)<br>Li et al. (2017)<br>Nascimento et al. (2017)<br>Gu et al. (2018)<br>McCarthy et al. (2018)<br>Wakeman et al. (2018a)<br>Chomérat et al. (2019)<br>Moreira-González et al. (2019)<br>Verma et al. (2019)<br>Sunesen et al. (2020)<br>Gottschling et al. (2021b)
micans (= P. arenarium, P. maculosum)<br>M14649<br>NCMA1589 EU780638<br>CCMP1589 EU927525<br>CCMP1589 AY585526	N/NA/Y	Y	Y	Lenaers et al. (1989)<br>McNally et al. (1994)<br>Zardoya et al. (1995)<br>Adachi et al. (1997)<br>Grzebyk et al. (1998)<br>Guillou et al. (2002)<br>Hansen and Daugbjerg (2004)<br>Shao et al. (2004)<br>Gottschling et al. (2005a)<br>Murray et al. (2005)<br>Dolapsakis et al. (2006)<br>Hoppenrath and Leander (2007)<br>Kim and Kim (2007)<br>Moestrup et al. (2008)<br>Sparmann et al. (2008)<br>Howard et al. (2009)<br>Chomérat et al. (2010)

				Cohen-Fernández et al (2010)<br>Gómez et al. (2010a,b)<br>Leblond et al. (2010)<br>Chomérat et al. (2011)<br>Reñé et al. (2013)<br>David et al. (2014a)<br>Saburova and Chomérat (2014)<br>Takano et al. (2014)<br>Gómez et al. (2015a)<br>Yamada et al. (2015)<br>Gómez et al. (2017c)<br>Hoppenrath et al. (2017)<br>Li et al. (2017)<br>Luo et al. (2017a)<br>de Queiroz Mendes et al. (2019)<br>Verma et al. (2019)<br>Sunesen et al. (2020)<br>Zou et al. (2020)<br>Gottschling et al. (2021a,b)
norrisianum			Y	Moreira-González et al. (2019)
obtusidens (= Prorocentrum donghaiense)	Y	Y	Y	Hoppenrath and Leander (2007)<br>Murray et al. (2007a)<br>Chomérat et al. (2010)<br>Gómez et al. (2010a,b)<br>Leblond et al. (2010)<br>Chomérat et al. (2011)<br>Delmail et al. (2011)<br>Craveiro et al. (2013)<br>Saburova and Chomérat (2014)<br>Gómez et al. (2017c)<br>Nascimento et al. (2017)<br>Moreira-González et al. (2019)<br>Sunesen et al. (2020)<br>Gottschling et al. (2021a,b)
panamense	YP	YP	Y	Grzebyk et al. (1998)<br>Hoppenrath and Leander (2007)<br>Chomérat et al. (2010)<br>Chomérat et al. (2011)<br>Lim et al. (2013)<br>You et al. (2015)<br>Hoppenrath et al. (2017)<br>Luo et al. (2017a)<br>Dawut et al. (2018)<br>Gu et al. (2018)<br>de Queiroz Mendes et al. (2019)<br>Sunesen et al. (2020)
playfairii			YP	Murray et al. (2007a)<br>Delmail et al. (2011)

				Lim et al. (2013)<br>Luo et al. (2017a)<br>Nascimento et al. (2017)<br>Verma et al. (2019)<br>Zou et al. (2020)
pseudopanamense	YP			Chomérat et al. (2011)<br>Lim et al. (2013)<br>Hoppenrath et al. (2017a)<br>Dawut et al. (2018)
reticulatum (clades A and B)	Y	Y	Y	Kim, S.H. et al. (2004)<br>Kim and Kim (2007)<br>Howard et al. (2009)<br>Saburova and Chomérat (2014)<br>Akselman et al. (2015)<br>Hoppenrath et al. (2017)<br>Kim and Park (2017)<br>Mertens et al. (2018)<br>Salgado et al. (2018)<br>Selina and Efimova (2020)<br>Zhang et al. (2020)
rhathymum	N	Y	Y	Hansen and Daugbjerg (2004)<br>Murray et al. (2007a)<br>Chomérat et al. (2011)<br>Delmail et al. (2011)<br>Laza-Martinez et al. (2011)<br>Stock et al. (2012)<br>Lim et al. (2013)<br>Reñé et al. (2013)<br>David et al. (2014a)<br>Hoppenrath et al. (2017)<br>Luo et al. (2017a)<br>Nascimento et al. (2017)<br>Chomérat et al. (2019)<br>Sunesen et al. (2020)<br>Zou et al. (2020)
rivalis			YP	Delmail et al. (2011)
rostratum		YP		Gómez et al. (2017c)<br>Sunesen et al. (2020)
sculptile			Y	Delmail et al. (2011)<br>Wakeman et al. (2018a)<br>Chomérat et al. (2019)
sipadanense			YP	Chomérat et al. (2019)<br>Verma et al. (2019)
sp.<br>FL32	YP			Chomérat et al. (2011)
sp.<br>RAV2	YP			Chomérat et al. (2011)

sp. Type I<br>QUN37P10 KC415604			YP	Nishimura et al. (2020)
sp. Type 2<br>QUN248P KC415605<br>PL01011 DQ336188			Y	Nishimura et al. (2020)
steidingerae<br>NCMA687	N	Y	Y	Gómez et al. (2017c)<br>Gómez and Artigas (2019)<br>Sunesen et al. (2020)<br>Gottschling et al. (2021b)
texanum	N	Y	Y	Gómez et al. (2017c)<br>Sunesen et al. (2020)<br>Zou et al. (2020)
triestinum	N/NA	Y	Y	Zardoya et al. (1995)<br>Shao et al. (2004)<br>Hoppenrath and Leander (2007)<br>Chomérat et al. (2010)<br>Gómez et al. (2010a,b)<br>Leblond et al. (2010)<br>Chomérat et al. (2011)<br>Delmail et al. (2011)<br>Lim et al. (2013)<br>Kim and Park (2014)<br>Hoppenrath et al. (2017)<br>Luo et al. (2017a)<br>Nascimento et al. (2017)<br>de Queiroz Mendes et al. (2019)<br>Reñé et al. (2019)<br>Verma et al. (2019)<br>Sunesen et al. (2020)<br>Zou et al. (2020)
tropicale			YP	Moreira-González et al. (2019)<br>Verma et al. (2019)
tsawwassenense	YP		Y	Hoppenrath and Leander (2008)<br>Hoppenrath and Leander (2007)<br>Chomérat et al. (2010)<br>Chomérat et al. (2011)<br>Lim et al. (2013)<br>David et al. (2014a)<br>Luo et al. (2017a)<br>Verma et al. (2019)<br>Zou et al. (2020)
Protoceratium				
reticulatum (probably encompasses at least 3 different cryptic species)<br>PRPV-1	YP	Y	Y	Hansen and Daugbjerg (2004)<br>Murray et al. (2005)<br>Dolapsakis et al. (2006)<br>Hoppenrath and Leander (2007)<br>Gómez et al. (2010a,b,c)

				Hoppenrath et al. (2012b)<br>Boutrup et al. (2016)<br>Li et al. (2017)<br>Li et al. (2019)<br>Reñé and Hoppenrath (2019)<br>Luo, Z. et al. (2020a)<br>Selina and (2020)<br>Gottschling et al. (2021a,b)
reticulatum ribotype A		Y		Wang, N. et al. (2019)
reticulatum ribotype B		Y		Wang, N. et al. (2019)
reticulatum ribotype C		Y		Wang, N. et al. (2019)
Proterythropsis				
sp.<br>BSL-2009a FJ947037	YP			Gómez et al. (2019a)
sp.<br>Isolates 1 and 2	Y			Hoppenrath et al. (2009)
Pseliodinium				
cf. convolutum			YP	Gómez (2018)
falcatum KF245457			YP	Gómez (2018)
cf. helix KF245459			YP	Gómez (2018)
fusus (= Ceratoperidinium falcatum, Gyrodinium caudatum, Gyrodinium falcatum, Gyrodinium sugashimanii, Pseliodinium vaubanii)<br>GFPL01 AY320049<br>IFR1100 KJ508394<br>isolate 1 KF245457<br>isolate 2 KF245458	Y	YP	Y	Reñé et al. (2013)<br>Nézan et al. (2014)<br>Reñé et al. (2015)<br>Boutrup et al. (2017)<br>Na et al. (2017)<br>Gómez (2018)<br>Luo et al. (2018b)<br>Hu et al. (2019)<br>Shin et al. (2019)<br>Hu et al. (2020)<br>Shin and Matsuoka (2020)<br>Gottschling et al. (2021a)
sp.<br>#1 KF245461			YP	Gómez (2018)
Pseudadenoides				
kofoidii<br>PSE6	Y	YP	Y	Gómez et al. (2015a)<br>Hoppenrath et al. (2017)<br>Gu, H. et al. (2018)<br>Saburova and Chomérat (2018)<br>Wakeman et al. (2018a)<br>Gómez et al. (2019b)<br>Sunesen et al. (2020)<br>Gottschling et al. (2021a,b)
polypyrenoides	YP		YP	Hoppenrath et al. (2017)<br>Gu, H. et al. (2018)
Protodinium				

simplex (= Gymnodinium simplex)<br>CCMP419 U41086	YP	YP	Y	Grzebyk et al. (1998)<br>Gast and Caron (2001)<br>Guillou et al. (2002)<br>Sparmann et al. (2008)<br>Moestrup et al. (2009b)<br>Siano et al. (2009)<br>Siano et al. (2010)<br>Balzano et al. (2012)<br>Gottschling et al. (2012)<br>Hoppenrath et al. (2012b)<br>Fodor (2014)<br>Jeong et al. (2014c)<br>You et al. (2015)<br>Luo, Z. et al. (2015)<br>Boutrup et al. (2016)<br>Na et al. (2017)<br>Gu et al. (2018)<br>LaJeunesse et al. (2018)<br>Luo et al. (2018b)
Protoperidinium				
abei (= Peridinium abei)	Y	YP	YP	Yamaguchi and Horiguchi (2005)<br>Kawami et al. (2006)<br>Yamaguchi et al. (2006)<br>Ribeiro, et al. (2010)<br>Gomez et al. (2011b)<br>Yamaguchi et al. (2011)<br>Gottschling and McLean (2013)<br>Mertens et al. (2013)<br>Potvin et al. (2013)<br>Gu et al. (2015b)<br>Liu et al. (2015a,b)<br>Mertens et al. (2015b)<br>Yamaguchi et al. (2016)<br>Luo et al. (2018a)<br>Gurdebek et al. (2020)
abei var. rotunda<br>Yellow Sea KM042422	Y		YP	Gu et al. (2015b)<br>Liu et al (2015b)<br>Gurdebek et al. (2020)
americanum (= “Peridinium americanum”)<br>080218-12 AB716911	Y/YA	YP	Y	Liu et al. (2013)<br>Mertens et al. (2013)<br>Liu et al (2015b)<br>Mertens et al. (2015b)<br>Yamaguchi et al. (2016)<br>Luo et al. (2018a)<br>Potvin et al. (2018)<br>Gómez et al. (2019b)<br>Reñé and Hoppenrath (2019)<br>Reñé et al. (2019)

				Gottschling et al. (2021a,b)
angustum			YP	Gribble and Anderson (2006)<br>Kim and Kim (2007)<br>Ribeiro, et al. (2010)<br>Potvin et al. (2013)<br>Gu et al. (2015b)<br>Liu et al (2015b)<br>Gu et al. (2016)<br>Gurdebek et al. (2020)
avellana (= Properidinium avellana, Peridinium avellana)			YP	Liu et al. (2015a,b)<br>Reñé and Hoppenrath (2019)<br>Gurdebek et al. (2020)
bipes (= Glenodinium bipes, Minuscula bipes)	YP	YP	YP	Ribeiro, et al. (2010)<br>Yamaguchi et al. (2011)<br>Gottschling and McLean (2013)<br>Mertens et al. (2013)<br>Potvin et al. (2013)<br>Gu et al. (2015b)<br>Liu et al. (2015a,b)<br>Mertens et al. (2015b)<br>Gu et al. (2016)<br>Yamaguchi et al. (2016)<br>Efimova et al. (2019)<br>Gurdebek et al. (2020)<br>Gottschling et al. (2021a,b)
biconicum (= Peridinium biconicum)			YP	Gu et al. (2015b)<br>Liu et al. (2015a)<br>Gurdebek et al. (2020)
claudicans<br>isolate #2 AB255842<br>isolate #11 AB255841	Y		Y	Yamaguchi et al. (2006)<br>Gómez et al. (2010a,b)<br>Ribeiro, et al. (2010)<br>Hoppenrath et al. (2012b)<br>Gottschling and McLean (2013)<br>Mertens et al. (2013)<br>Potvin et al. (2013)<br>Sarai et al. (2013)<br>Liu et al. (2015a,b)<br>Mertens et al. (2015b)<br>Gu et al. (2016)<br>Yamaguchi et al. (2016)<br>Gómez et al. (2019b)<br>Reñé et al. (2019)<br>Gurdebek et al. (2020)
conicoides (= Peridinium conicoides)			YP	Gribble and Anderson (2006)<br>Ribeiro, et al. (2010)<br>Yamaguchi et al. (2011)<br>Gu et al. (2015b)

				Liu et al (2015b)<br>Mertens et al. (2015b)<br>Gu et al. (2016)<br>Gurdebek et al. (2020)
conicum (= Peridinium divergens var. conica, Peridinium conicum)	Y		Y	Yamaguchi and Horiguchi (2005)<br>Gribble and Anderson (2006)<br>Kawami et al. (2006)<br>Yamaguchi et al. (2006)<br>Gómez et al. (2010a,b)<br>Ribeiro, et al. (2010)<br>Gomez et al. (2011b)<br>Yamaguchi et al. (2011)<br>Gottschling and McLean (2013)<br>Mertens et al. (2013)<br>Potvin et al. (2013)<br>Gu et al. (2015b)<br>Liu et al. (2015a,b)<br>Mertens et al. (2015b)<br>Gurdebek et al. (2020)
crassipes	Y		Y	Yamaguchi and Horiguchi (2005)<br>Gribble and Anderson (2006)<br>Yamaguchi et al. (2006)<br>Gómez et al. (2009c)<br>Gómez et al. (2010a,b)<br>Ribeiro, et al. (2010)<br>Gomez et al. (2011b)<br>Yamaguchi et al. (2011)<br>Gottschling and McLean (2013)<br>Mertens et al. (2013)<br>Potvin et al. (2013)<br>Gu et al. (2015b)<br>Liu et al (2015b)<br>Mertens et al. (2015b)<br>Boutrup et al. (2016)<br>Gu et al. (2016)<br>Yamaguchi et al. (2016)<br>Gómez et al. (2019b)<br>Efimova et al. (2019)<br>Gurdebek et al. (2020)
denticulatum	Y		YP	Yamaguchi and Horiguchi (2005)<br>Kawami et al. (2006)<br>Yamaguchi et al. (2006)<br>Kim and Kim (2007)<br>Ribeiro, et al. (2010)<br>Gomez et al. (2011b)<br>Yamaguchi et al. (2011)<br>Gottschling and McLean (2013)<br>Mertens et al. (2013)

				Potvin et al. (2013)<br>Gu et al. (2015b)<br>Liu et al (2015b)<br>Mertens et al. (2015b)<br>Gu et al. (2016)<br>Yamaguchi et al. (2016)<br>Reñé and Hoppenrath (2019)<br>Gurdebek et al. (2020)
depressum<br>cell29lg_clone5 EF152847	YP		Y	Gribble and Anderson (2006)<br>Yamaguchi et al. (2006)<br>Kim and Kim (2007)<br>Gómez et al. (2010a,b)<br>Ribeiro, et al. (2010)<br>Yamaguchi et al. (2011)<br>Hoppenrath et al. (2012b)<br>Gottschling and McLean (2013)<br>Mertens et al. (2013)<br>Potvin et al. (2013)<br>Sarai et al. (2013)<br>Liu et al. (2015a,b)<br>Mertens et al. (2015b)<br>Boutrup et al. (2016)<br>Gu et al. (2016)<br>Gómez et al. (2019b)<br>Reñé et al. (2019)<br>Gurdebek et al. (2020)
divaricatum (= Peridinium divaricatum)			YP	Gu et al. (2015b)<br>Liu et al. (2015a)<br>Gurdebek et al. (2020)
divergens (= Peridinium divergens, Ceratium divergens)	YP		YP	Yamaguchi and Horiguchi (2005)<br>Kawami et al. (2006)<br>Yamaguchi et al. (2006)<br>Ribeiro, et al. (2010)<br>Yamaguchi et al. (2011)<br>Hoppenrath et al. (2012b)<br>Gottschling and McLean (2013)<br>Mertens et al. (2013)<br>Potvin et al. (2013)<br>Gu et al. (2015b)<br>Liu et al (2015b)<br>Mertens et al. (2015b)<br>Gu et al. (2016)<br>Yamaguchi et al. (2016)<br>Efimova et al. (2019)<br>Gurdebek et al. (2020)
elegans	YP		YP	Yamaguchi et al. (2006)<br>Gómez et al. (2010a,b)<br>Ribeiro, et al. (2010)

				Gomez et al. (2011b)<br>Yamaguchi et al. (2011)<br>Gottschling and McLean (2013)<br>Mertens et al. (2013)<br>Potvin et al. (2013)<br>Gu et al. (2015b)<br>Liu et al. (2015a,b)<br>Mertens et al. (2015b)<br>Gurdebek et al. (2020)
excentricum (= Peridinium excentricum)	YP	YP	Y	Gribble and Anderson (2006)<br>Yamaguchi et al. (2006)<br>Gómez et al. (2010a,b)<br>Ribeiro, et al. (2010)<br>Gomez et al. (2011b)<br>Yamaguchi et al. (2011)<br>Gottschling and McLean (2013)<br>Mertens et al. (2013)<br>Potvin et al. (2013)<br>Gu et al. (2015b)<br>Liu et al. (2015a,b)<br>Mertens et al. (2015b)<br>Gu et al. (2016)<br>Luo et al. (2018a)<br>Gómez et al. (2019b)<br>Gurdebek et al. (2020)
fukuyoi<br>Brentwood Bay AB780842<br>TY-2003	YP		YP	Liu et al. (2013)<br>Mertens et al. (2013)<br>Liu et al (2015b)<br>Mertens et al. (2015b)<br>Yamaguchi et al. (2016)<br>Potvin et al. (2018)<br>Gottschling et al. (2021b)
fuzhouense			YP	Gu et al. (2015b)<br>Liu et al. (2015a,b)<br>Gurdebek et al. (2020)
glenanicum			YP	Moreira-González et al. (2019)
haizhouense<br>(not clear based on LSU sequence if different species from Protoperidinium americanum or misidentified sequence data, see Reñé et al., 2019)	YA		Y/YA	Liu et al. (2013)<br>Liu et al. (2015a,b)<br>Yamaguchi et al. (2016)<br>Potvin et al. (2018)<br>Reñé et al. (2019)<br>Gurdebek et al. (2020)
humile (= Peridinium humile)			Y	Gu et al. (2015b)<br>Liu et al. (2015a)<br>Gu et al. (2016)<br>Gurdebek et al. (2020)

latidorsale			Y	Sarai et al. (2013)<br>Gurdebek et al. (2020)
latissimum (= Peridinium latissimum, Peridinium pentagonum var. latissimum)			Y	Gu et al. (2015b)<br>Liu et al. (2015a)<br>Gurdebek et al. (2020)
leonis (= Peridinium leonis)	YP		YP	Yamaguchi and Horiguchi (2005)<br>Kawami et al. (2006)<br>Yamaguchi et al. (2006)<br>Kim and Kim (2007)<br>Ribeiro, et al. (2010)<br>Yamaguchi et al. (2011)<br>Gottschling and McLean (2013)<br>Mertens et al. (2013)<br>Potvin et al. (2013)<br>Gu et al. (2015b)<br>Liu et al. (2015a,b)<br>Mertens et al. (2015b)<br>Gurdebek et al. (2020)
lewisiae<br>KM820891	YP		YP	Mertens et al. (2015a)<br>Yamaguchi et al. (2016)<br>Hoppenrath et al. (2017)<br>Potvin et al. (2018)<br>Reñé et al. (2019)
louisianensis (cyst stage designated as trinovantedinium pallidifulvum)			YP	Mertens et al. (2017b)<br>Gurdebek et al. (2020)
monovelum<br>Jinzhou Harbour AB716927	Y		YP	Mertens et al. (2013)<br>Liu et al. (2015a)<br>Mertens et al. (2015b)<br>Gu et al. (2016)<br>Yamaguchi et al. (2016)<br>Potvin et al. (2018)<br>Gómez et al. (2019b)<br>Reñé et al. (2019)<br>Gurdebek et al. (2020)
oblongum (= Peridinium divergens var. oblongum, Peridinium oblongum, Peridinium oceanicum var. oblongum, Peridinium oceanicum f. oblongum)<br>isolate #22 AB255858<br>cell1sm_clone1 EF152890			YP	Gribble and Anderson (2006)<br>Yamaguchi et al. (2006)<br>Kim and Kim (2007)<br>Ribeiro, et al. (2010)<br>Potvin et al. (2013)<br>Liu et al (2015b)<br>Mertens et al. (2015b)<br>Gu et al. (2016)<br>Reñé et al. (2019)
pallidum (= Peridinium pallidum)	YP		YP	Yamaguchi and Horiguchi (2005)<br>Yamaguchi et al. (2006)

				Ribeiro, et al. (2010)<br>Yamaguchi et al. (2011)<br>Hoppenrath et al. (2012b)<br>Gottschling and McLean (2013)<br>Mertens et al. (2013)<br>Potvin et al. (2013)<br>Gu et al. (2015b)<br>Liu et al. (2015a,b)<br>Mertens et al. (2015b)<br>Gurdebek et al. (2020)
panamense			YP	Moreira-González et al. (2019)
paraoblongum			Y	Liu et al. (2013)<br>Sarai et al. (2013)<br>Liu et al. (2015a,b)<br>Gu et al. (2016)<br>Gurdebek et al. (2020)
parthenopes<br>031217-4 AB716915	YP/YA		YP	Mertens et al. (2013)<br>Liu et al. (2015a,b)<br>Mertens et al. (2015b)<br>Yamaguchi et al. (2016)<br>Potvin et al. (2018)<br>Gurdebek et al. (2020)
pellucidum	YP	YP	YP	Gribble and Anderson (2006)<br>Yamaguchi et al. (2006)<br>Kawami et al. (2006)<br>Kim and Kim (2007)<br>Gómez et al. (2010a,b)<br>Ribeiro, et al. (2010)<br>Gomez et al. (2011b)<br>Yamaguchi et al. (2011)<br>Hoppenrath et al. (2012b)<br>Mertens et al. (2013)<br>Potvin et al. (2013)<br>Gu et al. (2015b)<br>Liu et al. (2015a,b)<br>Mertens et al. (2015b)<br>Gu et al. (2016)<br>Yamaguchi et al. (2016)<br>Gómez et al. (2019b)<br>Gurdebek et al. (2020)<br>Gottschling et al. (2021a,b)
pentagonum (= Peridinium pentagonum)	YP		YP	Gómez et al. (2010a,b)<br>Ribeiro, et al. (2010)<br>Gomez et al. (2011b)<br>Yamaguchi et al. (2011)<br>Gottschling and McLean (2013)<br>Mertens et al. (2013)

				Potvin et al. (2013)<br>Gu et al. (2015b)<br>Liu et al. (2015a,b)<br>Mertens et al. (2015b)<br>Yamaguchi et al. (2016)<br>Gurdebek et al. (2020)
punctulatum (= Peridinium punctulatum, Peridinium subinerme var. punctulatum, Peridinium subinerme var. punctulatum)	YP		YP	Yamaguchi and Horiguchi (2005)<br>Kawami et al. (2006)<br>Yamaguchi et al. (2006)<br>Kim and Kim (2007)<br>Ribeiro, et al. (2010)<br>Yamaguchi et al. (2011)<br>Mertens et al. (2013)<br>Potvin et al. (2013)<br>Gu et al. (2015b)<br>Liu et al. (2015a,b)<br>Mertens et al. (2015b)<br>Gu et al. (2016)<br>Gómez et al. (2019b)<br>Gurdebek et al. (2020)
quadrioblongum (var. symmetricum)			YP	Sarai et al. (2013)
shanghaiense			Y	Gu et al. (2015b)<br>Liu et al. (2015a)<br>Gu et al. (2016)<br>Gurdebek et al. (2020)
sp.<br>M064-sm-1 DQ444225			YP	Gu et al. (2015b)<br>Gurdebek et al. (2020)
steidingerae<br>MV0923-PO-cl7 DQ444231	YP		Y	Gribble and Anderson (2006)<br>Ribeiro, et al. (2010)<br>Yamaguchi et al. (2011)<br>Hoppenrath et al. (2012a)<br>Potvin et al. (2013)<br>Sarai et al. (2013)<br>Liu et al. (2015a.b)<br>Mertens et al. (2015b)<br>Gu et al. (2016)<br>Gurdebek et al. (2020)
stellatum (= Peridinium stellatum, Stelladinium stellatum)			YP	Gu et al. (2015b)<br>Liu et al. (2015a,b)<br>Mertens et al. (2015b)<br>Gu et al. (2016)<br>Gurdebek et al. (2020)
thorianum	YP			Yamaguchi and Horiguchi (2005)<br>Ribeiro, et al. (2010)<br>Gomez et al. (2011b)<br>Yamaguchi et al. (2011)

				Mertens et al. (2013)<br>Potvin et al. (2013)<br>Mertens et al. (2015b)
thulesense (= Peridinium thulesense)	Y		YP	Ribeiro, et al. (2010)<br>Yamaguchi et al. (2011)<br>Mertens et al. (2013)<br>Potvin et al. (2013)<br>Gu et al. (2015b)<br>Liu et al. (2015a,b)<br>Mertens et al. (2015b)<br>Gu et al. (2016)<br>Yamaguchi et al. (2016)<br>Efimova et al. (2019)<br>Gurdebek et al. (2020)
tricingulatum AB716918				Reñé et al. (2019)
Psammodinium				
inclinatum (= Thecadinium inclinatum, Sabulodinium inclinatum)	Y/NA		Y	Gómez et al. (2011a).<br>Saburova and Chomérat (2014)<br>Reñé and Hoppenrath (2019)<br>Selina et al. (2019)<br>Selina and Efimova (2020)
Psammosa				
atlantica	YP			Okamoto et al. (2012)
pacifica	YP			Okamoto et al. (2012)
Pseudadenoides				
kofoidii	Y		Y	Gómez et al. (2015a)<br>You et al. (2015)<br>Hoppenrath et al. (2017)<br>Gu et al. (2018)
polypyrenoides	YP		YP	You et al. (2015)<br>Hoppenrath et al. (2017)
Pseudopfiesteria (= Pfiesteria)				
shumwayae<br>Pamlico River AF080098	Y	Y	Y	Saito et al. (2002)<br>Litaker et al. (2005)<br>Seaborn et al. (2006)<br>Litaker et al. (2007)<br>Logares et al. (2007)<br>Coats et al. (2010)<br>Hoppenrath et al. (2012b)<br>Craveiro et al. (2013)<br>Gottschling and McLean (2013)<br>Jeong et al. (2014c)<br>Tillmann et al. (2014)<br>Gómez et al. (2015a)<br>Jung et al. (2015)<br>Kang et al. (2015)<br>Yamada et al. (2015)

				Hoppenrath et al. (2017)<br>Hoppenrath et al. (2020)<br>Li, Z. et al. (2020)
Pseudothecadinium				
campbellii	Y		Y	Efimova et al. (2019)<br>Selina and Efimova (2020)
Ptychodiscus				
noctiluca<br>Sao Sebastiao Channel<br>KU640194	YP			Gómez et al. (2016c)
Pyramidodinium				
atrofuscum	YP			Horiguchi et al. (2017)
spinulosum	YP			Horiguchi et al. (2017)
Pyrocystis				
lunula<br>CCCM 517 AF274274	YP			Hoppenrath and Leander (2007)<br>Saburova and Chomérat (2014)<br>Mertens et al. (2015b)<br>Yamada et al. (2015)
pseudonoctiluca (= Pyrocystis noctiluca)<br>CCMP732 AF022156	YP		YP	Grzebyk et al. (1998)<br>Kawami et al. (2006)<br>Amorim et al. (2013)<br>Gómez et al. (2015a)<br>Yamada et al. (2015)<br>Wakeman et al. (2018a)<br>Gómez and Artigas (2019)<br>Gottschling et al. (2021b)
sp.<br>NY007 LC054939	YP			Yamada et al. (2015)
Pyrodinium				
bahamense<br>(Pyrodinium bahamense likely a species complex contining 2 ribotypes not readily separated by morphology - Mertens et al. 2015c)	YP	YP	Y	Leaw et al. (2005)<br>Hoppenrath and Leander (2007)<br>Iwataki et al. (2007)<br>Howard et al. (2009)<br>Gómez et al. (2010a,b)<br>Amorim et al. (2013)<br>Mertens et al. (2015c)<br>Hoppenrath et al. (2017)<br>Kim and Park (2017)<br>Efimova et al. (2019)<br>Gómez and Artigas (2019)<br>Gottschling et al. (2021a)
bahamense var. compressum			YP	Dolapsakis et al. (2006)<br>Gribble and Anderson (2006)<br>Kim and Kim (2007)<br>Howard et al. (2009)<br>Mertens et al. (2015c)<br>Boutrup et al. (2016)

Pyrophacus				
steinii	YP		YP	Gómez et al. (2010a)<br>Amorim et al. (2013)<br>Li and Shin (2019)
Pyrrhotriadinium				
polyedricum<br>G1	YP	YP	Y	Gómez and Artigas (2019)<br>Li et al. (2019)<br>Li and Shin (2019)<br>Hoppenrath et al. (2020)<br>Gottschling et al. (2021a,b)
Pyxidinopsis				
challengerensis	Y		Y	Zhang et al. (2020)
psilata	Y	YP	Y	Mertens et al. (2017a)<br>Zhang et al. (2020)<br>Gottschling et al. (2021a)
Qia				
lebouriae (= Diplopsalis lebouriae)<br>040817-2 AB716921	YP		Y	Kawami et al. (2006)<br>Gómez et al. (2010a,b)<br>Hoppenrath et al. (2012b)<br>Liu et al. (2015a)<br>Mertens et al. (2015b)<br>Gu et al. (2016)<br>Yamaguchi et al. (2016)<br>Gómez et al. (2019b)<br>Reñé et al. (2019)<br>Gurdebek et al. (2020)
Roscoffia				
capitata	YP			Saldarriaga et al. (2003)<br>Hoppenrath and Leander (2007)<br>Hoppenrath et al. (2007)<br>Gómez et al. (2010a.b)<br>Yamaguchi et al. (2011)<br>Hoppenrath et al. (2012b)<br>Gómez et al. (2015a)<br>Li et al. (2017)<br>Yamaguchi et al. (2018)<br>Gómez et al. (2019b)
Rufusiella				
insignis	YP	YP	YP	Gottschling and McLean (2013)<br>Anglès et al. (2017)
Rhinodinium				
broomeense	YP		YP	Murray et al. (2006)<br>Yamaguchi et al. (2011)<br>Hoppenrath et al. (2012a)<br>Saburova and Chomérat (2014)<br>Kang et al. (2015)<br>Mertens et al. (2015b)

				Saburova and Chomérat (2018)<br>Gottschling et al. (2021b)
Sabulodinium				
undulatum<br>clone 5 DQ975474	Y			Hoppenrath and Leander (2007)<br>Hoppenrath et al. (2007)<br>Alves-De-Souza et al. (2011)<br>Gómez et al. (2011a)<br>Hoppenrath et al. (2012b)<br>Hoppenrath et al. (2013)<br>Saburova and Chomérat (2014)<br>Gómez et al. (2015a)<br>Yamada et al. (2015)<br>Gómez (2016b)<br>Yamamoto et al. (2020)
Scrippsiella				
acuminata (= Calciodinellum faeroense, Scrippsiella trochoidea)<br>GeoB*185<br>GeoB 255<br>GeoB 427<br>GeoJ 011<br>ST-1<br>ST-K	Y	Y	Y	Kim, S.H. et al. (2004)<br>Dolapsakis et al. (2006)<br>Hoppenrath et al. (2017)<br>Kretschmann et al. (2018b)<br>Efimova et al. (2019)<br>Gottschling et al. (2019a)<br>Lee, S.Y. et al. (2019)<br>Žerdoner Čalasan et al. (2019)<br>Hu et al. (2020)<br>Li, Z. et al. (2020)<br>Gottschling et al. (2021b)<br>Kim, H.J. et al. (2021)
cf acuminata<br>GeoB 219	YP	YP	YP	Žerdoner Čalasan et al. (2019)
aff. acuminata<br>GeoB*200<br>GeoB*201<br>GeoB 360<br>NCMA2271	Y	Y	Y	Kretschmann et al. (2018a)<br>Gottschling et al. (2019a)<br>Lee, S.Y. et al. (2019)<br>Žerdoner Čalasan et al. (2019)<br>Kim, H.J. et al. (2021)<br>Gottschling et al. (2021b)
bicarinata<br>GeoB*414<br>GeoB 411<br>GeoB 416<br>GeoB 453<br>GeoB 458	Y	Y	Y	Zinssmeister et al. (2012)<br>Craveiro et al. (2013)<br>Kretschmann et al. (2014)<br>Craveiro et al. (2015)<br>Li et al. (2015)<br>Luo, Z. et al. (2016a)<br>Kretschmann et al. (2018b)<br>Gottschling et al. (2019a)<br>Lee, S.Y. et al. (2019)<br>Žerdoner Čalasan et al. (2019)<br>Li, Z. et al. (2020)<br>Gottschling et al. (2021a,b)

				Kim, H.J. et al. (2021)
donghaienis<br>CS-168<br>CS-297<br>D132<br>GeoB 356<br>GeoB 424<br>SSDH01	YP	Y	YP	Zinssmeister et al. (2011)<br>Gu et al. (2013c)<br>Craveiro et al. (2015)<br>Li et al. (2015)<br>Gottschling et al. (2019a)<br>Lee, S.Y. et al. (2019)<br>Žerdoner Čalasan et al. (2019)<br>Gottschling et al. (2021b)<br>Kim, H.J. et al. (2021)
erinaceus<br>GeoM*533<br>GeoM*534	YP	YP	YP	Kretschmann et al. (2014)<br>Gottschling et al. (2019a)<br>Lee, S.Y. et al. (2019)<br>Žerdoner Čalasan et al. (2019)<br>Gottschling et al. (2021b)
c.f. erinaceus (May contain multiple species)<br>Ribotype 1<br>ITS seq<br>GeoB 470<br>GeoB 471<br><br>Ribotype 1<br>From combination of Kretschmann et al. (2014) and Lee, S.Y. et al. (2019)<br>LSU seq M34-*25/5<br>GeoB 338<br>GeoB 421<br>GeoB 430<br>GeoB*533<br><br>Ribotype 2<br>CCPO2<br>CCPO3<br>GeoB 283<br>GeoB 338<br>GeoB 421<br>GeoB 430<br>GeoB 472<br>GeoB 457<br>GeoB*534<br>SNZ 64<br>SNZ 69<br>SNZ 70<br>SNZ 72<br>SNZ 91<br>SNZ 93	Y	Y	Y	Kretschmann et al. (2014)<br>Kretschmann et al. (2018a,b)<br>Lee, S.Y. et al. (2019)<br>Žerdoner Čalasan et al. (2019)<br>Li, Z. et al. (2020)

Ribotype 3<br>GeoB 147<br>HH200907-1<br>ST-D6<br>34-*25/5				
enormis<br>SSDH21		YP	YP	Gu et al. (2013c)<br>Luo, Z. et al. (2016a)<br>Lee, S.Y. et al. (2019)<br>Kim, H.J. et al. (2021)
hangoei (= Apocalathium malmogiense, Peridinium hangoei) Divergence works for all species examined except A. aciculiferum which has the same ITS1, ITS2, 5.8S, SSU, and partial LSU sequences. Whether or not this is due to a misidentified species could not be determined.<br>SHTV1 AY970662	YP/N	Y/N	Y/N	Gottschling et al. (2005a)<br>Attaran-Fariman and Bolch (2007)<br>Logares et al. (2007)<br>Gómez et al. (2010a,b)<br>Gu et al. (2013a,c)<br>Saburova and Chomérat (2014)<br>Annenkova et al. (2015)<br>Kang et al. (2015)<br>Li et al. (2015)<br>Luo, Z. et al. (2016a)<br>Boutrup et al. (2017)<br>Li et al. (2017)<br>Lee, S.Y. et al. (2019)<br>Hu et al. (2020)<br>Kim, H.J. et al. (2021)
aff. hangoei<br>SSCAP K-0399		Y	Y	Annenkova et al. (2015)<br>Li et al. (2015)
infula (= Calcigonellum indula)<br>D129<br>GeoB 110<br>SZN62	YP	Y	Y	D'Onofrio et al. (1999)<br>Montresor et al. (2003)<br>Gottschling et al. (2005a.b)<br>Attaran-Fariman and Bolch (2007)<br>Gottschling et al. (2008)<br>Zinssmeister et al. (2012)<br>Craveiro et al. (2013)<br>Gu et al. (2013c)<br>Kretschmann et al. (2014)<br>Craveiro et al. (2015)<br>Li et al. (2015)<br>Luo, Z. et al. (2016a)<br>Zhang et al. (2016)<br>Lee, S.Y. et al. (2019)<br>Kim, H.J. et al. (2021)
irregularis<br>SPGX01<br>SPGX02<br>SCBC17<br>SCBC19		Y	YP	Attaran-Fariman and Bolch (2007)<br>Soehner et al (2012)<br>Craveiro et al. (2015)<br>Luo, Z. et al. (2016a)<br>Lee, S.Y. et al. (2019)

				Žerdoner Čalasan et al. (2019)<br>Kim, H.J. et al. (2021)
kirschiae<br>GeoB 408<br>GeoB 432	Y	Y	Y	Zinssmeister et al. (2012)<br>Craveiro et al. (2013)<br>Kretschmann et al. (2014)<br>Craveiro et al. (2015)<br>Li et al. (2015)<br>Luo, Z. et al. (2016a)<br>Lee, S.Y. et al. (2019)<br>Žerdoner Čalasan et al. (2019)<br>Kim, H.J. et al. (2021)
lachrymosa<br>D192<br>GeoB 285<br>GeoB 288<br>IO25-01<br>SLBS1703	Y	Y	Y	D'Onofrio et al. (1999)<br>Montresor et al. (2003)<br>Gottschling et al. (2008)<br>Zinssmeister et al. (2011)<br>Soehner et al (2012)<br>Craveiro et al. (2013)<br>Gu et al. (2013c)<br>Kretschmann et al. (2014)<br>Craveiro et al. (2015)<br>Li et al. (2015)<br>Luo, Z. et al. (2016a)<br>Efimova et al. (2019)<br>Gottschling et al. (2019a)<br>Lee, S.Y. et al. (2019)<br>Žerdoner Čalasan et al. (2019)<br>Gottschling et al. (2021b)<br>Kim, H.J. et al. (2021)
masanensis<br>Geo 277<br>SSMS0908<br>SSMS1807<br>SSND14 EU325947		Y	Y	Gu et al. (2013c)<br>Luo, Z. et al. (2016a)<br>Lee, S.Y. et al. (2019)
nutricula	YP			Grzebyk et al. (1998)<br>Gast and Caron (2001)<br>Gómez et al. (2010a,b)<br>Gómez et al. (2015)<br>Wakeman et al. (2018a)
plana<br>LH006<br>SSFC12<br>SSFC13		Y	Y	Luo, Z. et al. (2016a)<br>Lee, S.Y. et al. (2019)<br>Žerdoner Čalasan et al. (2019)<br>Kim, H.J. et al. (2021)
polonicum	YP			Attaran-Fariman and Bolch (2007)<br>Gómez et al. (2010a,b)
precaria	YP	Y	Y	Gottschling et al. (2005a)

CS-294<br>GeoJ 002<br>G05<br>GeoB 378<br>KWO10818-1D6 DQ847435<br>SPBH01				Gottschling et al. (2008)<br>Gómez et al. (2010a,b)<br>Zinssmeister et al. (2012)<br>Soehner et al (2012)<br>Craveiro et al. (2013)<br>Gu et al. (2013c)<br>Kretschmann et al. (2014)<br>Saburova and Chomérat (2014)<br>Craveiro et al. (2015)<br>Yamada et al. (2015)<br>Luo, Z. et al. (2016a)<br>Li et al. (2017)<br>Efimova et al. (2019)<br>Gottschling et al. (2019a)<br>Lee, S.Y. et al. (2019)<br>Žerdoner Čalasan et al. (2019)<br>Gottschling et al. (2021b)<br>Kim, H.J. et al. (2021)
ramonii<br>GeoB 280<br>GeoB 398<br>SZN7	YP	Y	YP	D'Onofrio et al. (1999)<br>Montresor et al. (2003)<br>Gottschling et al. (2005a)<br>Attaran-Fariman and Bolch (2007)<br>Zinssmeister et al. (2011)<br>Soehner et al (2012)<br>Zinssmeister et al. (2012)<br>Craveiro et al. (2013)<br>Gu et al. (2013c)<br>Kretschmann et al. (2014)<br>Craveiro et al. (2015)<br>Li et al. (2015)<br>Luo, Z. et al. (2016a)<br>Lee, S.Y. et al. (2019)<br>Žerdoner Čalasan et al. (2019)<br>Kim, H.J. et al. (2021)
rotunda<br>SSDH03<br>SSND11<br>SZN66<br>GeoB 280	YP	Y	Y	D'Onofrio et al. (1999)<br>Montresor et al. (2003)<br>Gottschling et al. (2005a)<br>Attaran-Fariman and Bolch (2007)<br>Gottschling et al. (2008)<br>Zinssmeister et al. (2012)<br>Craveiro et al. (2013)<br>Kretschmann et al. (2014)<br>Craveiro et al. (2015)<br>Li et al. (2015)<br>Luo, Z. et al. (2016a)<br>Lee, S.Y. et al. (2019)<br>Žerdoner Čalasan et al. (2019)<br>Kim, H.J. et al. (2021)

sp.<br>D342 HQ845330	YP			Gu et al. (2018)
sp.<br>GeoB*61	YP	YP	YP	Kretschmann et al. (2014)
sp.<br>MH497039<br>GeoB*161 AY499527<br>MG-2014 D066 KJ189483	YP	YP	YP	Kretschmann et al. (2014)
sp.<br>GeoB 243<br>GeoB 245<br>MG-2014 D259 KJ189489	Y	Y	Y	Kretschmann et al. (2014)
sp.<br>GeoB 283<br>D342 HQ845330	YP	YP	YP	Kretschmann et al. (2014)<br>Kretschmann et al. (2018a,b)<br>Žerdoner Čalasan et al. (2019)<br>Gottschling et al. (2021b)
sp.<br>HJ-2013 KM357282<br>SSND14 EU325947	YP	YP	YP	Žerdoner Čalasan et al. (2019)
sp.<br>KJ22-3-60	YP	YP	YP	Žerdoner Čalasan et al. (2019)
sp.<br>NCMA2818	YP	YP	YP	Žerdoner Čalasan et al. (2019)
sp.<br>NY012 LC054940	YP			Yamada et al. (2015)
sp.<br>SCKS 0701 AM494499	YP			Gómez et al. (2010a,b)
sp.<br>SCM38C7 AY664960	YP	YP	YP	Žerdoner Čalasan et al. (2019)
sp.<br>SSND04 EU325944		YP		Gu et al. (2013c)<br>Luo, Z. et al. (2016a)
sp.<br>SSND07 EU325945		YP		Gu et al. (2013c)<br>Luo, Z. et al. (2016a)
sp.<br>ST-D6 EU370959<br>EU370962	YP	YP	YP	Žerdoner Čalasan et al. (2019)<br>Gottschling et al. (2021b)
spinifera<br>NIEHS 684<br>SSHWI01 KT804915<br>KT804919	YP	Y	Y	Luo, Z. et al. (2016a)<br>Efimova et al. (2019)<br>Lee, S.Y. et al. (2019)<br>Žerdoner Čalasan et al. (2019)<br>Kim, H.J. et al. (2021)
cf. spinifera<br>NIES-684<br>SSFC02<br>SSFC03		Y	YP	Gu et al. (2013c)<br>Luo, Z. et al. (2016a)<br>Lee, S.Y. et al. (2019)
sweeneyae<br>CCCM 280 AF274276	YP	Y	YP	Gottschling et al. (2005a,b)<br>Kawami et al. (2006)

D069<br>D161<br>NIES 684				Attaran-Fariman and Bolch (2007)<br>Hoppenrath and Leander (2007)<br>Gottschling et al. (2008)<br>Sparmann et al. (2008)<br>Gómez et al. (2010a,b)<br>Zinssmeister et al. (2011)<br>Hoppenrath et al. (2012b)<br>Zinssmeister et al. (2012)<br>Craveiro et al. (2013)<br>Gottschling and McLean (2013)<br>Gu et al. (2013c)<br>Potvin et al. (2013)<br>Kretschmann et al. (2014)<br>Gómez et al. (2015a)<br>Kang et al. (2015)<br>Li et al. (2015)<br>Mertens et al. (2015b)<br>Yamada et al. (2015)<br>Luo, Z. et al. (2016a)<br>Hoppenrath et al. (2017)<br>Li et al. (2017)<br>Kretschmann et al. (2018a,b)<br>Gottschling et al. (2019a)<br>Lee, S.Y. et al. (2019)<br>Žerdoner Čalasan et al. (2019)<br>Li, Z. et al. (2020)<br>Gottschling et al. (2021a,b)<br>Kim, H.J. et al. (2021)
trifida<br>GeoB*109<br>GeoB*238 AY628427<br>GeoB*290 DQ167866<br>GeoB 434	YP	Y	YP	Gottschling et al. (2005a)<br>Attaran-Fariman and Bolch (2007)<br>Gu et al. (2013c)<br>Kretschmann et al. (2014)<br>Luo, Z. et al. (2016a)<br>Lee, S.Y. et al. (2019)<br>Žerdoner Čalasan et al. (2019)<br>Kim, H.J. et al. (2021)
trochoidea<br>FIU26 EU165286<br>Jinhae AF042819<br>GSW9808 EF613366<br>ST-K EU370961	Y	Y	Y	D'Onofrio et al. (1999)<br>Montresor et al. (2003)<br>Gottschling et al. (2005a)<br>Hoppenrath and Leander (2007)<br>Litaker et al. (2007)<br>Attaran-Fariman and Bolch (2007)<br>Gottschling et al. (2008)<br>Moestrup et al. (2008)<br>Gómez et al. (2010a,b)<br>Tang et al. (2010)<br>Hansen and Daugbjerg (2011)<br>Zinssmeister et al. (2011)

				Attaran-Fariman and Bolch (2012)<br>Hoppenrath et al. (2012b)<br>Nézan et al. (2012)<br>Soehner et al (2012)<br>Craveiro et al. (2013)<br>Gottschling and McLean (2013)<br>Gu et al. (2013c)<br>Potvin et al. (2013)<br>Kretschmann et al. (2014)<br>Gómez et al. (2015a)<br>Kang et al. (2015)<br>Li et al. (2015)<br>Yamada et al. (2015)<br>Luo, Z. et al. (2016a)<br>Hoppenrath et al. (2017)<br>Li et al. (2017)<br>Efimova et al. (2019)<br>Kim, H.J. et al. (2021)
trochoidea<br>ITS/5.8S Ribotype STR1<br><br>GeoB 138 AY499525<br>GeoB 140 AY676152<br>GeoB 188 AY499524<br>GeoB*200 KY996800<br>GeoB*201 AY676158<br>GeoB 214 AY676160<br>GeoB*216 AY728079<br>GeoB 331 HQ729488<br>GeoB 339 JQ422480<br>GeoB 360 HQ729495<br>GeoB 369 JQ422486<br>GeoB 371 JQ422483<br>GeoB 375 JQ422484<br>GeoB 377 HQ729499<br>GeoB*405 JQ422485<br>GeoB 450 JQ422489<br>GeoB 468 JQ422495<br>I0 26-01 AY676163<br>STND01 EU325957<br>STGX01 EU325959<br>STNDO1 EU325957<br>SNZ 76 AF527093-AF527095<br>SZN 77 AF527096-AF527100		Y	Y	Zinssmeister et al. (2011)<br>Zinssmeister et al. (2012)
trochoidea<br>ITS/5.8S ribotype STR2A<br>GeoB 246 KJ189492<br>GeoB 247 KJ189493		Y	Y	Zinssmeister et al. (2011)<br>Soehner et al (2012)<br>Zinssmeister et al. (2012)

GeoB 444<br>GSW9608<br>GeoB 447 KJ189493<br>ST-K EU370964<br>SZN 33 AF527069-AF527070<br>SZN 89 AF527102-AF527108<br>SZN 90 AF527109-AF527115<br><br>ITS/5.8S ribotype STR2B<br>CCAC 3450 = GeoB*185<br>HQ729493<br>CCAP1134/9 FR865630<br>GeoB 210 AY67615<br>GeoB 219 AY676154<br>GeoB*238 AY628427<br>GeoB 251 AY788358<br>GeoB 290 DQ16786<br>GeoB 352 HQ72949<br>GeoB 427 JN982383<br>IO14-01 AY676162<br>IO24-01 AY72808<br>NIES-369 AY499530<br>ST-1 EU370963<br><br>ITS/5.8S ribotype STR2C<br>7 B3 JQ422509<br>7 D4 JQ422510<br>7 F3<br>42 B1 JQ422512<br>42 B10 JQ422513<br>42 07<br>42 D8 JQ422515<br>42 E9 JQ422516<br>42 F7 JQ422517<br>42 EB<br>42 G3 JQ422518<br>1014-01 AY676162<br>AC24-3 JX661036<br>GeoB*185 HQ729493<br>GeoB 186<br>GeoB 204 KJ189491<br>GeoB 269 AY788359<br>GeoB 349<br>GeoB 352 HQ729491<br>NIES 369 AY499530<br>RCC 3006 JX661021<br>VGOS3V AM998537				
trochoidea		Y	Y	Zinssmeister et al. (2011)

ITS/5.8S ribotype STR3<br>1008C JQ422481<br>Florida 1008B HQ729494<br>GeoB*64 JQ422487<br>GeoB 111 AY800132<br>GeoB 122 AY676146<br>GeoB 147 HQ729483<br>GeoB*165 AY676147<br>GeoB 199 AY676149<br>GeoB*205 HQ729485<br>GeoB 283 HQ845330<br>GeoB 311 JQ422482<br>GeoB 338 HQ729490<br>GeoB*362 HQ729496<br>GeoB 421 HQ729501<br>GeoB 430 JN982379<br>GeoB 457 JQ422488<br>GeoB 462 JQ422492<br>GeoB 470 JQ422490<br>GeoB 471 JQ422491<br>GeoB 472 JQ422493<br>GeoB 473 JQ422494<br>M34-*25/5 AY499531<br>SCPC18 EF584454<br>SCPC36 EF584455<br>SCPC39 EF584458<br>SCPC51 EF584457<br>SCPC73 EF584456<br>STXM01 EU325956<br>STND02 EU325958<br>SZN 61 AF527074-AF527076<br>SZN 64 AF527079-AF527081<br>SZN 69 AF527082-AF527084<br>SZN 70 AF527085-AF527089<br>SZN 72 AF527090-AF527092<br>SZN 82 AF527101<br>SZN 91 AF527116-AF527119<br>SZN 93 AF527120-AF527121				Zinssmeister et al. (2012)<br>Soehner et al (2012)
trochoidea cf. trochoidea<br>NCMA2271	YP	Y	YP	D'Onofrio et al. (1999)<br>Tang et al. (2010)<br>Zinssmeister et al. (2011)<br>Žerdoner Čalasan et al. (2019)
trochoidea cf. / var. aciculifera<br>D141<br>GeoB*160 AY499526<br>GeoB*213 MH497041<br>SCCAP499<br>AF527061-AF527068	YP	YP/YA	YP	D'Onofrio et al. (1999)<br>Montresor et al. (2003)<br>Hansen and Daugbjerg (2004)<br>Gottschling et al. (2005a)<br>Attaran-Fariman and Bolch (2007)<br>Zinssmeister et al. (2012)

SZN 60 AF527071-AF527073<br>SZN 63 AF527077-AF527078				Gu et al. (2013c)<br>Kretschmann et al. (2014)<br>Luo, Z. et al. (2016a)<br>Žerdoner Čalasan et al. (2019)<br>Kim, H.J. et al. (2021)
Shimiella				
gracilenta	YP			Ok et al. (2020)
Sinophysis				
ebriola	YP			Gómez et al. (2012b)<br>Hoppenrath et al. (2013)<br>Gómez et al. (2015a)<br>Wakeman et al. (2018a)
grandis	Y			Gómez et al. (2012b)<br>Hoppenrath et al. (2013)
microcephala	YP			Hoppenrath et al. (2013)
stenosoma	Y			Hoppenrath et al. (2013)
verruculosa	YP			Hoppenrath et al. (2013)
Sourniaea				
diacantha (=Amylax diacantha, Gonyaulax verior)	Y	YP	Y	Kim and Kim (2007)<br>Gómez et al. (2010a,b)<br>Efimova et al. (2019)<br>Selina and Efimova (2020)<br>Zhang et al. (2020)<br>Gottschling et al. (2021a)
Spatulodinium				
pseudonoctiluca	Y			Gómez et al. (2010b)<br>Cooney et al. (2020)
sp.<br>FG541	YP			Gómez et al. (2010b)<br>Cooney et al. (2020)
Sphaerodinium				
cracoviense			YP	Craveiro et al. (2010)<br>Jeong et al. (2014c)<br>Takahashi et al. (2015)
polinicum var. tatricum		YP	YP	Pandeirada et al. (2021)
Spiniferites				
belerius	YP	YP	YP	Mertens et al. (2017a)<br>Zhang et al. (2020)<br>Gottschling et al. (2021a)
Spiniferodinium				
galeiforme (taxon requires further investigation)<br>TM57 LC054941	Y	YP	Y	Horiguchi et al. (2011)<br>Gómez and Skovgaard (2014)<br>Horiguchi et al. (2017)<br>Na et al. (2017)<br>Saburova and Chomérat (2014)<br>Takano et al. (2014)<br>Yamada et al. (2015)<br>Luo et al. (2018b)

				Wakeman et al. (2018a)<br>Gómez et al. (2019a)<br>Romeikat et al. (2019)<br>Žerdoner Čalasan et al. (2019)<br>Gottschling et al. (2021b)
limneticum<br>GeoM*505<br>GeoM*517	Y	YP	Y	Kretschmann et al. (2015)<br>Na et al. (2017)<br>Luo et al. (2018b)<br>Romeikat et al. (2019)<br>Žerdoner Čalasan et al. (2019)<br>Gottschling et al. (2021a,b)
palauense<br>GeoM*719<br>Hokkaido, Lake Shikotsu<br>AB921299<br>AB921300<br>PA-041	YP	YP	YP	Horiguchi et al. (2011)<br>Kretschmann et al. (2015)<br>Horiguchi et al. (2017)<br>Na et al. (2017)<br>Luo et al. (2018b)<br>Gómez et al. (2019a)<br>Žerdoner Čalasan et al. (2019)<br>Gottschling et al. (2021b)
sp.<br>PA-034	Y	Y	Y	Kretschmann et al. (2015)<br>Romeikat et al. (2019)<br>Žerdoner Čalasan et al. (2019)
Stoeckeria				
algicida<br>SAMS07<br>Sa2YD<br>SaZY<br>Shephard's crook St. Lucie River AY590484<br>South Korea:Masan AJ841809	Y	Y	Y	Jeong et al. (2005)<br>Litaker et al. (2005)<br>Coats et al. (2010)<br>Gottschling et al. (2012)<br>Craveiro et al. (2013)<br>Jeong et al. (2014c)<br>Kretschmann et al. (2014)<br>Kang et al. (2015)<br>Gómez et al. (2015a)<br>Jung et al. (2015)<br>Kretschmann et al. (2018b)<br>Žerdoner Čalasan et al. (2019)<br>Hoppenrath et al. (2020)<br>Li, Z. et al. (2020)<br>Gottschling et al. (2021b)
changwonensis<br>Shephard's crook Trout River AY590479<br>SSSC09 HG005132<br>SSMS0806vv FN557541	Y	YP	Y	Gottschling et al. (2012)<br>Craveiro et al. (2013)<br>Jeong et al. (2014c)<br>Kretschmann et al. (2014)<br>Kang et al. (2015)<br>Kretschmann et al. (2018b)<br>Žerdoner Čalasan et al. (2019)<br>Hoppenrath et al. (2020)<br>Gottschling et al. (2021b)

sp.<br>SSSC09 FN557541	Y	Y	Y	Gottschling et al. (2012)<br>Craveiro et al. (2013)<br>Gu et al. (2013a)<br>Kretschmann et al. (2014)<br>Li, Z. et al. (2020)
Stylodinium				
littorale<br>NY017 LC054942	YP			Yamada et al. (2015)<br>Gómez et al. (2019a)
Syltodinium				
listii	Y		YA	Gómez et al. (2019a)
undulans (= Gyrodinium undulans)			YA	Gómez et al. (2019a)
Symbiodinium (Clade A)				
SSU sequences that do not clearly map directly to species identified using ITS sequences)				Strychar et al. (2005)
clade A (not delineated into speices)	Y	Y	Y	LaJeunesse (2001)<br>Jeong et al. (2014a)<br>Hehenberger et al. (2017)<br>Jang et al. (2017a,b)<br>Rodríguez et al. (2019)
bemudense (clade A)			YP	Siano et al. (2010)<br>Jeong et al. (2014a)<br>Jang et al. (2017a,b)
californium (clade A)<br>ex Anthopleura elegantissima<br>AF334659<br>AF225965	YP	Y		Shao et al. (2004)<br>Strychar et al. (2005)<br>Yamada et al. (2015)<br>Rodríguez et al. (2019)
linuchae (clade A4) (= Gymnodinium linuchae) (clade A)<br>A4<br>clone 368 AF060893<br>FGB05_04 KF364602		Y	YP	LaJeunesse (2001)<br>Guillou et al. (2002)<br>Litaker et al. (2007)<br>Siano et al. (2010)<br>Jeong et al. (2014a,b)<br>Teschima et al. (2019)
microadriaticum (clade A1.1)<br>M88521<br>clone 61 AF060896<br>rt-272 KF364600	YP	Y	Y	Grzebyk et al. (1998)<br>LaJeunesse (2001)<br>Edvardsen et al. (2003)<br>Kawami et al. (2006)<br>Litaker et al. (2007)<br>Sparmann et al. (2008)<br>Howard et al. (2009)<br>Gómez et al. (2010a,b)<br>Siano et al. (2010)<br>Hoppenrath et al. (2012b)<br>Yoon et al. (2012)<br>Gottschling and McLean (2013)

				Jeong et al. (2014a)<br>Probert et al. (2014)<br>Lee, S.Y. et al. (2015)<br>Yamada et al. (2015)<br>Gómez et al. (2017a)<br>Jang et al. (2017a,b)<br>LaJeunesse (2017)<br>Wakeman et al. (2018a)<br>LaJeunesse et al. (2018)<br>Teschima et al. (2019)
muscatinei (clade B4)<br>Considered an invalid name, but still unique ribotype. See LaJeunesse (2001).		Y		LaJeunesse (2001)
natans (clade A)<br>Tenerife, Canary Islands<br>EU315917		Y	Y	Siano et al. (2010)<br>Hansen and Daugbjerg (2011)<br>Jeong et al. (2014a,c)<br>Moestrup et al. (2014)<br>Boutrup et al. (2016)<br>Luo et al. (2016b)<br>Jang et al. (2017a,b)<br>Li et al. (2017)<br>LaJeunesse et al. (2018)
necroappetens (clade A) (= Zooxanthella necroappetens)		Y	Y	LaJeunesse et al. (2015)<br>LaJeunesse et al. (2018)
pilosum (clade A2)<br>X62650, Same SSU sequences as Symbiodinium pilosum also identified as Symbiodinium meandrinae and S. corculorum, both of which are considered invalid names.<br>rt-185 AF060894<br>rt-185 KF740671	Y	Y	YP	Sadler et al. (1992)<br>McNally et al. (1994)<br>Grzebyk et al. (1998)<br>LaJeunesse (2001)<br>Edvardsen et al. (2003)<br>Kawami et al. (2006)<br>Siano et al. (2010)<br>Yoon et al. (2012)<br>Jeong et al. (2014a,b)<br>Probert et al. (2014)<br>Gómez et al. (2015a)<br>Lee, S.Y. et al. (2015)<br>LaJeunesse et al. (2015)<br>Hehenberger et al. (2017)<br>Jang et al. (2017a,b)<br>LaJeunesse et al. (2018)<br>Wakeman et al. (2018a)
pulchrorum (Clade B1)<br>Considered an invalid name, but still unique ribotype. See LaJeunesse (2001)	Y	YP	Y	McNally et al. (1994)<br>Grzebyk et al. (1998)<br>LaJeunesse (2001)<br>Shao et al. (2004)<br>Siano et al. (2010)

				Jang et al. (2017a)
sp. (clade A)<br>NCMA2456	YP		YP	Jeong et al. (2014a)<br>Luo et al. (2016b)<br>Gottschling et al. (2021a,b)
toratum (clade A)	YP		YP	Jeong et al. (2014a)
sp.<br>HG193 AB863030	YP			Yamada et al. (2015)
sp.<br>NY010 AB863031	YP			Yamada et al. (2015)
sp. (clade A)<br>ex Millepora sp. AJ311946			YP	Siano et al. (2010)
sp. (clade A)<br>ex Porites astreodies<br>AY074967			YP	Siano et al. (2010)
tridacnidarum (clade A3) (= Zooxanthella tridacnidarum)		Y	Y	Jeong et al. (2014b)<br>Lee, S.Y. et al. (2015)<br>LaJeunesse et al. (2018)<br>Teschima et al. (2019)
sp.<br>Clade D Foraminifera			Y	LaJeunesse et al. (2018)
clade F (SSU and ITS/LSU sequences from different isolates so hard to match)	Y			Takishita et al. (2003)<br>Siano et al. (2010)<br>Jeong et al. (2014a)<br>Hehenberger et al. (2017)<br>Jang et al. (2017b)
clade Fr2 (clade F sensu stricto)<br>AJ830908<br>AJ830912<br>ex. Sorites sp. AJ830911		Y	Y	Siano et al. (2010)<br>Jeong et al. (2014b)<br>LaJeunesse et al. (2018)<br>Shi et al. (2020)
clade Fr3 (clade F sensu stricto)<br>ex. Marginopora kudakajimaensis<br>AJ308895<br>Amphisorus hemprichii<br>AJ830916		Y	Y	Siano et al. (2010)<br>Jeong et al. (2014b)<br>LaJeunesse et al. (2018)<br>Teschima et al. (2019)<br>Shi et al. (2020)
clade Fr4 (clade F sensu stricto)		Y	Y	Jeong et al. (2014b)<br>LaJeunesse et al. (2018)<br>Shi et al. (2020)
sp. (clade F)<br>ex Sorites sp. AJ621139				Siano et al. (2010)
Symbiodinium clade G - isolated from foraminifera (likely represent different species)<br>sp. 1582 ex. Amphisorus sp.			YP	Jeong et al. (2014b)<br>LaJeunesse et al (2018)

AJ291537<br>sp. 1584 ex. Amphisorus sp. AJ291539<br>sp. 1645 ex. Marginopora vertebralis AJ291538				
clade H<br>ex. Sorites sp. AJ291513<br>ex. Sorites sp. AJ291520<br>ex. Sorites sp. AJ621129<br>ex. Sorites sp. AJ621148<br>ex. Marginopora vertebralis AJ830905		Y	Y	Siano et al. (2010)<br>Jeong et al. (2014b)<br>LaJeunesse et al. (2018)
clade I<br>nr-i4 ex. Soritid Foraminifera FN561562<br>nr-i1 ex. Soritid Foraminifera FN561559		Y	Y	Jeong et al. (2014b)<br>LaJeunesse et al. (2018)<br>Shi et al. (2020)
Syndinium				
sp.<br>ex. Corycaeus sp. DQ146406	YA	Y		Skovgaard et al. (2005)<br>Harada et al. (2007)<br>Small et al. (2011)<br>Kim and Park (2014)<br>Wakeman et al. (2018b)
turbo<br>ex. Paracalanus parvus<br>DQ146403<br>DQ146404<br>DQ146405	YP	Y	YP	Skovgaard et al. (2005)<br>Harada et al. (2007)<br>Skovgaard et al. (2007)<br>Guillou et al. (2008)<br>Gómez et al. (2010a)<br>Kim and Park (2014)<br>Gómez and Skovgaard (2015)<br>Jung et al. (2015)<br>Gómez (2016b)<br>Gómez et al. (2017a)<br>Li et al. (2017)<br>Gómez et al. (2019a)
Takayama				
acrotrocha<br>GT7 DQ656115<br>DQ656117<br>GT15 DQ656116<br>CCMP2960 HQ834208<br>MC728-D5 HM067010	YP	YA	Y	Siano et al. (2009)<br>Hansen and Daugbjerg (2011)<br>Nézan et al. (2012)<br>Qiu et al. (2013)<br>Moestrup et al. (2014)<br>Reñé et al. (2014)<br>Reñé et al. (2015)<br>Baytut et al. (2016)<br>Boutrup et al. (2016)<br>Luo et al. (2016b)<br>Gómez et al. (2017a)

				Li et al. (2017)<br>Annenkova (2018)<br>Wang et al. (2018)<br>Benico et al. (2019)<br>Gómez et al. (2019a)<br>Benico et al. (2020)<br>Ok et al. (2020)<br>Cen et al. (2021)<br>Gottschling et al. (2021a,b)
helix			Y	de Salas et al. (2003)<br>Haywood et al. (2004)<br>Bergholtz et al. (2005)<br>de Salas et al. (2005a,b)<br>Bergholtz et al. (2006)<br>Kim and Kim (2007)<br>de Salas et al. (2008)<br>Siano et al. (2009)<br>Qiu et al. (2013)<br>Baytut et al. (2016)<br>Wang et al. (2018)<br>Benico et al. (2020)<br>Ok et al. (2020)<br>Cen et al. (2021)
pulchella (= Gymnodinium pulchellum)	YP	Y	YP	Garcés<br>Jeong et al. (2014c)<br>Saburova and Chomérat (2014)<br>Reñé et al. (2015)
cf. pulchellum (= cf. Gymnodinium pulchellum)	YP		YP	Sparmann et al. (2008)<br>Hoppenrath et al. (2012b)<br>Miranda et al. (2012)<br>Gómez et al. (2015b)
sp.<br>IEO-VG034 AM183261		YP		Wang et al. (2018)<br>Cen et al. (2021)
sp. France Atlantic<br>IFR10-074 KJ508378<br>IFR12-234 KJ508379			Y	Nézan et al. (2014)
sp.<br>IEO-VGO341 AM183261		YP		Li and Shin (2018)
sp.<br>Kawau Isand, New Zealand<br>U92254			YP	de Salas et al. (2003)<br>Haywood et al. (2004)<br>de Salas et al. (2005a)<br>de Salas et al. (2008)
sp.<br>China			YP	Haywood et al. (2004)<br>de Salas et al. (2008)
tasmanica (if one sequence misidentified as T. tuberculata			Y	de Salas et al. (2003)<br>Haywood et al. (2004)<br>Bergholtz et al. (2005)

then there is no ambiguity among LSU sequences)				de Salas et al. (2005a,b)<br>Bergholtz et al. (2006)<br>Kim and Kim (2007)<br>de Salas et al. (2008)<br>Siano et al. (2009)<br>Nézan et al. (2012)<br>Qiu et al. (2013)<br>Jeong et al. (2014c)<br>Nézan et al. (2014)<br>Saburova and Chomérat (2014)<br>Reñé et al. (2015)<br>Wang et al. (2018)<br>Benico et al. (2019)<br>Benico et al. (2020)
tuberculata (one sequence may be misidentified, actually tasmanica)			YP/YA	Haywood et al. (2004)<br>de Salas et al. (2008)<br>Siano et al. (2009)<br>Hansen and Daugbjerg (2011)<br>Hoppenrath et al. (2012a)<br>Moestrup et al. (2014)<br>Nézan et al. (2014)<br>Pandeirada et al. (2014)<br>Baytut et al. (2016)<br>Boutrup et al. (2016)<br>Luo et al. (2016b)<br>Zhang et al. (2016)
xiamenensis	YP	Y	Y	Nézan et al. (2014)<br>Li and Shin (2018)<br>Wang et al. (2018)<br>Benico et al. (2019)<br>Takahashi et al. (2019)<br>Benico et al. (2020)<br>Ok et al. (2020)<br>Cen et al. (2021)
Testudodinium				
corrugatum<br>HG163 AB704004<br>TM-85 AB704003	Y			Horiguchi et al. (2012)<br>Yamada et al. (2013)<br>Saburova and Chomérat (2014)<br>Yamada et al. (2015)<br>Pinto et al. (2017)
maedaense<br>MAE-18 AB704005	YP			Horiguchi et al. (2012)<br>Yamada et al. (2013)<br>Gómez et al. (2015a)<br>Pinto et al. (2017)<br>Yamada et al. (2015)<br>Wakeman et al. (2018a)<br>Yamamoto et al. (2020)

magnum	YP			Pinto et al. (2017)
sp.<br>HG230 LC054943	YP			Yamada et al. (2015)
testudo<br>KOM-30 AB704002	YP			Horiguchi et al. (2012)<br>Saburova and Chomérat (2014)<br>Gómez et al. (2015a)<br>Pinto et al. (2017)<br>Yamamoto et al. (2020)
Thalassicolla (radiolarian parasite species)	Y			
unknown species affiliation<br>BBSRP1ex Thalassicolla nucleata DQ116021<br>DQ116022				Gast (2006)
Thecadinium				
kofoidii (= Phalacroma kofoidi, Thecadinium kofoidi, Thecadinium petasatum)<br>GU295204	Y	Y	Y	Hoppenrath et al. (2004)<br>Hoppenrath and Leander (2007)<br>Gómez et al. (2010a,b)<br>Hoppenrath et al. (2012b)<br>Potvin et al. (2013)<br>Yamada et al. (2015)<br>Boutrup et al. (2016)<br>Gómez (2016b)<br>Hoppenrath et al. (2017)<br>Yamaguchi et al. (2016)<br>Gu et al. (2018)<br>Salgado et al. (2018)<br>Efimova et al. (2019)<br>Reñé and Hoppenrath (2019<br>Selina et al. (2019)<br>Selina and Efimova (2020)<br>Zhang et al. (2020)<br>Gottschling et al. (2021a,b)
cf. kofoidii<br>Th.cf.k-2	YP	YP	YP	Gottschling et al. (2021a,b)
ovatum			YP	Reñé and Hoppenrath (2019)
pseudokofoidii	Y	Y	Y	Efimova et al. (2019)<br>Reñé and Hoppenrath (2019)<br>Selina et al. (2019)<br>Selina and Efimova (2020)
yashimaense (= Thecadinium mucosum) some sequences misidentified as other species. e.g.<br>EF492515<br>DQ388458<br>AY238477	Y		Y	Hoppenrath et al. (2004)<br>Hoppenrath and Leander (2007)<br>Gómez et al. (2010a,b)<br>Gómez et al. (2011a)<br>Hoppenrath et al. (2012b)<br>Potvin et al. (2013)<br>Saburova and Chomérat (2014)

				Gómez (2016b)<br>Yamaguchi et al. (2016)<br>Yamaguchi et al. (2018)<br>Efimova et al. (2019)<br>Gómez et al. (2019b)<br>Reñé and Hoppenrath (2019)<br>Selina et al. (2019)<br>Selina and Efimova (2020)<br>Gottschling et al. (2021b)
Theleodinium				
calcisporum<br>MP69	YP	YP	YP	Craveiro et al. (2013)<br>Craveiro et al. (2015)<br>Craveiro et al. (2016)<br>Luo, Z. et al. (2016a)<br>Hoppenrath et al. (2017)<br>Kretschmann et al. (2018a,b)<br>Saburova and Chomérat (2018)<br>Gottschling et al. (2019a)<br>Žerdoner Čalasan et al. (2019)<br>Li, Z. et al. (2020)<br>Gottschling et al. (2021a,b)<br>Kim, H.J. et al. (2021)
Thoracosphaera				
heimii<br>CCCM670 AF274278<br>HG252 LC054944	YP	Y	YP	Gottschling et al. (2005a,b)<br>Gottschling et al. (2008)<br>Hansen and Daugbjerg (2011)<br>Hoppenrath et al. (2012b)<br>Craveiro et al. (2013)<br>Gottschling and McLean (2013)<br>Gu et al. (2013a)<br>Saburova and Chomérat (2014)<br>Craveiro et al. (2015)<br>Kang et al. (2015)<br>Yamada et al. (2015)<br>Luo et al. (2016b)<br>Hoppenrath et al. (2017)<br>Kretschmann et al. (2018b)<br>Žerdoner Čalasan et al. (2019)<br>Tillman et al. (2020)<br>Li, Z. et al. (2020)<br>Gottschling et al. (2021a,b)<br>Kim, H.J. et al. (2021)
Tintinnophagus				
acutus<br>ex. Tintinnopsis cylindrica<br>HM483397	YP	YP	YP	Coats et al. (2010)<br>Gottschling et al. (2012)<br>Craveiro et al. (2013)<br>Gottschling and McLean (2013)<br>Gu et al. (2013a)

				Kim and Park (2014)<br>Kang et al. (2015)<br>Jung et al. (2015)<br>Hoppenrath et al. (2017)<br>Kretschmann et al. (2018b)<br>Žerdoner Čalasan et al. (2019)<br>Hoppenrath et al. (2020)<br>Li, Z. et al. (2020)<br>Gottschling et al. (2021a,b)
Togula				
britannica (= Amphidinium britannicum)<br>AY443010	YA		YP	Hansen and Daugbjerg (2004)<br>Jørgensen et al. (2004a,b)<br>Murray et al. (2005)<br>Tamura et al. (2005)<br>Hoppenrath and Leander (2007)<br>Kim and Kim (2007)<br>Moestrup et al. (2008)<br>Sparmann et al. (2008)<br>Hoppenrath et al. (2009)<br>Hansen and Daugbjerg (2011)<br>Yamaguchi et al. (2011)<br>Hoppenrath et al. (2012a)<br>Jeong et al. (2012)<br>Saburova and Chomérat (2014)<br>Gómez et al. (2015a)<br>Reñé et al. (2015)<br>Yamada et al. (2015)<br>Boutrup et al. (2016)<br>Luo et al. (2016b)
compacta			YP	Hansen and Daugbjerg (2004)<br>Jørgensen et al. (2004b)<br>Kim and Kim (2007)<br>Hoppenrath et al. (2009)<br>Jeong et al. (2012)
jolla (= Amphidinium corpulentum)<br>UTEX LB 1562 AF274252	YA	YP	YP	Jørgensen et al. (2004b)<br>Hansen and Daugbjerg (2004)<br>Murray et al. (2005)<br>Tamura et al. (2005)<br>Moestrup et al. (2008)<br>Sparmann et al. (2008)<br>Hansen and Daugbjerg (2011)<br>Jeong et al. (2012)<br>Jeong et al. (2014c)<br>Saburova and Chomérat (2014)<br>Reñé et al. (2015)<br>Yamada et al. (2015)<br>Boutrup et al. (2016)<br>Luo et al. (2016b)

				Wakeman et al. (2018a)<br>Gottschling et al. (2021a,b)
Tovellia				
aveirensis / cf. aveirensis TSJL01	YP	YP	Y	Pandeirada et al. (2014)<br>Li, Z. et al. (2015)<br>Takahashi et al. (2015)<br>Luo et al. (2016b)<br>Zhang et al. (2016)<br>Pandeirada et al. (2017)<br>Pandeirada et al. (2019)<br>Gottschling et al. (2021a,b)
coronata (= Gymnodinium coronatum)	Y		Y	Lindberg et al. (2005)<br>Calado et al. (2006)<br>Moestrup et al. (2007)<br>Moestrup et al. (2008)<br>Moestrup et al. (2009a)<br>Hansen and Daugbjerg (2011)<br>Jeong et al. (2014c)<br>Pandeirada et al. (2014)<br>Li, Z. et al. (2015)<br>Takahashi et al. (2015)<br>Boutrup et al. (2016)<br>Luo et al. (2016b)<br>Zhang et al. (2016)<br>Pandeirada et al. (2017)<br>Takahashi et al. (2017)<br>Pandeirada et al. (2019)
diexiensis	YP		YP	Zhang et al. (2016))
paldangensis			Y	Li, Z. et al. (2015)<br>Takahashi et al. (2015)<br>Luo et al. (2016b)<br>Zhang et al. (2016)<br>Takahashi et al. (2017)<br>Pandeirada et al. (2019)
rinoi			YP	Pandeirada et al. (2017)<br>Pandeirada et al. (2019)
rubescens			YP	Pandeirada et al. (2019)
sanguinea (= Glenodinium pulvisculus var. oculatum, Glenodinium sanguineum)			Y	Calado et al. (2006)<br>Moestrup et al. (2007)<br>Moestrup et al. (2008)<br>Moestrup et al. (2009a)<br>Hansen and Daugbjerg (2011)<br>Jeong et al. (2014c)<br>Pandeirada et al. (2014)<br>Li, Z. et al. (2015)<br>Takahashi et al. (2015)<br>Boutrup et al. (2016)

				Luo et al. (2016b)<br>Zhang et al. (2016)<br>Pandeirada et al. (2017)<br>Takahashi et al. (2017)<br>Pandeirada et al. (2019)
sp.<br>HBI:SC201101a JQ639766	YP	YP	YP	Gottschling et al. (2021a,b)
sp.<br>HBI:HB200905a JQ639765	YP			Zhang,Q. et a;
Torodinium				
robustum	Y	YP	Y	Reñé et al. (2015)<br>Boutrup et al. (2016)<br>Gómez et al. (2016a,b)<br>Takahashi et al. (2019)
teredo	Y	YP	YP	Reñé et al. (2015)<br>Boutrup et al. (2016)<br>Gómez et al. (2016a,b)<br>Takahashi et al. (2019)
Torquentidium				
convolutum (= Cochlodinium convolutum)	YP		Y	Shin et al. (2019)<br>Hu et al. (2020)<br>Shin and Matsuoka (2020)
flavescens (= Gyrodinium flavescens)			YP	Hu et al. (2020)<br>Shin and Matsuoka (2020)
helix (= Cochlodinium helix)			YP	Reñé et al. (2013)
pirum (= Pseliodinium pirum)	YP	YP	YP	Hu et al. (2019)<br>Gottschling et al. (2021a)
Triadinium				
polyedricum	YP		Y	Shin et al. (2016)<br>Saburova and Chomérat (2018)<br>Reñé and Hoppenrath (2019)
Tripos (= Neoceratium, Ceratium)				
arietinus (= Ceratium arietinum, Neoceratium arietinum)	YP			Gómez et al. (2010c)
balechii (= Ceratium balechii, Neoceratium balechii)	YP		YP	Efimova et al. (2019)
candelabrum (= Ceratium azoricum, Neoceratium azoricum)	Y			Accattatis et al. (2020)<br>Gómez et al. (2010c)
concilians (= Ceratium, Neoceratium concilians)	Y			Accattatis et al. (2020)<br>Gómez et al. (2010c)
contrarius (= Ceratium tripos var. contrarium, Ceratium contrarium, Ceratium trichoceros var. contrarium, Neoceratium contrarium)	YP			Gómez et al. (2010c)

declinatus (= Ceratium declinatum, Neoceratium declinatum)	YP			Gómez et al. (2010c)
digitatum (= Ceratium digitatum, Neoceratium digitatum)				Gómez et al. (2010c)
extensus (= Ceratium fusus var. extensum, Ceratium extensum, Neoceratium extensum)	YP			Grzebyk et al. (1998)<br>Kawami et al. (2006)<br>Gómez et al. (2010c)
euarcuatus (= Ceratium euarcuatum, Neoceratium euarcuatum)	YP			Gómez et al. (2010c)
furca (= Ceratium furca, Neoceratium furca)	YP	YP	YP	Dolapsakis et al. (2006)<br>Gómez et al. (2010a,b,c)<br>Kim and Park (2014)<br>Gómez and Artigas (2019)<br>Accattatis et al. (2020)<br>Gottschling et al. (2021a,b)
fusus (= Ceratium fusus, Neoceratium fuscus)	Y		YP	Daugbjerg et al. (2000)<br>Hansen and Daugbjerg (2004)<br>Jørgensen et al. (2004a)<br>Dolapsakis et al. (2006)<br>Kim and Kim (2007)<br>Moestrup et al. (2008)<br>Gómez et al. (2010c)<br>Yamaguchi et al. (2011)<br>Yoon et al. (2012)<br>Gómez et al. (2015a)<br>Kim and Park (2017)<br>Gómez and Artigas (2019)<br>Accattatis et al. (2020)<br>Zhang et al. (2020)
gravidius (= Ceratium gravidum, Poroceratium gravidum, Neoceratium gravidum)	YP			Gómez et al. (2010c)
hexacanthus (= Ceratium hexacanthum)	YP			Gómez et al. (2010c)<br>Accattatis et al. (2020)
horridus (= Ceratium tripos var. horridum, Ceratium horridum, Neoceratium horridum, considered invalid)	YP			Gómez et al. (2010c)<br>Accattatis et al. (2020)
kofoidii (= Ceratium kofoidii, Neoceratium kofoidii)	YP			Gómez et al. (2010c)<br>Accattatis et al. (2020)

	limulus (= Ceratium limulus, Ceratium tripos var. limulus, Neoceratium limulus)	Y			Gómez et al. (2010c)
	lineatus (= Peridinium lineatum, Ceratium lineatum, Biceratium lineatum, Neoceratium lineatum)	YP		YP	Daugbjerg et al. (2000)<br>Hansen and Daugbjerg (2004)<br>Jørgensen et al. (2004a)<br>Murray et al. (2005)<br>Dolapsakis et al. (2006)<br>Gribble and Anderson (2006)<br>Kim and Kim (2007)<br>Moestrup et al. (2008)<br>Zhang et al. (2020)
	longipes (= Ceratium arcticum var. longipes, Peridinium longipes, Ceratium longipes, Neoceratium longipes) NCMA1770	YP	YP	YP	Gómez et al. (2010c)<br>Gottschling et al. (2012)<br>Accattatis et al. (2020)<br>Gottschling et al. (2021a,b)
	massiliensis (= Ceratium tripos var. massiliense, Ceratium tripos f. massiliense, Ceratium massiliense, Neoceratium massiliense)	YP		YP	Gómez et al. (2010c)<br>Accattatis et al. (2020)<br>Selina and Efimova (2020)
	minutus (= Ceratium minutum, Neoceratium minutum)	YP			Gómez et al. (2010c)<br>Accattatis et al. (2020)
	muelleri (= Ceratium tripos, Peridinium tripos, Ceratophorus tripos, Neoceratium tripos)	YP		YP	Daugbjerg et al. (2000)<br>Hansen and Daugbjerg (2004)<br>Murray et al. (2005)<br>Kim and Kim (2007)<br>Yamaguchi et al. (2011)<br>Kim and Park (2017)<br>Efimova et al. (2019)<br>Accattatis et al. (2020)
	paradoxoides (= Ceratium paradoxides, Neoceratium paradoxides)	YP			Gómez et al. (2010c)<br>Accattatis et al. (2020)
	pentagonus (= Ceratium pentagonum, Neoceratium pentagonum)	YP			Gómez et al. (2010c)
	petersii (= Ceratium petersii)	Y			Gómez et al. (2010c)
	playcornis (= Ceratium platycorne, Neoceratium platicornea0	YP			Gómez et al. (2010c)
	symmetricus (= Ceratium symmetricum, Ceratium gracile var. symmetricum, Neoceratium symmetricum)	YP			Gómez et al. (2010c)

tenuis (= Ceratium tenue, Neoceratium tenue)	YP			Grzebyk et al. (1998)<br>Dolapsakis et al. (2006)<br>Hoppenrath and Leander (2007)<br>Gómez et al. (2010c)<br>Hoppenrath et al. (2012b)<br>Yoon et al. (2012)<br>Kim and Park (2014)
Triposolenia				
bicornis	Y			Gómez et al. (2011a)<br>Gómez et al. (2012b)
Tyrannodinium				
berolinense<br>single cell FJ167681			YP	Calado et al. (2009)<br>Hansen and Daugbjerg (2011)<br>Zhang, Q. et al. (2011b)<br>Jeong et al. (2014c)<br>Pandeirada et al. (2014)<br>Luo et al. (2016b)<br>Boutrup et al. (2017)<br>Gottschling et al. (2021b)
edax<br>HBI:HB2010a	YP	YP	YP	Kang et al. (2015)<br>Boutrup et al. (2016)<br>Žerdoner Čalasan et al. (2019)<br>Gottschling et al. (2021b)
Unruhdinium				
jiulongense (= Peridiniopsis jiulongensis)<br>PSJL01 KM217384	YP	YP		You et al. (2015)<br>Yamada et al. (2017)<br>Kretschmann et al. (2018b)<br>Satta et al. (2020)<br>Gottschling et al. (2021b)
kevei (= Peridiniopsis kevei)	Y	Y	YP	Gottschling et al. (2012)<br>Saburova et al. (2012)<br>Yamada et al. (2015)<br>You et al. (2015)<br>Yamada et al. (2017)<br>Kretschmann et al. (2018b)<br>Satta et al. (2020)
cf. kevei<br>DA08 LC054935<br>HG327 LC054936<br>Tomi-iwa Canal Park AB353770	Y	Y	Y	Yamada et al. (2015)<br>Yamada et al. (2017)<br>Žerdoner Čalasan et al. (2019)<br>Satta et al. (2020)
minimum (= Peridiniopsis minima)<br>QZ-2012 HBI:FJ201101a JQ639767	YP	YP	YP	Yamada et al. (2017)<br>Kretschmann et al. (2018b)<br>Žerdoner Čalasan et al. (2019)<br>Satta et al. (2020)
niei (= Peridiniopsis niei)<br>Donghu HM596542	YP/YA	YP	YP	Gottschling et al. (2012)<br>Craveiro et al. (2013)

				Gu et al. (2013a)<br>You et al. (2015)<br>Yamada et al. (2017)<br>Gu et al. (2018)<br>Luo et al. (2019)<br>Satta et al. (2020)
penardii / var. robustum<br>(Glenodinium penardii,<br>Peridinium penardii,<br>Peridiniopsis penardii)<br>AB353773<br>HM596547<br>HM596548<br>Jiulonjiang HM596543<br>JLJ HM596544<br>Manwan HM596546<br>Shiga, Lake Biwa AB353771<br>SZ HM596545	Y	Y	Y	Zhang, Q. et al. (2011b)<br>Gottschling et al. (2012)<br>Saburova et al. (2012)<br>Craveiro et al. (2013)<br>Gottschling and McLean (2013)<br>Gu et al. (2013a)<br>Jeong et al. (2014c)<br>You et al. (2015)<br>Yamada et al. (2017)<br>Gu et al. (2018)<br>Kretschmann et al. (2018b)<br>Luo et al. (2019)<br>Žerdoner Čalasan et al. (2019)<br>Hoppenrath et al. (2020)<br>Luo, Z. et al. (2020)<br>Satta et al. (2020)<br>Gottschling et al. (2021a,b)
niei<br>Donghu HM596542<br>Xiangxi HM596552	Y	Y	Y	Gottschling et al. (2012)<br>Gottschling and McLean (2013)<br>Yamada et al. (2017)<br>Žerdoner Čalasan et al. (2019)<br>Luo, Z. et al. (2020)
sp.<br>QZ-2012 HBI:FJ201010	YA	YA	YA	Gu et al. (2013a)
Votadinium				
concavum			Y	Gurdebek et al. (2020)
Vulcanodinium				
rugosum<br>IFR10-027	Y	Y	Y	Nézan and Chomérat (2011)<br>Sarai et al. (2013)<br>Anglès et al. (2017)<br>Luo et al. (2018a)<br>Luo, Z. et al. (2019)<br>Žerdoner Čalasan et al. (2019)<br>Gottschling et al. (2021a,b)
Wangodinium				
sinense			Y	Luo et al. (2018b)<br>Gómez et al. (2019a)
Warnowia				
sp.<br>BC	YP	YP	YP	Žerdoner Čalasan et al. (2019)<br>Gottschling et al. (2021a,b)
sp.	YP	YP	YP	Reñé et al. (2015)

Florida FJ947046				Žerdoner Čalasan et al. (2019)
sp. 1<br>AR83 KP790234<br>AR86 KP790186<br>AR87 KP790187<br>AR199 KP790188			Y	Reñé et al. (2015)
sp. 2<br>AR93 KP790236<br>AR223 KP790237<br>AR224 KP790238			Y	Reñé et al. (2015)<br>Annenkova (2018)
sp. 3<br>AR215 KP790239			YP	Reñé et al. (2015)
sp. 4<br>AR253 KP790240<br>AR254 KP790241<br>AR255 KP790168<br>(equivalent to D1-D3 of Protoerythropsis<br>UBC1 FJ947037<br>UBC2 FJ947036	Y		Y	Reñé et al. (2015)<br>Romeikat et al. (2019)
sp. 5<br>FJ947042<br>BSL-2009a FJ947040<br>AR297 KP790242<br>AR320 KP790170	Y		Y	Hoppenrath et al. (2009)<br>Hoppenrath et al. (2012a)<br>Nézan et al. (2014)<br>Reñé et al. (2015)<br>Na et al. (2017)<br>Luo et al. (2018b)<br>Moestrup et al. (2018)<br>Gómez et al. (2019a)
sp.<br>IFR1101 KJ508397	YP		YP	Nézan et al. (2014)<br>Annenkova (2018)<br>Romeikat et al. (2019)<br>Žerdoner Čalasan et al. (2019)
SCM38C1 AY664983	YP	YP	YP	Žerdoner Čalasan et al. (2019)
SCM16C30 AY665026	YP	YP	YP	Žerdoner Čalasan et al. (2019)
Yihiella				
yeosuensis	Y		YP	Hehenberger et al. (2017)<br>Jang et al. (2017a,b)<br>LaJeunesse et al. (2018)<br>Gottschling et al. (2021b)

References

Accattatis, V., Piccini, C., Huber, P., Metz, S., Rueda, E., Devercelli, M. (2020). Identifying Invaders: The case of *Ceratium furcoides* (Gonyaulacales, Dinophyceae) in South America. J. Phycol. 56(5):1362-1366 DOI: 10.1111/jpy.13015 PMID: 32399960

Amorim, A., Veloso, V., Rodríguez, F. & Fraga, S. (2013). Life cycle, morphology and phylogeny of species of *Fragilidium* Balech from west Iberia. In: Biological and geological perspectives of dinoflagellates. [Eds.] F. Marret, J.M. Lewis & L.R. Bradley, pp. 223–232. The Micropalaeontological Society, Special Publication, Geological Society, London, UK. https://doi.org/10.1080/00318884.2019.1620582

Adachi, M., Y. Sako & Ishida, Y. (1996). Analysis of *Alexandrium* (Dinophyceae) species using sequences of the 5.8S ribosomal DNA and internal transcribed spacer regions. J. Phycol. 32:424-432. DOI: 10.1111/j.0022-3646.1996.00424.x

Adachi, M., Sako, Y. & Ishida, Y. (1997). Analysis of *Gymnodinium catenatum* Dinophyceae using sequences of the 5.8S rDNA-ITS regions and random amplified polymorphic DNA. Fish Sci. 63:701-707. https://doi.org/10.2331/fishsci.63.701

Alves-de-Souza, C., Cornet, C., Nowaczyk, A., Gasparini, S., Skovgaard, A. & Guillou, L. (2011). *Blastodinium* spp. infect copepods in the ultra-oligotrophic marine waters of the Mediterranean Sea. Biogeosciences 8:2125-2136. https://doi.org/10.5194/bg-8-2125-2011

Anderson, D.M., Alpermann, T.J., Cembella, A.D., Collos, Y., Masseret, E. & Montresore, M. (2012). The globally distributed genus *Alexandrium*: Multifaceted roles in marine ecosystems and impacts on human health. Harmful Algae 14:10-35. DOI: 10.1016/j.hal.2011.10.012

Anglès, S., Reñé, A., Garcés, E., Lugliè, A., Sechi, N., Camp, J. & Satta, C.T. (2017). Morphological and molecular characterization of *Bysmatrum subsalsum* (Dinophyceae) from the western Mediterranean Sea reveals the existence of cryptic species. J. Phycol. 53:833–847. DOI: 10.1111/jpy.12546

Annenkova, N.V., Lavrov, D.V. & Belikov, S.I. (2011). Dinoflagellates associated with freshwater sponges from the ancient Lake Baikal. Protist 1622:222–236. DOi: 10.1016/j.protis.2010.07.002

Annenkova, N.V. (2018). Identification of Lake Baikal plankton dinoflagellates from the genera *Gyrodinium* and *Gymnodinium* using single-cell PCR. Russ. J. Genet. 54(11):1302–1313. https://doi.org/10.1134/S1022795418110030

Annenkova, N.V., Hansen, G., Moestrup, Ø. & Rengefors, K. (2015). Recent radiation in a marine and freshwater dinoflagellate species flock. ISME J. 9: 1821–1834. https://doi.org/10.1038/ismej.2014.267

Akselman, R., Krock, B., Alpermann, T.J., Tillmann, U., Borel, C.M., Almandoz, G.O. & Ferrario, M.E. (2015). *Protoceratium reticulatum* (Dinophyceae) in the austral southwestern Atlantic and the first report on YTX-production in shelf waters of Argentina. Harmful Algae 45:40-52. https://doi.org/10.1016/j.hal.2015.03.001

Attaran-Fariman, G. & Bolch, C.J.S. (2007). *Scrippsiella irregularis* sp. nov. (Dinophyceae), a new dinoflagellate from the southeast coast of Iran. Phycologia 46: 572-582. DOI: 10.2216/07-02.1

Attaran-Fariman, G. & Bolch, C.J.S. (2012). Morphology and phylogeny of *Scrippsiella trochoidea* (Dinophyceae) a potentially harmful bloom forming species isolated from the sediments of Iran's south coast. Iran. J. Fish. Sci. 11(2):252-270. DOI: magiran.com/p1034016

Bachvaroff, T.R., Kim, S., Guillou, L., Delwiche, C.F. & Coats, D.W. (2012). Molecular diversity of the Syndinean genus *Euduboscquella* based on single-cell PCR analysis. Appl. Environ. Microbiol. 78(2):334-345. DOI: 10.1128/AEM.06678-11

Balzano S., Gourvil P., Siano R., Chanoine, M., Marie, D., Lessard, S., Sarno, D. & Vaulot, D. (2012). Diversity of cultured photosynthetic flagellates in the northeast Pacific and Arctic Oceans in summer. Biogeosciences 9:4553-4571. DOI:10.5194/bg-9-4553-2012

Baytut, Ö., Gürkanli, C.T., Deniz, E., Özkoç, İ. & Gönülol, A. (2016). First molecular records of potentially harmful planktonic dinoflagellates from the southern Black Sea. Turk. J. Bot. 40: 546-556. DOI: 10.3906/bot-1506-34

Benico, G., Takahashi, K., Lum, W.M. & Iwataki, M. (2019). Morphological variation, ultrastructure, pigment composition and phylogeny of the star-shaped dinoflagellate *Asterodinium gracile* (Kareniaceae, Dinophyceae). Phycologia 58(4):405-418. https://doi.org/10.1080/00318884.2019.1601948

Benico, G., Takahashi, K., Lum, W.M., Yñiguez, A.T. & Iwataki, M. (2020). The harmful unarmored dinoflagellate *Karlodinium* in Japan and Philippines, with reference to ultrastructure and micropredation of *Karlodinium azanzae* sp. nov. (Kareniaceae, Dinophyceae). J. Phycol. 56:1264–1282. DOI: 10.1111/jpy.13030-19-248

Bergholtz, T., Daugbjerg, N., Moestrup, Ø. & Fernández-Tejedor, M. (2005). On the identity of *Karlodinium veneficum* and description of *Karlodinium armiger* sp. nov. (Dinophyceae), based on light and electron microscopy, nuclear-encoded LSU rDNA, and pigment composition. J. Phycol. 42:170– 93. https://doi.org/10.1111/j.1529-8817.2006.00172.x

Bolch, C.J.S. & Campbell, C.N. (2004). Morphology and phylogenetic affinities of *Thecadinium foveolatum* sp. nov. (Dinophyceae: Thecadiniaceae), a new marine benthic dinoflagellate from the West of Scotland. Europ. J. Phycol. 39: 351-360. https://doi.org/10.1080/09670260410001728098

Boo, W.H., Wah, L.Y., Keryea, S. & Davis, R.J. (2020). The diversity of Symbiodiniaceae hosted by *Palythoa tuberculosa* found at the edge of the South China Sea. J. Sustain. Sci. and Manag. 15(4):54-65. http://doi.org/10.46754/jssm.2020.06.006

Borsato, G.T., Salgueiro, F., Silva, C.G., Menezes-Salgueiro, A.D., & Nascimento, S.M. (2020). *Ostreopsis lenticularis* Y. Fukuyo (Dinophyceae, Gonyaulacales) from the South Atlantic Ocean: morphological and molecular characterization. Mar. Pollut. Bull. 158:111441. https://doi.org/10.1016/j.marpolbul.2020.111441

Botes, L., Sym, S. D. & Pitcher, G. C. (2003). *Karenia cristata* sp. nov. and *Karenia bicuneiformis* sp. nov. (Gymnodiniales, Dinophyceae): two new *Karenia species* from the South African coast. Phycologia 42:563–571. DOI: 10.2216/i0031-8884-42-6-563.1

Boutrup, P.V., Moestrup, Ø., Tillmann, U. & Daugbjerg, N. (2016). *Katodinium glaucum* (Dinophyceae) revisited: proposal of new genus, family and order based on ultrastructure and phylogeny. Phycologia 55(2):147-164. https://doi.org/10.2216/15-138.1

Boutrup, P.V., Moestrup, Ø., Tillmann, U. & Daugbjerg, N. (2017). Ultrastructure and phylogeny of *Kirithra asteri* gen. et sp. nov. (Ceratoperidiniaceae, Dinophyceae) — a free-living, thin-walled marine photosynthetic dinoflagellate from Argentina. Protist 168(5):586-611. DOI: 10.1016/j.protis.2017.08.001

Cai, R., Kayal, E., Alves-de-Souza, C., Bigeard, E., Corre, E., Jeanthon, C., Marie, D., Porcel, B.M., Siano, R., Szymczak, J., Wolf, M. & Guillou, L. (2020). Cryptic species in the parasitic *Amoebophrya* species complex revealed by a polyphasic approach. Sci. Rep. 10:2531. https://doi.org/10.1038/s41598-020-59524-z

Calado, A.J., Craveiro, S.C., Daugbjerg, N. & Moestrup, Ø. (2006). Ultrastructure and LSU rDNA-based phylogeny of *Esoptrodinium gemma* (dinophyceae), with notes on feeding behavior and the description of the flagellar base area of a planozygote. J. Phycol. 42:434–452. DOI: 10.1111/j.1529-8817.2006.00195.x

Calado, A.J., Craveiro, S.C., Daugbjerg, N. & Moestrup, Ø. (2009). Description of *Tyrannodinium* gen. nov., a freshwater dinoflagellate closely related to the marine *Pfiesteria*-like species. J. Phycol. 45:1195–1205. DOI: 10.1111/j.1529-8817.2009.00735.x

Carnicer, O., Tunin-Ley, A., Andree, K.B., Turquet, J., Diogène, J. & Fernández-Tejedor, M. (2015). Contribution to the genus *Ostreopsis* in Reunion Island (Indian Ocean): Molecular, morphologic and toxicity characterization. Cryptogam. Algol. 36:101–119. DOI: 10.7872/crya.v36.iss1.2015.101.

Carnicer O., Okolodkov Y.B., Garcia-Altares, M., Keith, I., Andree, K.B., Diogène, J. & Fernández-Tejedor, M. (2020). *Ostreopsis* cf. *ovata* and *Ostreopsis lenticularis* (Dinophyceae: Gonyaulacales) in the Galapagos Marine Reserve. Sci. Mar. 84(3):199-213. https://doi.org/10.3989/scimar.05035.08A

Cen, J., Wang, J., Huang, L., Lin, Y., Ding, G., Qi, Y. & Lü, S. (2021). *Karlodinium elegans* sp. nov. (Gymnodiniales, Dinophyceae), a novel species isolated from the East China Sea in a dinoflagellate bloom. J. Ocean. Limnol. 39:242–258. https://doi.org/10.1007/s00343-020-0221-4

Chinain, M., Faust, M.A. & Pauillac, S. (1999). Morphology and molecular analyses of three species of *Gambierdiscus* (Dinophyceae): *G. pacificus*, sp. nov., *G. australes*, sp. nov., and *G. polynesiensis*, sp. nov. J. Phycol. 35:1282–1296. https://doi.org/10.1046/j.1529-8817.1999.3561282.x

Chomérat, N. & Bilien, G. (2014). *Madanidinium loirii* gen. et sp. nov. (Dinophyceae), a new marine benthic dinoflagellate from Martinique Island, Eastern Caribbean. Europ. J. Phycol. 49(2):165-178. https://doi.org/10.1080/09670262.2014.898797

Chomérat, N., Sellos, D.Y., Zentz, F. & Nézan, E. (2010b). Morphology and molecular phylogeny of *Prorocentrum consutum* sp. nov. (Dinophyceae), a new benthic dinoflagellate from South Brittany (northwestern France). J. Phycol. 46:183–194. https://doi.org/10.1111/j.1529-8817.2009.00774.x

Chomérat, N., Zentz, F., Boulben, S., Bilien, G., van Wormhoudt, A. & Nézan, E. (2011). *Prorocentrum glenanicum* sp. nov. and *P. pseudopanamense* sp. nov. (Prorocentrales, Dinophyceae), two new benthic dinoflagellate species from South Brittany (northwestern France). Phycologia 50:202–214. https://doi.org/10.2216/10-12.1

Chomérat, N., Saburova, M., Bilien, G. & Al-Yamani, F. (2012). *Prorocentrum bimaculatum* sp. nov. (Dinophyceae, Prorocentrales), a new benthic dinoflagellate species from Kuwait (Arabian Gulf). J. Phycol. 48:211–221. https://doi.org/10.1111/j.1529-8817.2011.01102.x

Chomérat, N. & Bilien, G. (2014). *Madanidinium loirii* gen. et sp. nov. (Dinophyceae), a new marine benthic dinoflagellate from Martinique Island, eastern Caribbean. Europ. J. Phycol. 49:165–178. https://doi.org/10.1080/09670262.2014.898797

Chomérat, N., Bilien, G. & Zentz, F.A. (2019a). A taxonomical study of benthic *Prorocentrum* species (Prorocentrales, Dinophyceae) from Anse Dufour (Martinique Island, eastern Caribbean Sea). Mar. Biodiv. 49:1299–1319. https://doi.org/10.1007/s12526-018-0913-6

Chomérat, N., Bilien, G., Derrien, A., Henry, K., Ung, A., Viallon, J., Darius, H., Gatti, C.M., Roué, M., Herve, F., Réveillon, D., Amzil, Z. & Chinain, M. (2019b). *Ostreopsis lenticularis* Y. Fukuyo (Dinophyceae, Gonyaulacales) from French Polynesia (South Pacific Ocean): A revisit of its morphology, molecular phylogeny and toxicity. Harmful algae 84:95-111. https://doi.org/10.1016/j.hal.2019.02.004

Chomérat, N., Bilien, G., Couté, A. & Quod, J. (2020a). Reinvestigation of *Ostreopsis mascarenensis* Quod (Dinophyceae, Gonyaulacales) from Réunion Island (SW Indian Ocean): molecular phylogeny and emended description. Phycologia 59:140-153. https://doi.org/10.1080/00318884.2019.1710443

Chomérat, N., Bilien, G., Viallon, J., Hervé, F., Réveillon, D., Henry, K., Zubia, M., Vieira, C., Ung, A., Gatti, C., Roué, M., Derrien, A., Amzil, Z., Darius, H.T. & Chinain, M. (2020b). Taxonomy and toxicity of a bloom-forming *Ostreopsis* species (Dinophyceae, Gonyaulacales) in Tahiti island (South Pacific Ocean): one step further towards resolving the identity of *O. siamensis*. Harmful Algae 98:101888. https://doi.org/10.1016/j.hal.2020.101888

Coats, D.W., Sunju, K., Bachvaroff, T.R., Handy, S.M. & Delwiche, C.F. (2010). *Tintinnophagus acutus* n. g., n. sp. (Phylum Dinoflagellata), an ectoparasite of the ciliate *Tintinnopsis cylindrica* Daday 1887, and its relationship to *Duboscquodinium collini* Grassé 1952. J. Eukaryot. Microbiol. 57(6):468-82. DOI: 10.1111/j.1550-7408.2010.00504.x.

Cohen-Fernández, E.J., Pedroche, F.F., Palacios, M.R., Hernández, S.Á. & Castillo, E.M. (2010). Molecular phylogeny of *Prorocentrum* (Dinoflagellata) from the Pacific coast of Mexico based on the parsimony analysis of fragment of LSU rDNA and SSU rDNA. Int. J. Plant. Physiol. Biochem. 2:29–37. https://doi.org/10.5897/IJPPB.9000031

Cooney, E.C., Okamoto, N., Cho, A., Hehenberger, E., Richards, T.A., Santoro, A.E., Worden, A.Z., Leander, B.S. & Keeling P.J. (2020). Single-cell transcriptomics of *Abedinium* reveals a new early-branching dinoflagellate lineage. Genome Biol. Evol. 12(12):2417–2428. DOI: 10.1093/gbe/evaa196

Craveiro, S. C., Calado, A.J., Daugbjerg, N., Moestrup, Ø. (2009). Ultrastructure and LSU rDNA–based revision of *Peridinium* group *Palatinum* (Dinophyceae) with the description of *Palatinus* gen. nov. J. Phycol. 45(56):1175-1194. https://doi.org/10.1111/j.1529-8817.2009.00739.x

Craveiro, S.C., Moestrup, Ø., Daugbjerg, N. & Calado, A.J. (2010). Ultrastructure and large subunit rDNA-based phylogeny of *Sphaerodinium cracoviense*, an unusual freshwater dinoflagel-late with a

novel type of eyespot. J. Eukaryot. Microbiol. 57:568–585. https://doi.org/10.1111/j.1550-7408.2010.00512.x

Craveiro, S. C., Calado, A.J., Daugbjerg, N., Hansen, G. & Moestrup, Ø. (2011). Ultrastructure and LSU rDNA-based phylogeny of *Peridinium lomnickii* and description of *Chimonodinium* gen. nov (Dinophyceae). Protist 162:590–615. DOI: 10.1016/j.protis.2011.03.003

Craveiro, S.C., Pandeirada, M.S., Daugbjerg, N., Moestrup, Ø. & Calado, A.J. (2013). Ultrastructure and phylogeny of *Theleodinium calcisporum* gen. et sp. nov., a freshwater dinoflagellate that produces calcareous cysts. Phycologia 52:488–507. DOI: 10:2216/13–152.

Craveiro, S.C., Daugbjerg, N., Moestrup, Ø. & Calado, A.J. (2015). Fine-structural characterization and phylogeny of *Peridinium polonicum*, type species of the recently described genus *Naiadinium* (Dinophyceae). Eur. J. Protistol. 51(4):259-279. http://dx.doi.org/10.1016/j.ejop.2015.05.001

Craveiro, S.C., Daugbjerg, N., Moestrup, Ø., & Calado, A.J. (2016). Studies on *Peridinium aciculiferum* and *Peridinium malmogiense* (=*Scrippsiella hangoei*): comparison with *Chimonodinium lomnickii* and description of *Apocalathiu*m gen. nov. (Dinophyceae). Phycologia 56(1):21-35. DOI: 10.2216/16-20.1

Cyronak, T. & Tomas, C. (2008). Morphological and phylogenetic description of an unusual *Amphidinium* (Dinophyceae) species. Moestrup, O., Doucette, G., Enevoldsen, H., Godhe, A., Hallegraeff, G., Luckas, B., Lundholm, N., Lewis, J., Rengefors, K., Sellner, K., Steidinger, K., Tester, P. & Zingone, A. (Eds). Proceedings of the 12th International Conference on Harmful Algae. International Society for the Study of Harmful Algae and Intergovernmental Oceanographic Commission of UNESCO, 2008 Copenhagen, Denmark, 4-8 September 2006. pp. 249-252.

Daugbjerg, N., Hansen, G., Larsen, J. & Moestrup, Ø. 2000. Phylogeny of some of the major genera of dinoflagellates based on ultrastructure and partial LSU rDNA sequence data, including the erection of three new genera of unarmoured dinoflagellates. Phycologia 39:302-317. DOI: 10.2216/i0031-8884-39-4-302.1

Daugbjerg, N., Hansen, S.A. & Richardson, K. (2019). Cryptic diversity of small-sized species of *Phalacroma* (Dinophysales, Dinophyceae) from Denmark Strait (Eastern Arctic Greenland). Phycol. Res. 67:244–249. DOI: 10.1111/pre.12366

David, H., Laza-Martínez, A., Miguel, I. & Orive, E. (2013). *Ostreopsis* cf. *siamensis* and *Ostreopsis* cf. *ovata* from the Atlantic Iberian Peninsula: Morphological and phylogenetic characterization. Harmful Algae 30:44-55. DOI: 10.1016/j.hal.2013.08.006

David, H., Laza-Martínez, A., Garcia-Etxebarria, K., Riobó, P. & Orive, E. (2014a). Characterization of *Prorocentrum elegans* and *Prorocentrum levis* (Dinophyceae) from the southeastern Bay of Biscay by morphology and molecular phylogeny. J. Phycol. 50(4):718-26. DOI: 10.1111/jpy.12200.

David, H., Laza-Martínez, A., Miguel, I. & Orive, E. (2014b). Broad distribution of *Coolia monotis* and restricted distribution of *Coolia* cf. *canariensis* (Dinophyceae) in the Atlantic coast of the Iberian Peninsula. Phycologia 53(4):342-352. DOI: 10.2216/13-214.1

David, H., Laza-Martínez, A., Rodríguez, F., Fraga, S. & Orive, E. (2020). *Coolia guanchica* sp. nov. (Dinophyceae) a new epibenthic dinoflagellate from the Canary Islands (NE Atlantic Ocean). Europ. J. Phycol. 55(1):76-88. DOI: 10.1080/09670262.2019.1651400

Dawut, M., Sym, S.D., Suda, S. & Horiguchi, T. (2018). *Bysmatrum austrafrum* sp. nov. (Dinophyceae), a novel tidal pool dinoflagellate from South Africa. Phycologia 57(2):169-178. DOI: 10.2216/17-54.1

de Azevedo Tibiriçá, C.E.J., Sibat, M., Fernandes, L.F., Bilien, G., Chomérat, N., Hess, P. & Mafra, L.L. Jr. (2020). Diversity and toxicity of the genus *Coolia* Meunier in Brazil, and detection of 44-methyl gambierone in *Coolia tropicalis*. Toxins 12:327. doi:10.3390/toxins12050327

de Queiroz Mendes, M.C., de Castro Nunes, J.M., Fraga, S., Rodríguez, F., Franco, J.M., Riobó, P., Branco, S. & Menezes, M. (2019). Morphology, molecular phylogeny and toxinology of *Coolia* and *Prorocentrum* strains isolated from the tropical South Western Atlantic Ocean. Bot. Mar. 62:125–140. DOI: 10.1515/bot-2018-005

de Salas, M.F., Bolch, C.J.S., Botes, L., Nash G., Wright, S.W. & Hallegraeff, G.M. (2003). *Takayama* gen. nov. (Gymnodiniales, Dinophyceae), a new genus of unarmored dinoflagellates with sigmoid apical grooves, including the description of two new species. J. Phycol. 39:1233-1246 https://doi.org/10.1111/j.0022-3646.2003.03-019.x

de Salas, M.F., Bolch, C.J.S. &Hallegraeff, G.M. (2005a). *Karlodinium australe* sp. nov. (Gymnodiniales, Dinophyceae), a new potentially ichthyotoxic unarmoured dinoflagellate from lagoonal habitats of south-eastern Australia. Phycologia 44:640–650. DOI: 10.2216/0031-8884(2005)44[640:KASNGD]2.0.CO;2

de Salas, M.F., Rhodes, L.L., Mackenzie, L.A. & Adamson, J.E. (2005b). Gymnodinoid genera *Karenia* and *Takayama* (Dinophyceae) in New Zealand coastal waters. New Zeal. J. Mar. Fresh. Res. 39:135-139. https://doi.org/10.1080/00288330.2005.9517296

de Salas, M. F., Laza-Martinez, A. & Hallegraeff, G. M. (2008). Novel unarmored dinoflagellates from the toxigenic family Kareniaceae (Gymnodiniales): Five new species of *Karlodinium* and one new *Takayama* from the Australian sector of the Southern Ocean. J. Phycol. 44:241–257. DOI: 10.1111/j.1529-8817.2007.00458.x

Delmail D., Labrousse P., Crassous P., Hourdin, P., Guri, M., & Botineau, M. (2011). *Prorocentrum rivalis* sp. nov. (Dinophyceae) and its phylogenetic affinities inferred from analysis of a mixed morphological and LSU rRNA data set. Biologia 66:418–424. DOI: 10.1111/j.1529-8817.2007.00458.x

D'Onofrio, G., Marino, D., Bianco, L., Busico, E. & Montresor, M. (1999). Toward an assessment on the taxonomy of dinoflagellates that produce calcareous cysts (Calciodinelloideae, Dinophyceae): A morphological and molecular approach. J. Phycol. 35:1063–1078. https://doi.org/10.1046/j.1529-8817.1999.3551063.x

Dolapsakis, N.P., Kilpatrick, M.W., Economou-Amilli, A. & Tafas, T. (2006). Morphology and rDNA phylogeny of a Mediterranean *Coolia monotis* (Dinophyceae) strain from Greece. Sci. Mar. 70:67–76. https://scientiamarina.revistas.csic.es/index.php/scientiamarina/article/view/184

Dolapsakis, N.P. & Economou-Amilli, A. (2009). A new marine species of *Amphidinium* (Dinophyceae) from Thermaikos Gulf, Greece. Acta Protozool. 48:153–170.

Edvardsen, B., Shalchian-Tabrizi, K., Jakobsen, K.S., Medlin, L.K., Dahl, E., Brubak, S. & Paasche, E. (2003). Genetic variability and molecular phylogeny of *Dinophysis* species (Dinophyceae) from Norwegian waters inferred from single cell analyses of rDNA. J. Phycol. 39(2):395–408. https://doi.org/10.1046/j.1529-8817.2003.01252.x

Efimova, K.V., Orlova, T.Y. & Brykov, V.A. (2014). Phylogenetic characterization of cryptic species of the marine dinoflagellate, *Ostreopsis* sp. Shmidt, 1902, from Russian coastal waters, the Sea of Japan. J. Bio. & Env. Sci. 5(4):317–332. Corpus ID: 51769280

Efimova, K.V., Selina, M.S. & Hoppenrath, M. (2019). New morphological data and molecular phylogeny of the benthic dinoflagellate *Pseudothecadinium campbellii* (Dinophyceae, Gonyaulacales). Eur. J. Protistol. DOI: https://doi.org/10.1016/j.ejop.2019.125638

Ellegaard, M., Daugbjerg, N., Rochon, A., Lewis, J. & Harding, I. (2003). Morphological and LSU rDNA sequence variation within the *Gonyaulax spinifera-Spiniferites* group (Dinophyceae) and proposal of *G. elongata* comb. nov. and *G. membranacea* comb. nov. Phycologia 42(2):151-164. DOI: 10.2216/i0031-8884-42-2-151.1

Ellegaard, M., Head, M.J. & Versteegh, G.J.M. (2018). Linking biological and geological data on dinoflagellates using the genus *Spiniferites* as an example: the implications of species concepts, taxonomy and dual nomenclature. Palynology 42(S1):221–230. https://doi.org/10.1080/01916122.2018.1465732

Fawcett, R.C. & Parrow, M.W. 920120. Cytological and phylogenetic diversity in fresh water *Esoptrodinium / Bernardinium* species (Dinophyceae). J. Phycol. 48:793–807. DOI: 10.1111/j.1529-8817.2012.01174.x

Fodor, L. (2014). Dinoflagellate diversity and dynamics in Outer Oslofjorden as revealed by molecular methods. Master Thesis in Marine Biology, Department of Biosciences, University of Oslo. 69 p.

Fraga, S. & Rodríguez, F. (2014). Genus *Gambierdiscus* in the Canary Islands (NE Atlantic Ocean) with description of *Gambierdiscus silvae* sp. nov., a new potentially toxic epiphytic benthic dinoflagellate. Protist 165(6):839–853. https://doi.org/10.1016/j.protis.2014.09.003

Fraga, S., Penna, A., Bianconi, I., Paz, B. & Zapata, M. (2008). *Coolia canariensis* sp. nov. (Dinophyceae), a new nontoxic epiphytic benthic dinoflagellate from the Canary Islands J. Phycol. 44(4):1060-1070. https://doi.org/10.1111/j.1529-8817.2008.00555.x

Fraga, S., Rodríguez, F., Caillaud, A., Diogène, J, Raho, N. & Zapata, M. (2011). *Gambierdiscus excentricus* sp. nov. (Dinophyceae), a benthic toxic dinoflagellate from the Canary Islands (NE Atlantic Ocean). Harmful Algae. 11:10–22. https://doi.org/10.1016/j.hal.2011.06.013

Fraga, S., Rodríguez, F., Riobó, P. & Bravo, I. (2016). *Gambierdiscus balechii* sp. nov (Dinophyceae), a new benthic toxic dinoflagellate from the Celebes Sea (SW Pacific Ocean). Harmful Algae 58:93-105. http://dx.doi.org/10.1016/j.hal.2016.06.004

Garcés, E., Fernandez, M., Penna, A., Van Lenning, K., Gutierrez, A., Camp J. & Zapata, M. (2006). Characterization of NW Mediterranean *Karlodinium* spp. (Dinophyceae) strains using morphological, molecular, chemical, and physiological methodologies. J. Phycol. 42:1096–1112. https://doi.org/10.1111/j.1529-8817.2006.00270.x

Gast, R.J. (2006). Molecular phylogeny of a potentially parasitic dinoflagellate isolated from the solitary radiolarian, *Thalassicolla nucleata*. J. Eukaryot. Microbiol. 53:43–45. DOI: 10.1111/j.1550-7408.2005.00071.x

Gast, R.J. & Caron, D.A. (1996). Molecular phylogeny of symbiotic dinoflagellates from plankton foraminifera and radiolarian. Mol. Biol. Evol. 13:1192-1197.

Gast, R.J. & Caron, D.A. (2001). Photosymbiotic associations in planktonic foraminifera and radiolaria. Hydrobiologia 461:1–7. https://doi.org/10.1023/A:1012710909023

Gómez, F. (2018). Redefinition of *Ceratoperidinium* and *Pseliodinium* (Ceratoperi - Diniaceae, Dinophyceae) including reassignment of *Gymnodinium fusus*, *Cochlodinium helix* and *C. pirum* to *Pseliodinium*. CICIMAR Oceánides 33(1):1-11. DOI: 10.37543/oceanides.v33i1.218

Gómez, F. & Skovgaard, A. (2014). Molecular phylogeny of the parasitic dinoflagellate *Chytriodinium* within the *Gymnodinium* clade (Gymnodiniales, Dinophyceae). J. Eukaryot. Microbiol. 62:422–425. DOI: 10.1111/jeu.12180

Gómez, F. & Skovgaard, A. (2015). The molecular phylogeny of the type-species of *Oodinium* Chatton 1912 (dinoflagellata: oodiniaceae) a highly divergent parasitic dinoflagellate with nondinokaryotic characters. Syst. Parasitol. 90:125–135. https://doi.org/10.1007/s11230-014-9538-8

Gómez, F. & Gast, R.J. (2018). Dinoflagellates *Amyloodinium* and *Ichthyodinium* (Dinophyceae), parasites of marine fishes in the South Atlantic Ocean. Dis. Aquat. Org. 131: 29–37. https://doi.org/10.3354/dao03274

Gómez, F. & Artigas, L.F. (2019). Redefinition of the dinoflagellate genus *Alexandrium* based on *Centrodinium*: Reinstatement of *Gessnerium* and *Protogonyaulax*, and *Episemicolon* gen. nov. (Gonyaulacales, Dinophyceae). J. Mar. Sci. (Hindawi) 1284104:1-17. https://doi.org/10.1155/2019/1284104

Gómez, F., López-García, P. & Moreira, D. (2009a). Molecular phylogeny of the ocelloid-bearing dinoflagellates *Erythropsidinium* and *Warnowia* (Warnowiaceae, Dinophyceae). J. Eukaryot. Microbiol. 56(5):440–445. DOI: 10.1111/j.1550-7408.2009.00420.x

Gómez, F., Moreira, D. & López-García, P. (2009b). Life cycle and molecular phylogeny of the dinoflagellates *Chytriodinium* and *Dissodinium*, ectoparasites of copepod eggs. Eur. J. Protistol. 45(4):260-70. DOI: 10.1016/j.ejop.2009.05.004

Gómez, F., López-García, P., Nowaczyk, A. & Moreira, D. (2009c). The crustacean parasites *Ellobiopsis* Caullery, 1910 and *Thalassomyces* Niezabitowski, 1913 form a monophyletic divergent clade within the Alveolata. Syst Parasitol 74:65–74. https://doi-org.libproxy.lib.unc.edu/10.1007/s11230-009-9199-1

Gómez, F., Moreira, D. & López-García, P. (2010a). Molecular phylogeny of the dinoflagellates *Podolampas* and *Blepharocysta* (Peridiniales, Dinophyceae). Phycologia 49(3):212-220. DOI: 10.2216/09-29.1

Gómez, F., Moreira, D. & López-García, P. (2010b). Molecular phylogeny of Noctilucoid dinoflagellates (Noctilucales, Dinophyceae). Protist 161(3):466-78. DOI: 10.1016/j.protis.2009.12.005.

Gómez, F., Moreira, D. & López-García, P. (2010c). *Neoceratium* gen. nov., a new genus for all marine species currently assigned to *Ceratium* (Dinophyceae) Protist 161(1):35-54. DOI: 10.1016/j.protis.2009.06.004

Gómez, F., López-García, P. & Moreira, D. (2011a). Molecular phylogeny of dinophysoid dinoflagellates. The systematic position of *Oxyphysis oxytoxoides* and the *Dinophysis hastata* group (Dinophysales, Dinophyceae). J. Phycol. 47:393–406. DOI: 10.1111/j.1529-8817.2011.00964.x

Gomez, F., López-García, P. & Moreira, D. (2011b). Molecular phylogeny of the sand-dwelling dinoflagellates *Amphidiniopsis hirsuta* and *A*. *swedmarkii* (Peridiniales, Dinophyceae). Acta Protozoologica, 50:141-153. https://doi.org/10.4467/16890027AP.11.024.0024

Gómez, F., López-García, P., Dolan, J.R. & Moreira, D. (2012a). Molecular phylogeny of the marine dinoflagellate genus *Heterodinium* (Dinophyceae). Europ. J. Phycol. 47(2):95-104. DOI: 10.1080/09670262.2012.662722

Gómez, F., Moreira, D. & López-García, P. (2012b). *Sinophysis* and *Pseudophalacroma* are distantly related to typical Dinophysoid dinoflagellates (Dinophysales, Dinophyceae). J. Eukaryot. Microbiol. 59(2):188-190. DOI: 10.1111/j.1550-7408.2011.00598.x

Gómez, F., Onuma, R., Artigas, L.F. & Horiguchi, T. (2015a). A new definition of *Adenoides eludens*, an unusual marine sand-dwelling dinoflagellate without cingulum, and *Pseudadenoides kofoidii* gen. & comb. nov. for the species formerly known as *Adenoides eludens*. Europ. J. Phycol. 50(2):125-138. DOI:10.1080/09670262.2015.1009174

Gómez, F., López-García, P., Takayama, H. & Moreira, D. (2015b). *Balechina* and the new genus *Cucumeridinium* gen. nov. (Dinophyceae), unarmoured dinoflagellates with thick cell coverings. J. Phycol. 51:1088–1105. DOI:10.1111/jpy.12346

Gómez, F., Qiu, D., Lopes, R.M. & Lin, S. (2015c). *Fukuyoa paulensis* gen. et sp. nov., a new genus for the globular species of the dinoflagellate *Gambierdiscus* (Dinophyceae). J. Mol. Evolut. 10(4):e0119676. https://doi.org/10.1371/journal.pone.0119676

Gómez, F., Takayama, H., Moreira, D. & López-García, P. (2016a). Unarmoured dinoflagellates with a small hyposome: *Torodinium* and *Lebouridinium* gen. nov. for *Katodinium glaucum* (Gymnodiniales, Dinophyceae). Europ. J. Phycol. 51(2):226-241. https://doi.org/10.1080/09670262.2015.1126767

Gómez, F., Wakeman, K.C., Yamaguchi, A. & Nozaki, H. (2016b). Molecular phylogeny of the marine planktonic dinoflagellate *Oxytoxum* and *Corythodinium* (Peridiniales, Dinophyceae). Acta Protozool. 55(4):239–248. https://doi.org/10.4467/16890027AP.16.026.6095

Gómez, F., Qiu, D., Dodge, J.D., Lopes, R.M. & Lin, S. (2016c). Morphological and molecular characterization of *Ptychodiscus noctiluca* revealed the polyphyletic nature of the order Ptychodiscales (Dinophyceae). J. Phycol. 52:793–805. DOI: 10.1111/jpy.12438

Gómez, F., Qiu, D., Otero-Morales, E., Lopes, R.M. & Lin, S. (2016d). Circumtropical distribution of the epiphytic dinoflagellate *Coolia malayensis* (Dinophyceae): Morphology and molecular phylogeny from Puerto Rico and Brazil. Phycol. Res. 64(3):194-199. https://doi-org.libproxy.lib.unc.edu/10.1111/pre.12134

Gómez, F., Kiriakoulakis, K. & Lara, E. (2017a). *Achradina pulchra*, a unique dinoflagellate (Amphilothales, Dinophyceae) with a Radiolarian-like endoskeleton of celestite (strontium sulfate). Acta Protozool. 56:71–76. DOI: 10.4467/16890027AP.17.006.7481

Gómez, F., Qiu, D., Lopes, R.M. & Lin, S. (2017b). Morphological and molecular characterization of the toxic dinoflagellate *Ostreopsis* cf. *ovata* (Gonyaulacales: Dinophyceae) from Brazil (South Atlantic Ocean). Rev. Biol. Trop. 65(3):1022-1032. http://dx.doi.org/10.15517/rbt.v65i3.29436

Gómez, F., Qiu, D. & Lin, S. (2017c). The synonymy of the toxic dinoflagellates *Prorocentrum mexicanum* and *P. rhathymum* and the description of *P. steidingerae* sp. nov. (Prorocentrales, Dinophyceae). J. Eukaryot. Microbiol. 64:668–677. DOI: 10.1111/jeu.12403

Gómez, F., Richlen, M. L. & Anderson, D. M. (2017d). Molecular characterization and morphology of *Cochlodinium strangulatum*, the type species of *Cochlodinium*, and *Margalefidinium* gen. nov. for *C. Polykrikoides* and allied species (Gymnodiniales, Dinophyceae). Harmful Algae 63:32–44. DOI: 10.1016/j.hal.2017.01.008

Gómez, F., Artigas, L.F. & Gast, R.J. (2019a). Molecular phylogeny of the parasitic dinoflagellate *Syltodinium listii* (Gymnodiniales, Dinophyceae) and generic transfer of *Syltodinium* undulans comb. nov. (=Gyrodinium undulans). Europ. J. Phycol. 71:125636. https://doi.org/10.1016/j.ejop.2019.125636

Gómez, F., Nakamura, Y. & Artigas, L.F. (2019b). Molecular phylogeny of the sand-dwelling dinoflagellate *Planodinium striatum* and *Chrysodinium* gen. nov. for *Plagiodinium ballux* (Dinophyceae). Acta Protozool. 58: 115–124. DOI: 10.4467/16890027AP.19.012.11421.

Gottschling, M., Keupp, H., Plötner, J., Knop, R., Willems, H. & Kirsch, M. (2005a). Phylogeny of calcareous dinoflagellates as inferred from ITS and ribosomal sequence data. Mol. Phylogenet. Evol. 36:444–55. https://doi.org/10.1016/j.ympev.2005.03.036

Gottschling, M., Knop, R., Plötner, J., Kirsch, M., Willems, H. & Keupp, H. (2005b). A molecular phylogeny of *Scrippsiella* sensu lato (Calciodinellaceae, Dinophyta) with interpretations on morphology and distribution. Europ. J. Phycol. 40:207–20. DOI:10.1080/09670260500109046

Gottschling, M., Renner, S.S., Meier, K.J.S, Willems, H. & Keupp, H. (2008). Timing deep divergence events in calcareous dinoflagellates. J. Phycol. 44:429–438. DOI: 10.1111/j.1529-8817.2008.00479.x

Gottschling, M. & McLean, T.I. (2013). New home for tiny symbionts: Dinophytes determined as zooxanthella are Peridiniales and distantly related to *Symbiodinium*. Mol. Phylogenet. Evol. 67(1):217-222. https://doi.org/10.1016/j.ympev.2013.01.003

Gottschling, M., Knop, R., Plotner, J., Kirsch, M., Willems, H. & Keupp, H. (2005). A molecular phylogeny of *Scrippsiella* sensu lato (Calciodinellaceae, Dinophyta) with interpretations on morphology and distribution. Europ. J. Phycol. 40:207–20. DOI: 10.1016/j.ympev.2005.03.036

Gottschling, M., Söhner, S., Zinßmeister, C., John, U., Plötner, J., Schweikert, M., Aligizaki, K. & Elbrächter, M. (2012). Delimitation of the Thoracosphaeraceae (Dinophyceae), including the calcareous dinoflagellates, based on large amounts of ribosomal RNA sequence data. Protist 163:15–24. doi:10.1016/j.protis.2011.06.003

Gottschling, M., Chacón, J., Žerdoner Čalasan, A., Neuhaus, S., Kretschmann, J., Stibor, H. & John, U. (2019a). Phylogenetic placement of environmental sequences using taxonomically reliable databases helps to rigorously assess dinophyte biodiversity in Bavarian lakes (Germany). Freshw. Biol. DOI: 10.1111/fwb.13413

Gottschling, M., Tillmann, U., Elbrächter, M., Kusber, W.-H., & Hoppenrath, M. (2019b). *Glenodinium triquetrum* Ehrenb. is a species not of *Heterocapsa* F. Stein but of *Kryptoperidinium* Er.Lindem. (Kryptoperidiniaceae, Peridiniales). Phytotaxa 391(2):155–158. https://www.mapress.com/j/pt/

Gottschling, M. et al. (2020). Phylogenetic placement of environmental sequences using taxonomically reliable databases helps to rigorously assess dinophyte biodiversity in Bavarian lakes (Germany). Freshw. Biol. 65:193–208. https://doi.org/10.1111/fwb.13413

Gottschling, M., Carbonell-Moore, M.C., Mertens, K.N., Kirsch, M., Elbrächter, M. & Tillmann, U. (2021a). Fensomea setacea, gen. & sp. nov. (Cladopyxidaceae, Dinophyceae), is neither gonyaulacoid nor peridinioid as inferred from morphological and molecular data. Sci. Rep. 11:12824. https://doi.org/10.1038/s41598-021-92107-0

Gottschling, M., L. Czech, Mahé, F., Adlf, S. & Dunthorn, M. (2021b). The windblown: possible explanations for dinophyte DNA in forest soils. J. Eukaryot. Microbiol. 68(1):e12833. DOI: 10.1111/jeu.12833

Gribble, K.E. & Anderson, D.M. (2006). Molecular phylogeny of the heterotrophic dinoflagellates, *Protoperidinium*, *Diplopsalis* and *Preperidinium* (Dinophyceae), inferred from large subunit rDNA. J. Phycol. 42:1081-1095. https://doi.org/10.1111/j.1529-8817.2006.00267.x

Grzebyk, D., Sako, Y. & Berland, B. (1998). Phylogenetic analysis of nine species of *Prorocentrum* (Dinophyceae) inferred from 18S ribosomal DNA sequences, morphological comparisons, and description of *Prorocentrum panamensis*, sp. nov. J. Phycol. 34:1055-1068. https://doi.org/10.1046/j.1529-8817.1998.341055.x

Gu, H., Kirsch, M., Zinssmeister, C., Soehner, S., Meier, K.J.S., Liu, T. & Gottschling, M. (2013a). Waking the dead: morphological and molecular characterization of extant *Posoniella tricarinelloides* (Thoracosphaeraceae, Dinophyceae). Protist 164(5):583-597. DOI: 10.1016/j.protis.2013.06.001

Gu, H.F., Zeng, N., Liu, T.T., Yang, W.D., Muller, A. & Krock, B. (2013b). Morphology, toxicity, and phylogeny of *Alexandrium* (Dinophyceae) species along the coast of China. Harmful Algae 27:68–81. https://doi.org/10.1016/j.hal.2013.05.008

Gu, H., Luo, Z., Liu, T. & Lan, D. (2013c). Morphology and phylogeny of *Scrippsiella enormis* sp. nov. and *S. cf. spinifera* (Peridiniale, Dinophyceae) from the China Sea. Phycologia 52(2):182-190. DOI: 10.2216/12-036

Gu, H., Luo, Z., Zeng, N., Lan, B. & Lan, D. (2013d). First record of *Pentapharsodinium* (Peridiniales, Dinophyceae) in the China Sea, with description of *Pentapharsodinium dalei* var*. aciculiferum*. Phycol. Res. 61:(4):256-267. https://doi-org.libproxy.lib.unc.edu/10.1111/pre.12024

Gu, H., Liu, T., Vale, P. & Luo, Z. (2013e). Morphology, phylogeny and toxin profiles of *Gymnodinium inusitatum* sp. nov., *Gymnodinium catenatum* and *Gymnodinium microreticulatum* (Dinophyceae) from the Yellow Sea, China. Harmful Algae 28:97-107. https://doi.org/10.1016/j.hal.2013.06.001

Gu, H., Luo, Z., Mertens, K.N., Price, A.M., Turner, R.E. & Rabalais, N.N. (2015a). Cyst-motile stage relationship, morphology, ultrastructure, and molecular phylogeny of the gymnodinioid dinoflagellate *Barrufeta resplendens* comb. nov., formerly known as *Gyrodinium resplendens*, isolated from the Gulf of Mexico. J. Phycol. 51:990–99. DOI: 10.1111/jpy.12342

Gu, H., Liu, T. & Mertens, K.N. (2015b). Cyst-theca relationship and phylogenetic positions of *Protoperidinium* (Peridiniales, Dinophyceae) species of the sections *Conica* and *Tabulata*, with description of *Protoperidinium shanghaiense* sp. nov. Phycologia 54:49–66. https://doi.org/10.2216/14-047.1

Gu, H., Mertens, K.N. & Liu, T. (2016) *Huia caspica* gen. & comb. nov., a dinoflagellate species that recently crossed the marine-freshwater boundary. Phycol. Res. doi: 10.1111/pre.12146

Gu, H., Li, X., Chomerat, N., Luo, Z., Sarno, D., Gourvil, P., Balzano, S. & Siano, R. (2018). *Adenoides sinensis*, a new sand-dwelling dinoflagellate species from China and reexamination of *Adenoides eludens* from an Atlantic strain. Phycologia 57(2):179-190. https://doi.org/10.2216/17-76.1

Guillou, L., Nézan, E., Cueff, V., Erard-Le Denn, E., Cambon-Bonavita, M., Gentien, P. & Barbier, G. (2002). Genetic diversity and molecular detection of three toxic dinoflagellate genera (*Alexandrium*, *Dinophysis*, and *Karenia*) from French coasts. Protist 153:223– 38. DOI: 10.1078/1434-4610-00100

Guillou, L., Viprey, M., Chambouvet, A., Welsh, R. M., Kirkham, A. R., Massana, R., Scanlan, D. J. & Worden, A. Z. (2008). Widespread occurrence and genetic diversity of marine parasitoids belonging to Syndiniales (Alveolata). Environ. Microbiol. 10:3349–3365. https://doi-org.libproxy.lib.unc.edu/10.1111/j.1462-2920.2008.01731.x

Gunderson, J.H., Goss, S.H. & Coats D.W. (1999). The phylogenetic position of *Amoebophrya* sp. infecting *Gymnodinium sanguineum*. J. Eukaryot. Microbiol. 46:194-197. https://doi.org/10.1111/j.1550-7408.1999.tb04603.x

Gunderson, J.H., John, S.A., Boman, II W.C. & Coats, D.W. (2002). Multiple strains of the parasitic dinoflagellate *Amoebophrya* exist in Chesapeake Bay. J. Eukaryot. Microbiol. 49469-474. DOI:10.1111/j.1550-7408.2002.tb00230.x

Gurdebek, P.R., Mertens, K.N., Pospelova, V., Matsuoka, K., Li, Z., Gribble, K.E., Gu, H., Bogus, K., Vrielinck, H. & Louwye, S. (2020). Taxonomic revision, phylogeny, and cyst wall composition of the dinoflagellate cyst genus *Votadinium* Reid (Dinophyceae, Peridiniales, Protoperidiniaceae). Palynology 44(2):310-335. https://doi.org/10.1080/01916122.2019.1580627

Hamilton, K.M., Morritt, D. & Shaw, P.W. (2010). Genetic diversity of the crustacean parasite *Hematodinium* (Alveolata, Syndinea). Eur. J. Protistol. 46(1):17-28. DOI: 10.1016/j.ejop.2009.10.002.

Handy, S.M., Bachvaroff, T.R., Timme, R.E., Coats, D.W., Kim, S. & Delwiche, C.F. (2009). Phylogeny of four dinophysiacean genera (dinophyceae, dinophysiales) based on rDNA sequences from single cells and environmental samples. J. Phycol. 45(5):1163-1174. https://doi.org/10.1111/j.1529-8817.2009.00738.x

Hansen, G. & Daugbjerg, N. (2004). Ultrastructure of *Gyrodinium spirale*, the type species of Gyrodinium (Dinophyceae), including a phylogeny of *G. dominans*, *G. rubrum* and *G. spirale* deduced from partial LSU rDNA sequences. Protist 155: 271-294. https://doi.org/10.1078/1434461041844231

Hansen, G. & Daugbjerg, N. (2011). *Moestrupia oblonga* gen. et comb. nov. (syn.: *Gyrodinium oblongum)*, a new marine dinoflagellate genus characterized by light and electron microscopy, photosynthetic pigments and LSU rDNA sequence. Phycologia 50:583–599. DOI: 10.2216/11-11.1

Hansen, G., Daugbjerg, N. & Henriksen, P. (2000). Comparative study of *Gymnodinium mikimotoi* and *Gymnodinium aureolum*, comb. nov. (= *Gyrodinium aureolum*) based on morphology, pigment composition, and molecular data. J. Phycol. 36(2):394–410. https://doi.org/10.1046/j.1529-8817.2000.99172.x

Hansen, G., Daugbjerg, N. & Franco, J.M. (2003). Morphology, toxin composition and LSU rDNA phylogeny of *Alexandrium minutum* (Dinophyceae) from Denmark, with some morphological observations on other European strains. Harmful Algae 2(4):317-335. https://doi.org/10.1016/S1568-9883(03)00060-X

Hansen, G., Daugbjerg, N. & Henriksen, P. (2007a). *Baldinia anaunienss*is gen. et sp. nov.: a 'new' dinoflagellate from Lake Tovel, N. Italy. Phycologia 46: 86–108. DOI: 10.2216/PH06-23.1

Hansen, G., Botes, L. & De Salas, M. (2007b). Ultrastructure and large subunit rDNA sequences of *Lepidodinium viride* reveal a close relationship to *Lepidodinium chlorophorum* comb. nov. (=*Gymnodinium chlorophorum*). Phycol. Res. 55, 25–41 (2007). https://doi.org/10.1111/j.1440-1835.2006.00442.x

Hansen, G., Daugbjerg, N. & Moestrup, Ø. (2018). The rainwater rock-pool dinoflagellate *Nottbeckia ochracea* gen. et comb. nov. (syn.: *Hemidinium ochraceum*) - A fine-structural and molecular study with emphasis on the motile stage. Protist 169(2):280-306. https://doi.org/10.1016/j.protis.2018.02.003

Harada, A., Ohtsuka, S. & Horiguchi, T. (2007). Species of the parasitic genus *Duboscquella* are members of the enigmatic marine Alveolate Group I. Protist 158: 337-347. https://doi.org/10.1016/j.protis.2007.03.005

Haywood, A.J., Steidinger, K.A., Truby, E.W., Bergquist, P.R., Bergquist, P.L., Adamson, J. & Mackenzie, L. (2004). Comparative morphology and molecular phylogenetic analysis of three new species of the genus *Karenia* (Dinophyceae) from New Zealand. J. Phycol. 40: 165– 79. https://doi.org/10.1111/j.0022-3646.2004.02-149.x

Hehenberger, E., James, E.R., del Campo, J., Buckland-Nicks, J.A., Reimchen, T.E. & Keeling, P.J. (2017). Fish parasite dinoflagellates *Haidadinium ichthyophilum* and *Piscinoodinium* share a recent common ancestor. J. Eukaryot. Microbiol. 65(1) :127-131. DOI: 10.1111/jeu.12430

Henrichs, D.W., Sosik, H.M., Olson, R.J. & Campbell, L. (2011). Phylogenetic analysis of *Brachidinium capitatum* (Dinophyceae) from the Gulf of Mexico indicates membership in the Kareniaceae. J. Phycol. 47:366–374. DOI: 10.1111/j.1529-8817.2011.00960.x

Hoppenrath, M. & Leander, B. S. (2007). Morphology and phylogeny of the pseudocolonial dinoflagellates *Polykrikos lebourae* and *Polykrikos herdmanae* n. sp. Protist 158:209–27. https://doi.org/10.1016/j.protis.2006.12.001

Hoppenrath, M., Saldarriaga, J.F., Schweikert, M., Elbrächter, M. & Taylor, F.J.R. (2004). Description of *Thecadinium mucosum* sp. nov. (Dinophyceae), a new sand-dwelling, marine dinoflagellate, and an emended description of *Thecadinium inclinatum* Balech. J. Phycol. 40:946-961. https://doi.org/10.1111/j.1529-8817.2004.03045.x

Hoppenrath, M., Horiguchi, T., Miyoshi, Y., Selina, M., Taylor, F.J.R. & Leander, B.S. (2007). Taxonomy, phylogeny, biogeography, ecology of *Sabulodinium undulatum*, including an amended description of the species. Phycol. Res. 55:159–175. DOI:10.1111/j.1440-1835.2007.00459.x

Hoppenrath, M. & Leander, B. S. (2008). Morphology and molecular phylogeny of a new marine sand-dwelling *Prorocentrum* species, *P. tsawwassenense* sp. nov. (Dinophyceae, Prorocentrales) from British Columbia, Canada. J. Phycol. 44:451–66. DOI: 10.1111/j.1529-8817.2008.00483.x

Hoppenrath, M., Bachvaroff, T.R., Handy, S.M., Delwiche, C.F. & Leander, B.S. (2009). Molecular phylogeny of ocelloid-bearing dinoflagellates (Warnowiaceae) as inferred from SSU and LSU rDNA sequences. BMC Evol. Biol. 9:116. DOI:10.1186/1471-2148-9-116

Hoppenrath, M., Murray, S., Sparmann, S.F. & Leander, B.S. (2012a). Morphology and molecular phylogeny of *Ankistrodinium* gen. nov. (dinophyceae), a new genus of marine sand-dwelling dinoflagellates formerly classified within *Amphidinium*. J. Phycol. 48:1143-52. DOI: 10.1111/j.1529-8817.2012.01198.x

Hoppenrath, M., Selina, M., Yamaguchi, A. & Leander, B. (2012b). Morphology and molecular phylogeny of *Amphidiniopsis rotundata* sp. nov. (Peridiniales, Dinophyceae), a benthic marine dinoflagellate. Phycologia 51:157–167. DOI: 10.2216/11-35.1

Hoppenrath, M., Chomérat, N. & Leander, B.S. (2013) Molecular phylogeny of *Sinophysis*: Evaluating the possible early evolutionary history of dinophysoid dinoflagellates. Lewis, J.M., Marret, F.,

Bradley, L. [Eds.]. Biological and Geological Perspectives of Dinoflagellates. The Micropalaeontological Society, Special Publications, Geological Society, London. https://doi.org/10.1144/TMS5.19

Hoppenrath, M., Yubuki, N., Stern, R. & Leander, B.S. (2017). Ultrastructure and molecular phylogenetic position of a new marine sand-dwelling dinoflagellate from British Columbia, Canada: *Pseudadenoides polypyrenoides* sp. nov. (Dinophyceae). Europ. J. Phycol. 52(2):208-224. https://doi-org.libproxy.lib.unc.edu/10.1080/09670262.2016.1274788

Hoppenrath, M., Kretzschmar, A.L., Kaufmann, M.J. & Murray, S.A. (2019). Morphological and molecular phylogenetic identification and record verification of *Gambierdiscus excentricus* (Dinophyceae) from Madeira Island (NE Atlantic Ocean). Mar. Biodivers. Rec. 12:16. https://doi.org/10.1186/s41200-019-0175-4

Hoppenrath, M., Reñé, A., Satta, C.T., Yamaguchi, A. & Leander, B.S. (2020). Morphology and molecular phylogeny of a new marine, sand-dwelling dinoflagellate genus, *Pachena* (Dinophyceae), with descriptions of three new species. 56(3):798-817. https://doi.org/10.1111/jpy.12984

Howard, M.D.A., Smith, G.J. & Kudela, R.M. (2009). Phylogenetic relationships of yessotoxin-producing dinoflagellates, based on the large subunit and internal transcribed spacer ribosomal DNA domains. Appl. Environ. Microbiol. 75:54–63. doi:10.1128/AEM.00818-08

Horiguchi, T., Yoshizawa-Ebata, J. & Nakayama, T. (2000). *Halostylodinium arenarium*, gen. et sp. nov. (Dinophyceae), a coccoid sand-dwelling dinoflagellate from subtropical Japan. J. Phycol. 36: 960–71. https://doi.org/10.1046/j.1529-8817.2000.00007.x

Horiguchi, T., Hayashi,Y., Kudo H. & Hara, Y. (2011). A new benthic dinoflagellate *Spiniferodinium palauense* sp. nov. (Dinophyceae) from Palau. Phycologia 50: 616–623. DOI: 10.2216/11-15.1

Horiguchi, T., Tamura M., Katsumata K. & Yamaguchi, A. (2012). *Testudodinium* gen. nov. (Dinophyceae), a new genus of sand-dwelling dinoflagellates formerly classified in the genus *Amphidinium*. Phycol. Res. 60(2):137-149. https://doi-org.libproxy.lib.unc.edu/10.1111/j.1440-1835.2012.00645.x

Horiguchi, T., Moriya, R., Pinto, S. K. &Terada, R. (2017). *Pyramidodinium spinulosum* sp. nov. (Dinophyceae), a sand-dwelling non-motile dinoflagellate from the seafloor (36 m deep) off Mageshima Island, Kagoshima, Japan. Phycol. Res. 65:272–279 DOI: 10.1111/pre.12183.

Hu, Z., Deng, Y., Li, Y. & Tang, Y.Z. (2018). The morphological and phylogenetic characterization for the dinoflagellate *Margalefidinium fulvescens* (=*Cochlodinium fulvescens*) isolated from the Jiaozhou Bay, China. Acta Oceanol. Sin. 37(10):11-17. DOI: 10.1007/s13131-018-1295-0

Hu, Z., Deng, Y., Luo, Z., Shang, L., Kong, F., Gu, H., Zhao, Z. & Tang, Y.Z. (2019). Characterization of the unarmored dinoflagellate *Pseliodinium pirum* (Ceratoperidiniaceae) from Jiaozhou Bay, China. Phycol. Res. https://doi.org/10.1111/pre.12385

Hu, Z., Li, Z., Deng, Y., Iwataki, M., Luo, Z., Wang, J., Sun, Y., Zhao, Z., Gu, H., Shin, H.H. & Tang, Y.Z. (2020). Morphology, ultrastructure, and molecular phylogeny of the unarmoured dinoflagellate

Kirithra sigma sp. nov. (Ceratoperidiniaceae, Dinophyceae). Phycologia 59(5):385-396. DOI: 10.1080/00318884.2020.1771660

Hume, B.C., D'Angelo, C., Smith, E.G., Stevens, J.R., Burt, J. & Wiedenmann, J. (2015). *Symbiodinium thermophilum* sp. nov., a thermotolerant symbiotic alga prevalent in corals of the world's hottest sea, the Persian/Arabian Gulf. Sci. Rep. 5:8562. DOI: 10.1038/srep08562

Iwataki, M., Hansen, G., Sawaguchi, T., Hiroishi, S. & Fukuyo, Y. (2004). Investigations of body scales in twelve *Heterocapsa* species (Peridinales, Dinophyceae), including a new species *H. pseudotriquetra* sp. nov. Phycologia 43:394–403. DOI: 10.2216/i0031-8884-43-4-394.1

Iwataki, M., Kawami, H. & Matsuoka, K. (2007). *Cochlodinium fulvescens* sp. nov. (Gymnodiniales, Dinophyceae), a new chain-forming unarmored dinoflagellate from Asian coasts. Phycol. Res. 55:231-239. https://doi.org/10.1111/j.1440-1835.2007.00466.x

Iwataki, M., Kawami, H., Mizushima, K., Mikulski, C.M., Doucette, G.J., Relox, J.R. Jr., Anton, A., Fukuyo, Y. & Matsuoka, K. (2008). Phylogenetic relationships in the harmful dinoflagellate *Cochlodinium polykrikoides* (Gymnodiniales, Dinophyceae) inferred from LSU rDNA sequences. Harmful Algae 7(3):271-277. https://doi.org/10.1016/j.hal.2007.12.003

Jang, S.H., Jeong, J.J., Moestrup, O., Kang, N.S., Lee, S.Y., Lee, K.H., Lee, M.J. & Noh, J.H. (2015). Morpological, molecular and ecophysiological characterization of the phototrophic dinoflagellate *Biecheleriopsis adriatica* from Korean coastal waters. Europ. J. Phycol. 50(3):301-317. https://doi.org/10.1080/09670262.2015.1054892

Jang, S.H., Jeong, H.J., Kwon, J.E. & Lee, K.H. (2017a). Mixotrophy in the newly described dinoflagellate *Yihiella yeosuensis*: a small, fast dinoflagellate predator that grows mixotrophically, but not autotrophically. Harmful Algae. 62:94–103. DOI: 10.1016/j.hal.2016.12.007

Jang, S.H., Jeong, H.J., Moestrup, Ø., Kang, N.S., Lee, S.Y., Lee, K.H. & Seong, KA. (2017b). *Yihiella yeosuensis* gen. et sp. nov. (Suessiaceae, Dinophyceae), a novel dinoflagellate isolated from the coastal waters of Korea. J. Phycol. 53:131–145. https://doi.org/10.1111/jpy.12486

Jang, S.H., Jeong, H.J. & Yoo, Y.D. (2018). *Gambierdiscus jejuensis* sp. nov., an epiphytic dinoflagellate from the waters of Jeju Island, Korea, effect of temperature on the growth, and its global distribution. Harmful Algae. 80:149-157. https://doi.org/10.1016/j.hal.2018.11.007

Janson, S., Gisselson, L.A, Salomon, P.S & Granéli, E. 2000. Evidence for multiple species within the endoparasitic dinoflagellate *Amoebophrya ceratii* as based on 18S rRNA gene-sequence analysis. Parasitol. Res. 86:929-933. DOI: 10.1007/s004360000272

Jedlicki, A., Fernández, G., Astorga, M., Oyarzún, P., Toro, J.E., Navarro, J.M. & Martínez, V. (2012). Molecular detection and species identification of *Alexandrium* (Dinophyceae) causing harmful algal blooms along the Chilean coastline. AoB Plants: 2012:pls033. DOI: 10.1093/aobpla/pls033

Jensen, M.H. & Daugbjerg, N. (2009). Molecular phylogeny of selected species of the order Dinophysiales (Dinophyceae) –testing the hypothesis of a dinophysioid radiation. J. Phycol. 45:1136–1152. DOI: 10.1111/j.1529-8817.2009.00741

John, U., Litaker, W., Montresor, M., Murray, S., Broshanan, M. & Anderson, D. (2014). Formal revision of the *Alexandrium tamarense* species complex (Dinophyceae) taxonomy: The introduction of five species with emphasis on molecular-based (rDNA) classification. Protist 63:932-933. https://doi.org/10.1016/j.protis.2014.10.001

Jeong, H.J., Kim, J.S., Park, J.Y., Kim, J.H., Kim, S., Lee, I., Lee, S.H., Ha, J.H. & Yih, W.H. (2005). *Stoeckeria algicida* n. gen., n. sp. (Dinophyceae) from the coastal waters off Southern Korea: morphology and small subunit ribosomal DNA gene sequence. J. Eukaryot. Microbiol. 52(4): 382-390. DOI: 10.1111/j.1550-7408.2005.00051.x

Jeong, H.J., Yih, W., Kang, N., Lee, S.Y., Yoon, E.Y., Yoo, Y.D., Kim, H.S. & Kim, J.H. (2012). First report of the epiphytic benthic dinoflagellates *Coolia canariensis* and *Coolia malayensis* in the waters off Jeju Island, Korea: morphology and rDNA sequences. J. Eukaryot. Microbiol. 59(2):114-33. DOI: 10.1111/j.1550-7408.2012.00610.x

Jeong, H.J., Jang, S.H., Moestrup, Ø., Kang, N.S., Lee, S.Y., Potvin, É. & Noh, J.H. (2014a). *Ansanella granifera* gen. et. sp. nov. (Dinophyceae), a new dinoflagellate from the coastal waters of Korea. Algae 29(2):75-99. DOI: https://doi.org/10.4490/algae.2014.29.2.075

Jeong, H.J., Lee, S. Y., Kang, N. S., Yoo, Y. D., Lim, A. S., Lee, M. J., Kim, H. S., Yih, W., Yamashita, H. & LaJeunesse, T.C. (2014b). Genetics and morphology characterize the dinoflagellate *Symbiodinium voratum*, n. sp. (Dinophyceae) as the sole representative of *Symbiodinium* Clade E. J. Eukaryot. Microbiol. 61:75–94. DOI: 10.1111/jeu.12088

Jeong, H.J., Kang, N.S., Moestrup, Ø., Yoo, Y.D. & Potvin, E. (2014c). Description of the new heterotrophic dinoflagellate Stoeckeria changwonensis n. sp from Korean coastal waters and emended description of the genus Stoeckeria and the type species *Stoeckeria algicida*. Harmful Algae 36:38-56. https://doi.org/10.1016/j.hal.2014.04.017

Jørgensen, M.F., Murray, S. & Daugbjerg, N. (2004a). *Amphidinium* revisited. I. Redefinition of Amphidinium (dinophyceae) based on cladistic and molecular phylogenetic analyses. J. Phycol. 40(2):351-365. https://doi.org/10.1111/j.1529-8817.2004.03131.x

Jørgensen, M.,F., Murray S. & Daugbjerg N. (2004b). A new genus of athecate interstitial dinoflagellates, *Togula* gen. nov., previously encompassed within *Amphidinium* sensu lato: Inferred from light and electron microscopy and phylogenetic analyses of partial large subunit ribosomal DNA sequences. Phycol. Res. 52:284-299. https://doi.org/10.1111/j.1440-1835.2004.tb00338.x

Jung, J.-H., Choi, J. M., Coats, D.W. & Kim, Y.-O. (2015). *Euduboscquella costata* n. sp. (Dinoflagellata, Syndinea), an intracellular parasite of the ciliate Schmidingerella arcuata: Morphology, molecular phylogeny, life cycle, prevalence, and infection intensity. 63(1):3-15. DOI: 10.1111/jeu.12231

Jung, J.-H., Choi, J. M. & Kim, Y.-O. (2018). Genus-specific PCR primers targeting intracellular parasite *Euduboscquella* (Dinoflagellata: Syndinea). Ocean Sci. J. 53:81–90. http://dx.doi.org/10.1007/s12601-017-0063-y

Karafas, S., Teng, S.T., Leaw, C.P. & Alves-de-Souza, C. (2017). An evaluation of the genus *Amphidinium* (Dinophyceae) combining evidence from morphology, phylogenetics, and toxin production, with the introduction of six novel species. Harmful Algae 68:128–151. DOI: 10.1016/j.hal.2017.08.001

Kang, N.S., Jeong, H.J., Lee, S.Y., Lim, A.S., Lee, M.J., Kim, H.S. & Yih, W. (2013). Morphology and molecular characterization of the epiphytic benthic dinoflagellate *Ostreopsis* cf. *ovata* in the temperate waters off Jeju Island, Korea. Harmful Algae 27:98-112. DOI: 10.1016/j.hal.2013.05.006

Kang, N.S., Jeong, H.J., Moestrup, Ø., Jang, T.Y., Lee, S.Y. & Lee, M.J. (2015). *Aduncodinium* gen. nov and *A. glandula* comb. nov (Dinophyceae, Pfiesteriaceae), from coastal waters off Korea: Morphology and molecular characterization. Harmful Algae 41:25–37. https://doi.org/10.1016/j.hal.2014.11.002

Kang, W. & Wang, Z.-H. (2018). Identification of a marine woloszynskioid dinoflagellate *Biecheleriopsis adriatica* and germination of its cysts from southern Chinese coasts. J. Environ. Sci. 66:246-254. https://doi.org/10.1016/j.jes.2017.04.031

Kawami, H., Iwataki, M. & Matsuoka, K. (2006) A new diplopsalid species *Oblea acanthocysta* sp. nov. (Peridiniales, Dinophyceae). Plankton Benthos Res. 1(4):183–190. DOI:10.3800/pbr.1.183

Kim, J.-H., Tillmann, U., Adams, N.G., Krock, B., Stutts, W.L., Deeds, J.R., Han, M.S. & Trainer, V.L. (2017). Identification of *Azadinium* species and a new azaspiracid from *Azadinium poporum* in Puget Sound, Washington State, USA. Harmful Algae 68:152-167. DOI: 10.1016/j.hal.2017.08.004

Kim, H.-J., Li, Z., Kang, N.S., Gu, H., Kim, D., Seo, M.H., Lee, S.D., Yun, S.M., Oh, S.-J., & Shin, H.H. (2021). Morphology and Phylogeny of *Scrippsiella precaria* Montresor & Zingone (Thoracosphaerales, Dinophyceae) from Korean Coastal Waters. J. Mar. Sci. Eng. 9(2):154. https://doi.org/10.3390/jmse9020154

Ki, J.-S. (2010). Nuclear 28S rDNA phylogeny supports the basal placement of *Noctiluca scintillans* (Dinophyceae; Noctilucales) in dinoflagellates. Eur. J. Protistol. 46(2):111-20. DOI: 10.1016/j.ejop.2009.11.001

Ki, J.-S., Park, M.-H. & Han, M.-S. (2011). Discriminative power of nuclear rDNA for the DNA taxonomy of the dinoflagellate genus Peridinium (Dinophyceae). J. Phycol. 47:426-435. doi:https://doi.org/10.1111/j.1529-8817.2010.00950.x.

Kim, K.-Y. & Kim, C.-H. (2007). Molecular phylogenetic relationships among diverse dinoflagellate species occurring in coastal waters off Korea inferred from large subunit ribosomal DNA sequence data. Algae 22(2):57-67. https://doi.org/10.4490/ALGAE.2007.22.2.057

Kim K.-Y., Yoshida, M. & Kim, C.-H. (2005b). Molecular phylogeny of three hitherto unreported *Alexandrium* species: *A. hiranoi*, *A. leei* and *A. satoanum* (Gonyaulacales, Dinophyceae) inferred from the 18S and 26S rDNA sequence data. Phycologia 44:361-368. https://doi.org/10.2216/0031-8884(2005)44[361:MPOTHU]2.0.CO;2

Kim, S. & Park, M.G. (2014). *Amoebophrya* spp. from the bloom-forming dinoflagellate *Cochlodinium polykrikoides*: parasites not nested in the "*Amoebophrya ceratii* complex". J. Eukaryot. Microbiol. 61(2):173-81. DOI: 10.1111/jeu.12097

Kim, S. & Park, M.G. (2018). Feeding characteristics and molecular phylogeny of the thecate mixotrophic dinoflagellate *Fragilidium mexicanum*. Harmful Algae 63:154-163. DOI: 10.1016/j.hal.2017.02.007

Kim, S.H., Kim, K.-Y., Kim, C.-H., Lee, W. S., Chang, M. & Lee, J.-H. (2004). Phylogenetic analysis of harmful algal blooming (HAB)-causing dinoflagellates along the Korean coasts based on the SSU rRNA gene. J. Microbiol. Biotechnol. 14: 959-966. DOI: 10.1016/j.hal.2017.02.007

Kim, S., Park, M.G., Kim, K.-Y., Kim, C.-H., Yih, W., Park, J.S. & Coats, D.W. (2008). Genetic diversity of parasitic dinoflagellates in the genus *Amoebophrya* and its relationship to parasite biology and biogeography. J. Eukaryot. Microbiol. 55:1–8. DOI: 10.1111/j.1550-7408.2007.00295.x

Kremp, A., Elbrächter, M., Schweikert, M., Wolny, J.L. & Gottschling, M. (2005). *Woloszynskia halophila* (Biecheler) comb. nov.: A bloom-forming cold-water dinoflagellate co-occurring with Scrippsiella hangoei (Dinophyceae) in the Baltic Sea. J. Phycol. 41:629–642. DOI: 10.1111/j.1529-8817.2005.00070.x.

Kremp, A., Tahvanainen, P., Litaker, W., Krock B., Suikkanen, S., Leaw, C.P. & Tomas, C. (2014). Phylogenetic relationships, morphological variation, and toxin patterns in the *Alexandrium ostenfeldii* (Dinophyceae) complex: implications for species boundaries and identities. 50(1):81-100. https://doi-org.libproxy.lib.unc.edu/10.1111/jpy.12134

Kretschmann, J., Zinssmeister, C. & Gottschling, M. (2014). Taxonomic clarification of the dinophyte *Rhabdosphaera erina*ceus Kamptner, = *Scrippsiella erinaceus* comb. nov. (Thoracosphaeraceae, Peridiniales). Syst. Biodivers. 12: 393-404. https://doi.org/10.1080/14772000.2014.934406

Kretschmann, J., Filipowicz, N.H., Owsianny, P.M., Zinßmeister, C. & Gottschling, M. (2015). Taxonomic clarification of the unusual dinophyte *Gymnodinium limneticum* Wołosz. (Gymnodiniaceae) from the Tatra Mountains. Protist 166:621–37. http://dx.doi.org/10.1016/j.protis.2015.09.002

Kretschmann, J., Owsianny, P.M., Žerdoner Čalasan, A., Gottschling, M. (2018a) The Hot Spot in a Cold Environment: Puzzling *Parvodinium* (Peridiniopsidaceae, Peridiniales) from the Polish Tatra Mountains. Protist. 169(2):206-230. DOI: 10.1016/j.protis.2018.02.004

Kretschmann, J., Žerdoner Čalasan, A. & Gottschling, M. (2018b). Molecular phylogenetics of dinophytes harbouring diatoms asendosymbionts (Kryptoperidiniaceae, Peridiniales), with evolutionary interpretations and a focus on the identity of *Durinskia oculata*. Mol. Phylogen. Evol. 118: 392–402. https://doi.org/10.1016/j.ympev.2017.10.011

Kretzschmar, A.L., Verma, A., Harwood, T., Hoppenrath, M. & Murray, S. (2017). Characterization of *Gambierdiscus lapillus* sp. nov. (Gonyaulacales, Dinophyceae): A new toxic dinoflagellate from the Great Barrier Reef (Australia). J. Phycol. 53(2):283–297. DOI: 10.1111/jpy.12496

Kretzschmar, A.L., Larsson, M.E., Hoppenrath, M., Doblin, M.A. & Murray, S.A. (2019). Characterisation of two toxic *Gambierdiscus* spp. (Gonyaulacales, Dinophyceae) from the Great Barrier Reef

(Australia): *G. lewisii* sp. nov. and *G.holmesii* sp. nov. Protist 170:125699. http://www.elsevier.de/protis

Kühn, S.F. & Medlin, L.K. (2005). The systematic position of the parasitoid marine dinoflagellate *Paulsenella vonstoschii* (Dinophyceae) inferred from nuclear encoded small subunit ribosomal DNA. Protist 156, 393–398. DOI: 10.1016/j.protis.2005.09.002

LaJeunesse, T. C. 2001. Investigating the biodiversity, ecology, and phylogeny of endosymbiotic dinoflagellates in the genus *Symbiodinium* using the ITS region: in search of a "species" level marker. J. Phycol. 37:866-880. https://doi.org/10.1046/j.1529-8817.2001.01031.x

LaJeunesse, T. C. (2005). "Species" Radiations of symbiotic dinoflagellates in the Atlantic and Indo-Pacific since the Miocene-Pliocene transition, Mol. Biol. and Evol. 22(3):570–581. https://doi.org/10.1093/molbev/msi042

LaJeunesse, T.C. (2017). Validation and description of *Symbiodinium microadriaticum*, the type species of *Symbiodinium* (Dinophyta). J. Phycol. 53:1109-1114. https://doi.org/10.1111/jpy.12570

LaJeunesse, T.C., Parkinson, J.E. & Reimer, J.D. (2012). A genetics-based description of *Symbiodinium minutum* sp. nov. and *S. psygmophilum* sp. nov. (Dinophyceae), two dinoflagellates symbiotic with cnidarian. J. Phycol. 48:1380-1391. https://doi.org/10.1111/j.1529-8817.2012.01217.x

LaJeunesse, T.C., Wham, D.C., Pettay, D.T., Parkinson, J.E., Keshavmurthy, S. & Chen, C.A. (2014). Ecologically differentiated stress-tolerant endosymbionts in the dinoflagellate genus *Symbiodinium* (Dinophyceae) Clade D are different species. Phycologia 53:305–319. DOI: 10.2216/13-186.1

LaJeunesse, T.C., Lee, S.Y., Gil-Agudelo, D.L., Knowlton, N. & Jeong, H.J. (2015). *Symbiodinium necroappetens* sp. nov. (Dinophyceae): an opportunist 'zooxanthella' found in bleached and diseased tissues of Caribbean reef corals. Europ. J. Phycol. 50(2):223-238.

LaJeunesse, T.C., Parkinson, J.E., Gabrielson, P.W., Jeong, H.J., Reimer, J.D., Voolstra, C.R. & S.R. Santos. (2018). Systematic revision of Symbiodiniaceae highlights the antiquity and diversity of coral endosymbionts. Curr. Biol. 28(16):1–11. https://doi.org/10.1016/j.cub.2018.07.008

Larsson, M.E., Laczka, O.F., Harwood, D.T., Lewis, R.J., Himaya, S.W.A., Murray, S.A. & Doblin, M.A. (2018). Toxicology of *Gambierdiscus* spp. (Dinophyceae) from tropical and temperate Australian waters. Mar Drugs. 16(1):7. DOI: 10.3390/md16010007

Laza-Martinez, A., Orive, E., & Miguel, I. (2011). Morphological and genetic characterization of benthic dinoflagellates of the genera *Cooli*a, *Ostreopsis* and *Prorocentrum* from the south-eastern Bay of Biscay. Europ. J. Phycol. 46:45-65. https://doi.org/10.1080/09670262.2015.1025857

Leaw, C.P., Lim, P.T., Ng, B.K., Cheah, M.Y., Ahmad, A. & Usup, G. (2005). Phylogenetic analysis of *Alexandrium* species and *Pyrodinium bahamense* (Dinophyceae) based on theca morphology and nuclear ribosomal gene sequence. Phycologia 44: 550–565. DOI: 10.2216/0031-8884(2005)44[550:PAOASA]2.0.CO;2

Leaw, C.P., Lim, P., Cheng, K.W., Ng, B.K. & Usup, G. (2010). Morphology and molecular characterization of a new species of thecate benthic dinoflagellate, *Coolia malayensis* sp. nov. (Dinophyceae) J. Phycol. 46(1):162-171. https://doi.org/10.1111/j.1529-8817.2009.00778.x

Leaw C.P., Tan, T.H., Lim, H.C., Teng, S.T., Yong, H.L., Smith, K.F., Rhodes, L., Wolf, M., Holland, W.C., Vandersea, M.W., Litaker, R.W., Tester, P.A., Gu, H., Usup, G. & Lim, P.T. (2016). New scenario for speciation in the benthic dinoflagellate genus *Coolia* (Dinophyceae). Harmful Algae 55:137-149. DOI: 10.1016/j.hal.2016.02.010

Leblond, J.D., Lasiter, A.D., Li, C., Logares, R., Rengefors, K. & Evens, T.J. (2010). A data mining approach to dinoflagellate clustering according to sterol composition: correlations with evolutionary history. Int. J. Data Min. Bioinform. 4(4): 431–451.

Lee, H.G., Lee, J.Y. & Lee, D.H. (2001). Cloning and Characerization of the Ribosomal RNA Gene from *Gonyaulax polyedra*. J. Microbiol. Biotech. 11(3)515-523.

Lee, K.H., Jeong, H.J., Park, K., Kang, N.S., Yoo, Y.D., Lee, M.J., Lee, J.-W., Lee, S., Kim, T., Kim, H.S. & Noh, J.H. (2013). Morphology and molecular characterization of the epiphytic dinoflagellate *Amphidinium massartii*, isolated from the temperate waters off Jeju Island, Korea. Algae 28(3):213-231. http://dx.doi.org/10.4490/algae.2013.28.3.213

Lee, S.Y., Jeong, H.J., Kang, N.S., Jang, T.Y., Jang, S.H. & LaJeunesse, T.C. (2015). *Symbiodinium tridacnidorum* sp. nov., a dinoflagellate common to Indo-Pacific giant clams, and a revised morphological description of *Symbiodinium microadriaticum* Freudenthal, emended Trench & Blank. Europ. J. Phycol. 50:155-172. https://doi.org/10.1080/09670262.2015.1018336

Lee, S.Y., Jeong, H.J., Kim, S.J., Lee, K.H. & Jang, S.H. (2019). *Scrippsiella masanensis* sp. nov. (Thoracosphaerales, Dinophyceae), a phototrophic dinoflagellate from the coastal waters of southern Korea. Phycologia 58(3):287-299. DOI: 10.1080/00318884.2019.1568794

Lenaers, G., Maroteaux, L., Michot, B. & M. Herzog. (1989). Dinoflagellates in evolution. A molecular phylogenetic analysis of large subunit ribosomal RNA. J. Mol. Evol.29:40-51. https://doi.org/10.1007/BF02106180

Lewis, A.M., Chan, A.N. & LaJeunesse, T.C. (2019). New species of closely related endosymbiotic dinoflagellates in the Greater Caribbean have niches corresponding to host coral phylogeny. J. Eukaryot. Microbiol. 66(3):469-482. https://doi.org/10.1111/jeu.12692

Leung, P., Yan, M., Yiu, S.K., Lam, V.T., Ip, J.C., Au, M.W., Chen, C., Wai, T. & Lam, P.K. (2017). Molecular phylogeny and toxicity of harmful benthic dinoflagellates *Coolia* (Ostreopsidaceae, Dinophyceae) in a sub-tropical marine ecosystem: The first record from Hong Kong. Mar. Pollut. Bull. 124(2):878-889. DOI: 10.1016/j.marpolbul.2017.01.017

Leung, P.T.Y., Yan, M., Lam, V.T.T., Yiu, S.K.F., Chen, C.-Y., Murray, J.S., Harwood, D.T., Rhodes, L.L., Lam, P.K.S. & Wai, T.-C. (2018). Phylogeny, morphology and toxicity of benthic dinoflagellates of the genus *Fukuyoa* (Goniodomataceae, Dinophyceae) from a subtropical reef ecosystem in the South China Sea. Harmful Algae 74:78-97. DOI: 10.1016/j.hal.2018.03.003

Levy, M.G., Litaker, R.W., Goldstein, R.J., Dykstra, M.J., Vandersea, M.W. & Noga, E.J. (2007). *Piscinoodinium*, a fish-ectoparasitic dinoflagellate, is a member of the class Dinophyceae, subclass Gymnodiniphycidae: convergent evolution with Amyloodinium. J. Parasitol. 93(5):1006-1015. 10.1645/GE-3585.1

Li, Z., Shin, H.H. & Han, M.-S. (2015). Morphology and phylogeny of a new woloszynskioid dinoflagellate *Tovellia paldangensis* sp. nov. (Dinophyceae). Phycologia 54(1):67–77. DOI: 10.2216/14-080.1

Li, Z. & Shin, H.H. (2018). Morphology and phylogeny of an unarmored dinoflagellate, *Karlodinium jejuense* sp. nov. (Gymnodiniales), isolated from the northern East China Sea. Phycol. Res. 66: 318– 28. DOI: 10.1111/pre.12334

Li, Z. & Shin, H.H (2019). Morphology, phylogeny and life cycle of *Fragilidium mexicanum* Balech (Gonyaulacales, Dinophyceae). Phycologia 58(4):419-432. DOI: 10.1080/00318884.2019.1620582

Li, Z., Shin, H.H., Lim, W.A., Lee, T., Yoon, Y.H. & Han, M.S. (2015). Morphology and phylogeny of *Pentapharsodinium jinhaense* sp. nov. (Dinophyceae) producing a calcareous resting cyst. Phycologia 54:566-577. DOI 10.2216/15-50.1

Li, Z., Oh, S.J., Park, J.-W., Lim, W.-A. & Shin, H.H. (2017). Cyst-motile stage relationship, morphology and phylogeny of a new chain-forming, marine dinoflagellate *Grammatodinium tongyeonginum* gen. & sp. nov. from Korea. Phycologia 56(4):430-443. https://doi.org/10.2216/16-88.1

Li, Z., Mertens, K.N., Nézan, E., Chomérat, N., Bilien, G., Iwataki, M. & Shin, H.H. (2019). Discovery of a new clade nested within the genus *Alexandrium* (Dinophyceae): Morpho-molecular characterization of *Centrodinium punctatum* (Cleve) F.J.R. Taylor. Protist 170(2):168-186. DOI: 10.1016/j.protis.2019.02.003

Li, Z., Mertens, K.N, Gottschling, M., Gu, H. Söhner, S., Price, A.M., Marret, F., Pospelova, V., Smith, K.F. Carbonell-Moore, C., Nézan, E., Bilien, G. & Shin, H.H. (2020). Taxonomy and molecular phylogenetics of *Ensiculiferaceae*, fam. nov. (Peridiniales, Dinophyceae), with Consideration of their Life-history. Protist 171(5):125759. DOI: 10.1016/j.protis.2020.125759

Lilly, E.L., Halanych, K.M. & Anderson, D. M. (2005). Phylogeny, biogeography, and species boundaries within the *Alexandrium minutum* group. Harmful Algae. 4:1004–1020. https://doi.org/10.1016/j.hal.2005.02.001

Lilly, E.L., Halanych, K.M. & Anderson, D.M. (2007). Species boundaries and global biogeography of the *Alexandrium tamarense* complex (Dinophyceae). J. Phycol. 43:1329–1338. https://doi.org/10.1111/j.1529-8817.2007.00420.x

Lim, A.S., Jeong, H.J., Jang, T.Y., Kang, N.S., Lee, S.Y., Yoo, Y.D. & Kim, H.S. (2013). Morphology and molecular characterization of the epiphytic dinoflagellate *Prorocentrum* cf. rhathymum in temperate waters off Jeju Island, Korea. Ocean Sci. J. 48(1):1-17. http://dx.doi.org/10.1007/s12601-013-0001-6

Lim, A.S., Jeong, H.J. & Ok, J.H. (2019). Five *Alexandrium* species lacking mixotrophic ability. Algae 34(4):289-301. DOI: https://doi.org/10.4490/algae.2019.34.11.21

Lin, S., Hu, Z., Deng, Y., Shang, L., Gobler, C.J. & Tang, Y.Z. (2020). An assessment on the intrapopulational and intraindividual genetic diversity in LSU rDNA in the harmful algal blooms-forming dinoflagellate *Margalefidinium* (= *Cochlodinium*) *fulvescens* based on clonal cultures and bloom samples from Jiaozhou Bay, China. Harmful Algae. 96:101821. DOI: 10.1016/j.hal.2020.101821

Lindberg, K., Moestrup, Ø. & Daugbjerg, N. (2005). Studies on woloszynskioid dinoflagellates I: *Woloszynskia coronata* re-examined using light and electron microscopy and partial LSU rDNA sequences, with description of *Tovellia* gen. nov. and *Jadwigia* gen. nov. (Tovelliaceae fam. nov.). Phycologia 44: 416-440. DOI: 10.2216/0031-8884(2005)44[416:SOWDIW]2.0.CO;2

Litaker, R.W., Steidinger, K.A., Mason, P.L., Landsberg, J.H., Shields, J.D., Reece, K.S., Haas, L.W., Vogelbein, W.K., Vandersea, M.W., Kibler, S.R. & Tester, P.A. (2005). The reclassification of *Pfiesteria shumwayae* (Dinophyceae): *Pseudopfiesteria*, gen nov. J Phycol. 41:643–651. https://doi.org/10.1111/j.1529-8817.2005.00075.x

Litaker, R. W., M. W. Vandersea, S. R. Kibler, K. S. Reece, N. A. Stokes, F. M. Lutzoni, B. A. Yonish, M. A. West, M. N. D. Black, & P. A. Tester. 2007. Recognizing dinoflagellate species using ITS rDNA sequences. J. Phycol.43:344-355. DOI: 10.1111/j.1529-8817.2007.00320.x

Litaker, R.W., Vandersea, M.W., Faust, M.A., Kibler, S.R., Chinain, M., Holmes, M.J., Holland, W.C. & Tester, P.A. (2009). Taxonomy of *Gambierdiscus* including four new species, Gambierdiscus caribaeus, *Gambierdiscus carolinianus*, *Gambierdiscus carpenteri* and *Gambierdiscus ruetzleri* (Gonyaulacales, Dinophyceae). Phycologia 48:344– 90. https://doi.org/10.2216/07-15.1

Liu, T., Gu, H., Mertens, K.N. & Lan, D. (2013). New dinoflagellate species *Protoperidinium haizhouense* sp. nov. (Peridiniales, Dinophyceae), its cyst-theca relationship and phylogenetic position within the Monovela group. Phycol. Res. 62(2):109–124. DOI:10.1111/pre.12041

Liu, T., Mertens, K.N. & Gu, H. (2015a). Cyst–theca relationship and phylogenetic positions of the diplopsalioideans (Peridiniales, Dinophyceae), with description of *Niea* and *Qia* gen. nov. Phycologia 54(2):210–232. https://doi.org/10.2216/14-94.1

Liu, T., Mertens, K.N., Ribeiro, S., Ellegaard, M., Matsuoka, K. & Gu, H. (2015b). Cyst-theca relationships and phylogenetic positions of Peridiniales (Dinophyceae) with two anterior intercalary plates, with description of *Archaeperidinium bailongense* sp. nov. and *Protoperidinium fuzhouense* sp. nov. Phycol Res. 63(2):134–151. https://doi.org/10.1111/pre.12081

Liu, Y., Chen, Z., Gao, Y., Zou, J., Lu, S. & Zhang, L. (2021). identifying the source organisms producing paralytic shellfish toxins in a subtropical bay in the South China Sea. Environ. Sci. Technol. 55(5):3124-3135. DOI: 10.1021/acs.est.0c06991

Logares, R., K. Rengefors, A. Kremp, K. Shalchian-Tabrizi, A. Boltovskoy, T. Tengs, A. Shurtleff & D. Klaveness. (2007). Phenotypically different microalgal morphospecies with identical ribosomal DNA: A case of rapid adaptive evolution? Microb. Ecol. 53: 549-561. DOI: 10.1007/s00248-006-9088-y

Lum, W. M., Takahashi, K., Benico, G., Takayama, H. & Iwataki, M. (2019). *Dactylodinium arachnoides* sp. nov. (Borghiellaceae, Dinophyceae): a new marine dinoflagellate with a loop-shaped apical

structure complex and tubular membranous extrusomes. Phycologia 58:661-674. https://doi.org/10.1080/00318884.2019.1658399

Luo, Z., Gu, H., Krock, B. & Tillmann, U. (2013). *Azadinium dalianense*, a new dinoflagellate from the Yellow Sea, China. Phycologia 52:625–36. DOI: 10.2216/13-178.1

Luo, Z., Yang, W., Xu, B., Zheng, B. & Gu, H. (2015). Morphology, ultrastructure, and phylogeny of *Protodinium* simplex and *Biecheleriopsis* cf. *adriatica* (Dinophyceae) from the China Sea. Nova Hedwigia. DOI: 101:251-268. 10.1127/nova_hedwigia/2015/0268

Luo, Z., Mertens, K.N., Bagheri, S., Aydin, H., Takano, Y., Matsuoka, K., McCarthy, F.M.G. & Gu, H. (2016a). Cyst-theca relationship and phylogenetic positions of *Scrippsiella plana* sp. nov. and *S. spinifera* (Peridiniales, Dinophyceae). Europ. J. Phycol. 51(2):188-202. DOI: 10.1080/09670262.2015.1120348

Luo, Z.H., You, X.J., Mertens, K.N. & Gu H.F. (2016b). Morphological and molecular characterization of *Tovellia* cf. *aveirensis* (Dinophyceae) from Jiulong River, China. Nova Hedwigia 103:79-94. DOI:10.1127/nova_hedwigia/2016/0339

Luo, Z., Zhang, H., Krock, B., Lu, S., Yang, W. & Gu, H. (2017a). Morphology, molecular phylogeny and okadaic acid production of epibenthic *Prorocentrum* (Dinophyceae) species from the northern South China Sea. Algal Res. 22:14–30. DOI: 10.1016/j.algal.2016.11.020

Luo, Z., Krock, B., Mertens, K.N., Nézan, E., Chomérat, N., Bilien, G., Tillmann, U. & Gu, H. (2017b). Adding new pieces to the *Azadinium* (Dinophyceae) diversity and biogeography puzzle: non-toxigenic *Azadinium zhuanum* sp. nov. from China, toxigenic *A. poporum* from the Mediterranean, and a non-toxigenic *A. dalianense* from the French Atlantic. Harmful Algae 66:65–78. https://doi.org/10.1016/j.hal.2017.05.001.

Luo, Z., Lim, Z.F., Mertens, K.N., Gurdebeke, P., Bogus, K., Carbonell-Moore, M.C., Vrielinck, H., Leaw, C.P., Lim, P.T., Chomérat, N., Lia, X. & Gu, H. (2018a). Morpho-molecular diversity and phylogeny of *Bysmatrum* (Dinophyceae) from the South China Sea and France. Europ. J. Phycol. 53 (3):318-335. https://doi.org/10.1080/09670262.2018.1449014

Luo, z., Hu, Y., Tang, Y., Mertens, K.N., Leaw, C.P., Lim, P.T., Teng, S.T., Wang, L. & Gu, H. (2018b). Morphology, ultrastructure, and molecular phylogeny of *Wangodinium sinense* gen. et sp. nov. (Gymnodinales, Dinophyceae) and revisiting of *Gymnodinium dorsalisulcum* and *Gymnodinium impudicum*. J. Phycol. 54(5):744-761. https://doi.org/10.1111/jpy.12780

Luo, Z., Wang, L., Chan, L., Lu, S. & Gu, H. (2018c). *Karlodinium zhouanum*, a new dinoflagellate species from China, and molecular phylogeny of *Karenia digitata* and *Karenia longicanalis* (Gymnodiniales, Dinophyceae). Phycologia 57:401– 12. https://doi.org/10.2216/17-106.1

Luo, Z., Mertens, K.N., Nézan, E., Gu, L. Pospelova, V., Thoha, H. & Gu, H. (2019). Morphology, ultrastructure and molecular phylogeny of cyst producing *Caladoa arcachonensis* gen. et sp. nov. (Peridiniales, Dinophyceae) from France and Indonesia. Europ. J. Phycol. 54:235-248. DOI: 10.1080/09670262.2018.1558287

Luo, Z., Lim, Z.F., Mertens, K.N., Krock, B., Teng, S.T., Tan, T.H., Leaw, C.P., Lim, P.T. & Gu, H. (2020a). Attributing *Ceratocorys*, *Pentaplacodinium* and *Protoceratium* to Protoceratiaceae (Dinophyceae), with descriptions of *Ceratocorys malayensis* sp. nov . and *Pentaplacodinium usupianum* sp. nov. Phycologia, 59)1):6-23. DOI: 10.1080/00318884.2019.1663693

Luo, Z., Mertens, K.N., Gu, H., Wang. N., Wu, Y., Uttayarnmanee, P., Pransilpa, M. & Roeroe, K.A. (2020b). Morphology, ultrastructure and molecular phylogeny of *Johsia chumphonensis* gen. et sp. nov. and *Parvodinium parvulum* comb. nov. (Peridiniopsidaceae, Dinophyceae). Europ. J. Phycol. DOI: 10.1080/09670262.2020.1829078

Macher, J.-N., Prazeres, M., Taudien, S., Jompa, J., Sadekov, A. & Renema, W. (2021). Integrating morphology and metagenomics to understand taxonomic variability of Amphisorus (Foraminifera, Miliolida) from Western Australia and Indonesia. PLoS ONE 16(1):e0244616. https://doi-org.libproxy.lib.unc.edu/10.1371/journal.pone.0244616

Mardones, J.I., Norambuen, L., Paredes, J., Fuenzalida, G., Dorantes-Aranda, J.J., Chang, K.J.L., Guzmán L., Krock, B. & Hallegraeff, G. (2020). Unraveling the *Karenia selliformis* complex with the description of a non-gymnodimine producing Patagonian phylotype. Harmful Algae 98, 101892. https://doi.org/10.1016/j.hal.2020.101892

Mason, P.L., Litaker, R.W., Jeong, H.J., Ha, J.H., Reece, K.S., Vogelbein, W.K., Stokes, N.A., Park, J.Y., Steidinger, K.A., Vandersea, M.W., Kibler, S. & Tester, P. A. (2007). Description of a new genus of Pfiiesteria-like dinoflagellate, *Luciella* gen. nov. (Dinophyceae), including two new species: *Luciella masanensis* sp. nov. and *Luciella atlantis* sp. nov. J. Phycol. 43:799-810. https://doi-org.libproxy.lib.unc.edu/10.1111/j.1529-8817.2007.00370.x

Masseret, E., Grzebyk, D., Nagai, S., Genovesi, B., Lasserre, B., Laabir, M., Collos, Y., Vaquer, A., & Berrebi, P. (2009). Unexpected genetic diversity among and within populations of the toxic dinoflagellate *Alexandrium catenella* as revealed by nuclear microsatellite markers. Appl. Environ. Microbiol. 75:2037–2045. 10.1128/AEM.01686-08

McCarthy, F.M.G., Gu, H.F., Mertens, K.N., Carbonell-Moore, C., Krueger, A.M., Takano, Y. & Matsuoka, K. (2018). Transferring the freshwater dinoflagellate *Peridinium wisconsinense* (Dinophyceae) to the family Thoracosphaeraceae, with the description of *Fusiperidinium* gen. nov. Phycol. Res. 66(2):137-148. https://doi.org/10.1111/pre.12215

McNally, K.L., Govind, N.S., Thome, P.E. & Trench, R.K. (1994). Small-subunit ribosomal DNA sequence analyses and a reconstruction oe the inferred phylogeny among symbiotic dinoelagellates (Pyrrophyta). J. Phycol. 30:316-329. https://doi.org/10.1111/j.0022-3646.1994.00316.x

Medlin, L.K., Lange M., Wellbrock U., Donner G., Elbrachter M., Hummert C. & Luckas B. (1998). Sequence comparisons link toxic European isolates of *Alexandrium tamarense* from the Orkney isolates to toxic North American stocks. Eur. J. Protistol. 34:329-335. https://doi.org/10.1016/S0932-4739(98)80060-6

Menezes M, Branco S, Miotto MC & Alves-de-Souza, C. (2018). The genus *Alexandrium* (Dinophyceae, Dinophyta) in Brazilian coastal waters. Front. Mar. Sci. 5:421. DOI: 10.3389/fmars.2018.00421

Mertens, K.N., Yamaguchi, A., Kawami, H., Ribeiro, S., Leander, B.S., Price, A.M., Pospelova, V., Ellegaard, M. & Matsuoka, K. (2012). *Archaeperidinium saanichi* sp. nov.: a new species based on morphological variation of cyst and theca within the *Archaeperidinium minutum* Jörgensen 1912 species complex. Mar. Micropaleontol. 96–97:48–62. https://doi.org/10.1016/j.marmicro.2012.08.002

Mertens, K.N., Yamaguchi, A., Takano, Y., Pospelova, V., Head, M.J., Radi, T., Pienkowski, A.J., de Vernal , A., Kawami, H. & Matsuoka, K. (2013). A new heterotrophic dinoflagellate from the north-eastern Pacific, *Protoperidinium fukuyoi*: cyst-theca relationship, phylogeny, distribution and ecology. J. Eukaryot. Microbiol. 60(6):545–563. DOI: 10.1111/jeu.12058

Mertens, K.N., Takano, Y., Gu, H., Yamaguchi, A., Pospelova, V., Ellegaard, M. & Matsuoka, K. (2015a). Cyst-theca relationship of a new dinoflagellate with a spiny round brown cyst, *Protoperidinium lewisiae* sp. nov., and its comparison to the cyst of *Oblea acanthocysta*. Phycol. Res. 63:110–124. DOI: 10.1111/pre.12083

Mertens, K.N., Takano, Y., Yamaguchi, A., Gu, H., Bogus, K., Kremp, A., Bagheri, S., Matishov, G. & Matsuoka, K. (2015b). The molecular characterization of the enigmatic dinoflagellate *Kolkwitziella acuta* reveals an affinity to the *Excentrica* section of the genus *Protoperidinium*, Systematics and Biodiversity 2015, 1-6. http://dx.doi.org/10.1080/14772000.2015.1078855.

Mertens, K. N., Wolny, J., Carbonell-Moore, C., Bogus, K., Ellegaard, M., Limoges, A., de Vernal, A., Gurdebeke, P., Omura, T., Al-Muftah, A. & Matsuoka, K. (2015c). Taxonomic re-examination of the toxic armored dinoflagellate *Pyrodinium bahamense* Plate 1906: can morphology or LSU sequencing separate *P. bahamense* var. *compressum* from var. *bahamense*? Harmful Algae 41:1–24. DOI: 10.1016/j.hal.2014.09.010

Mertens, K.N., Gu, H., Takano, Y., Price, A., Pospelova, V., Bogus, K., Versteegh, G, Marret, F., Turner, R.E., Rabalais, N. & Matsuoka, K. (2017a). Cyst-theca relationship and phylogenetic position of *Impagidinium caspienense* incubated from Caspian sea surface sediments: relation to *Gonyaulax baltica* and evidence for heterospory within Gonyaulacoid dinoflagellates. J. Eukaryot. Microbiol. 64(6):829-842. doi:10.1111/jeu.12417.

Mertens, K.N., Gu, H., Takano, Y., Price, A., Pospelova, V., Bogus, K., Versteegh, G, Marret, F., Turner, R.E., Rabalais, N. & Matsuoka, K. (2017b). The cyst-theca relationship of *Trinovantedinium pallidifulvum*, with erection of *Protoperidinium lousianensis* sp. nov. and their phylogenetic position within the Conica group. Palynology 41(2):183-202. https://doi.org/10.1080/01916122.2016.1147219

Mertens, K.N., Carbonell-Moore, M.C., Pospelova, V., Head, M.J., Highfield, A., Schroeder, D., Gu, H., Andree, K.B., Fernández, M., Yamaguchi, A., Takano, Y., Matsuoka, K., Nézan, E., Bilien, G., Okolodkov, Y., Koike, K., Hoppenrath, M., Pfaff, M., Pitcher, G., Al-Muftah, A., Rochon, A., Lim, P.T., Leaw, C.P., Lim, Z.F. & Ellegaard, M. (2018). *Pentaplacodinium saltonense* gen. et sp. nov. (Dinophyceae) and its relationship to the cyst-defined genus *Operculodinium* and yessotoxin-producing *Protoceratium reticulatum*. Harmful Algae 71: 57-77. DOI: 10.1016/j.hal.2017.12.003

Miranda, L. N., Zhuang, Y. & Zhang, H., Lin, S. (2012). "Phylogenetic analysis guided by intragenomic SSU rDNA polymorphism refines classification of "*Alexandrium tamarense*" species complex". Harmful Algae.16: 35–48. DOI:10.1016/j.hal.2012.01.002.

Mizuyama M., Iguchi, A., Iijima, M., Gibu, K. & Reimer, J.D. (2020). Comparison of Symbiodiniaceae diversities in different members of a *Palytho*a species complex (Cnidaria: Anthozoa: Zoantharia)—implications for ecological adaptations to different microhabitats. PeerJ 8:e8449 http://doi.org/10.7717/peerj.8449

Moestrup, Ø., Hansen, G., Daugbjerg, N., Flaim, G. & D'andrea, M. (2007). Studies on woloszynskioid dinoflagellates II: On *Tovellia sanguinea* sp. nov., the dinoflagellate responsible for the reddening of Lake Tovel, N. Italy. Europ. J. Phycol. 41:57-65. https://doi-org.libproxy.lib.unc.edu/10.1080/09670260600556682

Moestrup, Ø., Hansen, G. & Daugbjerg, N. (2008). Studies on woloszynskioid dinoflagellates III: on the ultrastructure and phylogeny of *Borghiella dodgei* gen. et sp. nov., a cold-water species from Lake Tovel, N. Italy, and on *B. tenuissima* comb. nov. (syn. Woloszynskia tenuissima). Phycologia 47(1):54–78. DOI: 10.2216/07-32.1

Moestrup, Ø., Lindberg, K. & Daugbjerg, N. (2009a). Studies on woloszynskioid dinoflagellates IV: the genus *Biecheleria* gen. nov. Phycol. Res. 57(3):203-220. https://doi.org/10.1111/j.1440-1835.2009.00540.x

Moestrup, Ø., Lindberg, K. & Daugbjerg, N. (2009b). Studies on woloszynskioid dinoflagellates V. Ultrastructure of *Biecheleriopsis* gen. nov., with description of *Biecheleriopsis adriatica* sp. nov. Phycol. Res. 57(3):221-237. https://doi.org/10.1111/j.1440-1835.2009.00541.x

Moestrup, Ø., Hakanen, P., Hansen, G., Daugbjerg, N. & Ellegaard, M. (2014). On *Levanderina fissa* gen. & comb. nov. (Dinophyceae) (syn. *Gymnodinium fissum*, *Gyrodinium instriatum*, *Gyr. uncatenum*), a dinoflagellate with a very unusual sulcus. Phycologia, 53:265–292. DOI: 10.2216/13-254.1

Moestrup, Ø., Nicholls, K.H. & Daugberg, N. (2018). Studies on woloszynskioid dinoflagellates IX: ultrastructure, cyst formation and phylogeny of the 'red-snow' alga *Borghiella pascheri* (Suchlandt) Moestrup (= *Glenodinium pascheri*, *Woloszynskia pascheri*, *Gyrodinium nivalis*). Europ. J. Phycol. 53(3):393-409. DOI: 10.1080/09670262.2018.1453091

Mohammad-Noor, N., Moestrup, Ø., Lundholm, N., Fraga, S., Adam, A., Holmes, M.J. & Saleh, E. (2013). Autecology and phylogeny of *Coolia tropicalis* and *Coolia malayensis* (Dinophyceae), with emphasis on taxonomy of *C. tropicalis* based on light microscopy, scanning electron microscopy and LSU rDNA. J Phycol. 49(3):536-45. DOI:10.1111/jpy.12062

Montresor, M., Sgrosso, S., Procaccini, G. & Kooistra, W. (2003). Intraspecific diversity in *Scrippsiella trochoidea* (Dinopbyceae): evidence for cryptic species. Phycologia 42: 56–70. DOI: 10.2216/i0031-8884-42-1-56.1

Moreira-González, A.R., Fernandes, L.F., Uchida, H., Uesugi, A., Suzuki, T., Chomérat, N., Bilien, G., Pereira, T.A. & Mafra, Jr. L.L. (2019). Morphology, growth, toxin production, and toxicity of

cultured marine benthic dinoflagellates from Brazil and Cuba. J. Appl. Phycol. 31, 3699–3719. https://doi.org/10.1007/s10811-019-01855-0

Murray, S., Flo Jorgensen, M., Daugbjerg, N. & Rhodes, L. (2004). *Amphidinium* revisited. II. Resolving species boundaries in the *Amphidinium operculatum* species complex (Dinophyceae), including the descriptions of *Amphidinium trulla* sp. nov. and *Amphidinium gibbosum.* comb. nov. J. Phycol. 40: 366-382. https://doi.org/10.1046/j.1529-8817.2004.03132.x

Murray, S., Jørgensen, M.F., Ho, S.Y.W., Patterson D.J. & Jermiin, L.S. (2005). Improving the analysis of dinoflagellate phylogeny based on rDNA. Protist 156: 269-286. DOI: 10.1016/j.protis.2005.05.003

Murray, S., Hoppenrath, M., Preisfeld, A., Larsen, J., Yoshimatsu, S., Toriumi, S. & Patterson, D. (2006). Phylogenetics of *Rhinodinium broomeense* gen. et sp. nov., a Peridinioid, sand-dwelling dinoflagellate (Dinophyceae) J. Phycol. 42: 934–942. https://doi.org/10.1111/j.1529-8817.2006.00241.x

Murray, S., Nagahama, Y. & Fukuyo, Y. (2007a). Phylogenetic study of benthic, spine-bearing prorocentroids, including *Prorocentrum fukuyoi* sp. nov. Phycol. Res. 55:91-102. https://doi.org/10.1111/j.1440-1835.2007.00452.x

Murray, S., de Salas, M., Luong-Van, J. & Hallegraeff, G. (2007b). Phylogenetic study of *Gymnodinium dorsalisulcum* comb. nov. from tropical Australian coastal waters (Dinophyceae). Phycol. Res. 55(2):176-184. DOI:10.1111/j.1440-1835.2007.00460.x

Murray, S.A., Garby, T., Hoppenrath, M. & Neilan, B.A. (2012). Genetic diversity, morphological uniformity and polyketide production in dinoflagellates (*Amphidinium*, Dinoflagellata). PLoS ONE 7(6):e38253. DOI: 10.1371/journal.pone.0038253

Murray, S.A., Hoppenrath, M., Orr, R.J.S., Bolch, C., John, U., Diwan, R., Yauwenas, R., Harwood, T., De Salas, M., Neilan, B. & Hallegraeff, G. (2014a). *Alexandrium diversaporum* sp. nov., a new non-saxitoxin producing species: Phylogeny, morphology and sxtA genes Harmful Algae 31:54-65. https://doi.org/10.1016/j.hal.2013.09.005

Murray, S., Momigliano, P., Heimann, K. & Blair, D. (2014b). Molecular phylogenetics and morphology of *Gambierdiscus yasumotoi* from tropical eastern Australia. Harmful Algae. 39:242–252. https://doi.org/10.1016/j.hal.2014.08.003

Na, W., Luo, Z., Mertens, K.N., McCarthy, F.M.G., Gu, L. & Gu, H. (2017). Cyst-motile stage relationship and molecular phylogeny of a new freshwater dinoflagellate *Gymnodinium plasticum* from Plastic Lake, Canada. Phycol. Res. 65(4):312-321. http://dx.doi.org/10.1111/pre.12190

Nascimento, S.M., Mendes, M.C.Q., Menezes, M., Rodriguez, F., Alves-de-Souza, C., Branco, S., Riobó, P., Franco, J., Nunes, J.M.C., Huk, M., Morris, S. & Fraga, S. (2017). Morphology and phylogeny of *Prorocentrum caipirignum* sp. nov. (Dinophyceae), a new tropical toxic benthic dinoflagellate. Harmful Algae 70: 73-89. https://doi.org/10.1016/j.hal.2017.11.001

Nascimento, S.M., da Silva, R.A.F., Oliveira, F., Fraga, S., Salgueiro, F. (2019). Morphology and molecular phylogeny of *Coolia tropicalis*, *Coolia malayensis* and a new lineage of the *Coolia canariensis*

species complex (Dinophyceae) isolated from Brazil. Europ. J. Phycol. 54(3):484-496. https://doi.org/10.1080/09670262.2019.1599449

Nascimento, S.M., Neves, R.A.F., De'Carli, G.A.L., Borsato, G.T., da Silva, R.A.F., Melo, G.A., de Morais, A.M., Cockell, T.C., Fraga, S., Menezes-Salgueiro, A.D., Mafra, L.L., Jr., Hess, P. & Salgueiro, F. (2020). *Ostreopsis* cf. *ovata* (Dinophyceae) molecular phylogeny, morphology, and detection of ovatoxins in strains and field samples from Brazil. Toxins 12:70. DOI:10.3390/toxins12020070

Nézan, E. & Chomérat, N. (2011). *Vulcanodinium rugosum* gen. et sp. nov. (Dinophyceae), un nouveau dinoflagellé Marin de la Côte Méditerranéenne Française. Cryptogam. Algol. 32(1):3-18. https://doi.org/10.7872/crya.v32.iss1.2011.003

Nézan, E., Tillmann, U., Bilien, G., Boulben, S., Chèze, K., Zentz, F., Salas, R. & Chomérat, N. (2012). Taxonomic revision of the dinoflagellate *Amphidoma caudata*: transfer to the genus *Azadinium* (Dinophyceae) and proposal of two varieties, based on morphological and molecular phylogenetic analyses. J. Phycol. 48:925–939. https://doi.org/10.1111/j.1529-8817.2012.01159.x

Nézan E., Siano R., Boulben S., Boulben S., Six C., Bilien G., Chèze K., Duval A., Le Panse S., Quéré J. & Chomérat, N. (2014). Genetic diversity of the harmful family Kareniaceae (Gymnodiniales, Dinophyceae) in France, with the description of *Karlodinium gentienii* sp. nov.: a new potentially toxic dinofl agellate. Harmful Algae 40 :75-91. https://doi.org/10.1016/j.hal.2014.10.006

Nicolás Raho, Santiago Fraga, José P. Abad & Marín, I. (2018). *Biecheleria tirezensis* sp. nov. (Dinophyceae, Suessiales), a new halotolerant dinoflagellate species isolated from the athalassohaline Tirez natural pond in Spain. Europ. J. Phycol. 53(1):99-113. https://doi.org/10.1080/09670262.2017.1386328

Nishimura, T., Sato, S., Tawong, W., Sakanari, H., Yamaguchi, H. & Adachi, M. (2014). Morphology of *Gambierdiscus scabrosus* sp. nov. (Gonyaulacales): a new epiphytic toxic dinoflagellate from coastal areas of Japan. J. Phycol. 50(3):506–514. https://doi.org/10.1111/jpy.12175

Nishimura, T, Uchida, H, Noguchi, R, Oikawa, H, Suzuki, T, Funaki, H, Ihara, C, Hagino, K, Arimitsu, S, Tanii, Y, Abe, S, Hashimoto, K, Mimura, K, Tanaka, K, Yanagida, I. & Adachi, M. (2020). Abundance of the benthic dinoflagellate *Prorocentrum* and the diversity, distribution, and diarrhetic shellfish toxin production of *Prorocentrum lima* complex and *P. caipirignum* in Japan. Harmful Algae. 96:101687. doi: 10.1016/j.hal.2019.101687.

Ok, J.H., Jeong, H.J., Yeon Lee, S., Park, S.A. & Noh, J.H. (2020). *Shimiella* gen. nov. and *Shimiella gracilenta* sp. nov. (Dinophyceae, Kareniaceae), a kleptoplastidic dinoflagellate from Korean waters and its survival under starvation. J. Phycol. DOI: 10.1111/jpy.13067-20-047

Ordás, M. C., Fraga, S., Franco, J.M., Ordás, A. & Figueras, A. (2004). Toxin and molecular analysis of Gymnodinium catenatum (Dinophyceae) strains from Galicia (NW Spain) and Andalucia (S Spain). J. Plankton Res. 26:341–349. DOI: 10.1093/plankt/fbh037

Okamoto, N., Horák, A. & Keeling P.J. (2012). Description of two species of early branching dinoflagellates, *Psammosa pacifica* n. g., n. sp. and *P. atlantica* n. sp. PLoS ONE 7(6):e34900. DOI: 10.1371/journal.pone.0034900

Onuma, R. & Horiguchi, T. (2016). Specificity of *Chroomonas* (Cryptophyceae) as a source of kleptochloroplast for *Nusuttodinium aeruginosum* (Dinophyceae). Phycol. Res. 64(1): https://doi-org.libproxy.lib.unc.edu/10.1111/pre.12117

Orr, R.J.S, Stüken, A., Rundberget, T., Eikrem, W. & Jakobsen, K.S. (2011). Improved phylogenetic resolution of toxic and non-toxic *Alexandrium* strains using a concatenated rDNA approach. Harmful Algae 10:676–688. DOI: 10.1016/j.hal.2011.05.003

Pandeirada, M.S., Craveiro, S.C., Daugbjerg, N., Moestrup, Ø. & Calado, A. J. (2014). Studies on woloszynskioid dinoflagellates VI: description of *Tovellia aveirensis* sp. nov. (Dinophyceae), a new species of Tovelliaceae with spiny cysts. Europ. J. Phycol. 49, 230–243. https://doi.org/10.1080/09670262.2014.910610

Pandeirada, M.S., Craveiro, S.C., Daugbjerg, N., Moestrup, Ø. & Calado, A.J. (2017). Studies on woloszynskioid dinoflagellates VIII: Life cycle, resting cyst morphology and phylogeny of *Tovellia rinoi* sp. nov. (Dinophyceae). Phycologia 56(5):533–548. DOI:10.2216/17-5.1.

Pandeirada, M.S., Craveiro, S.C., Daugbjerg, N., Moestrup, Ø., Domingues, P. & Calado, A. J. (2019). Studies on Woloszynskioid dinoflagellates X: Ultrastructure, phylogeny and colour variation in *Tovellia rubescens* n. sp. (Dinophyceae). J. Eukaryot. Microbiol. 66(6):937-953. https://doi-org.libproxy.lib.unc.edu/10.1111/jeu.12745

Pandeirada, M.S., Craveiro, S.C., Daugbjerg, N., Moestrup, Ø. & Calado, A. J. (2021). Fine-structural characterization and phylogeny of *Sphaerodinium* (Suessiales, Dinophyceae), with the description of an unusual type of freshwater dinoflagellate cyst. Eur. J. Protistol. 78: 125770. https://doi.org/10.1016/j.ejop.2021.125770

Park, M.G., Lee, H., Kim, K.Y. & Kim, S. (2011). Feeding behavior, spatial distribution and phylogenetic affinities of the heterotrophic dinoflagellate *Oxyphysis oxytoxoides*. Aquat. Microb. Ecol. 62:279–287. DOI: 10.3354/ame01474

Park, T.-G., de Salas, M., Bolch, C. & Hallegraeff, G. (2007). Development of a real-time pcr probe for quantification of the heterotrophic dinoflagellate *Cryptoperidiniopsis brodyi* (Dinophyceae) in environmental samples. Appli. Environ. Microbiol. 73(8):2552-2560. DOI: 10.1128/AEM.02389-06

Parkinson, J.E., Coffroth, M.A. & LaJeunesse, T.C. (2015). New species of Clade B Symbiodinium (Dinophyceae) from the greater Caribbean belong to different functional guilds: *S. aenigmaticum* sp. nov., *S. antillogorgium* sp. nov., *S. endomadracis* sp. nov., and *S. pseudominutum* sp. nov. J. Phycol. 51:850-858. DOI: 10.1111/jpy.12340

Penna, A., Garces, E., Vila, M., Giacobbe, M.G., Fraga, S., Luglie, A., Bravo, I., Bertozzini, E. & Vernesi, C. (2005a). *Alexandrium catenella* (Dinophyceae), a toxic ribotype expanding in the NW Mediterranean Sea. Mar. Biol. 148:13–23. DOI 10.1007/s00227-005-0067-5

Penna, A., Vila, M., Fraga, S., Giacobbe, M.G., Andreoni, F., Riobó, P., & Vernesi, C. (2005b). Characterization of *Ostreopsis* and *Coolia* (Dinophyceae) isolates in the western Mediterranean Sea based on morphology, toxicity and internal transcribed spacer 5.8S rDNA sequences J. Phycol. 41: 212–225. DOI: 10.1111/j.1529-8817.2005.04011.x

Penna, A., Fraga, S., Masó, M., Giacobbe, M.G., Bravo, I., Garcés, E., Vila, M., Bertozzini, E., Andreoni, F.A, Lugliè, Antonella & Vernesi, Cristiano (2008). Phylogenetic relationships among the Mediterranean *Alexandrium* (Dinophyceae) species based on sequences of 5.8S gene and Internal Transcript Spacers of the rRNA operon. Europ. J. Phycol. 43(2):163-178. DOI: 10.1080/09670260701783730

Penna, A., Fraga, S., Battocchi, C., Casabianca, S., Giacobbe, M.G., Riobo, P. & Vernesi, C. (2010). A phylogeographical study of the toxic benthic dinoflagellate genus *Ostreopsis* Schmidt. J. Biogeogr. 37:830–841. DOI: 10.1111/j.1365-2699.2009.02265.x

Percopo, I., Siano, R., Rossi, R, Soprano., V., Sarno, D. & Zingone, A. (2013). A new potentially toxic *Azadinium* species (Dinophyceae) from the Mediterranean Sea. *A. dexteroporum* sp. nov. J. Phycol. 49:950–966. https://doi.org/10.1111/jpy.12104

Pienaar, R.N., Sakai ,H. & Horiguchi, T. (2007). Description of a new dinoflagellate with a diatom endosymbiont, *Durinskia capensis* sp. nov. (Peridiniales, Dinophyceae) from South Africa. J. Plant. Res. 120: 247-225. DOI: 10.1007/s10265-006-0047-y

Pinto, S.K., Terada, R. & Horiguchi, T. (2017). *Testudodinium magnum* sp. nov. (Dinophyceae), a novel marine sand-dwelling dinoflagellate from subtropical Japan. Phycologia 56(2):136-146. https://doi.org/10.2216/16-59.1

Pochon, X. & LaJeunesse, T.C. (2021). *Miliolidium* n. gen, a new Symbiodiniacean genus whose members associate with Soritid foraminifera or are free-living. J. Eukaryot. Microbiol. e12856. https://doi.org/10.1111/jeu.12856

Potvin, E., Rochon, A. & Lovejoy, C. (2013). Cyst-theca relationship of the arctic dinoflagellate cyst *Islandinium minutum* (dinophyceae) and phylogenetic position based on SSU rDNA AND LSU rDNA. J. Phycol. 49(5):848-866. DOI: 10.1111/jpy.12089

Potvin, É., Jeong, H.J., Kang, N.S., Noh, J.H. & Yang, E.J. (2015). Morphology, molecular phylogeny, and pigment characterization of a novel phenotype of the dinoflagellate genus *Pelagodinium* from Korean waters. Algae 30(3):183-195. http://dx.doi.org/10.4490/algae.2015.30.3.183

Potvin, É., Kim, S.-Y., Yang, E.J., Head, M.J., Kim, H.-C., Nam, S.-I., Yim, J.H. & Kang, S.-H. (2018). *Islandinium minutum* subsp. *barbatum* subsp. nov. (Dinoflagellata), a new organic-walled dinoflagellate cyst from the Western Arctic: Morphology, phylogenetic position based on SSU rDNA and LSU rDNA, and distribution. Eukary. Microbiol. 65(6):750-772. DOI: 10.1111/jeu.12518.

Prabowo, D.A., Hiraishi, O. & Suda, S. (2013). Diversity of *Crypthecodinium* spp. (Dinophyceae) From Okinawa Prefecture, Japan. L. Mar. Sci. Technol. 21(Suppl.):181-191. DOI: 10.6119/JMST-013-1220-8

Prabowo, D.A., Shah, Md.M., Horiguchi, T. & Suda, S. (2016).. 46:197-209. https://doi.org/10.1007/s12526-015-0351-7

Probert, I., Siano, R., Poirier, C., Decelle, J., Biard, T., Tuji, A., Suzuki, N., & Not, F. (2014). *Brandtodinium* gen. nov. and *B. nutricula* comb. Nov. (Dinophyceae), a dinoflagellate commonly found in

symbiosis with polycystine radiolarians. J. Phycol. 50(2):388-399. https://doi.org/10.1111/jpy.12174

Qin, Z., Yu, K., Chen, B., Wang, Y., Liang, J., Luo, W., Xu, L. & Huang, X. (2019). Diversity of Symbiodiniaceae in 15 coral species from the southern South China Sea: potential relationship with coral thermal adaptability. Front. Microbiol. 10:2343. doi:10.3389/fmicb.2019.02343

Qiu, D., Huang, L., Liu, S & Lin, S. (2011). Nuclear, mitochondrial and plastid gene phylogenies of *Dinophysis miles* (Dinophyceae): Evidence of variable types of chloroplasts. PLoS ONE 6(12):e29398. DOI:10.1371/journal.pone.0029398

Qiu, D., Huang, L., Liu, S., Zhang, H. & Lin S. (2013) Apical groove type and molecular phylogeny suggests reclassification of *Cochlodinium geminatum* as *Polykrikos geminatum*. PLoS ONE 8(8):e71346. DOI: 10.1371/journal.pone.0071346

Raho, N., Pizarro, G., Escalera, L., Reguera, B. & Marin, I. (2008). Morphology, toxin composition and molecular analysis of *Dinophysis ovum* Schutt, a dinoflagellate of the ''*Dinophysis acuminata* complex''. Harmful Algae 7:839–848. https://doi.org/10.1016/j.hal.2008.04.006

Raho, N., Fraga, S., Abad, J.P. & Marín, I. (2018). *Biecheleria tirezensis* sp. nov. (Dinophyceae, Suessiales), a new halotolerant dinoflagellate species isolated from the athalassohaline Tirez natural pond in Spain. Europ. J. Phycol. 53(1):99-113. DOI:10.1080/09670262.2017.1386328

Ramos, V., Salvi, D., Machado, J.P., Vale, M., Azevedo, J. & Vasconcelos, V. (2015). Culture-independent study of the late-stage of a bloom of the toxic dinoflagellate *Ostreopsis* cf. *ovata*: Preliminary findings suggest genetic differences at the sub-species level and allow ITS2 structure characterization. Toxins 7:2514-2533. DOI: 10.3390/toxins7072514

Ramsby, B.D., Hill, M.S., Thornhill, D.J., Steenhuizen, S.F., Achlatis, M., Lewis, A.M., & LaJeunesse, T.C. (2017). Sibling species of mutualistic S*ymbiodinium* Clade G from bioeroding sponges in the western Pacific and western Atlantic oceans. J. Phycol. 53:951-960. https://doi.org/10.1111/jpy.12576

Rehnstam-Holm A.-S., Godhe, A. & Anderson, D.M. (2002). Molecular studies of *Dinophysis* (Dinophyceae) species from Sweden and North America. Phycologia 41:348-357. https://doi.org/10.2216/i0031-8884-41-4-348.1

Reñé, A., Satta, C.T., Garcés, E., Massana, R., Zapata, M., Anglès, S. & Camp, J. (2011) *Gymnodinium litoralis* sp. nov. (Dinophyceae), a newly identified bloom-forming dinoflagellate from the NW Mediterranean Sea. Harmful Algae 12:11-25. https://doi.org/10.1016/j.hal.2011.08.008

Reñé, A., de Salas, M., Camp, J., Balagué , V.& Garcés, E. (2013). A new clade, based on partial LSU rDNA sequences, of unarmoured dinoflagellates. Protist 164(5):673-685. https://doi.org/10.1016/j.protis.2013.07.002

Reñé A., Camp, J. & Garcés, E. (2014). *Polykrikos tanit* sp. nov., a new mixotrophic unarmoured pseudocolonial dinoflagellate from the NW Mediterranean Sea. Protist 165(1) 81-92. https://doi.org/10.1016/j.protis.2013.12.001

Reñé, A., Camp, J. & Garcés, E. (2015). Diversity and phylogeny of Gymnodiniales (Dinophyceae) from the NW Mediterranean Sea revealed by a morphological and molecular approach. Protist 166(2): 234-263. DOI: 10.1016/j.protis.2015.03.001

Reñé A. & Hoppenrath, M. (2019). *Psammodinium inclinatum* gen. nov et comb. nov. (= *Thecadinium inclinatum* Balech) is the closest relative to the toxic dinoflagellate genera *Gambierdiscus* and *Fukuyoa*. Harmful Algae 84:161-171. DOI: 10.1016/j.hal.2019.04.001

Reñé, A., Satta, C.T., López-García, P. & Hoppenrath, M. (2019). Re-evaluation of *Amphidiniopsis* (Dinophyceae) morphogroups based on phylogenetic relationships, and description of three new sand-dwelling species from the NW Mediterranean. J. Phycol. 56(1):68-84. DOI: 10.1111/jpy.12938

Ribeiro, S., Lundholm, N., Amorim, A. & Ellegaard, M. (2010). *Protoperidinium minutum* (Dinophyceae) from Portugal: cyst–theca relationship and phylogenetic position on the basis of single-cell SSU and LSU rDNA sequencing. Phycologia 49:48-63. DOI: 10.2216/09-11.1

Ribeiro, C.G., Santos, A.L. dos, Gourvil, P., Le Gall, F., Marie, D., Tragin, M., Probert, I. & Vaulot, D. (2019). Culturable diversity of arctic phytoplankton during pack ice melting. Elem. Sci. Anth. 8:6. https://doi.org/10.1101/642264

Rhodes, L., Smith, K., Papiol, G.G., Adamson, J., Harwood, T. & Munday, R. (2014). Epiphytic dinoflagellates in sub-tropical New Zealand, in particular the genus *Coolia* Meunier. Harmful Algae 34:36-41. http://dx.doi.org/10.1016/j.hal.2014.02.004

Rhodes, L., Smith, K.F., Verma, A., Curley B.G., Harwood., D.T., Murray, S., Kohli, G.S., Solomona, D., Rongo, T., Munday, R. & Murray, S.A. (2017a). A new species of *Gambierdiscus* (Dinophyceae) from the south-west Pacific: *Gambierdiscus honu* sp. nov. Harmful Algae. 65:61-70. http://dx.doi.org/10.1016/j.hal.2017.04.010

Rodrıguez, F., Escalera, L., Reguera, B., Rial, P., Riobo, P. & Silva, T.J. (2012). Morphological variability, toxicology and genetics of the dinoflagellate *Dinophysis tripos* (Dinophysiaceae, Dinophysiales). Harmful Algae 13:26–33. https://doi.org/10.1016/j.hal.2011.09.012

Rodríguez, F., Fraga, S., Ramilo, I., Rial, P., Figueroa, R.I., Riobó, P. & Bravo, I. (2017). Canary Islands (NE Atlantic) as a biodiversity ‘hotspot’of *Gambierdiscus*: implications for future trends of ciguatera in the area. Harmful Algae. 67:131–43. DOI: 10.1016/j.hal.2017.06.009

Rodríguez, L., López, C., Casado-Amezua, P., Ruiz-Ramos, D.V., Martínez, B., Banaszak, A., Tuya F., García-Fernández, A. & Hernández, M. (2019). Genetic relationships of the hydrocoral *Millepora alcicornis* and its symbionts within and between locations across the Atlantic. Coral Reefs 38:255–268. https://doi.org/10.1007/s00338-019-01772-1

Rogers, J.E., Leblond, J.D. & Moncreiff, C.A. (2006). Phylogenetic relationship of *Alexandrium monilatum* (Dinophyceae) to other *Alexandrium* species based on 18S ribosomal RNA gene sequences. Harmful Algae 5:275–280. https://doi.org/10.1016/j.hal.2005.08.005

Romeikat, C., Knechtel, J. & Gottschling, M. (2019). Clarifying the taxonomy of *Gymnodinium fuscum* var. *rubrum* from Bavaria (Germany) and placing it in a molecular phylogeny of the Gymnodiniaceae (Dinophyceae). Syst. Biodiver. 18(2):102-115. https://doi.org/10.1080/14772000.2019.1699197

Saburova, M. & Chomérat, N. (2014). *Ailadinium reticulatum* gen. et sp. nov. (Dinophyceae), a new thecate, marine, sand-dwelling dinoflagellate from the northern Red Sea. J. Phycol. 50:1120–1136. DOI: 10.1111/jpy.12244

Saburova, M. & Chomérat, N. (2019). *Laciniporus arabicus* gen. et sp. nov. (Dinophyceae, Peridiniales), a new thecate, marine, sand-dwelling dinoflagellate from the northern Indian Ocean (Arabian Sea). J. Phycol. 55(1):84-103. https://doi.org/10.1111/jpy.12783

Saburova, M., Chomérat, N. & Hoppenrath, M. (2012). Morphology and SSU rDNA phylogeny of *Durinskia agilis* (Kofoid & Swezy) comb. nov. (Peridiniales, Dinophyceae), a thecate, marine, sand-dwelling dinoflagellate formerly classified within Gymnodinium. Phycologia 51:287–302. DOI: 10.2216/10-22.1

Sadler, L.A., McNally, K.L., Govind, N.S., Brunk, C.E. & Trench R.K. (1992). The nucleotide sequence of the small subunit ribosomal RNA gene from *Symbiodinium pilosum*, a symbiotic dinoflagellate. Curr. Gene. 21:409-416. DOI: 10.1007/BF00351703

Saito, K., T. Drgon, J. A. F. Robledo, D. N. Krupatkina, & G. R. Vasta. (2002). Characterization of the rRNA locus of *Pfiesteria piscicida* and development of standard and quantitative PCR-based detection assays targeted to the nontranscribed spacer. Appl. Environ. Microbiol. 68:5394-5407. DOI: 10.1128/AEM.68.11.5394–5407.2002

Salas, R., Tillmann, U. & Kavanagh, S. (2014). Morphology and molecular characterization of the small armoured dinoflagellate *Heterocapsa minima* (Peridiniales, Dinophyceae). Europ. J. Phycol. 49:413–428. DOI: 10.1080/09670262.2014.956800

Salgado, P., Fraga, S., Rodríguez, F. & Bravo, I. (2018). Benthic flattened cells of the phylogenetically related marine dinoflagellates *Protoceratium reticulatum* and *Ceratocorys mariaovidiorum* (Gonyaulacales): a new type of cyst? J. Phycol. 54:138–149. DOI: 10.1111/jpy.12611

Saldarriaga, J.F., Leander, B.S., Taylor, F.J., & Keeling, P.J. (2003). *Lessardia elongata* gen. et sp. nov. (Dinoflagellata, Peridiniales, Podolampaceae) and the taxonomic position of the genus *Roscoffia* J. Phycol. 39:368–378. https://doi.org/10.1046/j.1529-8817.2003.02113.x

Salomon, P.S., Janson, S. & Granéli, E. (2003). Multiple species of the dinophagous dinoflagellate genus *Amoebophrya* infect the same host species. Environ. Microbiol. 5 (11):1046–1052. DOI: 10.1046/j.1462-2920.2003.00511.x

Sampayo, E.M., Dove, S. & Lajeunesse, T.C. (2009). Cohesive molecular genetic data delineate species diversity in the dinoflagellate genus *Symbiodinium*. Mol. Ecol. 18:500-519. DOI: 10.1111/j.1365-294X.2008.04037.x

Sampedro, N., Fraga, S., Penna, A., Casabianca, S., Zapata, M., Grünewald, C.F., Riobó, P. & Camp, J. (2011). *Barrufeta bravensis* gen. nov. sp. nov. (Dinophyceae): a new bloom-forming species from

the northwest Mediterranean Sea. J. Phycol. 47:375–392. DOI: 10.1111/j.1529-8817.2011.00968.x

Sampedro, N., Reñé, A., Matos, J., Fortuño, J.-M. & Garcés, E. (2021). Detection of the widespread presence of the genus *Ansanella* along the Catalan coast (NW Mediterranean Sea) and the description of *Ansanella catalana* sp. nov. (Dinophyceae). Europ. J. Phycol. DOI: 10.1080/09670262.2021.1914861

Sarai, C., Yamaguchi, A., Kawami, H. & Matsuoka, K. (2013). Two new species formally attributed to *Protoperidinium oblongum* (Aurivillius) Park et Dodge (Peridiniales, Dinophyceae): evidence from cyst incubation experiments. Rev. Palaeobot. Palynol. 192:103-118. https://doi.org/10.1016/j.revpalbo.2012.12.007

Sato, S., Nishimura, T., Uehara, K., Sakanari, H., Tawong, W., Hariganeya, N., Smith, K., Rhodes, L., Yasumoto, T., Taira, Y., Suda, S., Yamaguchi, H. & Adachi, M. (2011). Phylogeography of *Ostreopsis* along west Pacific Coast, with special reference to a novel clade from Japan. PLoS ONE 6(12): e27983. DOI: 10.1371/journal.pone.0027983

Satta, C.T., Pulina, S., Reñé, A., Padedda, B.M., Caddeo, T., Fois N &Lugliè A. (2020). Ecological, morphological and molecular characterization *of Kryptoperidinium* sp. (Dinophyceae) from two Mediterranean coastal shallow lagoons. Harmful Algae 97:101855. DOI: 10.1016/j.hal.2020.101855

Seaborn, D.W., Tengs, T., Cerbin, S., Kokocinski, M. & Marshall, H.G. (2006). A group of dinoflagellates similar to *Pfiesteria* as defined by morphology and genetic analysis. Harmful Algae 5:1–8. DOI:10.1016/j.hal.2005.01.002

Selina, M.S., Efimova, K.V. & Hoppenrath, M. (2019). Redefinition of the genus *Thecadinium* (Dinophyceae) using morphological and molecular data, and description of *Thecadinium pseudokofoidii* sp. nov. Phycologia 58:(1):36-50. DOI: 10.1080/00318884.2018.1517535

Selina, M.S. & Efimova, K.V. (2020). Morphology and phylogeny of *Thecadinium ovatum* (Dinophyceae) from the Pacific coast of Russia. Phycologia 59(6):1-13. DOI:10.1080/00318884.2020.1809194

Shao, P., Chen, Y.-Q., Zhou, H., Yuan, J., Qu, L.-H., Zhao, D. & Lin, Y.-S. (2004). Genetic variability in Gymnodiniaceae ITS regions: implications for species identification and phylogenetic analysis. Mar. Biol. 144:215-224. https://doi.org/10.1007/s00227-003-1157-x

Shi, T., Niu, G., Kvitt, H,. Zheng, X., Qin, Q., Sun, D., Ji, Z. & Tchernov, D. (2020). Untangling ITS2 genotypes of algal symbionts in zooxanthellate corals. Mol. Ecol. Resour. 21(1):137-152. https://doi-org.libproxy.lib.unc.edu/10.1111/1755-0998.13250

Shikata, T., Taniguchi, E., Sakamoto, S., Kitatsuji, S., Yamasaki, Y., Yoshida, M. & Oikawae, H. (2020). Phylogeny, growth and toxicity of the noxious red-tide dinoflagellate *Alexandrium leei* in Japan. Reg. Stud. Mar. Sci. 36:101265. DOI:10.1016/j.rsma.2020.101265

Shin, H.H., Li, Z., Kim, E.S., Youn, J.Y., Jeon, S.G., Oh., S.J. & Lim, W.-A. (2016). Morphology and phylogeny of *Triadinium polyedricum* (Pouchet) Dodge (Dinophyceae) from Korean coastal waters. Ocean Sci. J. 51(4):647-654. DOI: 10.1007/s12601-016-0049-1

Shin, H.H., Li, Z., Lee K.-W. & Matsuoka, K. (2019): Molecular phylogeny and morphology of *Torquentidium* gen. et comb. nov. for *Cochlodinium convolutum* and allied species (Ceratoperidiniaceae, Dinophyceae). 249-262. Europ. J. Phycol. 54:3, 249-262. DOI: 10.1080/09670262.2018.1550213

Shin, H.H., Li, Z. & Matsuoka, K. (2020). Reclassification of *Gyrodinium flavescens* Kofoid & Swezy as *Torquentidium flavescens* comb. nov. (Ceratoperidiniaceae, Dinophyceae), based on morphology and phylogeny. Phycologia 59(2):133-139. DOI: 10.1080/00318884.2019.1709396

Siano, R., Kooistra, W.H.C.F., Montresor, M. & Zingone, A. (2009). Unarmoured and thin-walled dinoflagellates from the Gulf of Naples, with the description of *Woloszynskia cincta* sp. nov. (Dinophyceae, Suessiales). Phycologia 48:44–65. DOI: 10.2216/08-61.1

Siano, P., Montresor, M., Probert, I., Not, F. & de Vargas, C. (2010). *Pelagodinium* gen. nov. and *P. béii* comb. nov., a dinoflagellate symbiont of planktonic Foraminifera. Protist 161:385-399. doi:10.1016/j.protis.2010.01.002

Skovgaard, A., Massana, R., Balagué, V. & Saiz, E. (2005). Phylogenetic position of the copepod-infesting parasite *Syndinium turbo* (Dinoflagellata, Syndinea). Protist 156(4):413-23. DOI: 10.1016/j.protis.2005.08.002

Skovgaard, A., Massana, D.R. & Saiz., E. 2007. Parasitic species of the genus *Blastodinium* (Blastodiniphyceae) are peridinioid dinoflagellates. J. Phycol. 43:553–560. DOI: 10.1111/j.1529-8817.2007.00338.x

Skovgaard, A. & Salomonsen, X.M. (2009). *Blastodinium galatheanum* sp. nov. (Dinophyceae) a parasite of the planktonic copepod Acartianegligens (Crustacea,Calanoida) in the central Atlantic Ocean. Europ. J. Phycol. 44:(3):425-438. DOI: 10.1080/09670260902878743

Skovgaard, A. Karpov, S.A., & Guillou, L. (2012). The parasitic dinoflagellates *Blastodinium* spp. inhabiting the gut of marine, planktonic copepods: morphology, ecology, and unrecognized species diversity. Front. Microbiol. 3:305 https://doi.org/10.3389/fmicb.2012.00305

Small, H.J., Shields, J.D., Reece, K.S., Bateman, K. & Stentiford, G.D. (2011). Morphological and molecular characterization of *Hematodinium perezi* (Dinophyceae: Syndiniales), a dinoflagellate parasite of the harbour crab, *Liocarcinus depurator*. J. Eukaryot. Microbiol. 59(1):54–66. DOI: 10.1111/j.1550-7408.2011.00592.x

Smith, K.F., Rhodes, L., Verma, A., Curley, B.G., Harwood, D.T., Kohli, G.S., Solomon, D., Rongo, T., Munday, R., Murray, S.A. (2016). A new *Gambierdiscus* species (Dinophyceae) from Rarotonga, Cook Islands: *Gambierdiscus cheloniae* sp. nov. Harmful Algae 60:45-56. https://doi.org/10.1016/j.hal.2016.10.006

Smith, K.F., Rhodes, L., Harwood, D.T., Adamson, J., Moisan, C., Munday, R. & Tillmann, U. (2016). Detection of *Azadinium poporum* in New Zealand: the use of molecular tools to assist with species isolations. J. Appl. Phycol., 28:1125–1132. DOI: 10.1007/s10811-015-0667-5

Smith, K.F., Rhodes, L., Verma, A., Curley, B.G., Harwood, D.T., Kohli, G.S., Solomon, D., Rongo, T., Munday, R., Murray, S.A. (2016). A new *Gambierdiscu*s species (Dinophyceae) from Rarotonga,

Cook Islands: *Gambierdiscus cheloniae* sp. nov. Harmful Algae 60:45–56. DOI: 10.1016/j.hal.2016.10.006

Soehner, S., Zinssmeister, C., Kirsch, M. & Gottschling, M. (2012). Who am I - and if so, how many? Species diversity of calcareous dinophytes (Thoracosphaeraceae, Peridiniales) in the Mediterranean Sea. Org. Divers. Evol. 12:339–348. https://doi-org.libproxy.lib.unc.edu/10.1007/s13127-012-0109-z

Sparmann, S.F. & Leander, B. (2008). Comparative morphology and molecular phylogeny of *Apicoporus* n. gen.: a new genus of marine benthic dinoflagellates formerly classified within *Amphidinium*. M. Hoppenrath. Protist 159(3):383-399. DOI:10.1016/j.protis.2007.12.002

Stock, A., Breiner, H.-W., Pachiadaki, M., Edgcomb, V., Filker, S., Cono, V.L., Yakimov, M.M. & Stoeck, T. (2012). Microbial eukaryote life in the new hypersaline deep-sea basin. Extremophiles 16:21–34. DOI:10.1007/s00792-011-0401-4

Strassert, J.F. H., Hehenberger, E., del Campoa, J., Okamotoa, N., Koliskoa, M., Richards, T.A., Worden, A.Z., Santorod, A.E. & Keeling, P.J. (2018). Phylogeny, evidence for a cryptic plastid, and distribution of *Chytriodinium* parasites (Dinophyceae) infecting copepods. J. Eukaryot Microbiol. 66(4):574-581. DOI: 10.1111/jeu.12701

Strychar, K. B., Coates, M., Sammarco, P. W., Piva, T. J. & Scott, P. T. (2005). Loss of *Symbiodinium* from bleached soft corals *Sarcophyton ehrenbergi*, *Sinularia* sp. and *Xenia* sp. J. Exper. Mar. Biol. Ecol. 320:159–177. DOI: 10.1093/icesjms/fsp048

Sunesen, I., Hernández, F.R., Juárez, D.A., Kubis, J.A.T., Lavigne, A.S., Rossignoli, A., Riobó, P. & Sar, E.A. (2020). Morphology, genetics and toxin profile of *Prorocentrum texanum* (Dinophyceae) from Argentinian marine coastal waters. Phycologia 59(6):634-650. DOI: 10.1080/00318884.2020.1830552

Takishita, K., Ishikura, M., Koike, K. & Maruyama, T. (2003). Comparison of phylogenies based on nuclear-encoded SSU rDNA and plastid-encoded psbA in the symbiotic dinoflagellate genus *Symbiodinium*. Phycologia 42:285–291. https://doi.org/10.2216/i0031-8884-42-3-285.1

Takahashi, K., Sarai, C. & Iwataki, M. (2014). Morphology of two marine woloszynskioid dinoflagellates, *Biecheleria brevisulcata* sp. nov. and *Biecheleriopsis adriatica* (suessiaceae, Dinophyceae), from Japanese coasts. Phycologia 53(1):52-65. DOI:10.2216/13-192.1

Takahashi, K., Moestrup Ø., Jordan, R.W. & Iwataki, M. (2015). Two new freshwater Woloszynskioids *Asulcocephalium miricentonis* gen. et sp. nov. and *Leiocephalium pseudosanguineum* gen. et sp. nov. (Suessiaceae, Dinophyceae) lacking an apical furrow apparatus. Protist 166(6):638-58. DOI: 10.1016/j.protis.2015.10.003. Epub 2015 Oct 19. PMID: 26599726.

Takahashi, K., Moestrup, Ø., Wada, M., Ishimatsu, A., Nguyen, V.N., Fukuyo, Y. & Iwataki, M. (2017). *Dactylodinium pterobelotum* gen. et sp. nov., a new marine woloszynskioid dinoflagellate positioned between the two families Borghiellaceae and Suessiaceae. J. Phycol. 53:1223-1240. DOI: 10.1111/jpy.12575

Takahashi K., G. Benico, W.M. Lum & M. Iwataki (2019). *Gertia stigmatica* gen. et sp. nov. (Kareniaceae, Dinophyceae), a new marine unarmored dinoflagellate possessing the peridinin-type chloroplast with an eyespot. Protist 170(5):125680. https://doi.org/10.1016/j.protis.2019.125680

Takahashi, K., Lum, W.M., Benico, G., Uchida; H., Ozawa, M., Oikawa, H., Suzuki, T., Nguyen, N.V., Ha, D.V., Iwataki., M. (2021). Toxigenic strains of *Azadinium poporum* (Amphidomataceae, Dinophyceae) from Japan and Vietnam, with first reports of *A. poporum* (ribotype A) and *A. trinitatum* in Asian Pacific. Phycological Res. 1.147. DOI: 10.1111/pre.12455

Takano, Y. & Horiguchi, T. (2004). Surface ultrastructure and molecular phylogenetics of four unarmored heterotrophic dinoflagellates, including the type species of the genus *Gyrodinium* (Dinophyceae). Phycol. Res. 52:107-116. https://doi.org/10.1111/j.1440-1835.2004.tb00319.x

Takano, Y., Yamaguchi, H., Inouye, I., Moestrup, Ø. & Horiguchi, T. (2014). Phylogeny of five species of *Nusuttodinium* gen. nov. (Dinophyceae), a genus of unarmoured kleptoplastidic dinoflagellates. Protist 165(6):759-778. DOI:10.1016/j.protis.2014.09.001

Tamura, M. & Horiguchi, T. (2005). *Pileidinium ciceropse* gen. et sp. nov. (Dinophyceae), a sand-dwelling dinoflagellate from Palau. Europ. J. Phycol. 40:(3):281-291. DOI: 10.1080/09670260500211677

Tamura, M., Shimada, S., & Horiguchi, T. (2005). *Galeidiniium rugatum* gen. et sp. nov. (Dinophyceae), a new coccoid dinoflagellate with a diatom endosymbiont. J. Phycol. 41:658-671. DOI:10.1080/09670260500211677

Tamura, M., Takano, Y. & Horiguchi, T. (2009). Discovery of a novel type of body scale in the marine dinoflagellate, *Amphidinium cupulatisquama* sp. nov. (Dinophyceae). Phycol. Res. 57:304–312. DOI: 10.1111/j.1440-1835.2009.00550.x

Tang, X., Yu, R., Zhang, Q. Y. Wang , Yan, T. & Zhou, M. (2010). Molecular phylogenetic analysis of dinoflagellate *Scrippsiella trochoidea* isolated from the East Asian waters. Chin. J. Ocean. Limnol. 28:323–328. https://doi.org/10.1007/s00343-010-9288-7

Tang, Y.Z., Harke, M.J. & Gobler, C.J. (2013). Morphology, phylogeny, dynamics, and ichthyotoxicity of *Pheopolykrikos hartmannii* (Dinophyceae) isolates and blooms from New York, USA. J. Phycol. 49(6):1084-1094. https://doi.org/10.1111/jpy.12114

Tawong, W., Nishimura, T., Sakanari, H., Sato, S., Yamaguchi, H. & Adachi, M. (2015). Characterization of *Gambierdiscus* and *Coolia* (Dinophyceae) isolates from Thailand based on morphology and phylogeny. Phycol. Res. 63(2):125-133. https://doi.org/10.1111/pre.12074

Teschima, M. M., Garrido A., Paris, A., Nunes, F.L.D. & Zilberberg, C. (2019) Biogeography of the endosymbiotic dinoflagellates (Symbiodiniaceae) community associated with the brooding coral *Favia gravida* in the Atlantic Ocean. PLOS ONE 14(4): e0215167. https://doi.org/10.1371/journal.pone.0215167

Tibiriçá, C.E., Sibat, M., Fernandes, L., Bilien, G., Chomérat, N., Hess, P. & Mafra, L.L. (2020). Diversity and toxicity of the genus *Coolia* Meunier in Brazil, and detection of 44-methyl gambierone in *Coolia tropicalis*. Toxins, 12, 327. doi:10.3390/toxins12050327

Tillmann, U., Elbrächter, M., Krock, B., John, U. & Cembella, A. (2009). *Azadinium spinosum* gen. et sp. nov. (Dinophyceae) identified as a primary producer of azaspiracid toxins. Europ. J. Phycol. 44:63–79. https://doi.org/10.1080/09670260802578534

Tillmann, U., Elbrächter, M., John, U., Krock, B. & Cembella, A. (2010). *Azadinium obesum* (Dinophyceae), a new nontoxic species in the genus that can produce azaspiracid toxins. Phycologia 49:169–82. DOI: 10.2216/09-35.1

Tillmann, U., Elbrächter, M., John, U& Krock, B. (2011). A new non-toxic species in the dinoflagellate genus *Azadinium*: *A. poporum* sp. nov. Europ. J. Phycol. 46:74–87. https://doi.org/10.1080/09670262.2011.556753

Tillmann, U., Söhner, S., Nézan, E. & Krock, B. (2012). First record of *Azadinium* from the Shetland Islands including the description of *A. polongum* sp. nov. Harmful Algae 20:142–155. https://doi.org/10.1016/j.hal.2012.10.001

Tillmann, U., Gottschling, M., Nézan, E., Krock, B & Bilien, G. (2014). Morphological and molecular characterization of three new *Azadinium* species (Amphidomataceae, Dinophyceae) from the Irminger Sea. Protist 165:417–444. https://doi.org/10.1016/j.protis.2014.04.004

Tillmann, U. & Akselman, R. (2016) Revisiting the 1991 algal bloom in shelf waters off Argentina: *Azadinium luciferelloides* sp. nov. (Amphidomataceae, Dinophyceae) as the causative species in a diverse community of other amphidomataceans. Phycol. Res. 64:160–175. https://doi.org/10.1111/pre.12133

Tillmann, U., Trefault, N., Krock, B., Parada-Pozo, G., De La Iglesia, R., & Vásquez, M. (2017b). Identification of *Azadinium poporum* (Dinophyceae) in the Southeast Pacific: morphology, molecular phylogeny, and azaspiracid profile characterization. J. Plankton Res. 39:350–367. https://doi.org/10.1093/plankt/fbw099

Tillmann, U., Wietkamp S., Krock, B., Tillmann, A., Voss, D. & Gu, H. (2020). Amphidomataceae (Dinophyceae) in the western Greenland area, including description of *Azadinium perforatum* sp. nov. Phycologia 59(1):63-88. DOI:10.1080/00318884.2019.1670013

Verma, A., Hoppenrath, M., Dorantes-Aranda, J.J., Harwood, D. & Murray, S. (2016a). Molecular and phylogenetic characterization of *Ostreopsis* (Dinophyceae) and the description of a new species, *Ostreopsis rhodesae* sp. nov., from a subtropical Australian lagoon. Harmful Algae 60:116-130. DOI: 10.1016/j.hal.2016.11.004

Verma, A., Hoppenrath, M., Harwood, T., Brett, S., Rhodes, L. & Murray, S. (2016b). Molecular phylogeny, morphology and toxigenicity of *Ostreopsis* cf. *siamensis* (Dinophyceae) from temperate south-east Australia. Phycol. Res. 64(3):146-159. https://doi.org/10.1111/pre.12128

Verma, A., Kazandjian, A., Sarowar, C., Harwood, D.T., Murray, J.S., Pargmann, I., Hoppenrath, M. & Murray, S. A. (2019). Morphology and phylogenetics of benthic Prorocentrum species (Dinophyceae) from tropical northwestern Australia. Toxins 11(10):571. https://doi.org/10.3390/toxins11100571

Wakeman, K.C., Yamaguchi, A., Roy, M.C. & Jenke- Kodama, H. (2015). Morphology, phylogeny and novel chemical compounds from *Coolia malayensis* (Dinophyceae) from Okinawa, Japan. Harmful Algae 44:8-19. https://doi.org/10.1016/j.hal.2015.02.009

Wakeman, K.C., Hoppenrath, M., Yamaguchi, A., Gavelis, G.S., Leander, B.S. & Nozaki, H. (2018a). Ultrastructure of the marine benthic dinoflagellate *Plagiodinium belizeanum* (Dinophyceae) from the southeast Pacific island of Okinawa, Japan. Phycologia 57(2):209-222. DOI: 10.2216/17-43.1

Wakeman, K.C. (2018b). Molecular phylogeny and morphology of *Haplozoon ezoense* n. sp. (Dinophyceae): A parasitic dinoflagellate with ultrastructural evidence of remnant non-photosynthetic plastids. Protist 169(3):333-350. DOI: 10.1016/j.protis.2018.04.008

Wang, H., Lu, D., Huang, H., Göbel, J., Dai, X. & Xia, P. (2011). First observation of *Karlodinium veneficum* from the East China Sea and the coastal waters of Germany. Acta Oceanol. Sin. 30(6):112-121. DOI: 10.1007/s13131-011-0168-6

Wang, J., Cen, J., Li, S. Lü, S. Moestrup, Ø., Chan, K.-K., C., Jiang, T., Lei, X. (2018). A re-investigation of the bloom-forming unarmored dinoflagellate *Karenia longicanalis* (syn. *Karenia umbella*) from Chinese coastal waters. J. Ocean. Limnol. 36, 2202–2215 (2018). https://doi.org/10.1007/s00343-019-7191-4

Wang, N., Mertens, K.N., Krock, B., Z., Luo, Derrien, A., Pospelova, V., Liang,Y., Bilien, G., Smith, K.F., De Schepper, S., Wietkamp, S., Tillmann, U. & Gu, H. (2019). Cryptic speciation in *Protoceratium reticulatum* (Dinophyceae): Evidence from morphological, molecular and ecophysiological data. Harmful Algae 88: 101610. https://doi.org/10.1016/j.hal.2019.05.003

Watanabe, K., Miyoshi, Y., Kubo, F., Onuma, R., Murray, S. & Horiguchi, T. (2014). *Ankistrodinium armigerum* sp. nov. (Dinophyceae), a new species of heterotrophic marine sand-dwelling dinoflagellate from Japan and America. Phycol. Res. 62(2):125-135. DOI: 10.1111/pre.12048

Wham, D.C., Ning, G. & LaJeunesse, T.C. (2017). *Symbiodinium glynnii* sp. nov., a species of stress-tolerant symbiotic dinoflagellates from pocilloporid and montiporid corals in the Pacific Ocean. Phycologia 56:396-409. DOI: 10.2216/16-86.1

Wolny, J.L., Egerton, T.A., Handy, S.M., Stutts, W.L., Smith, J.L., Whereat, E.B., Bachvaroff, T.R., Henrichs, D.W., Campbell, L. & Deeds, J.R. (2020). Characterization of *Dinophysis* spp. (Dinophyceae, Dinophysiales) from the mid-Atlantic region of the United States. J. Phycol. 56(2):404-424. https://doi.org/10.1111/jpy.12966

Xia, S., Zhang, Q., Zhu, H., Cheng, Y., Liu, G. & Hu, Z. (2013). Systematics of a kleptoplastidal dinoflagellate, *Gymnodinium eucyaneum* Hu (Dinophyceae), and its cryptomonad endosymbiont. PLoS ONE 8(1):e53820. DOI: 10.1371/journal.pone.0053820.

Yamada, N., Terada, R., Tanaka, A., Horiguchi, T. 2013. *Bispinodinium angelaceum* gen. et sp. nov. (Dinophyceae), a new sand-dwelling dinoflagellate from the seafloor off Mageshima Island, Japan. J. Phycol. 49(3):555-69. DOI: 10.1111/jpy.12064. Epub 2013 May 9. PMID: 27007044

Yamada, N., Tanaka, A. & Horiguchi, T. (2015). Pigment compositions are linked to the habitat types in dinoflagellates. J. Plant. Res. 128:923–932. https://doi.org/10.1007/s10265-015-0745-4

Yamada, N., Sym, S.D. & Horiguchi, T. (2017). Identification of highly divergent diatom-derived chloroplasts in dinoflagellates, Including a description of *Durinskia kwazulunatalensis* sp. nov. (Peridiniales, Dinophyceae). Mol. Biol. Evol. 34(6):1335–1351. DOI: 10.1093/molbev/msx054

Yamada, N., Mahmutjan, D., Ryuta, T. & Horiguchi, T. (2019). *Plagiodinium ballux* sp. nov. (Dinophyceae), a deep (36 m) sand dwelling dinoflagellate from subtropical Japan. Phycol. Res. 67(1):12-20. DOI: 10.1111/pre.12336

Yamada, N., Sakai, H., Onuma, R., Kroth, P.G. & Horoguichi, T. (2020). Five non-motile dinotom dinoflagellates of the genus *Dinothrix*. Front. Plant Sci. 11:591050. https://doi.org/10.3389/fpls.2020.591050

Yamaguchi, A. & Horiguchi, T. (2005). Molecular phylogenetic study of the heterotrophic dinoflagellate genus *Protoperidinium* (Dinophyceae) inferred from small subunit rRNA gene sequences. Phycol. Res. 53:30-42. https://doi.org/10.1111/j.1440-1835.2005.tb00355.x

Yamaguchi, A., Kawamura, H. & Horiguchi, T. (2006). A further phylogenetic study of the heterotrophic dinoflagellate genus, *Protoperidinium* (Dinophyceae) based on small and large subunit ribosomal RNA gene sequences. Phycol. Res. 54:317-329. https://doi.org/10.1111/j.1440-1835.2006.00438.x

Yamaguchi, A., Hoppenrath, M., Pospelova, V., Horiguchi, T. & Leander, B.S. (2011). Molecular phylogeny of the marine sand-dwelling dinoflagellate *Herdmania litoralis* and an emended description of the closely related planktonic genus *Archaeperidinium* Jörgensen. Europ. J. Phycol. 46:98–112. DOI: 10.1080/09670262.2011.564517

Yamaguchi, A., Yoshimatsu, S., Hoppenrath, M., Wakemand, K. C. & Kawai, H. (2016). Molecular phylogeny of the benthic dinoflagellate genus *Amphidiniopsis* and its relationships with the Family Protoperidiniaceae. Protist 167(6):568–83. https://doi.org/10.1016/j.protis.2016.09.003

Yamaguchi, A., Wakeman, K. C., Hoppenrath, M., Horiguchi, T. & Kawai, H. (2018). Molecular phylogeny of the benthic dinoflagellate *Cabra matta* (Dinophyceae) from Okinawa, Japan. Phycologia 57(6):630-640. DOI: http://dx.doi.org/10.2216/18-7.1

Yamamoto, M., Wakeman, K.C., Tomioka, S. & Horiguchi, T. (2020). Molecular phylogeny and ultrastructure of two novel parasitic dinoflagellates, *Haplozoon hracile* sp. nov. and *H. pugnus* sp. nov. Phycologia 59(4):305-319. DOI: 10.1080/00318884.2020.1753427

Yang, Z.B., Hodgkiss, I.J. & Hansen, G. (2001). *Karenia longicanalis* sp. nov. (Dinophyceae): a new bloom-forming species isolated from Hong Kong, May 1998. Bot. Mar. 44: 67-74. DOI: 10.1515/BOT.2001.009

Yokouchi, K. & Horiguchi, T. (2021). *Paragymnodinium verecundum* sp. nov. (Gymnodiniales, Dinophyceae), a new species of mixotrophic dinoflagellate from Japan. Phycol. Res. 69(2):124-136. https://doi.org/10.1111/pre.12452

Yokouchi, K., Onuma, R. & Horiguchi, T. (2018). Ultrastructure and phylogeny of a new species of mixotrophic dinoflagellate, *Paragymnodinium stigmaticum* sp. nov. (Gymnodiniales, Dinophyceae). J. Phycol. 57(5): 539-554.

Yoon, Kang, E.Y., N.S. & Jeong, H.J. (2012). *Gyrodinium moestrupii* n. sp., a new planktonic heterotrophic dinoflagellate from the coastal waters of western Korea: morphology and ribosomal DNA gene sequence. J. Eukaryot. Microbiol. 59(6):571-86. DOI: 10.1111/j.1550-7408.2012.00632.x.om

Yoshida, T., Nakai, R., Seto, H., Wang, M. K., Iwataki, M. & Hiroishi, S. (2003). Sequence analysis of 5.8S rDNA and the internal transcribed spacer region in dinoflagellate Heterocapsa species (Dinophyceae) and development of selective PCR primers for the bivalve killer *Heterocapsa circularisquama*. Microbes Environ. 18:216–22. DOI: 10.1264/jsme2.18.216

You, X., Luo, Z., Su, Y., Gu, L. & Gu, H. (2015). *Peridiniopsis jiulongensis*, a new freshwater dinoflagellate with a diatom endosymbiont from China. Nova Hedwigia 101:313–326. DOI: 10.1127/nova_hedwigia/2015/0272

Yuasa, T., Horiguchi, T., Mayama, S. & Takahashi, O. (2016). *Gymnoxanthella radiolariae* gen. et sp. nov. (Dinophyceae), a dinoflagellate symbiont from solitary polycystine radiolarians. J. Phycol. 52:89–104. DOI: 10.1111/jpy.12371

Zardoya, R., Costas, E., López-Rodas, V., Garrido-Pertierra, A. & Bautista, J. M. (1995). Revised dinoflagellate phylogeny inferred from molecular analysis of large-subunit ribosomal RNA gene sequences. J. Mol. Evol. 41:637-645. https://doi.org/10.1007/BF00175822

Zeng, N., Gu, H., Smith, K.F., Rhodes, L.L., Selwood, A.I. & Yang, W. 2012. The first report of *Vulcanodinium* r*ugosum* (Dinophyceae) from the South China Sea with a focus on the life cycle, New Zeal. J. Mar. Fresh. Res. 46:4:511-521. http://dx.doi.org/10.1080/00288330.2012.719911

Žerdoner Čalasan, A., Kretschmann, J. & Gottschling, M. (2019). They are young, and they are many: dating freshwater lineages in unicellular dinophytes. Environ. Microbiol. 21(11):4125-4135. DOI: 10.1111/1462-2920.14766

Zhang, H., Li, Y., Cen, J., Wang, H., Cui, L., Dong, Y., Lu, S. (2015). Morphotypes of *Prorocentrum* lima (Dinophyceae) from Hainan Island, South China Sea: morphological and molecular characterization. Phycologia 54(5):503-516, DOI: 10.2216/15-8.1

Zhang, H., Lu, S., Li, Y., Cen, J., Wang, H., Li, Q. & Nie, X. (2017). Morphology and molecular phylogeny of *Ostreopsis* cf. *ovata* and *O*. *lenticularis* (Dinophyceae) from Hainan Island, South China Sea. Phycol. Res. 66(1):3-14. DOI: 10.1111/pre.12192

Zhang, H., Lü, S., Cen, J., Li, Y., Li, Q. & Wu, Z. (2020). Morphology and molecular phylogeny of three species of *Coolia* (Dinophyceae) from Hainan Island, South China Sea. J. Ocean. Limnol. https://doi.org/10.1007/s00343-020-9326-z

Zhang, Q., Liu, G.-X., Hu, Z.-Y. (2011a). *Durinskia baltica* (Dinophyceae), a newly recorded species and genus from China, and its systematics. J. Syst. Evol. 49(5): 476-485. https://doi.org/10.1111/j.1759-6831.2011.00153.x

Zhang, Q., Liu, G.-X., Hu, Z.-Y. (2011b). Morphological differences and molecular phylogeny of freshwater blooming species, *Peridiniopsis* spp. (Dinophyceae) from China. Eur. J. Protistol. 47(3):149-160. https://doi.org/10.1016/j.ejop.2011.03.001

Zhang, Q., Song, H.-Y., Hu, Z.-Y 7 Liu, G.-X. (2015). Morphological examination and phylogenetic position ofthe newly recorded heterotrophic brackish dinoflagellate *Diplopsalis caspica* (Dinophyceae) in freshwater habitatfrom China. J. Syst. Evol. 53(6):512–519. doi: 10.1111/jse.12151

Zhang, Q., H., Zhu, Hu, Z. & Liu, G. (2016). Blooms of the woloszynskioid dinoflagellate *Tovellia diexiensis* sp. nov. (Dinophyceae) in Baishihai Lake at the eastern edge of Tibetan Plateau. Algae 31(3):205-217. http://dx.doi.org/10.4490/algae.2016.31.9.7

Zinssmeister, C., Soehner, S., Facher, E., Kirsch, M., Meier, K.J.S., Gottschling, M. (2011). Catch me if you can: The Who am I — and if so, how many? The taxonomic identity of *Scrippsiella trochoidea* (F.Stein) A.R.Loebl. (Thoracosphaeraceae, Dinophyceae). Systemat. Biodiver. 9:145–157. DOI: 10.1080/14772000.2011.586071

Zinssmeister, C., Soehner, S., Kirsch, M., Facher, E., Meier, K. J. S., Keupp, H. & Gottschling, M. (2012). Same but different: Two novel bicarinate species of extant calcareous dinophytes (Thoracosphaeraceae, Peridiniales) from the Mediterranean Sea. J. Phyol. 48(5):1107-18. DOI: 10.1111/j.1529-8817.2012.01182.x

Zou, J., Li, Q., Lu, S., Dong, Y., Chen, H., Zheng, C., Cui, L. (2020). The first benthic harmful dinoflagellate bloom in China: Morphology and toxicology of *Prorocentrum concavum*. Mar. Pollut. Bull. 158: 111313. https://doi.org/10.1016/j.marpolbul.2020.111313
